# Biologically engineered vectors enable topical mRNA delivery for skin regeneration

**DOI:** 10.1038/s42003-026-10651-9

**Published:** 2026-07-08

**Authors:** Julie Gérard, Héloïse Pilet, Charbel Bouez, Xun Du, Yong Chen, Yogeshvar N. Kalia, Pascal Clayette, Bruno A. Bernard, Nicolas Grandchamp

**Affiliations:** 1 GEG Tech, Office : 23 rue d’Anjou, Paris, France; 2 International Flavors & Fragrances, 521 W 57th St, New York, NY USA; 3 School of Pharmacy, Nantong University, 9 Seyuan Road, Nantong, Jiangsu Province China; 4 School of Pharmaceutical Sciences, University of Geneva, CMU - 1 rue Michel-Servet, 1211, Geneva, Switzerland; 5 Institute of Pharmaceutical Sciences of Western Switzerland, University of Geneva, CMU - 1 rue Michel-Servet, 1211, Geneva, Switzerland

**Keywords:** Molecular biology, Molecular medicine, Cell delivery

## Abstract

Messenger RNA therapeutics offer transient protein expression without altering the cellular genome, making them attractive for regenerative medicine, but efficient and safe delivery to the skin remains a major challenge. Lipid nanoparticles have transformed RNA delivery yet can remain limited in skin by tissue accessibility, formulation constraints and local tissue responses. Here we define Biologically Engineered Vectors (BEVs) as biologically assembled delivery particles generated through genetic or cellular engineering to transport therapeutic nucleic acids, and we develop RNA-BEVs as a platform for topical mRNA delivery. RNA-BEVs efficiently delivered functional mRNA to keratinocytes, induced transient protein expression throughout reconstructed epidermis after topical administration, and improved the histological quality of repair relative to mRNA-loaded lipid nanoparticles in a diabetic rat wound model. These findings establish RNA-BEVs as a biologically assembled platform for localized transient RNA delivery and skin regeneration.

## Abstract (web summary)

Biologically engineered vectors enable topical mRNA delivery to the epidermis and transient gene expression, improving histological repair in diabetic wounds and supporting localized RNA-based skin regeneration

## Introduction

Messenger RNA (mRNA) therapeutics are well-suited to regenerative applications because they enable transient protein expression without altering the cellular genome^1,2^. In skin, therapeutic translation is constrained by the challenge of achieving efficient and safe delivery to target cells across the epidermal barrier^3–5^. This challenge is especially relevant for cutaneous repair, where localized and temporally controlled expression of regenerative factors could be advantageous, yet keratinocytes have historically remained difficult to manipulate genetically^6–8^.

Most current RNA-delivery strategies rely on synthetic carriers such as lipid nanoparticles (LNPs), which have transformed systemic nucleic-acid delivery but can remain limited in skin by restricted tissue accessibility^5,9,10^, formulation-dependent performance^11–13^ and local tissue responses^14,15^. Alternative biologically assembled carriers have also emerged, including viral vectors^7,8,16^, engineered extracellular vesicles^17–19^, and virus-like particles^20–22^. However, viral vectors are generally optimized for durable gene transfer rather than transient expression and may raise concerns related to biosafety, genome integration or immunogenicity^23–26^. Other biologically derived systems have shown promise for cutaneous delivery but have thus far relied on intradermal administration and device-assisted deposition and can remain subject to practical constraints, including temperature sensitivity^27^. Although terms such as biological vectors, biogenic vectors, biomimetic vectors and biological nanoparticles have been used to describe biologically derived or biology-inspired delivery systems, these platforms are still often considered as distinct technologies despite shared biological assembly principles^28^.

Here, we use the term Biologically Engineered Vectors (BEVs) to delineate a unified class of biologically assembled delivery particles generated through genetic or cellular engineering and designed to transport therapeutic nucleic acid cargos. In contrast to broader terms that primarily refer to biological origin or biomimicry, BEVs are defined here by the combination of biological assembly and deliberate engineering of cargo, composition or functional properties. In this framework, BEVs include viral vectors, engineered extracellular vesicles, virus-like particles and related carriers derived from viral or cellular components. Within this class, we developed lentiviral particle-derived reverse-transcription-deficient BEVs, hereafter referred to as RNA-BEVs, as a viral BEV subtype engineered for transient mRNA delivery. By disabling reverse transcription, RNA-BEVs prevent RNA-to-DNA conversion and eliminate the risk of genomic integration while preserving efficient cellular entry. They encapsulate mRNA cargos for direct cytoplasmic delivery and show substantial room temperature stability, features that may be advantageous for localized regenerative applications.

Here, we asked whether RNA-BEVs could provide an effective platform for topical mRNA delivery to the skin. We show that RNA-BEVs deliver functional mRNA to keratinocytes, enable transient protein expression across reconstructed human epidermis after topical administration, and improve the histological quality of repair in a diabetic wound model.

## Results

### RNA-BEV morphology and titer

**Fig. 1:**
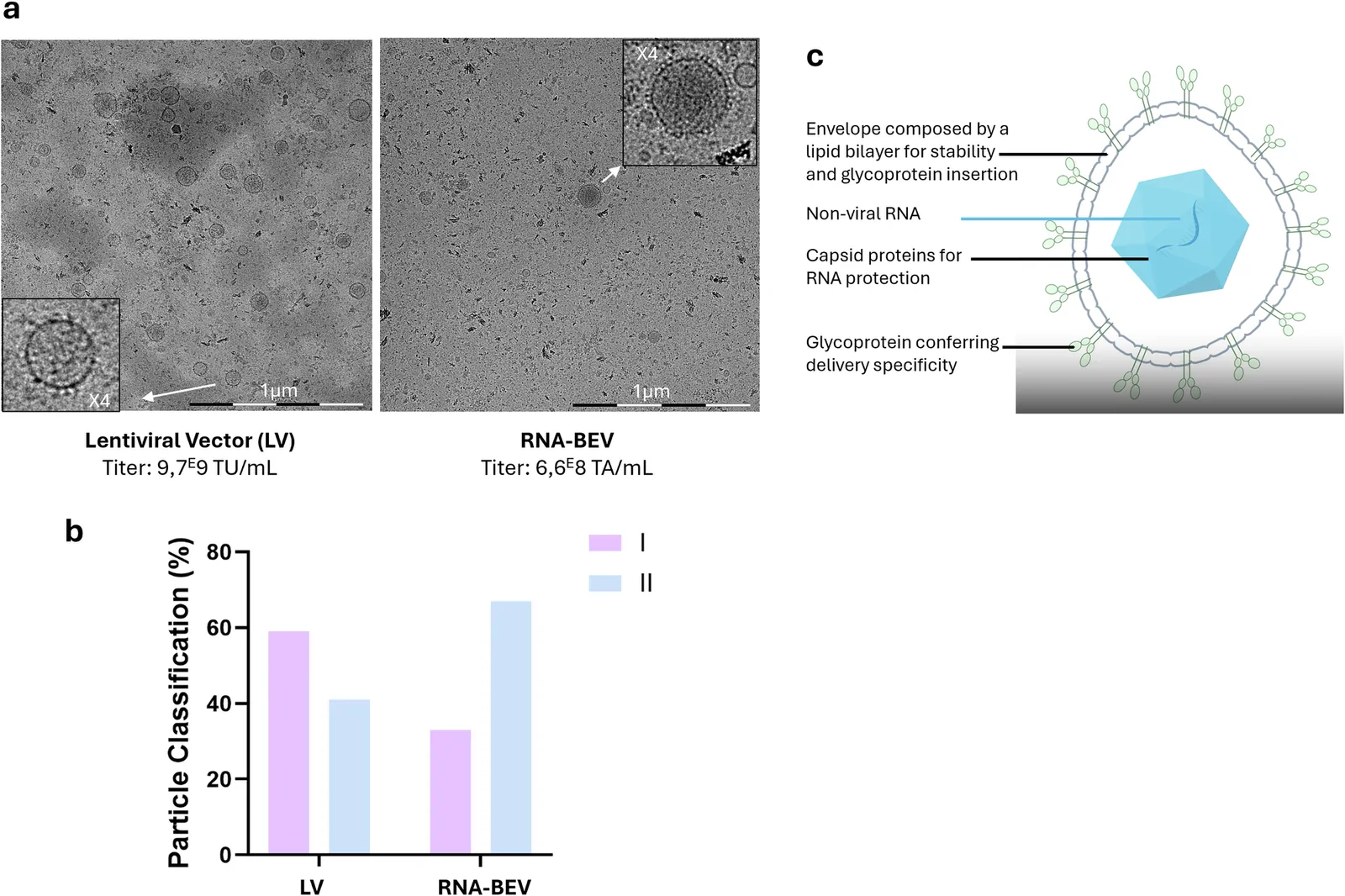
RNA-BEV morphology. **a** Morphology characterization of the nanoparticles using transmission electron microscopy (cryoTEM). The titer is expressed in transducing unit per ml for LV (TU/ml) or adsorbing unit per ml for RNA-BEV (AU/ml). **b** Quantification of nanoparticles in two groups: I- Nanoparticles having traceable spikes, displaying a distinct outer shell. II-Nanoparticles having no trace of spikes and that could not be unambiguously classified. Particle-class distribution was obtained from one cryoTEM analysis per vector type. Data are based on 25 images and 939 particles for LV, and 25 images and 567 particles for RNA-BEV. **c** Schema representing the RNA-BEV nanoparticle.

RNA-BEVs are lentivirus-derived BEV particles engineered to package mRNA while preventing RNA-to-DNA conversion through reverse-transcriptase inactivation, thereby supporting transient protein expression without genomic integration. We first assessed whether this reverse-transcriptase-deficient configuration affected particle formation or maturation by comparing RNA-BEV and conventional lentiviral vector morphology using cryo-transmission electron microscopy (CryoTEM). RNA-BEVs showed morphological features similar to conventional lentiviral particles, including capsid-like structures and envelope spike proteins (Fig. 1a). However, the proportion of mature particles classified in group I was lower for RNA-BEVs than for conventional lentiviral vectors, 33 versus 59%, respectively (Fig. 1b). This difference may contribute to the lower functional titer observed for RNA-BEVs.

### Transient expression and low toxicity

**Fig. 2:**
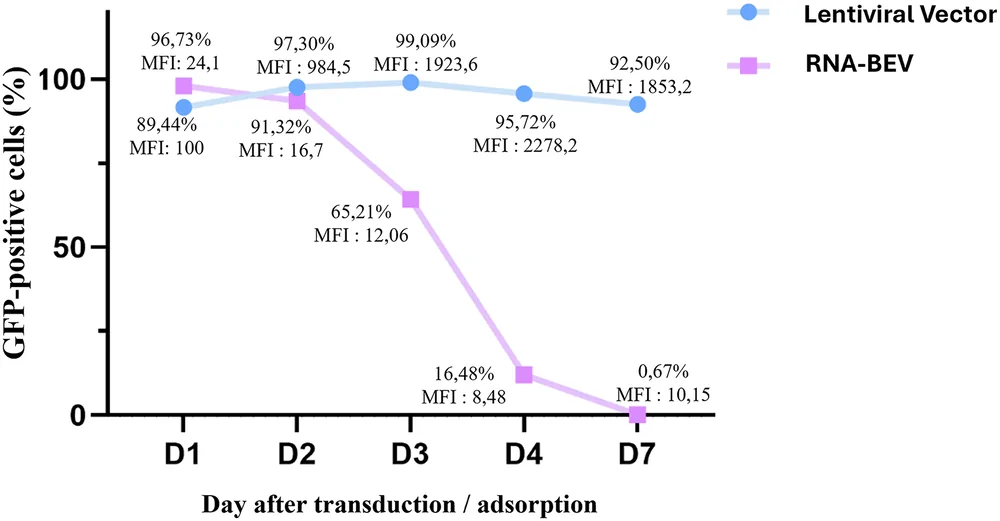
RNA-BEV Kinetics of GFP expression. Percentage of GFP expression in HEK-293T cells induced by an integrating lentiviral vector or RNA-BEV. MFI, median fluorescence intensity; values were normalized to lentiviral vector condition D1, set as 100, and are presented as relative fluorescence intensity across the RNA-BEV conditions. Data were shown from a single determination for the indicated condition and are presented descriptively; no error bars are shown because replicate measurements were not generated for this specific panel. Consistent results were obtained in independent experiments performed under related conditions.

Protein expression was assessed in HEK-293T cells using GFP as a reporter after exposure to RNA-BEV or to a conventional LV. Both vectors efficiently induced GFP expression, with more than 90% GFP-positive cells detected 24 h after treatment. RNA-BEV induced stronger early expression than the conventional LV, but this signal declined after 24 h and was completely lost by day 7. In contrast, GFP expression remained stable over the 7-day period following conventional LV treatment (Fig. 2).

**Fig. 3:**
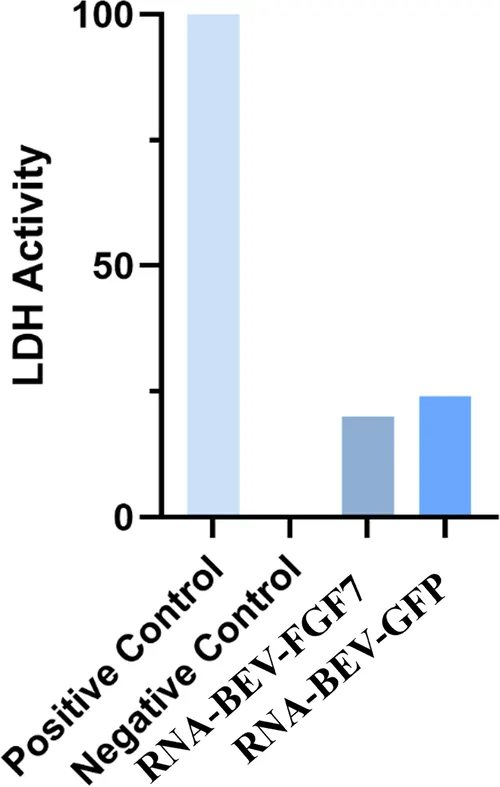
Evaluation of RNA-BEV toxicity. Level of Lactate Dehydrogenase activity after RNA-BEV addition coding FGF7 or GFP in HEK-293T cells. Negative control corresponds to a cell culture without a vector. Data were shown from a single determination for the indicated condition and are presented descriptively; no error bars are shown because replicate measurements were not generated for this specific panel. Consistent results were obtained in independent experiments performed under related conditions.

The potential cytotoxicity of RNA-BEV was assessed in the same HEK-293T cell model by measuring LDH release into the culture supernatant. RNA-BEV encoding either GFP or FGF7 was tested, as transient expression of FGF7 may be relevant for skin regeneration. Neither construct induced detectable membrane-damaging cytotoxicity under the tested conditions (Fig. 3). Together, these results show that RNA-BEV enables high yet transient expression of the encoded protein in HEK-293T cells without significant detectable membrane-damaging cytotoxicity^29–31^.

### Room temperature stability

**Fig. 4:**
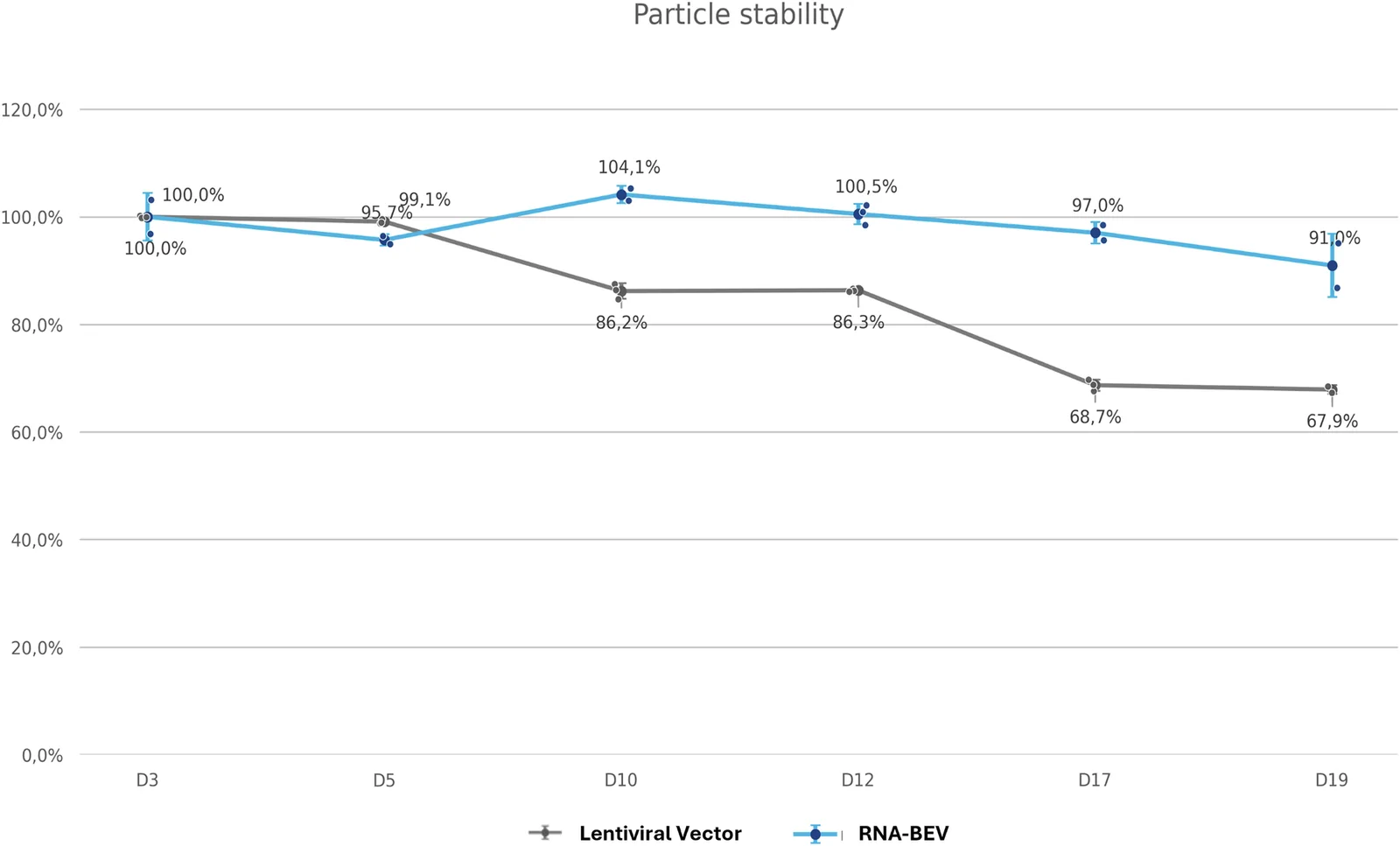
Stability of RNA-BEV at room temperature. Evaluation of the vector stability at room temperature (RT—19 °C). The vectors containing the GFP coding sequence were stored at RT for 19 days and their ability to induce GFP expression in HEK-293T is tested at several times. GFP expression is analyzed 48 h after GFP vectors addition (*n* = 3).

The long-term stability of vectors or nanoparticles is a key challenge since it influences the production and the availability of clinical-grade batches. Therefore, the exploration of RNA-BEV and LV particle stability was evaluated at room temperature (RT) in PBS 1X by comparing the GFP expression level in HEK-293T cells. The results showed a high stability for RNA-BEV, with a loss of only 9% at RT after 19 days (Fig. 4). Furthermore, RNA-BEV was more stable than a conventional LV, for which the efficacy decreased by 30% at RT during the same period.

### Delivery to primary keratinocytes

**Fig. 5:**
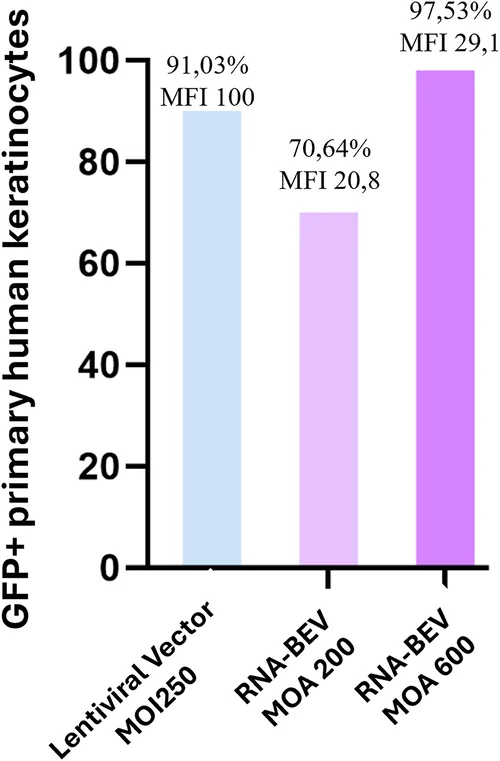
RNA-BEV efficacy to induce GFP expression in human primary keratinocytes. Representative experiment using Integrating lentiviral vector as a control with a multiplicity of infection (MOI) of 250 and RNA-BEV with a multiplicity of adsorption (MOA) of 200 and 600. MFI, median fluorescence intensity; values were normalized to the lentiviral vector condition, set as 100, and are presented as relative fluorescence intensity across the RNA-BEV conditions. GFP expression is analyzed 48 h after vector addition. Data were shown from a single determination for the indicated condition and are presented descriptively; no error bars are shown because replicate measurements were not generated for this specific panel. Consistent results were obtained in independent experiments performed under related conditions.

We investigated the ability and efficacy of the RNA-BEV vector to deliver mRNA in human primary keratinocytes to express a protein of interest. We used GFP with a conventional LV as a control. The results showed that the conventional LV induced GFP expression in 90% of keratinocytes 2 days post-infection with Multiplicity Of Infection (MOI) of 250. RNA-BEV was tested with two different doses, with a multiplicity of adsorption (MOA) of 200 and 600, respectively. The lower dose induced 70% of positive cells, while the higher dose led to nearly 100% of GFP-positive cells after 2 days. These representative data showed that RNA-BEV is a relevant vector to induce the expression of the protein of interest in human keratinocytes (Fig. 5).

### Promotion of keratinocyte migration

**Fig. 6:**
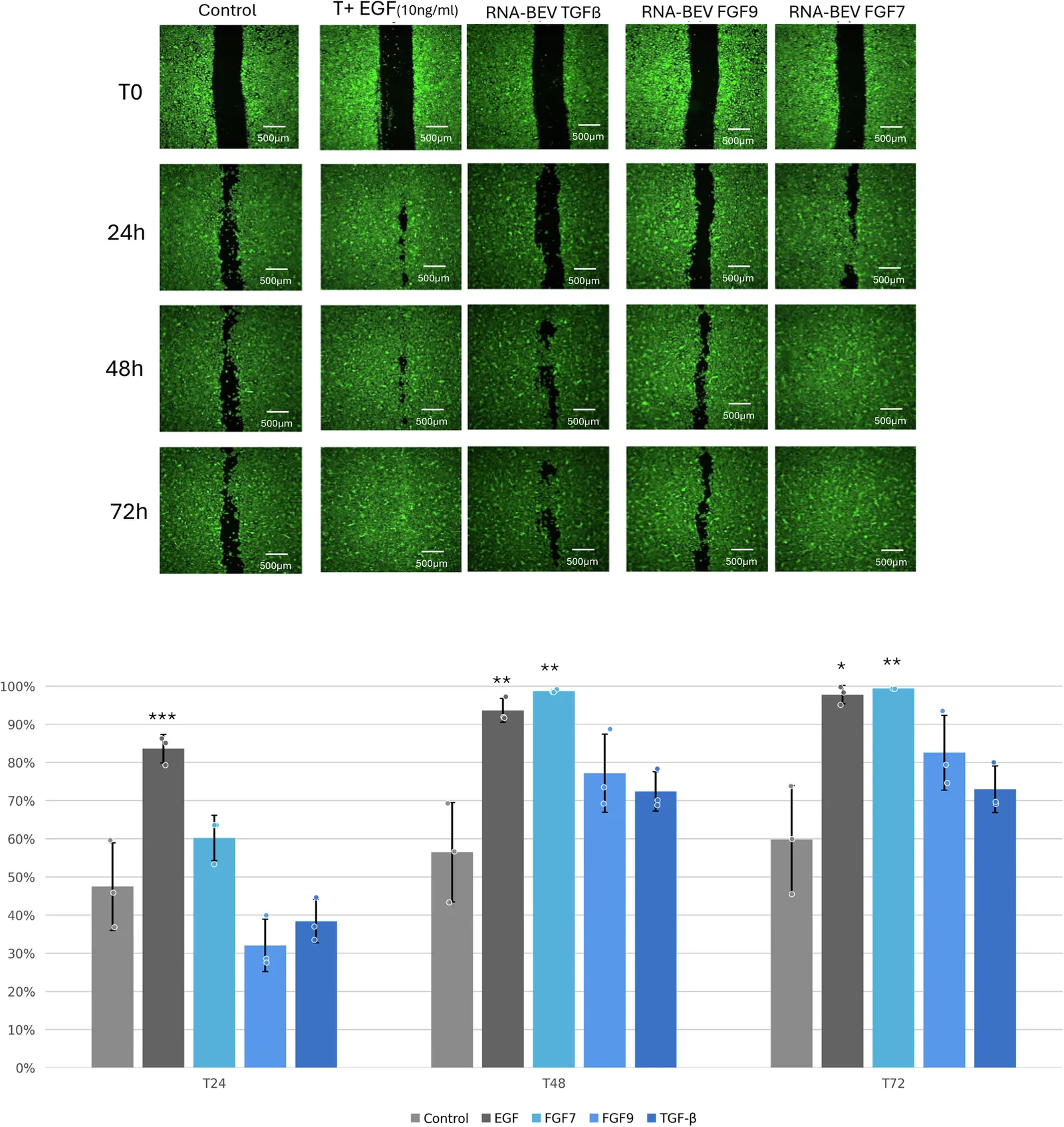
Effects of RNA-BEV inducing FGF7, FGF9 or TGF-ß expression on normal human epidermal keratinocytes (NHEKs) proliferation and migration. **a** Imagery analysis was performed 24, 48, and 72 h after scraping and RNA-BEV addition. **b** Quantification of wound closure using ImageJ. Data were presented as mean ± s.d. (*n* = 3). Statistical analysis was performed using two-way ANOVA followed by Šídák’s post hoc test for multiple comparisons. *p = 0.01 to 0.05, Significant **p = 0.001 to 0.01, highly significant ***p < 0.001, very highly significant.

We then investigated the ability of RNA-BEV to induce the expression of trophic factors and to promote skin healing using a scratch model of normal human epidermal keratinocytes (NHEKs), mimicking a human skin wound lesion. We tested the ability of three factors, FGF7, FGF9, and TGF-β, to stimulate wound closure when vectorized in the RNA-BEV system. In parallel, 10 ng/mL epidermal growth factor (EGF) was used as a positive control, since EGF is considered a reference pro-migratory factor in this wound-healing model. Results for RNA-BEV-FGF9 and RNA-BEV-TGF-β showed a trend favoring NHEK migration compared with the untreated control (Fig. 6). By contrast, RNA-BEV-FGF7 significantly increased NHEK migration, reaching a level higher than that observed with the EGF positive control.

### Topical delivery across reconstructed epidermis

**Fig. 7:**
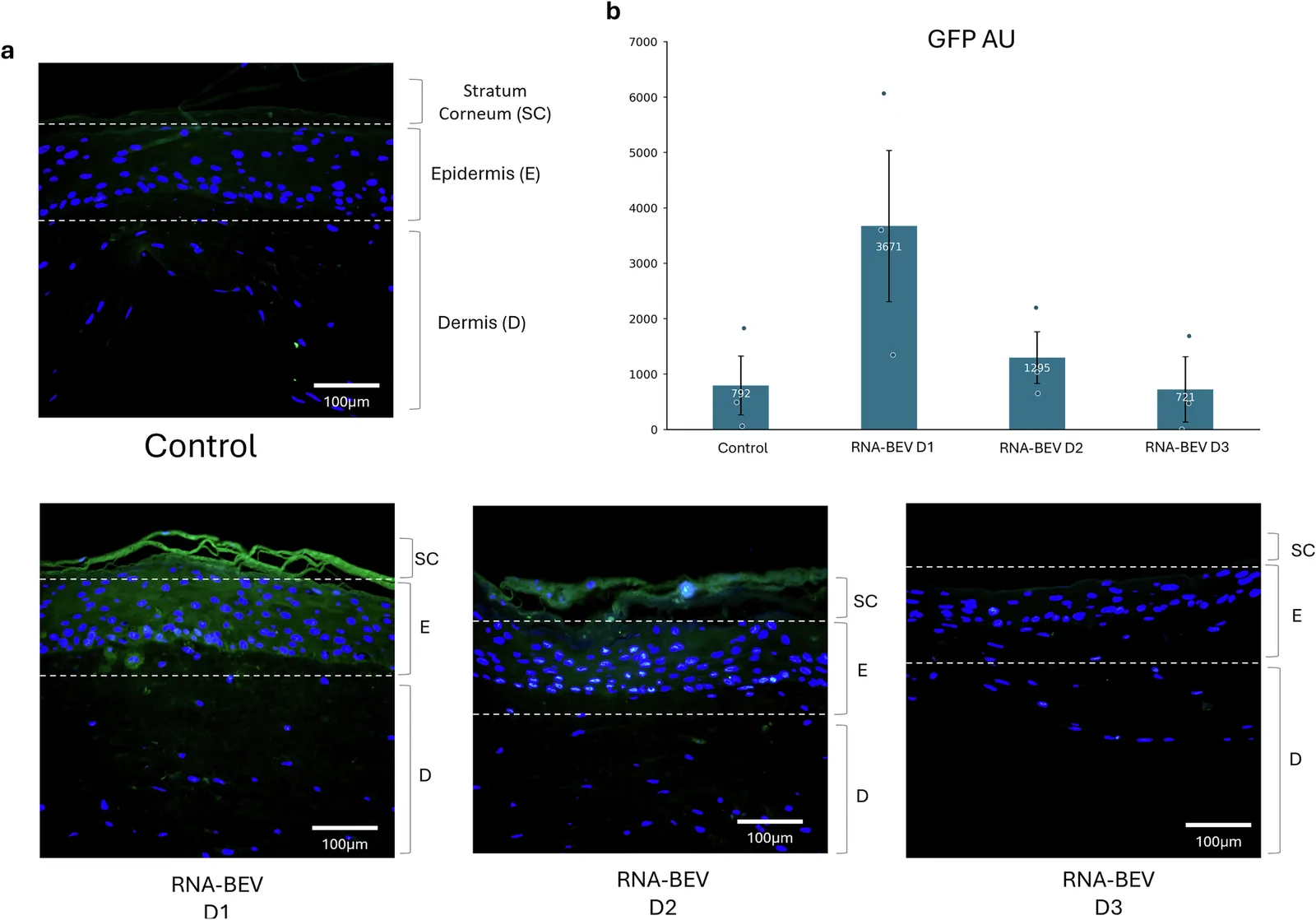
Biodistribution of GFP expression induced from RNA-BEV in full-thickness reconstructed Skin (FTSK). **a** Imagery analysis of FTSK is realized 24 h after RNA-BEV E GFP application. Nuclei were stained using Hoechst solution. Background autofluorescence was defined using untreated epidermis; the stratum corneum was excluded from quantification, and GFP signal was measured in the viable nucleated epidermis. **b** Graphical representation of the GFP penetration induced by RNA-BEV in FTSK with arbitrary units (AU). For each reconstructed skin sample, GFP fluorescence was averaged from three images. Data were presented as mean ± s.e.m. from three reconstructed skin samples per condition.

To determine whether RNA-BEVs could mediate topical delivery to epidermal tissue, we evaluated GFP expression in a reconstructed human skin model in vitro. RNA-BEV-GFP was applied topically, and untreated reconstructed skin samples imaged under identical acquisition settings were used to assess background fluorescence. Faint autofluorescence was detected in the stratum corneum of control samples (Fig. 7), consistent with the acellular nature of this superficial layer. Therefore, the stratum corneum signal was not interpreted as GFP expression and was excluded from fluorescence quantification. GFP-associated fluorescence was assessed within the viable nucleated epidermal compartment relative to the untreated control background. RNA-BEV-GFP induced detectable GFP expression throughout the viable reconstructed epidermis as early as day 1 post-treatment. Signal intensity progressively declined at days 2 and 3, consistent with transient mRNA expression kinetics. GFP expression followed a decreasing gradient from the upper viable epidermis toward the basal layer and was not detected in dermal cells.

### Improved diabetic wound repair

We next evaluated the therapeutic activity of RNA-BEV-FGF7 mRNA delivery in vivo in a streptozotocin-induced diabetic wound model and compared it with mRNA-FGF7 lipid nanoparticles (RNA-LNP-FGF7). Diabetic animals exhibited marked hyperglycemia relative to nondiabetic controls throughout the study period. Although fasting blood glucose levels decreased over time in diabetic groups, treated animals remained clearly separated from nondiabetic controls, and food and water intake profiles remained consistent with the diabetic phenotype. These observations indicate that the wound-healing effects observed here were not associated with normalization of the systemic metabolic state (Supplementary Fig. 1a–d).

Given the distinct architecture and intracellular delivery mechanisms of RNA-BEVs and LNPs, dose equivalence was not defined on the basis of nominal RNA molecule number. Instead, each formulation was evaluated within activity ranges compatible with topical administration and with the respective constraints of each delivery platform.

**Fig. 8:**
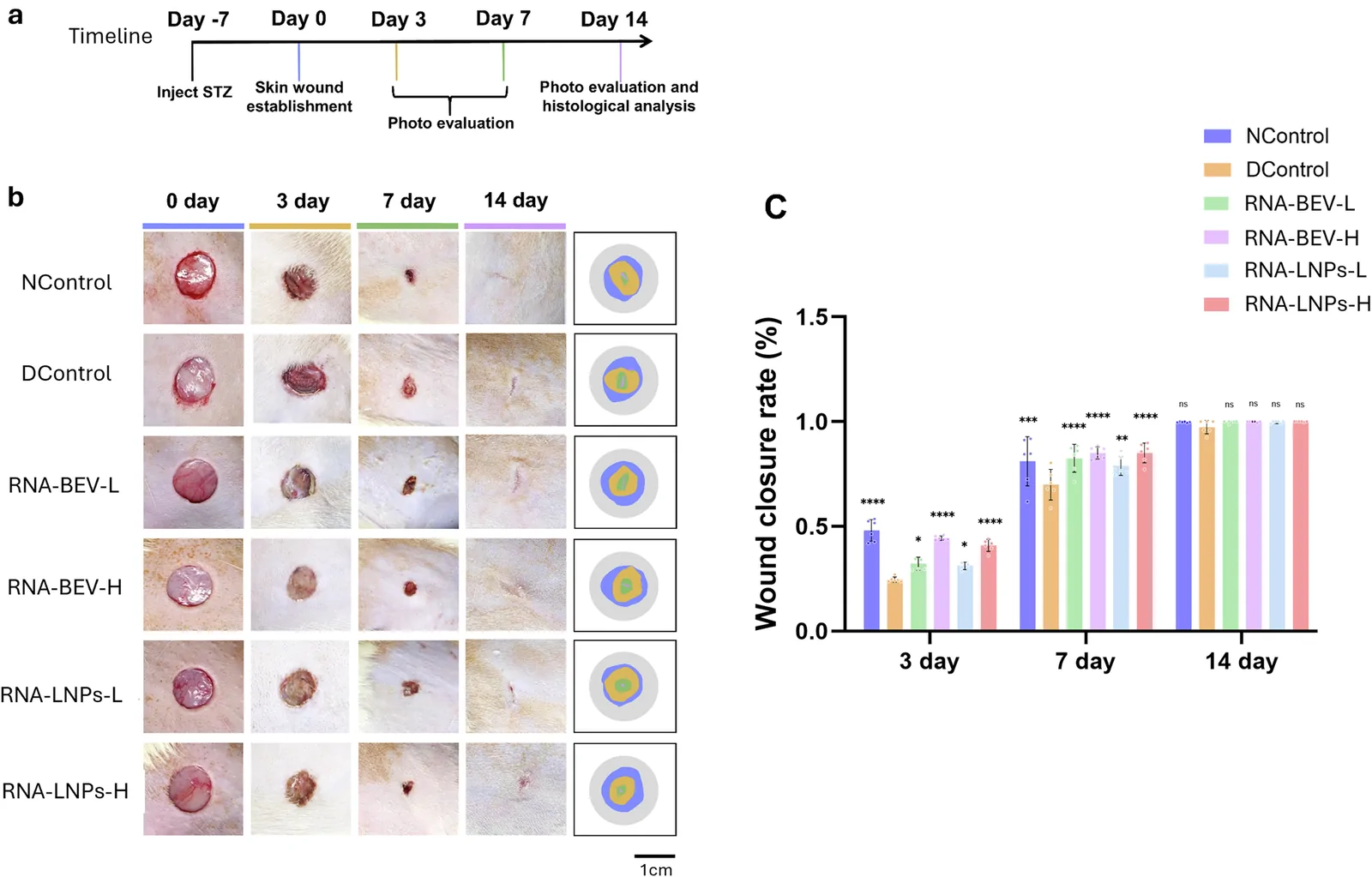
Wound-healing progression. **a** Schematic illustration of the operations on the rats in an order of time. **b** Representative photographs of wounds at days 0, 3, 7, and 14. **c** Representative photographs of the diabetic wound at day 0, 3, 7, and 14 treated with Ncontrol (nondiabetic rats), Dcontrol (diabetic rats), RNA-BEV-L (low dose), RNA-BEV-H (high dose), RNA-LNPs-L (low dose), and RNA-LNPs-H (high dose) group. Data were shown as mean ± SD (*n* = 6). Scale bar: 1 cm.

Macroscopic wound closure progressed in all groups over the 14-day observation period, with near-complete closure achieved by day 14 (Fig. 8). RNA-BEV-FGF7 treatment was associated with faster early wound closure kinetics, most notably in the high-dose group at day 7. However, under the conditions tested, macroscopic closure was broadly comparable between RNA-BEV-FGF7 and LNP-FGF7 groups by the end of the study, indicating that gross wound size alone did not fully capture treatment-related differences in repair quality.

**Fig. 9:**
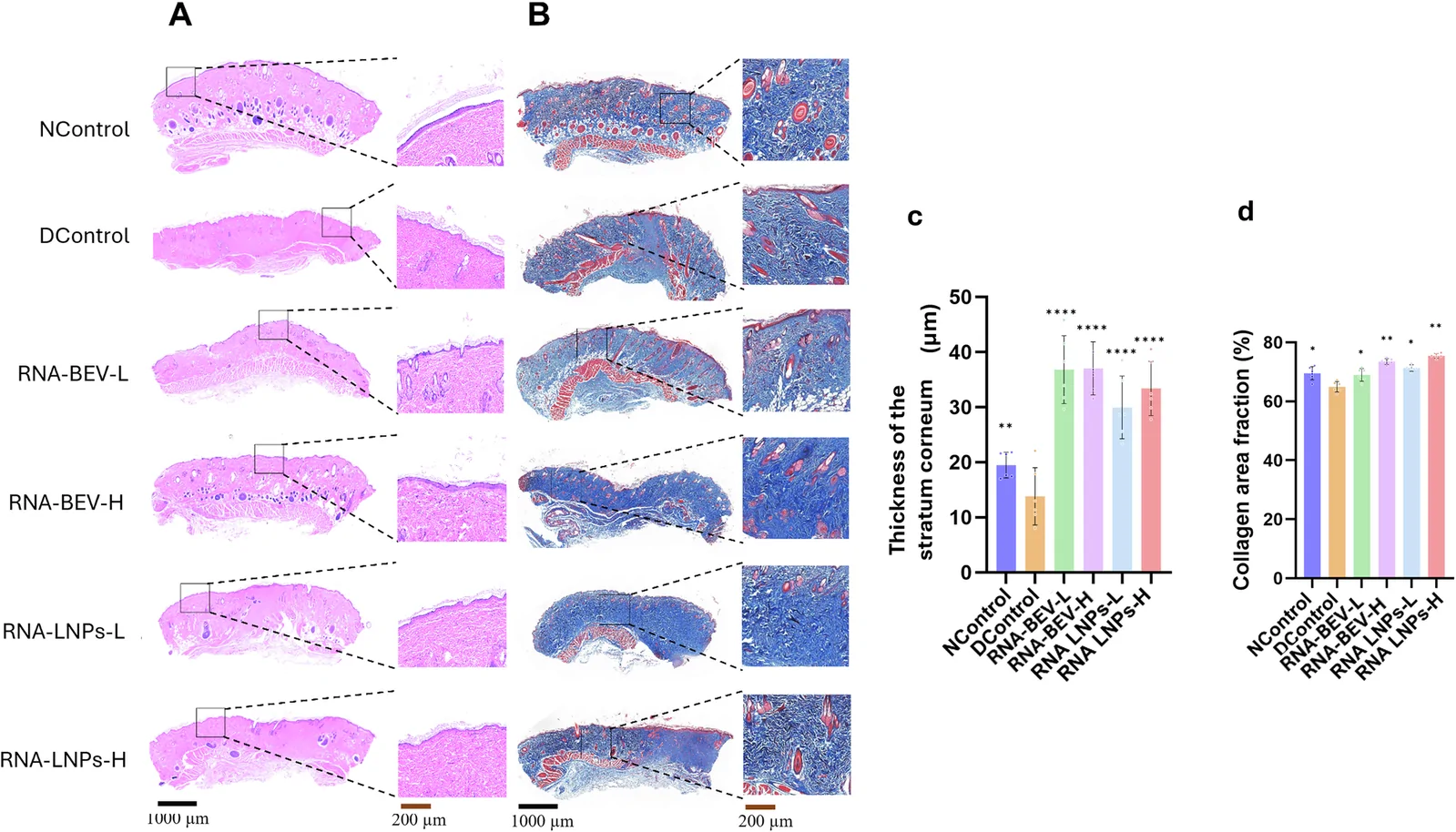
Histological evaluation of epidermal regeneration. **a** Representative HE staining of wound sections at day 14. **b** Representative Masson’s trichrome staining at day 14. **c** Quantitative analysis of epidermal thickness. Data shown as mean ± SD (*n* = 6). **d** Quantification of collagen area fraction. Data were shown as mean ± SD (*n* = 6). scale bar = 1000 µm (black) or = 200 µm (brown).

Histomorphometric analyses nevertheless revealed clear differences in tissue regeneration. Diabetic control wounds showed reduced epidermal thickness relative to nondiabetic skin, whereas both RNA-BEV-FGF7 and LNP-FGF7 treatment increased epidermal thickness. At both tested dose levels, epidermal thickness was greater in RNA-BEV-FGF7-treated wounds than in the corresponding LNP-FGF7-treated groups (Fig. 9a, c). Masson’s trichrome staining further showed impaired collagen organization in diabetic controls, characterized by sparse and fragmented fibers, whereas both delivery platforms improved collagen density and matrix organization at day 14 (Fig. 9b, d).

**Fig. 10:**
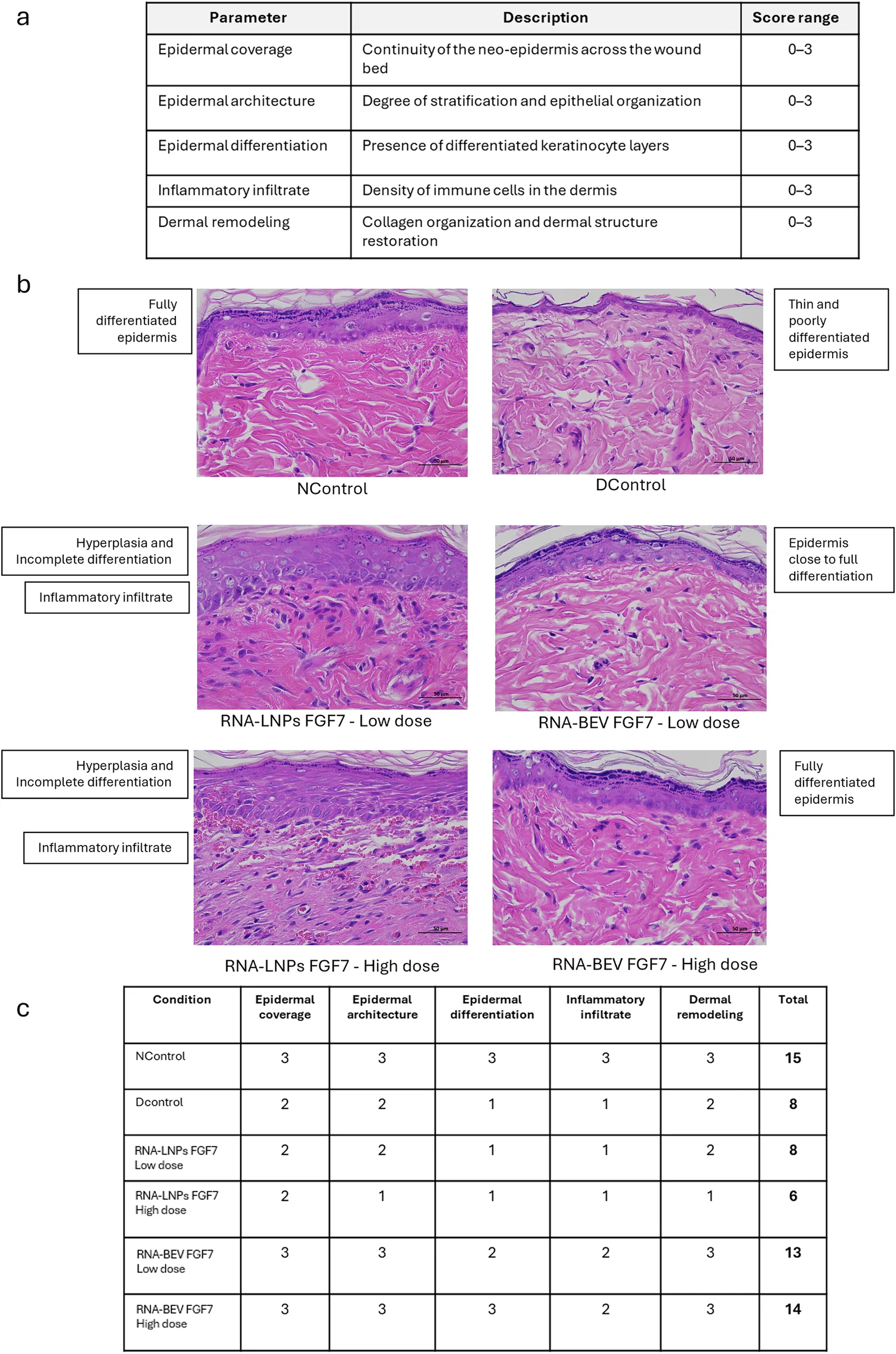
RNA-BEV treatment improves the histological quality of wound repair in diabetic skin. **a** Semi-quantitative histological scoring framework based on epidermal coverage, epidermal architecture, epidermal differentiation, inflammatory infiltrate, and dermal remodeling. Each parameter was scored from 0 to 3, yielding a maximum composite score of 15. **b** Representative H&E-stained wound sections collected at day 14 from nondiabetic controls (NControl), diabetic controls (DControl), and diabetic animals treated with low- or high-dose RNA-LNPs FGF7 or RNA-BEV FGF7. **c** Composite histological scores calculated from the five parameters shown in (**a**). RNA-BEV FGF7 low-dose and high-dose conditions yielded higher scores than RNA-LNPs FGF7 low-dose and high-dose conditions. The high-dose RNA-LNPs FGF7 condition scored lower than the low-dose condition. Representative H&E image sets for all groups at days 0 and 14 are shown in Supplementary Figs. 2 and 3. Scale bars, 50 μm. Scores were calculated from *n* = 6 animals per group.

These differences were more apparent on qualitative and semi-quantitative histological assessment. Using a predefined five-parameter scoring framework encompassing epidermal coverage, epidermal architecture, epidermal differentiation, inflammatory infiltrate and dermal remodeling, HE-stained day-14 wound sections were compared across treatment groups (Fig. 10a). Diabetic control wounds formed a thin and poorly differentiated epidermis associated with inflammatory infiltrates in the upper dermis. RNA-LNP-treated wounds showed partial re-epithelialization with epidermal hyperplasia and incomplete differentiation, whereas RNA-BEV-FGF7-treated wounds displayed a more continuous and differentiated epidermis with reduced inflammatory features (Fig. 10b). Composite histological scores were substantially higher for RNA-BEV-FGF7 low dose and high dose than for RNA-LNP-FGF7 low dose and high dose (13 and 14 versus 8 and 6, respectively), with RNA-BEV-FGF7-treated wounds approaching the nondiabetic control condition (Fig. 10c). Notably, increasing the LNP dose did not improve histological outcome and instead yielded a lower composite score than the low-dose LNP condition, together with poorer epidermal architecture and dermal remodeling, indicating a non-monotonic and potentially dose-limited response of the LNP formulation in this model. Representative H&E image sets for all groups at days 0 and 14 are provided in Supplementary Figs. 2 and 3.

Together, these findings indicate that RNA-BEV-mediated FGF7 mRNA delivery improves the histological quality of wound repair in diabetic skin, with more complete epidermal regeneration and fewer inflammatory features than observed with FGF7 mRNA LNP delivery. The poorer outcome observed with the high-dose LNP condition further identifies an important limitation of the LNP formulation in this setting.

## Discussion

Efficient and safe delivery of messenger RNA (mRNA) to epithelial tissues remains a major challenge for regenerative RNA therapeutics^1,2,5^. Reverse-transcription-deficient biologically engineered vectors (RNA-BEVs) combine a biologically assembled particle architecture with a non-integrating RNA payload, enabling localized and transient protein expression without RNA-to-DNA conversion. In addition, RNA-BEVs retained functional activity after room temperature storage, a property that may reflect their reliance on direct delivery and translation of packaged mRNA rather than on reverse-transcription-dependent post-entry steps required for conventional lentiviral vector activity. In the present study, we show that this platform enables topical delivery of functional mRNA to epidermal keratinocytes, resulting in protein expression throughout reconstructed human epidermis and improved repair in a diabetic wound model. Together, these findings support RNA-BEVs as a platform for regenerative mRNA delivery at epithelial surfaces.

Among biologically engineered vectors (BEVs), viral vectors have historically been the dominant and most mature delivery systems, owing to their efficient cellular entry and gene-transfer capabilities^32–34^. However, these systems are generally optimized for durable genetic modification rather than transient expression and can present constraints related to genome integration, or immunogenicity, depending on the application^23–26^. More recently, advances in synthetic biology and cellular engineering have broadened the BEV landscape to include engineered extracellular vesicles, virus-like particles and hybrid biological assemblies^17,20,27^. In this context, BEVs provide a useful conceptual framework for biologically assembled nucleic-acid delivery systems that share common assembly principles while differing in architecture, persistence and intracellular fate.

Within this framework, RNA-BEVs derive from the concept of reverse-transcription-deficient retroviral RNA-vector systems, in which retrovirus-derived particles unable to complete reverse transcription mediate transient expression from delivered RNA^35,36^. Here, this platform was refined and adapted as a lentivirus-derived BEV format for regenerative mRNA delivery and topical skin administration. By preventing RNA-to-DNA conversion while preserving efficient cell entry, RNA-BEVs support transient mRNA expression without generating a DNA intermediate required for genomic integration. Topical RNA-BEV-GFP application to reconstructed human epidermis induced GFP expression within the viable epidermal compartment without experimental wounding, supporting epidermal access in vitro. The in vivo wound model should nevertheless be interpreted in the context of epithelial repair, where barrier disruption may facilitate topical access for both RNA-BEVs and RNA-LNPs. Under these conditions, RNA-BEVs enabled effective regenerative mRNA delivery and improved histological repair compared with the LNP comparator. The biological activity observed with RNA-BEVs, despite lower nominal RNA input, further suggests that particle architecture may influence the fraction of mRNA that becomes functionally available intracellularly, although the intracellular trafficking and release steps underlying this effect remain to be defined.

Importantly, the principal in vivo benefit of RNA-BEV-FGF7 treatment appears to be an improvement in the quality of tissue repair rather than a simple increase in macroscopic wound closure. Histological analysis showed that RNA-BEV-FGF7-treated wounds had more complete epidermal coverage, improved epithelial architecture, greater differentiation, reduced inflammatory infiltrate and improved dermal remodeling. Composite histological scores for both RNA-BEV-FGF7 dose levels approached those of nondiabetic controls and exceeded those of both LNP-FGF7-treated groups, indicating that RNA-BEV delivery improved several features of diabetic wound repair in parallel rather than a single endpoint.

By contrast, increasing the dose of RNA-LNP-FGF7 did not improve histological outcome and was associated with a lower composite score than the low-dose LNP condition. This non-monotonic response is notable, as it indicates that increasing nominal RNA input did not translate into improved tissue organization in this model. Whether this reflects formulation-dependent tissue effects, altered local exposure to FGF7, or broader dose-related constraints of the LNP platform remains unclear. Nevertheless, this finding highlights a limitation of the LNP-based approach in this setting and strengthens the contrast with the more favorable profile observed with RNA-BEVs.

The histological pattern observed after RNA-BEV-FGF7 treatment also suggests that mRNA expression may shape the local wound environment in ways that support regeneration. Compared with LNP-treated wounds, RNA-BEV-FGF7-treated tissues showed a more continuous and differentiated epidermis together with reduced inflammatory features. These findings are consistent with a tissue context that is more permissive to repair, although the present study does not establish the underlying mechanisms. In this regard, transient local expression of trophic factors such as FGF7 in keratinocytes may support epithelial restoration while limiting prolonged or systemic exposure^37,38^.

The ability to achieve functional mRNA delivery across the cornified barrier by topical administration is relevant for cutaneous RNA therapeutics^5^, where many current approaches still rely on invasive delivery routes or indirect targeting strategies^5,9,27,39^. A platform that enables localized gene expression in the epidermis may therefore be particularly valuable for chronic skin disorders and hard-to-heal wounds.

From a translational perspective, these findings are of interest for chronic wounds, including diabetic ulcers, which are characterized by impaired epithelial regeneration, persistent inflammation and defective tissue remodeling^40–42^. The improvement in epidermal continuity, differentiation, and dermal organization observed with RNA-BEV-FGF7 treatment suggests that this BEV format may address several pathological features of diabetic wound healing simultaneously. Topical administration may also facilitate repeated local treatment without invasive delivery. The prolonged functional stability of RNA-BEVs at room temperature further supports the translational potential of this platform by indicating that biologically assembled RNA delivery can be coupled to functional robustness under practically relevant conditions.

This study has several limitations. First, the intracellular trafficking, unpackaging and release mechanisms that determine RNA-BEV-mediated mRNA delivery remain to be characterized. Second, direct dose equivalence between RNA-BEVs and LNPs could not be established because these platforms differ fundamentally in architecture, intracellular routing and functional RNA bioavailability. Third, the unfavorable trend observed with high-dose LNP treatment highlights the importance of dose–response relationships and local tissue compatibility when comparing delivery platforms. Although inflammatory mediators were not directly quantified in this study, formulation-dependent immunostimulatory properties of LNPs may plausibly contribute to histological differences between delivery platforms, without establishing a direct causal mechanism^14,43^. Finally, validation in large-animal models and human tissues will be required to further assess translational potential.

Taken together, our results show that RNA-BEV-mediated mRNA delivery enables localized and transient gene expression in epidermal keratinocytes and improves the histological quality of repair in diabetic skin. More broadly, these findings support BEVs as a useful conceptual framework for biologically assembled nucleic-acid delivery systems and identify reverse-transcription-deficient lentiviral particles as a promising platform for epithelial RNA therapeutics, complementary to existing synthetic nanoparticle strategies.

## Methods

### Vector production and quantification

Integrating LVs were generated by the transient transfection of HEK-293T cells using the calcium phosphate precipitation method. Cells were co-transfected with the vector plasmid (pTrip-CMV-GFP-WPRE), the transcomplementation plasmid p8.91 IN_WT_, and the plasmid encoding the VSV envelope glycoprotein (pMD-G)^44^. Cell culture supernatant was collected 48 hours after transfection, treated with DNaseI (Roche) and filtered (0.45 µm). Vector particles were then concentrated by ultracentrifugation (90 min, 22,000 rpm, Beckman SW28 rotor) and resuspended in 0.1 M phosphate buffer saline (PBS) solution.

RNA-BEV vectors were generated by the transient transfection of 293T cells using the calcium phosphate precipitation method. Four RNA-BEV candidates were generated vectorizing either the Green Fluorescence Protein (GFP), the Fibroblast growth factors (FGF) -7 and -9 or the Transforming growth factor (TGF-ß) -ß. To this end, cells were co-transfected with the vector plasmid (pGFP-WPRE; pFGF7-WPRE; pFGF9-WPRE; and pTGF-ß-WPRE) containing an RNA Booster sequence^45^, the transcomplementation plasmid p8.91 IN_D100E_ containing a mutation of reverse transcriptase inactivating the reverse-transcription process^36^, and the plasmid encoding the VSV envelope glycoprotein (pMD-G). Cell culture supernatant was collected 48 h after transfection, treated with DNaseI (Roche) and filtered (0.45 µm). Vector particles were then concentrated using the same experimental protocol previously described for LV.

Vector titers were determined using GFP-expressing constructs. For LVs, 80,000 HEK-293T cells were transduced with serial dilutions, and titers were expressed as transducing units per mL (TU/mL). For RNA-BEV particles, cells were exposed to serial dilutions under identical conditions, and titers were expressed as adsorbing units per mL (AU/mL), defined as the number of particles capable of inducing detectable GFP expression per target cell population under standardized conditions.

Vectors lacking GFP were quantified by one-step qRT-PCR (QuantStudio™ 7 Pro, Applied Biosystems) using the CellsDirect™ One-Step qRT-PCR Kit. Quantification was performed relative to GFP-expressing reference vectors to estimate functional TU or AU equivalents.

### Cell culture

HEK-293T cells and human primary keratinocytes were obtained from ATCC. HEK-293T cells were grown in Dulbecco’s modified medium supplemented with 100 U/mL penicillin, 100 μg/mL streptomycin, and 10% heat-inactivated fetal calf serum (56 °C for 45 min). Human primary keratinocytes were grown in Dermal Cell Basal Medium (ATCC PCS-200-030) supplemented with Keratinocyte Growth Kit (ATCC PCS-200-040). Cells were routinely tested for mycoplasma contamination and were negative. No additional authentication was performed by the authors after receipt.

### CryoTEM

CryoTEM imaging and image analysis were performed by QuTEM AB (Stockholm, Sweden). The morphology of the RNA-BEV vector was assessed by Cryo-transmission electron microscopy (CryoTEM). Vector samples were deposited onto a thin supporting film of carbon in a temperature and humidity-controlled environment. Excess sample was removed in a blotting process, leaving a thin film of specimen on the grid. The grid is subsequently vitrified in liquid ethane and then stored in liquid nitrogen. CryoTEM analysis of vectors is utilized to assess the overall specimen morphology and size. The images were analyzed using semi-automatic particle detection. Only particles located within image boundaries and displaying well-defined edges were included. Particles smaller than 50 nm and aggregated particles were excluded from classification. Detected particles were classified as high-density lentiviral particles (HDP), low-density lentiviral particles (LDP), liposome-like particles (LLP), or uncertain particles (UNT), according to morphology, spike visibility and internal density. For Fig. 1b, one cryoTEM analysis was performed per vector type, with 25 images analyzed and 939 particles classified for the conventional lentiviral vector, and 25 images analyzed and 567 particles classified for RNA-BEV.

### LDH assay

The lactate dehydrogenase assay (LDH) colorimetric kit (Invitrogen—CyQUANT C20300) was used to control the cell viability of HEK-293T using the provider’s recommendations. The optimal cell number for this LDH assay under our experimental conditions was previously defined as 10,000 cells per well, and absorbances at 490 and 680nm were measured sequentially in time using the Multiskan SkyHigh Microplate reader (Thermo Fisher Scientific, Les Ulis, France).

### Keratinocyte migration assay

The study was led by QIMA Bioalternatives (Gençay, France) using the NHEK-0244 model. Keratinocytes were seeded on a fibroblast-populated collagen lattice in 24-well plates and 600 µl cell culture medium per well. A mechanical scraping was performed, and cells were labeled with calcein-AM. RNA-BEV (3E10 particles) or EGF (10 ng/ml) was added to keratinocyte cell cultures for 5 h and then, at 0, 24, 48, and 72 h, the cell migration area was monitored with a high-resolution imaging system (INCell Analyzer™2200 microscope, GE Healthcare Life Sciences/x4 objective). The artificial wound surface (i.e., cell-free area) was analyzed using the ImageJ software and surfaces obtained at 24, 48, and 72 h of culture were reported to the initial surface measured at 0 h. The effect of RNA-BEV on keratinocyte migration was compared to that of the untreated keratinocyte control. All the conditions were tested in triplicate (*n* = 3). Statistical analysis was performed using two-way ANOVA followed by Šídák’s post hoc test for multiple comparisons.

### Reconstructed skin model

This full-thickness reconstructed skin (FTSK) model (QIMA Bioalternatives) consisted of human primary keratinocytes seeded on a fibroblast-populated collagen lattice (dermis equivalent) and cultured at the air/liquid interface to obtain a reconstructed epidermis. Seven-day-differentiated FTSK were placed at 37 °C in 5% CO_2_ in six-well cell culture plates containing cell culture medium (QIMA Bioalternatives differentiation medium) and were topically treated or not (control 72 h) with RNA-BEV (4 × 10^6^ Units of RNA-BEV). These FTSK cultures, performed in triplicate (*n* = 3), were then maintained for 24, 48, and 72 h.

At the end of the incubation, FTSKs were washed in phosphate-buffered saline (PBS) solution, rinsed and fixed using a formaldehyde solution. The fixed samples were dehydrated in multiple baths containing increasing concentrations of ethanol and then embedded in paraffin. Transversal sections were produced using a microtome (5-μm thickness) and stored at room temperature until analysis.

The sections were deparaffinized, and the nuclei were stained using Hoechst solution 33258 (bisbenzimide). The sections were washed in a PBS-Tween 0.05% solution and mounted in “fluorescent mounting medium”.

The sections were observed using a Nikon microscope. Images were captured using a Nikon camera and processed with the NIS-Elements software. Three images per replicate were captured (objective lens x40).

The fluorescence intensity of GFP was measured on the captured images with ImageJ software (9 values per treatment condition). For the analysis, the results were normalized to the epidermal surface.

### Diabetic wound model

#### Animals

Male Sprague-Dawley rats (initial body weight 220 ± 10 g, aged 8 weeks) were obtained from the Animal Center of Nantong University. Animals were housed under controlled conditions (12-h light/dark cycle, 22 ± 2°C, 50 ± 10% humidity) with ad libitum access to standard rodent chow and water. All experimental procedures, including the anesthetic regimen used for wound creation, were approved by the Institutional Animal Care and Use Committee of Nantong University (Approval No. S20250522-004) and conducted in accordance with the National Institutes of Health Guide for the Care and Use of Laboratory Animals. We have complied with all relevant ethical regulations for animal use.

#### Induction of diabetes

After 1 week of acclimatization, diabetes was induced by intraperitoneal injection of streptozotocin (STZ; Sigma-Aldrich, USA) at 65 mg/kg body weight, dissolved in ice-cold 0.1 M citrate buffer (pH 4.5). Rats were fasted for 6 h prior to injection. Blood glucose levels were monitored for one week using a glucometer (Sinocare, China). Rats with fasting blood glucose (FBG) levels consistently ≥16.6 mmol/L (300 mg/dL) were included as diabetic models. During the wound-healing experiments, body weight was also measured daily. Food and water intake were recorded every 24 h. Blood glucose levels were measured from the tail vein on days 0 (baseline), 3, 7, and 14 using a commercial glucometer.

#### Wound creation and treatment

Rats were anesthetized with intraperitoneal injection of pentobarbital sodium (40 mg/kg). The dorsal hair was shaved and disinfected with 70% ethanol. A full-thickness circular wound (10 mm of diameter) was created using a sterile biopsy punch. Treatments were applied topically to the wound bed immediately after injury.

#### Experimental design and grouping

Diabetic rats were randomly assigned to six experimental groups (*n* = 6 per group):

- NControl: Nondiabetic rats + wound + 100 µL PBS (pH 7.4)
- DControl: Diabetic rats + wound + 100 µL PBS (pH 7.4)
- RNA-BEV-L: Diabetic rats + wound + low-dose RNA-BEV/1.4 × 10^6^ units/(5 µL in 95 µL PBS)
- RNA-BEV-H: Diabetic rats + wound + high-dose RNA-BEV/4.2 × 10^6^ units/(15 µL in 85 µL PBS)
- RNA-LNPs-L: Diabetic rats + wound + low-dose RNA-LNPs/0.5 µg/(5 µL in 95 µL TBS/10% sucrose)
- RNA-LNPs-H: Diabetic rats + wound + high-dose RNA-LNPs/1.5 µg/(15 µL in 85 µL TBS/10% sucrose)

FGF7-encoding mRNA was encapsulated in lipid nanoparticles (RNA-LNPs) by RiboPro (Brussels, Belgium) using its proprietary formulation process. The RNA-LNPs were supplied as ready-to-use particles and used as an external comparator in this study. Detailed lipid identities, lipid molar ratios and manufacturing parameters were not disclosed by the provider. According to the batch characterization provided, the particles had an average size of 120 ± 10 nm, a polydispersity index below 0.2, and mRNA encapsulation was confirmed using a RiboGreen-based assay.

Given the distinct structural organization and intracellular delivery pathways of RNA-BEV and RNA lipid nanoparticles (LNPs), direct dose equivalence based solely on nominal RNA copy number may not accurately reflect biologically effective exposure.

For LNP formulations, administered RNA mass represents total encapsulated RNA but does not necessarily correspond to intracellularly released and translationally competent RNA, as delivery efficiency is influenced by encapsulation efficiency, cellular uptake, endosomal escape, and degradation kinetics. Based on molecular weight calculations (250,368 g·mol⁻¹), 1 µg of FGF7 mRNA corresponds to ~2.4 × 10¹² theoretical RNA molecules; however, this value represents nominal input rather than effective cytoplasmic availability.

RNA-BEV particles encapsulate defined RNA cargo within a protein capsid and envelope architecture designed to facilitate cellular adsorption and cytoplasmic delivery. Each particle contains two RNA molecules, although functional intracellular exposure depends on adsorption efficiency and internalization dynamics. Estimated nominal RNA inputs were 2.8 × 10⁶ and 8.4 ×  10⁶ molecules for low and high doses, respectively.

Accordingly, dose selection for comparative in vivo evaluation was based on biologically optimized and administrable formulation volumes rather than theoretical RNA molecule equivalence. This strategy enables platform-relevant comparison under translationally realistic conditions while acknowledging that nominal RNA input does not directly equate to effective intracellular bioavailability across delivery systems.

#### Wound-healing assessment

Wounds were photographed on days 0, 3, 7, and 14 using a digital camera under standardized lighting and distance conditions. Wound area was quantified using ImageJ software (NIH, USA). The percentage of wound closure was calculated as $[({A}_{0}-{A}_{t})/{A}_{0}]\times 100$, where ${A}_{0}$ is the initial wound area and ${A}_{t}$ is the wound area at time t.

#### Histology and scoring

Wound tissues were harvested on days 0 and 14 with a 2-mm margin of surrounding skin. Tissues were fixed in 4% paraformaldehyde for 24 h, dehydrated through graded ethanol series, embedded in paraffin, and sectioned at 5-µm thickness.

Sections were deparaffinized, rehydrated, and stained with hematoxylin and eosin according to standard protocols. Epidermal thickness was measured using ImageJ by drawing perpendicular lines from the epidermal surface to the dermal-epidermal junction at ten randomly selected locations per section.

H&E-stained wound sections collected at day 14 were scored using a predefined semi-quantitative rubric based on five parameters: epidermal coverage, epidermal architecture, epidermal differentiation, inflammatory infiltrate and dermal remodeling. Each parameter was assigned a score from 0 to 3, with higher scores indicating a more advanced or more organized repair pattern, yielding a maximum composite score of 15 per section. Epidermal coverage was scored as follows: 0, absence of re-epithelialization; 1, partial and discontinuous epidermal coverage; 2, continuous but incomplete or immature coverage; and 3, complete re-epithelialization. Epidermal architecture was scored as follows: 0, absent or severely disorganized epidermis; 1, poorly organized epidermis; 2, partially restored stratification; and 3, near-normal stratified architecture. Epidermal differentiation was scored as follows: 0, absent differentiation; 1, weak or irregular differentiation; 2, partial differentiation; and 3, well-defined keratinocyte maturation. Inflammatory infiltrate was scored as follows: 0, marked inflammatory infiltration; 1, moderate infiltration; 2, mild infiltration; and 3, minimal or absent infiltration. Dermal remodeling was scored as follows: 0, poor granulation tissue organization and collagen remodeling; 1, limited remodeling; 2, moderate remodeling; and 3, organized dermal repair approaching normal tissue structure. The composite histological score was calculated as the sum of the five-parameter scores. Scoring was performed independently by two observers blinded to treatment allocation on coded images. For each animal, scores were averaged across the evaluated sections or images to generate a single composite value. In cases of disagreement greater than 1 point for a given parameter, slides were re-reviewed jointly to reach consensus.

### Statistics and reproducibility

All statistical analyses were performed using GraphPad Prism (v9.0). Data were presented as mean ± s.d. Comparisons across multiple groups were evaluated by one-way ANOVA with Tukey’s post hoc test. For datasets involving two independent variables, two-way ANOVA was used, followed by Šídák’s post hoc test for multiple comparisons where applicable. Statistical significance was defined as *P* < 0.05. Sample sizes and replicate definitions for each experiment are provided in the relevant Methods subsections and figure legends. In vitro experiments were performed in triplicate where indicated, including the keratinocyte migration assay and reconstructed skin experiments. For the in vivo wound-healing study, diabetic rats were randomly assigned to six experimental groups (*n* = 6 animals per group). Histological scoring was performed independently by two observers blinded to treatment allocation on coded images, and scores were averaged per animal after review of the evaluated sections or images. In cases of disagreement greater than 1 point for a given parameter, slides were re-reviewed jointly to reach consensus.

## Supplementary information

Transparent Peer Review file

Supplementary Information

Description of Additional Supplementary Files

Supplementary Data 1.xlsx

Reporting-summary

## Data Availability

Source data underlying the graphs in the main figures are provided in Supplementary Data 1. All data supporting the findings of this study are available within the Article and its Supplementary Information. Additional raw data supporting the findings of this study are available from the corresponding author upon reasonable request.
