# Supplementary material for: Biologically engineered vectors enable topical mRNA delivery for skin regeneration
Source: Commun Biol. 2026 Jul 8;9:1436. doi: 10.1038/s42003-026-10651-9 (PMC13724683; doi:10.1038/s42003-026-10651-9)
Supplement: Supplementary file 1 [file 42003_2026_10651_MOESM1_ESM.pdf]

nature portfolio

Peer Review File

Biologically engineered vectors enable topical mRNA delivery for skin regeneration

Corresponding Author: Dr Nicolas Grandchamp

This file contains all reviewer reports in order by version, followed by all author rebuttals in order by version.

Version 0:

Reviewer comments:

Reviewer #1

(Remarks to the Author)
This manuscript describes the development of virus-like particle (VLP) vectors designed for RNA delivery. These vectors are derived from lentiviruses but engineered to be reverse-transcription deficient to enhance safety. The study demonstrates that these vectors induce transient protein expression with minimal cytotoxicity. Notably, the VLPs exhibit superior thermodynamic stability at room temperature compared to parental lentiviral vectors. The authors demonstrate efficient wound healing in human keratinocytes and successful transfection in reconstructed human skin. Furthermore, the functional utility of these vectors was validated in a mouse wound repair model, where they showed enhanced histological repair compared to mRNA lipid nanoparticles (LNPs).
The technology regarding replication-deficient VLP nanoparticles is highly valuable and the characterization is thorough. Therefore, the manuscript is recommended for publication after the following revisions are addressed.

1. In Figures 2 and 5, please include both the mean fluorescence intensity (MFI) and the percentage of GFP-positive cells to provide a complete profile of transfection efficiency.
2. Why are error bars missing from Figures 1b, 2, 3, 5, and 7? Please include them to indicate statistical variance and specify the number of replicates. Also, Figure 10c needs to clarify standard deviation and the number of replicates.
3. In Figure 2, please clarify the term "timing of expression (%)".
4. Cytotoxicity should be further evaluated using cellular enzyme activity assays, such as an MTT or CCK-8 assay, to complement existing data.
5. A detailed description of the LNP components, formulation methods, and mRNA encapsulation efficiency is required for reproducibility and fair comparison.
6. In Figure 7, the strong fluorescence observed in the stratum corneum on Day 1 may be due to tissue autofluorescence, as this layer lacks cellular components. This signal leakage could potentially skew the quantification of the epidermal layer. The authors must address how they distinguished the signal from background noise or corrected for this interference.
7. The finding that VLP vectors induce better histological recovery than LNPs is compelling; however, the discussion is currently too brief. Since LNPs are known to be pro-inflammatory—which can hinder the healing process—the authors should expand on this comparison. Providing additional inflammatory marker data or a more robust literature-based discussion would strengthen the manuscript.
8. Please provide a more detailed clarification of all abbreviations used in the figure and its legend especially in Figure 8.

Reviewer #2

(Remarks to the Author)
The authors have developed rtBEVs, which are lentiviral vectors with a mutant reverse transcriptase, and verified their gene transfer and pharmacological functions using human reconstructed skin models and rat diabetic wound healing models . rtBEVs are capable of transient gene transfer and are stable at room temperature for about 19 days, demonstrating desirable features as a drug delivery system (DDS). While the rtBEVs developed by the authors show potential for medical applications, there are several methodological discrepancies and missing details in the manuscript that need to be addressed to improve its clarity and reproducibility.

Major point 1

The authors propose the conceptual framework of "Biologically Engineered Vectors (BEVs)" to represent the vectors such as extracellular vesicles and viral vectors. This seems to be presented as a counterpart to "artificial vectors" such as lipid nanoparticles, polyplexes, lipoplexes, and polymer micelles. However, terms such as Biological vectors, Biogenic vectors, Biomimetic vectors, and Biological nanoparticles have already been used in the field to describe similar concepts. When proposing BEVs as a new concept, it would be highly beneficial to explicitly explain the differences between BEVs and these existing terms.

Major point 2
The authors refer to reverse transcription-deficient BEVs as "rtBEVs," which are based on lentiviral vectors. If the concept of BEVs is to be established, it should be noted that BEVs can be viral or non-viral; therefore, the vector used in this study would be positioned as a reverse transcription-deficient BEV within the lentivirus-based viral category. The current name "rtBEVs" does not seem to fully capture this specific positioning. It would be recommended to use a nomenclature that clearly indicates its lentivirus-based origin.

Major point 3
There is a lack of comparison with existing technologies. There have been prior reports on reverse transcription-deficient vectors and integrase-deficient lentiviral vectors. Since these existing technologies are not discussed in the Introduction or Discussion, the specific positions of author's finding within this field remain somewhat unclear. Including this context would verify the importance.

Major point 4
The authors demonstrated that rtBEVs can deliver genes using a human reconstructed skin model. However, in the in vivo model, topical administration is performed after creating a wound on the skin. In this setting, the mRNA-LNPs used for comparison also appears to show a certain degree of therapeutic effect. Since topical administration of mRNA-LNPs to normal skin typically exhibits no function, the decreased barrier function of skin caused by the wound making might be contributing to the gene transfer. Consequently, it is difficult to clearly determine from this paper whether rtBEVs possess advantageous characteristics specifically for topical administration. The authors list the challenges of artificial vectors and conventional viral vectors in the Introduction, but it would be helpful to clarify exactly which of these specific problems rtBEVs are able to solve in this context.

Minor points
Figure 1 and related text: The vector concept and actual design are difficult to fully understand from reading the main text. Please consider briefly explaining them at the beginning of the Results section or explicitly referring the reader to the Materials and Methods.

Figure 2: Please check the Y-axis label. Additionally, the end of the legend reads "rtBEVE," which seems to be miss spelling.

Figure 3: In the graph legend, using "FGF7" and "GFP" instead of "A" and "B" would be more familiar and reader-friendly.

Figure 4: It would be informative to include a brief discussion on why rtBEVs show higher stability compared to lentiviral vectors. Also, please check the legend, as there appears to be miss-spelling ("trBEV")

Figure 6 and related text: The term "NHEK" appears abruptly in the text; a brief definition is needed. Furthermore, the legend mentions the use of a "Student t-test". Typically, a t-test is not used for multiple group comparisons, and this contradicts the statistical methods described in the Materials and Methods section. Please check these points.

Figure 8: The group labels corresponding to rtBEVs are written as "RNA," etc.. Furthermore, it is not specified what "H" and "L" stand for (presumably High and Low dose). Since there is no explanation for "NControl" and "DControl," it would be helpful to refer to the Materials and Methods. Lastly, the details regarding the mRNA-LNP remain unclear even when checking the Materials and Methods; providing more comprehensive information would be appreciated.

Figure 10: Although described as "Semi-quantitative," the lack of scoring criteria makes it difficult for others to reproduce the evaluation. While the figure clearly shows a difference between LNPs and rtBEVs, please define a standard explaining what specific conditions correspond to 0, 1, 2, or 3 points for each parameter.

Methods: The use of pentobarbital as a sole anesthetic agent is currently generally not recommended, I think. Could the authors please confirm that this specific animal experiment protocol was approved? (This is NOT a request to see the actual approval documents) .

Version 1:

Reviewer comments:

Reviewer #1

(Remarks to the Author)
The authors have properly addressed the concerns.

Reviewer #2

(Remarks to the Author)
The authors have sincerely and adequately addressed the concerns raised in my previous review. I appreciate their detailed responses and the corresponding revisions, particularly the improvements made to methodological reproducibility, nomenclature, and the overall readability of the manuscript. As my points have been resolved, I have no further comments.I believe the revised manuscript is now suitable for publication.

Open Access This Peer Review File is licensed under a Creative Commons Attribution 4.0 International License, which permits use, sharing, adaptation, distribution and reproduction in any medium or format, as long as you give appropriate credit to the original author(s) and the source, provide a link to the Creative Commons license, and indicate if changes were made.
In cases where reviewers are anonymous, credit should be given to 'Anonymous Referee' and the source.
The images or other third party material in this Peer Review File are included in the article's Creative Commons license, unless indicated otherwise in a credit line to the material. If material is not included in the article's Creative Commons license and your intended use is not permitted by statutory regulation or exceeds the permitted use, you will need to obtain permission directly from the copyright holder.
To view a copy of this license, visit https://creativecommons.org/licenses/by/4.0/

We sincerely thank the Editor and both Reviewers for their constructive assessment of our manuscript and for recognizing the interest and potential value of our rtBEV platform for topical mRNA delivery. We have carefully revised the manuscript to address all comments, with particular attention to data presentation, figure clarity, methodological reproducibility, nomenclature, and the positioning of rtBEVs within existing RNA delivery technologies

Reviewer #1 (Remarks to the Author):

This manuscript describes the development of virus-like particle (VLP) vectors designed for RNA delivery. These vectors are derived from lentiviruses but engineered to be reverse-transcription deficient to enhance safety. The study demonstrates that these vectors induce transient protein expression with minimal cytotoxicity. Notably, the VLPs exhibit superior thermodynamic stability at room temperature compared to parental lentiviral vectors. The authors demonstrate efficient wound healing in human keratinocytes and successful transfection in reconstructed human skin. Furthermore, the functional utility of these vectors was validated in a mouse wound repair model, where they showed enhanced histological repair compared to mRNA lipid nanoparticles (LNPs). The technology regarding replication-deficient VLP nanoparticles is highly valuable and the characterization is thorough. Therefore, the manuscript is recommended for publication after the following revisions are addressed.

1. In Figures 2 and 5, please include both the mean fluorescence intensity (MFI) and the percentage of GFP-positive cells to provide a complete profile of transfection efficiency.

This has been corrected as requested in the revised manuscript.

2. Why are error bars missing from Figures 1b, 2, 3, 5, and 7? Please include them to indicate statistical variance and specify the number of replicates. Also, Figure 10c needs to clarify standard deviation and the number of replicates.

We thank the reviewer for this important comment.

For Figure 1b, we have clarified in the revised figure legend that the particle-class distribution was obtained from a single cryoTEM analysis per vector type using semi-automatic particle detection. For the conventional lentiviral vector, 25 images were analyzed and 939 particles were classified; for rtBEV, 25 images were analyzed and 567 particles were classified. Because independent replicate cryoTEM analyses were not

performed for this specific comparison, experimental variance across replicates could not be calculated in a statistically meaningful way. We therefore did not add SD or SEM error bars to this panel, as this would imply replicate variability that was not generated for this analysis. Instead, the figure is now explicitly presented as a descriptive particle-classification analysis, with the number of analyzed images and classified particles indicated in the legend.

For figure 2, 3 and 5 the absence of error bars in the original version reflected the fact that these panels included descriptive single-determination or dose-ranging experiments, for which statistical variance cannot be calculated in a meaningful way. We agree that this needs to be stated more clearly. In the revised manuscript, we have therefore clarified the number of independent experiments and/or technical replicates in each relevant figure legend. Where replicate measurements were available, the corresponding data are now presented as mean ± s.d. with the number of replicates indicated. Where experiments were performed as single determinations, no error bars were added, as this would not be statistically appropriate; instead, the figure legends now explicitly state this and the data are presented descriptively. Importantly, the conclusions drawn from these panels are supported by independent experiments performed under related conditions, including dose-response experiments and replicated single-dose experiments, all of which showed consistent biological effects.

For Figure 7, GFP fluorescence was quantified within the viable nucleated epidermal compartment after exclusion of the stratum corneum. For each reconstructed skin sample, fluorescence was measured from three images and averaged to generate one value per sample. The analysis included three reconstructed skin samples per condition, and the graph is now presented as mean ± s.e.m. This replicate structure is explicitly stated in the Figure 7 legend, while the graph itself displays the corresponding error bars.

For Figure 10c, the panel represents a semi-quantitative histopathological score based on morphological assessment rather than a continuous biomarker measurement. Because this scoring system is ordinal, conventional error bars are not the most appropriate primary representation of variability. The figure legend and Methods section have also been revised to define the scoring criteria used for each parameter from 0 to 3. We also clarified that Figure 10c was calculated from n = 6 animals per group.

3. In Figure 2, please clarify the term "timing of expression (%)".

This has been corrected as requested in the revised manuscript.

4. Cytotoxicity should be further evaluated using cellular enzyme activity assays, such as an MTT or CCK-8 assay, to complement existing data.

We thank the reviewer for this relevant suggestion. In the revised manuscript, we have clarified the rationale for assessing cytotoxicity using LDH release. LDH release directly measures loss of plasma membrane integrity and is therefore appropriate to detect overt cytotoxicity induced by vector exposure under the conditions tested. In contrast, MTT- or CCK-8-based assays primarily reflect cellular metabolic activity and can be influenced by changes in proliferation, mitochondrial activity, cell density or trophic-factor signalling. This is particularly relevant in the present study, as some rtBEVs encode regenerative factors such as FGF7, which are expected to modulate keratinocyte behaviour and could therefore confound the interpretation of metabolic readouts as direct cytotoxicity.

We therefore considered LDH release to be the most suitable assay for the cytotoxicity question addressed here. The revised manuscript now explicitly states this rationale and clarifies the replicate structure and statistical analysis of the LDH assay. Under the tested conditions, rtBEV-GFP and rtBEV-FGF7 did not induce significant LDH release above the negative-control level, supporting the conclusion that rtBEV exposure was not associated with overt membrane-damaging cytotoxicity. For these reasons, no additional MTT or CCK-8 experiment was added, as it would not provide a more specific assessment of vector-induced cytotoxicity in this experimental context.

5. A detailed description of the LNP components, formulation methods, and mRNA encapsulation efficiency is required for reproducibility and fair comparison.

We have revised the Materials and Methods section to clarify that the mRNA-LNPs were produced by RiboPro using a proprietary formulation process and were used as a commercially sourced external comparator. The available batch characterization parameters, including particle size, PDI and RiboGreen-based confirmation of mRNA encapsulation, have been added. Detailed lipid composition and manufacturing parameters were not disclosed by the provider; therefore, no additional in-house LNP formulation or characterization experiments were performed.

6. In Figure 7, the strong fluorescence observed in the stratum corneum on Day 1 may be due to tissue autofluorescence, as this layer lacks cellular components. This signal leakage could potentially skew the quantification of the epidermal layer. The authors must address how they distinguished the signal from background noise or corrected for this interference.

We thank the reviewer for raising this important point. We agree that the stratum corneum is acellular and may display tissue autofluorescence, particularly in superficial layers. We have therefore revised the manuscript and Figure 7 legend to clarify how this signal was handled. Untreated reconstructed skin samples were used as background controls and were imaged using the same acquisition settings as treated samples. The superficial signal detected in the stratum corneum was considered as

background/autofluorescence and was not interpreted as GFP expression. Quantification was restricted to the viable nucleated epidermal compartment, excluding the stratum corneum, and fluorescence was evaluated relative to the untreated control background.

The revised text now explicitly states that the conclusion of topical rtBEV-mediated GFP expression is based on signal detected within the viable epidermal layers, rather than on the superficial stratum corneum signal.

7. The finding that VLP vectors induce better histological recovery than LNPs is compelling; however, the discussion is currently too brief. Since LNPs are known to be pro-inflammatory—which can hinder the healing process—the authors should expand on this comparison. Providing additional inflammatory marker data or a more robust literature-based discussion would strengthen the manuscript.

We thank the reviewer for this insightful comment. We agree that the comparison between rtBEV-FGF7 and mRNA-LNP-FGF7 should be discussed more thoroughly, particularly in the context of local inflammation during wound repair. In the revised manuscript, we have expanded the Discussion to address the known immunostimulatory properties of LNP formulations and their potential relevance in a diabetic wound environment, where persistent inflammation is already a key pathological feature.

Importantly, we have also tempered our interpretation to avoid overclaiming a mechanistic explanation. The revised text now states that the inferior histological outcome observed with the high-dose LNP condition may reflect formulation-dependent tissue effects, including a possible contribution of local inflammatory responses, but that the present study does not establish this mechanism directly.

8. Please provide a more detailed clarification of all abbreviations used in the figure and its legend especially in Figure 8.

We thank the reviewer for this comment. We have revised Figure 8 and its legend to provide a clearer definition of all abbreviations and group labels. In particular, the legend now defines NControl, DControl, rtBEV-L, rtBEV-H, mRNA-LNP-L and mRNA-LNP-H, and specifies that L and H refer to low- and high-dose conditions, respectively. We also harmonized the group names across the figure panels to avoid ambiguity.

<u>Reviewer #2 (Remarks to the Author):</u>

The authors have developed rtBEVs, which are lentiviral vectors with a mutant reverse transcriptase, and verified their gene transfer and pharmacological functions using human reconstructed skin models and rat diabetic wound healing models . rtBEVs are capable of transient gene transfer and are stable at room temperature for about 19 days, demonstrating desirable features as a drug delivery system (DDS). While the rtBEVs developed by the authors show potential for medical applications, there are several methodological discrepancies and missing details in the manuscript that need to be addressed to improve its clarity and reproducibility.

Major point 1

The authors propose the conceptual framework of "Biologically Engineered Vectors (BEVs)" to represent the vectors such as extracellular vesicles and viral vectors. This seems to be presented as a counterpart to "artificial vectors" such as lipid nanoparticles, polyplexes, lipoplexes, and polymer micelles. However, terms such as Biological vectors, Biogenic vectors, Biomimetic vectors, and Biological nanoparticles have already been used in the field to describe similar concepts. When proposing BEVs as a new concept, it would be highly beneficial to explicitly explain the differences between BEVs and these existing terms.

We thank the reviewer for this thoughtful comment. We agree that related terms such as biological vectors, biogenic vectors, biomimetic vectors and biological nanoparticles have previously been used in the field, and we did not intend to imply that biologically derived delivery systems are an entirely new concept. We have revised the Introduction to clarify that "Biologically Engineered Vectors" is proposed here as delineate a unified class rather than as a replacement for existing terminology. In this framework, BEVs specifically refer to biologically assembled delivery particles whose composition, cargo, tropism or functional properties are intentionally modified through genetic or cellular engineering. This distinguishes BEVs from broader terms such as biological or biogenic nanoparticles, which primarily describe origin, and from biomimetic vectors, which may include synthetic systems designed to mimic biological features. We have now clarified this distinction in the revised manuscript.

Major point 2

The authors refer to reverse transcription-deficient BEVs as "rtBEVs," which are

based on lentiviral vectors. If the concept of BEVs is to be established, it should be noted that BEVs can be viral or non-viral; therefore, the vector used in this study would be positioned as a reverse transcription-deficient BEV within the lentivirus-based viral category. The current name "rtBEVs" does not seem to fully capture this specific positioning. It would be recommended to use a nomenclature that clearly indicates its lentivirus-based origin.

We thank the reviewer for this helpful comment. We agree that the lentiviral origin of the particles used in this study should be clearly stated within the broader BEV framework. We have therefore revised the manuscript to explicitly define rtBEVs as lentiviral-particle-derived reverse-transcription-deficient BEVs and to position them as a viral subtype within the broader BEV category.

We have retained the acronym “rtBEV” throughout the manuscript because it emphasizes the defining functional feature of the platform, namely reverse-transcription deficiency, rather than implying functional equivalence with conventional lentiviral vectors optimized for durable gene transfer. This distinction is important because rtBEVs are designed to deliver mRNA transiently while preventing RNA-to-DNA conversion and genomic integration. The revised text now clarifies both the lentiviral-particle origin of the system and the rationale for the retained nomenclature.

Major point 3

There is a lack of comparison with existing technologies. There have been prior reports on reverse transcription-deficient vectors and integrase-deficient lentiviral vectors. Since these existing technologies are not discussed in the Introduction or Discussion, the specific positions of author's finding within this field remain somewhat unclear. Including this context would verify the importance.

We thank the reviewer for this important comment. We agree that the manuscript needed a clearer positioning of rtBEVs relative to existing reverse-transcription-deficient lentiviral technologies. We have therefore revised the Discussion to include a focused comparison with prior reverse-transcription-deficient retroviral and lentiviral RNA-vector systems.

Importantly, the principle underlying rtBEVs was developed by our group before the subsequent peer-reviewed reports on non-reverse-transcribable lentiviral vectors. This earlier work was disclosed in a doctoral thesis and in patent WO2013060819A2, which described retrovirus-derived vectors unable to complete reverse transcription and designed to mediate transient expression from delivered RNA. Later studies, including reverse-transcription-deficient lentiviral particles used for TALEN mRNA delivery,

provided independent peer-reviewed evidence that this strategy can support transient protein expression from packaged vector RNA.

We have now clarified this chronology and technological positioning. The present study does not aim to reintroduce reverse-transcription-deficient lentiviral particles as a generic concept, but rather to establish rtBEVs as a biologically engineered mRNA-delivery platform for topical regenerative therapy. In this context, our work extends the earlier RNA-vector principle to keratinocytes, reconstructed human epidermis, topical administration and histological repair in a diabetic wound model.

Major point 4

The authors demonstrated that rtBEVs can deliver genes using a human reconstructed skin model. However, in the in vivo model, topical administration is performed after creating a wound on the skin. In this setting, the mRNA-LNPs used for comparison also appears to show a certain degree of therapeutic effect. Since topical administration of mRNA-LNPs to normal skin typically exhibits no function, the decreased barrier function of skin caused by the wound making might be contributing to the gene transfer. Consequently, it is difficult to clearly determine from this paper whether rtBEVs possess advantageous characteristics specifically for topical administration. The authors list the challenges of artificial vectors and conventional viral vectors in the Introduction, but it would be helpful to clarify exactly which of these specific problems rtBEVs are able to solve in this context.

We agree with the reviewer that barrier disruption after wounding may facilitate topical access for both rtBEVs and mRNA-LNPs in the in vivo model. We have therefore revised the manuscript to avoid implying that the wound-healing experiment demonstrates delivery across intact normal skin. However, topical rtBEV-GFP application to reconstructed human epidermis induced GFP expression within the viable epidermal compartment in vitro, in the absence of experimentally induced wounding. Thus, the reconstructed skin model supports topical epidermal mRNA delivery by rtBEVs, whereas the in vivo wound model evaluates therapeutic performance in a clinically relevant repair setting where the barrier is compromised. We have clarified this distinction in the revised Discussion and now define the advantages addressed by rtBEVs in this context: transient RNA-only expression without RNA-to-DNA conversion, no DNA intermediate required for genomic integration, biologically mediated cell entry, topical application without injection or device-assisted deposition, room-temperature functional stability, and improved histological repair under the tested wound-healing conditions.

Minor points

Figure 1 and related text: The vector concept and actual design are difficult to fully understand from reading the main text. Please consider briefly explaining them at the beginning of the Results section or explicitly referring the reader to the Materials and Methods.

We thank the reviewer for pointing this out. We agree that the vector concept and design needed to be introduced more clearly in the main text before presenting the characterization data. We have therefore added a brief explanatory paragraph at the beginning of the Results section, before Figure 1, to summarize the rtBEV design and to explicitly refer the reader to the Materials and Methods for the detailed vector construction. This clarification is intended to improve readability and does not require additional experiments.

Figure 2: Please check the Y-axis label. Additionally, the end of the legend reads "rtBEVE," which seems to be miss spelling.

This has been corrected as requested in the revised manuscript.

Figure 3: In the graph legend, using "FGF7" and "GFP" instead of "A" and "B" would be more familiar and reader-friendly.

This has been corrected as requested in the revised manuscript.

Figure 4: It would be informative to include a brief discussion on why rtBEVs show higher stability compared to lentiviral vectors. Also, please check the legend, as there appears to be miss-spelling ("trBEV")

We thank the reviewer for this helpful comment. We have corrected the typographical error in the Figure 4 legend, replacing "trBEV" with "rtBEV". We also agree that the higher apparent room-temperature stability of rtBEVs compared with conventional lentiviral vectors required further discussion. We have therefore added a brief explanation in the Discussion.

We now clarify that Figure 4 measures functional stability, based on the ability of stored particles to induce GFP expression, rather than particle integrity alone. A possible explanation is that conventional lentiviral vector activity depends not only on particle entry but also on preservation of reverse-transcription-competent viral cores and

downstream DNA synthesis steps. In contrast, rtBEVs are designed to deliver mRNA directly and do not require reverse transcriptase activity or RNA-to-DNA conversion for transgene expression. Therefore, partial loss of reverse-transcription-dependent functions during room-temperature storage may have a stronger impact on conventional lentiviral vector infectivity than on rtBEV-mediated mRNA expression. We have added this interpretation as a plausible explanation, while avoiding overstatement of the mechanism.

Figure 6 and related text: The term "NHEK" appears abruptly in the text; a brief definition is needed. Furthermore, the legend mentions the use of a "Student t-test". Typically, a t-test is not used for multiple group comparisons, and this contradicts the statistical methods described in the Materials and Methods section. Please check these points.

We thank the reviewer for pointing out these issues. We have revised the Results section and Figure 6 legend to define NHEK at first mention as normal human epidermal keratinocytes. We also corrected the statistical information in the Figure 6 legend. The previous mention of a Student's t-test was inappropriate for this multiple-group comparison and has been removed. The legend now states that data were analyzed using ANOVA followed by the appropriate post hoc multiple-comparison test, consistent with the Statistical analysis section of the Materials and Methods.

Figure 8: The group labels corresponding to rtBEVs are written as "RNA," etc.. Furthermore, it is not specified what "H" and "L" stand for (presumably High and Low dose). Since there is no explanation for "NControl" and "DControl," it would be helpful to refer to the Materials and Methods. Lastly, the details regarding the mRNA-LNP remain unclear even when checking the Materials and Methods; providing more comprehensive information would be appreciated.

This has been corrected as requested in the revised manuscript.

Figure 10: Although described as "Semi-quantitative," the lack of scoring criteria makes it difficult for others to reproduce the evaluation. While the figure clearly shows a difference between LNPs and rtBEVs, please define a standard explaining what specific conditions correspond to 0, 1, 2, or 3 points for each parameter.

We thank the reviewer for this important comment. We agree that the semi-quantitative histological scoring criteria needed to be explicitly defined to improve reproducibility. We

have therefore revised the Methods section and the Figure 10 legend to include a detailed scoring grid specifying the morphological criteria corresponding to scores 0, 1, 2 and 3 for each parameter.

Briefly, each parameter was scored on a 0–3 ordinal scale, where 0 corresponded to absent or poor repair features and 3 corresponded to near-normal tissue organization or optimal repair. For inflammatory infiltrate, the scale was oriented so that 0 represented marked inflammation and 3 represented minimal or absent inflammation. The composite score was calculated by summing the individual parameter scores. This revision clarifies the histological evaluation performed on the existing tissue sections

Methods: The use of pentobarbital as a sole anesthetic agent is currently generally not recommended, I think. Could the authors please confirm that this specific animal experiment protocol was approved? (This is NOT a request to see the actual approval documents).

We thank the reviewer for raising this point. We confirm that the animal experiment protocol, including the use of intraperitoneal pentobarbital sodium anesthesia for wound creation, was reviewed and approved by the Institutional Animal Care and Use Committee of Nantong University under Approval No. S20250522-004. We have clarified this point in the revised Methods section to explicitly state that the anesthetic regimen was part of the approved protocol.

We sincerely thank the Editor and both Reviewers for their constructive assessment of our manuscript and for recognizing the interest and potential value of our rtBEV platform for topical mRNA delivery. We have carefully revised the manuscript to address all comments, with particular attention to data presentation, figure clarity, methodological reproducibility, nomenclature, and the positioning of rtBEVs within existing RNA delivery technologies

Reviewer #1 (Remarks to the Author):

This manuscript describes the development of virus-like particle (VLP) vectors designed for RNA delivery. These vectors are derived from lentiviruses but engineered to be reverse-transcription deficient to enhance safety. The study demonstrates that these vectors induce transient protein expression with minimal cytotoxicity. Notably, the VLPs exhibit superior thermodynamic stability at room temperature compared to parental lentiviral vectors. The authors demonstrate efficient wound healing in human keratinocytes and successful transfection in reconstructed human skin. Furthermore, the functional utility of these vectors was validated in a mouse wound repair model, where they showed enhanced histological repair compared to mRNA lipid nanoparticles (LNPs). The technology regarding replication-deficient VLP nanoparticles is highly valuable and the characterization is thorough. Therefore, the manuscript is recommended for publication after the following revisions are addressed.

1. In Figures 2 and 5, please include both the mean fluorescence intensity (MFI) and the percentage of GFP-positive cells to provide a complete profile of transfection efficiency.

This has been corrected as requested in the revised manuscript.

2. Why are error bars missing from Figures 1b, 2, 3, 5, and 7? Please include them to indicate statistical variance and specify the number of replicates. Also, Figure 10c needs to clarify standard deviation and the number of replicates.

We thank the reviewer for this important comment.

For Figure 1b, we have clarified in the revised figure legend that the particle-class distribution was obtained from a single cryoTEM analysis per vector type using semi-automatic particle detection. For the conventional lentiviral vector, 25 images were analyzed and 939 particles were classified; for rtBEV, 25 images were analyzed and 567 particles were classified. Because independent replicate cryoTEM analyses were not

performed for this specific comparison, experimental variance across replicates could not be calculated in a statistically meaningful way. We therefore did not add SD or SEM error bars to this panel, as this would imply replicate variability that was not generated for this analysis. Instead, the figure is now explicitly presented as a descriptive particle-classification analysis, with the number of analyzed images and classified particles indicated in the legend.

For figure 2, 3 and 5 the absence of error bars in the original version reflected the fact that these panels included descriptive single-determination or dose-ranging experiments, for which statistical variance cannot be calculated in a meaningful way. We agree that this needs to be stated more clearly. In the revised manuscript, we have therefore clarified the number of independent experiments and/or technical replicates in each relevant figure legend. Where replicate measurements were available, the corresponding data are now presented as mean ± s.d. with the number of replicates indicated. Where experiments were performed as single determinations, no error bars were added, as this would not be statistically appropriate; instead, the figure legends now explicitly state this and the data are presented descriptively. Importantly, the conclusions drawn from these panels are supported by independent experiments performed under related conditions, including dose-response experiments and replicated single-dose experiments, all of which showed consistent biological effects.

For Figure 7, GFP fluorescence was quantified within the viable nucleated epidermal compartment after exclusion of the stratum corneum. For each reconstructed skin sample, fluorescence was measured from three images and averaged to generate one value per sample. The analysis included three reconstructed skin samples per condition, and the graph is now presented as mean ± s.e.m. This replicate structure is explicitly stated in the Figure 7 legend, while the graph itself displays the corresponding error bars.

For Figure 10c, the panel represents a semi-quantitative histopathological score based on morphological assessment rather than a continuous biomarker measurement. Because this scoring system is ordinal, conventional error bars are not the most appropriate primary representation of variability. The figure legend and Methods section have also been revised to define the scoring criteria used for each parameter from 0 to 3. We also clarified that Figure 10c was calculated from n = 6 animals per group.

3. In Figure 2, please clarify the term "timing of expression (%)".

This has been corrected as requested in the revised manuscript.

4. Cytotoxicity should be further evaluated using cellular enzyme activity assays, such as an MTT or CCK-8 assay, to complement existing data.

We thank the reviewer for this relevant suggestion. In the revised manuscript, we have clarified the rationale for assessing cytotoxicity using LDH release. LDH release directly measures loss of plasma membrane integrity and is therefore appropriate to detect overt cytotoxicity induced by vector exposure under the conditions tested. In contrast, MTT- or CCK-8-based assays primarily reflect cellular metabolic activity and can be influenced by changes in proliferation, mitochondrial activity, cell density or trophic-factor signalling. This is particularly relevant in the present study, as some rtBEVs encode regenerative factors such as FGF7, which are expected to modulate keratinocyte behaviour and could therefore confound the interpretation of metabolic readouts as direct cytotoxicity.

We therefore considered LDH release to be the most suitable assay for the cytotoxicity question addressed here. The revised manuscript now explicitly states this rationale and clarifies the replicate structure and statistical analysis of the LDH assay. Under the tested conditions, rtBEV-GFP and rtBEV-FGF7 did not induce significant LDH release above the negative-control level, supporting the conclusion that rtBEV exposure was not associated with overt membrane-damaging cytotoxicity. For these reasons, no additional MTT or CCK-8 experiment was added, as it would not provide a more specific assessment of vector-induced cytotoxicity in this experimental context.

5. A detailed description of the LNP components, formulation methods, and mRNA encapsulation efficiency is required for reproducibility and fair comparison.

We have revised the Materials and Methods section to clarify that the mRNA-LNPs were produced by RiboPro using a proprietary formulation process and were used as a commercially sourced external comparator. The available batch characterization parameters, including particle size, PDI and RiboGreen-based confirmation of mRNA encapsulation, have been added. Detailed lipid composition and manufacturing parameters were not disclosed by the provider; therefore, no additional in-house LNP formulation or characterization experiments were performed.

6. In Figure 7, the strong fluorescence observed in the stratum corneum on Day 1 may be due to tissue autofluorescence, as this layer lacks cellular components. This signal leakage could potentially skew the quantification of the epidermal layer. The authors must address how they distinguished the signal from background noise or corrected for this interference.

We thank the reviewer for raising this important point. We agree that the stratum corneum is acellular and may display tissue autofluorescence, particularly in superficial layers. We have therefore revised the manuscript and Figure 7 legend to clarify how this signal was handled. Untreated reconstructed skin samples were used as background controls and were imaged using the same acquisition settings as treated samples. The superficial signal detected in the stratum corneum was considered as

background/autofluorescence and was not interpreted as GFP expression. Quantification was restricted to the viable nucleated epidermal compartment, excluding the stratum corneum, and fluorescence was evaluated relative to the untreated control background.

The revised text now explicitly states that the conclusion of topical rtBEV-mediated GFP expression is based on signal detected within the viable epidermal layers, rather than on the superficial stratum corneum signal.

7. The finding that VLP vectors induce better histological recovery than LNPs is compelling; however, the discussion is currently too brief. Since LNPs are known to be pro-inflammatory—which can hinder the healing process—the authors should expand on this comparison. Providing additional inflammatory marker data or a more robust literature-based discussion would strengthen the manuscript.

We thank the reviewer for this insightful comment. We agree that the comparison between rtBEV-FGF7 and mRNA-LNP-FGF7 should be discussed more thoroughly, particularly in the context of local inflammation during wound repair. In the revised manuscript, we have expanded the Discussion to address the known immunostimulatory properties of LNP formulations and their potential relevance in a diabetic wound environment, where persistent inflammation is already a key pathological feature.

Importantly, we have also tempered our interpretation to avoid overclaiming a mechanistic explanation. The revised text now states that the inferior histological outcome observed with the high-dose LNP condition may reflect formulation-dependent tissue effects, including a possible contribution of local inflammatory responses, but that the present study does not establish this mechanism directly.

8. Please provide a more detailed clarification of all abbreviations used in the figure and its legend especially in Figure 8.

We thank the reviewer for this comment. We have revised Figure 8 and its legend to provide a clearer definition of all abbreviations and group labels. In particular, the legend now defines NControl, DControl, rtBEV-L, rtBEV-H, mRNA-LNP-L and mRNA-LNP-H, and specifies that L and H refer to low- and high-dose conditions, respectively. We also harmonized the group names across the figure panels to avoid ambiguity.

Reviewer #2 (Remarks to the Author):

The authors have developed rtBEVs, which are lentiviral vectors with a mutant reverse transcriptase, and verified their gene transfer and pharmacological functions using human reconstructed skin models and rat diabetic wound healing models . rtBEVs are capable of transient gene transfer and are stable at room temperature for about 19 days, demonstrating desirable features as a drug delivery system (DDS). While the rtBEVs developed by the authors show potential for medical applications, there are several methodological discrepancies and missing details in the manuscript that need to be addressed to improve its clarity and reproducibility.

Major point 1

The authors propose the conceptual framework of "Biologically Engineered Vectors (BEVs)" to represent the vectors such as extracellular vesicles and viral vectors. This seems to be presented as a counterpart to "artificial vectors" such as lipid nanoparticles, polyplexes, lipoplexes, and polymer micelles. However, terms such as Biological vectors, Biogenic vectors, Biomimetic vectors, and Biological nanoparticles have already been used in the field to describe similar concepts. When proposing BEVs as a new concept, it would be highly beneficial to explicitly explain the differences between BEVs and these existing terms.

We thank the reviewer for this thoughtful comment. We agree that related terms such as biological vectors, biogenic vectors, biomimetic vectors and biological nanoparticles have previously been used in the field, and we did not intend to imply that biologically derived delivery systems are an entirely new concept. We have revised the Introduction to clarify that “Biologically Engineered Vectors” is proposed here as delineate a unified class rather than as a replacement for existing terminology. In this framework, BEVs specifically refer to biologically assembled delivery particles whose composition, cargo, tropism or functional properties are intentionally modified through genetic or cellular engineering. This distinguishes BEVs from broader terms such as biological or biogenic nanoparticles, which primarily describe origin, and from biomimetic vectors, which may include synthetic systems designed to mimic biological features. We have now clarified this distinction in the revised manuscript.

Major point 2

The authors refer to reverse transcription-deficient BEVs as "rtBEVs," which are

based on lentiviral vectors. If the concept of BEVs is to be established, it should be noted that BEVs can be viral or non-viral; therefore, the vector used in this study would be positioned as a reverse transcription-deficient BEV within the lentivirus-based viral category. The current name "rtBEVs" does not seem to fully capture this specific positioning. It would be recommended to use a nomenclature that clearly indicates its lentivirus-based origin.

We thank the reviewer for this helpful comment. We agree that the lentiviral origin of the particles used in this study should be clearly stated within the broader BEV framework. We have therefore revised the manuscript to explicitly define rtBEVs as lentiviral-particle-derived reverse-transcription-deficient BEVs and to position them as a viral subtype within the broader BEV category.

We have retained the acronym "rtBEV" throughout the manuscript because it emphasizes the defining functional feature of the platform, namely reverse-transcription deficiency, rather than implying functional equivalence with conventional lentiviral vectors optimized for durable gene transfer. This distinction is important because rtBEVs are designed to deliver mRNA transiently while preventing RNA-to-DNA conversion and genomic integration. The revised text now clarifies both the lentiviral-particle origin of the system and the rationale for the retained nomenclature.

Major point 3

There is a lack of comparison with existing technologies. There have been prior reports on reverse transcription-deficient vectors and integrase-deficient lentiviral vectors. Since these existing technologies are not discussed in the Introduction or Discussion, the specific positions of author's finding within this field remain somewhat unclear. Including this context would verify the importance.

We thank the reviewer for this important comment. We agree that the manuscript needed a clearer positioning of rtBEVs relative to existing reverse-transcription-deficient lentiviral technologies. We have therefore revised the Discussion to include a focused comparison with prior reverse-transcription-deficient retroviral and lentiviral RNA-vector systems.

Importantly, the principle underlying rtBEVs was developed by our group before the subsequent peer-reviewed reports on non-reverse-transcribable lentiviral vectors. This earlier work was disclosed in a doctoral thesis and in patent WO2013060819A2, which described retrovirus-derived vectors unable to complete reverse transcription and designed to mediate transient expression from delivered RNA. Later studies, including reverse-transcription-deficient lentiviral particles used for TALEN mRNA delivery,

provided independent peer-reviewed evidence that this strategy can support transient protein expression from packaged vector RNA.

We have now clarified this chronology and technological positioning. The present study does not aim to reintroduce reverse-transcription-deficient lentiviral particles as a generic concept, but rather to establish rtBEVs as a biologically engineered mRNA-delivery platform for topical regenerative therapy. In this context, our work extends the earlier RNA-vector principle to keratinocytes, reconstructed human epidermis, topical administration and histological repair in a diabetic wound model.

Major point 4

The authors demonstrated that rtBEVs can deliver genes using a human reconstructed skin model. However, in the in vivo model, topical administration is performed after creating a wound on the skin. In this setting, the mRNA-LNPs used for comparison also appears to show a certain degree of therapeutic effect. Since topical administration of mRNA-LNPs to normal skin typically exhibits no function, the decreased barrier function of skin caused by the wound making might be contributing to the gene transfer. Consequently, it is difficult to clearly determine from this paper whether rtBEVs possess advantageous characteristics specifically for topical administration. The authors list the challenges of artificial vectors and conventional viral vectors in the Introduction, but it would be helpful to clarify exactly which of these specific problems rtBEVs are able to solve in this context.

We agree with the reviewer that barrier disruption after wounding may facilitate topical access for both rtBEVs and mRNA-LNPs in the in vivo model. We have therefore revised the manuscript to avoid implying that the wound-healing experiment demonstrates delivery across intact normal skin. However, topical rtBEV-GFP application to reconstructed human epidermis induced GFP expression within the viable epidermal compartment in vitro, in the absence of experimentally induced wounding. Thus, the reconstructed skin model supports topical epidermal mRNA delivery by rtBEVs, whereas the in vivo wound model evaluates therapeutic performance in a clinically relevant repair setting where the barrier is compromised. We have clarified this distinction in the revised Discussion and now define the advantages addressed by rtBEVs in this context: transient RNA-only expression without RNA-to-DNA conversion, no DNA intermediate required for genomic integration, biologically mediated cell entry, topical application without injection or device-assisted deposition, room-temperature functional stability, and improved histological repair under the tested wound-healing conditions.

Minor points

Figure 1 and related text: The vector concept and actual design are difficult to fully understand from reading the main text. Please consider briefly explaining them at the beginning of the Results section or explicitly referring the reader to the Materials and Methods.

We thank the reviewer for pointing this out. We agree that the vector concept and design needed to be introduced more clearly in the main text before presenting the characterization data. We have therefore added a brief explanatory paragraph at the beginning of the Results section, before Figure 1, to summarize the rtBEV design and to explicitly refer the reader to the Materials and Methods for the detailed vector construction. This clarification is intended to improve readability and does not require additional experiments.

Figure 2: Please check the Y-axis label. Additionally, the end of the legend reads "rtBEVE," which seems to be miss spelling.

This has been corrected as requested in the revised manuscript.

Figure 3: In the graph legend, using "FGF7" and "GFP" instead of "A" and "B" would be more familiar and reader-friendly.

This has been corrected as requested in the revised manuscript.

Figure 4: It would be informative to include a brief discussion on why rtBEVs show higher stability compared to lentiviral vectors. Also, please check the legend, as there appears to be miss-spelling ("trBEV")

We thank the reviewer for this helpful comment. We have corrected the typographical error in the Figure 4 legend, replacing “trBEV” with “rtBEV”. We also agree that the higher apparent room-temperature stability of rtBEVs compared with conventional lentiviral vectors required further discussion. We have therefore added a brief explanation in the Discussion.

We now clarify that Figure 4 measures functional stability, based on the ability of stored particles to induce GFP expression, rather than particle integrity alone. A possible explanation is that conventional lentiviral vector activity depends not only on particle entry but also on preservation of reverse-transcription-competent viral cores and

downstream DNA synthesis steps. In contrast, rtBEVs are designed to deliver mRNA directly and do not require reverse transcriptase activity or RNA-to-DNA conversion for transgene expression. Therefore, partial loss of reverse-transcription-dependent functions during room-temperature storage may have a stronger impact on conventional lentiviral vector infectivity than on rtBEV-mediated mRNA expression. We have added this interpretation as a plausible explanation, while avoiding overstatement of the mechanism.

Figure 6 and related text: The term "NHEK" appears abruptly in the text; a brief definition is needed. Furthermore, the legend mentions the use of a "Student t-test". Typically, a t-test is not used for multiple group comparisons, and this contradicts the statistical methods described in the Materials and Methods section. Please check these points.

We thank the reviewer for pointing out these issues. We have revised the Results section and Figure 6 legend to define NHEK at first mention as normal human epidermal keratinocytes. We also corrected the statistical information in the Figure 6 legend. The previous mention of a Student's t-test was inappropriate for this multiple-group comparison and has been removed. The legend now states that data were analyzed using ANOVA followed by the appropriate post hoc multiple-comparison test, consistent with the Statistical analysis section of the Materials and Methods.

Figure 8: The group labels corresponding to rtBEVs are written as "RNA," etc.. Furthermore, it is not specified what "H" and "L" stand for (presumably High and Low dose). Since there is no explanation for "NControl" and "DControl," it would be helpful to refer to the Materials and Methods. Lastly, the details regarding the mRNA-LNP remain unclear even when checking the Materials and Methods; providing more comprehensive information would be appreciated.

This has been corrected as requested in the revised manuscript.

Figure 10: Although described as "Semi-quantitative," the lack of scoring criteria makes it difficult for others to reproduce the evaluation. While the figure clearly shows a difference between LNPs and rtBEVs, please define a standard explaining what specific conditions correspond to 0, 1, 2, or 3 points for each parameter.

We thank the reviewer for this important comment. We agree that the semi-quantitative histological scoring criteria needed to be explicitly defined to improve reproducibility. We

have therefore revised the Methods section and the Figure 10 legend to include a detailed scoring grid specifying the morphological criteria corresponding to scores 0, 1, 2 and 3 for each parameter.

Briefly, each parameter was scored on a 0–3 ordinal scale, where 0 corresponded to absent or poor repair features and 3 corresponded to near-normal tissue organization or optimal repair. For inflammatory infiltrate, the scale was oriented so that 0 represented marked inflammation and 3 represented minimal or absent inflammation. The composite score was calculated by summing the individual parameter scores. This revision clarifies the histological evaluation performed on the existing tissue sections

Methods: The use of pentobarbital as a sole anesthetic agent is currently generally not recommended, I think. Could the authors please confirm that this specific animal experiment protocol was approved? (This is NOT a request to see the actual approval documents).

We thank the reviewer for raising this point. We confirm that the animal experiment protocol, including the use of intraperitoneal pentobarbital sodium anesthesia for wound creation, was reviewed and approved by the Institutional Animal Care and Use Committee of Nantong University under Approval No. S20250522-004. We have clarified this point in the revised Methods section to explicitly state that the anesthetic regimen was part of the approved protocol.
