# Supplementary material for: Biologically engineered vectors enable topical mRNA delivery for skin regeneration
Source: Commun Biol. 2026 Jul 8;9:1436. doi: 10.1038/s42003-026-10651-9 (PMC13724683; doi:10.1038/s42003-026-10651-9)
Supplement: Supplementary file 2 [file 42003_2026_10651_MOESM2_ESM.pdf]

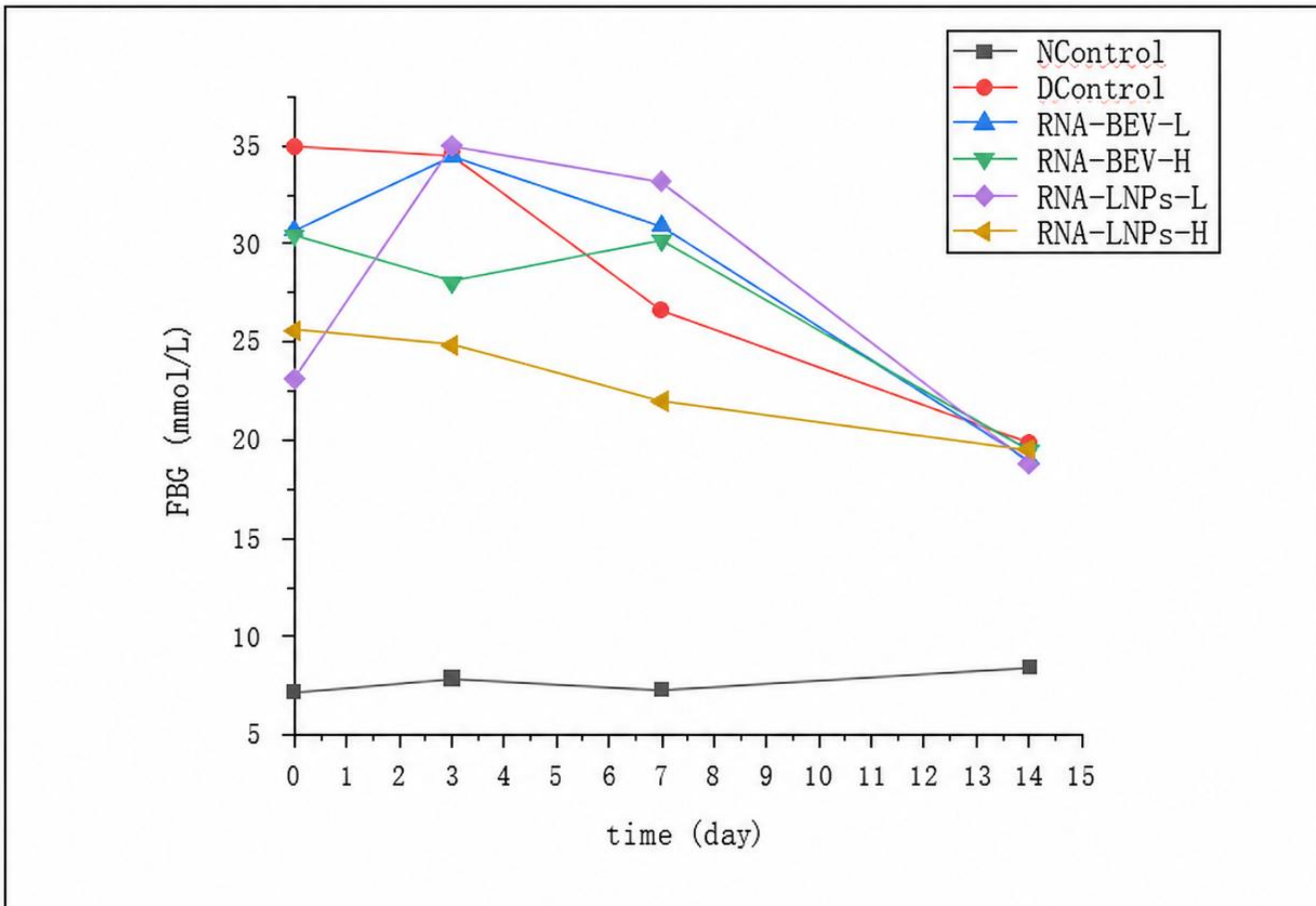

FBG (mmol/L)

Group	0	3	7	14
NControl	7.2±2.69	7.9±1.22	7.33±0.66	8.47±1.75
DControl	35±0	34.52±1.18	26.6±3	19.83±3.34
RNA-BEV-L	30.7±7.33	34.43±1.39	30.88±5.64	19.03±1.47
RNA-BEV-H	30.38±4.62	28.08±10.52	30.15±6.56	19.5±1.8
RNA-LNPs-L	23.17±13.35	35±0	33.18±2.96	18.82±1.4
RNA-LNPs-H	25.6±10.48	24.85±12.32	22±8.27	19.53±1.33

Supplementary Figure 1a | Changes in fasting blood glucose levels following treatment.
Fasting blood glucose (FBG) levels were measured at days 0, 3, 7, and 14 in normal control rat (NControl), diabetic control rat (DControl), and diabetic rat treated with low- or high-dose RNA-BEV (RNA-BEV-L and RNA-BEV-H) or mRNA lipid nanoparticles (RNA-LNPs-L and RNA-LNPs-H). Data are presented as mean ± s.d. (n = 6 per group). While NControl animals maintained stable normoglycaemia throughout the study (~7–8 mmol L^{-1}), diabetic groups exhibited markedly elevated glucose levels at baseline. Treatment with RNA-BEV or RNA-LNPs resulted in a progressive reduction in FBG over the 14-day period, with values converging to ~19 mmol L^{-1} by day 14. Source data for the graph are provided in the accompanying table.

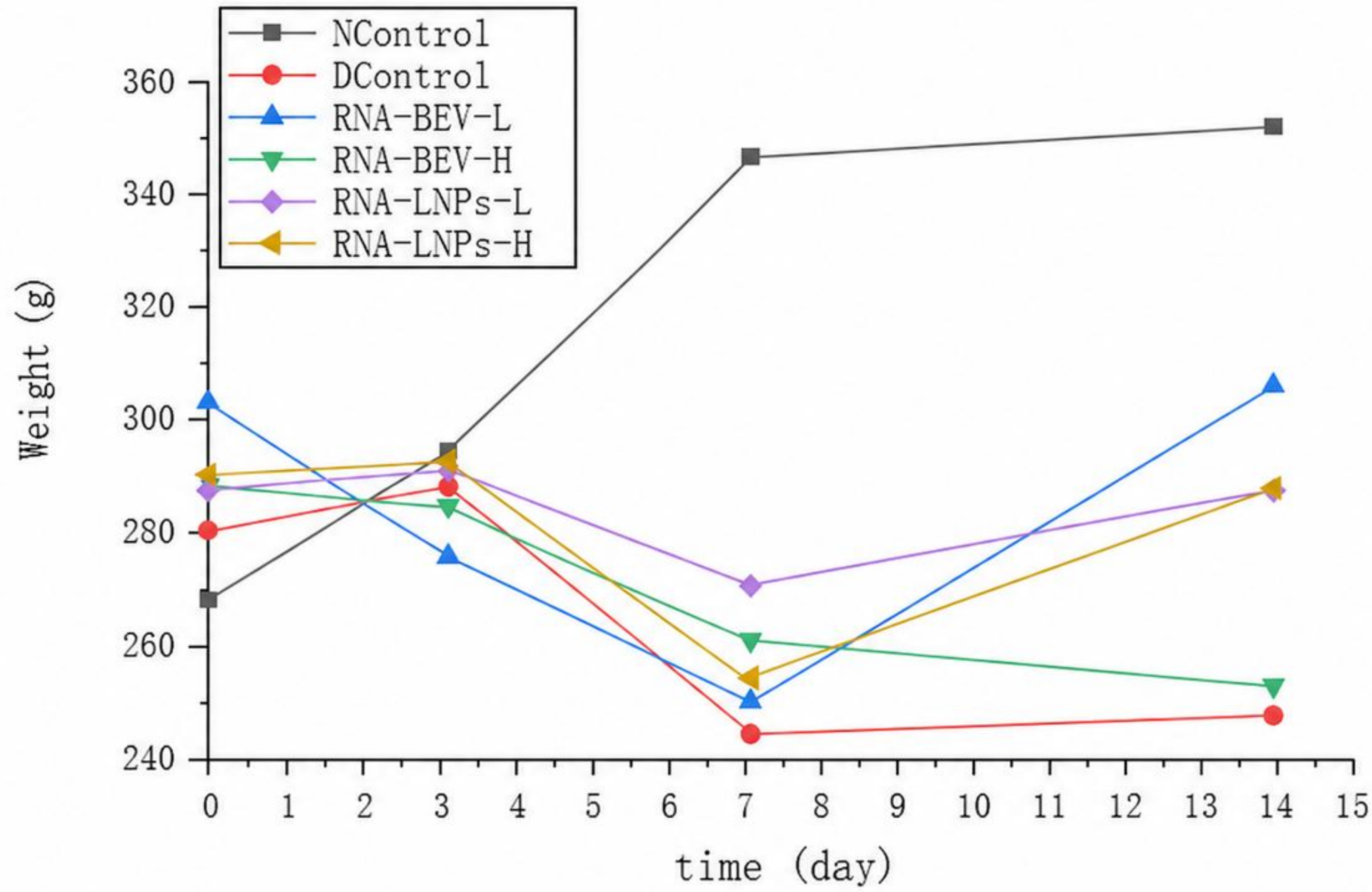

Weight (g)

Group	0	3	7	14
NControl	269±3.79	294.17±32.85	347.17±16.38	352.5±16.2
DControl	281±13.56	288.83±11.36	248.33±27.76	251.17±30.22
RNA-BEV-L	303.17±27.53	276.5±16.86	252.67±19.01	305.83±93.46
RNA-BEV-H	288.83±11.36	285.33±12.39	262.83±18.83	255.33±33.12
RNA-LNPs-L	288±36.85	291.17±16.25	271.67±16.15	288±108.87
RNA-LNPs-H	290.5±38.49	292.33±36.99	258±40.19	288.33±87.42

Supplementary Figure 1b | Changes in body weight during treatment
Body weight was monitored at days 0, 3, 7, and 14 in normal control rat (NControl), diabetic control rat (DControl), and diabetic rat treated with low- or high-dose RNA-BEV (RNA-BEV-L and RNA-BEV-H) or mRNA lipid nanoparticles (RNA-LNPs-L and RNA-LNPs-H). Data are presented as mean ± s.d. (n = 6 per group). NControl animals showed a progressive increase in body weight over the 14-day period. In contrast, diabetic control rat exhibited a reduction in body weight after day 3 that persisted until day 14. rat treated with RNA-BEV or RNA-LNPs displayed transient weight loss around day 7 followed by partial recovery by day 14. Source data corresponding to the graph are provided in the accompanying table.

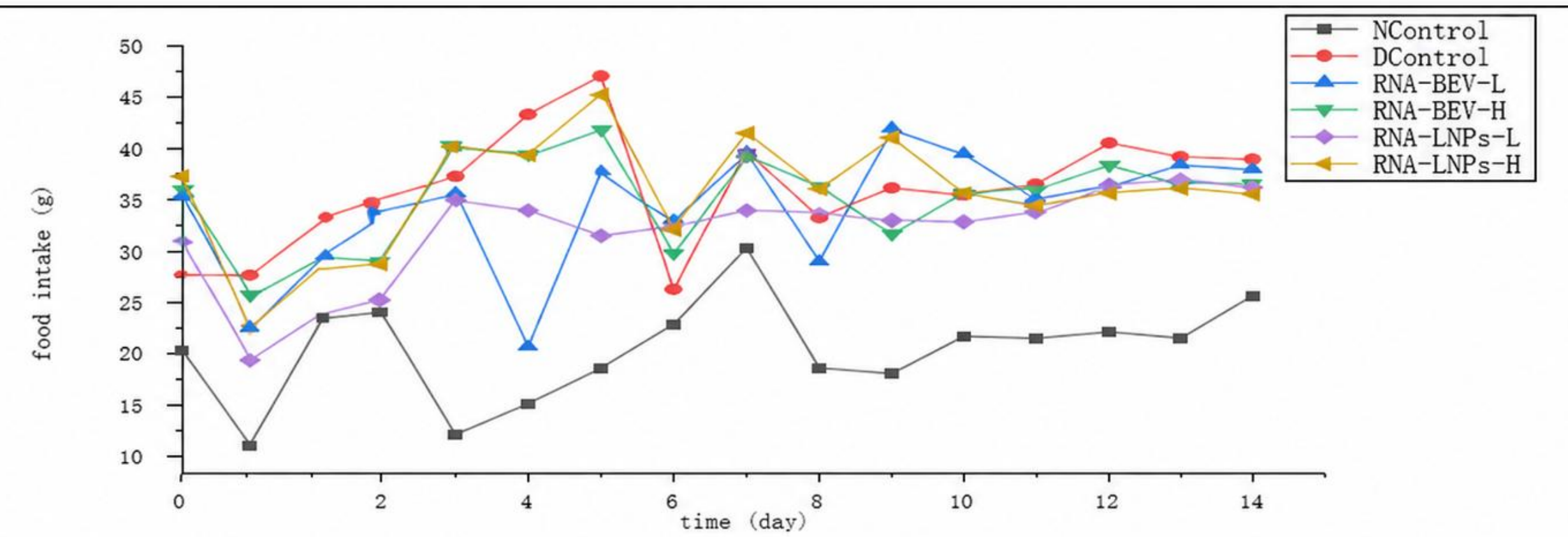

Food intake (g)

Group	0	1	2	3	4	6
NControl	20.35±7.84	20.35±7.84	23.8±3.41	12.1±2.47	14.98±4.43	18.48±1.81
DControl	27.65±8.54	27.65±8.54	34.6±4.65	37.3±3.47	43.55±3.4	47.42±4
RNA-BEV-L	35.65±5.32	35.65±5.32	33.95±6.39	35.62±4.78	20.53±10.57	37.57±3.79
RNA-BEV-H	36.13±14.64	36.13±14.64	28.93±7.04	40.15±6.29	39.42±5.46	29.02±7.54
RNA-LNPs-L	30.85±7.87	30.85±7.87	25.05±10.68	34.82±11.37	33.98±8.53	31.51±3.01
RNA-LNPs-H	37.37±5.37	37.37±5.37	28.55±4.4	40.23±4.66	39.55±11.45	31.88±5.3

Group	7	8	9	10	11	12	13	14
NControl	30.32±3.01	18.47±2.96	17.95±4.69	21.6±3.77	21.45±1.75	21.97±5.04	21.37±5.19	25.62±7.67
DControl	39.68±10.17	33.28±6.11	36.18±3.43	35.4±3.97	36.58±3.05	40.72±2.48	39.28±2.48	39±42.8
RNA-BEV-L	39.62±10.4	28.92±4.37	42.05±5.93	39.77±2.6	35.23±3.22	36.43±3.46	38.57±3.46	38.1±3.12
RNA-BEV-H	39.28±5.79	36.2±3.77	31.8±6.88	35.67±4.19	36±2.98	38.5±3.92	36.5±2.03	36.83±3.85
RNA-LNPs-L	34.03±7.38	33.7±8.97	32.95±4.06	32.93±4.85	33.73±1.88	36.23±1.87	37.1±3.95	36.23±1.87
RNA-LNPs-H	41.62±6.51	35.75±5.7	41.1±6.88	35.67±0.86	34.33±0.86	35.67±0.86	36±3.91	35.67±0.86

Supplementary Figure 1c | Daily food intake during the treatment period.
Daily food intake was recorded for 14 days in normal control rat (NControl), diabetic control rat (DControl), and diabetic rat treated with low- or high-dose RNA-BEV (RNA-BEV-L and RNA-BEV-H) or mRNA lipid nanoparticles (RNA-LNPs-L and RNA-LNPs-H). Food consumption was measured daily and expressed in grams (g). Data are presented as mean ± s.d. (n = 6 per group). NControl animals maintained relatively stable and lower food intake throughout the study. In contrast, diabetic control rat showed increased food consumption compared with NControl animals, consistent with diabetic hyperphagia. rat treated with RNA-BEV or RNA-LNPs displayed food intake patterns comparable to diabetic controls with moderate fluctuations during the treatment period. Source data corresponding to the graph are provided in the accompanying table.

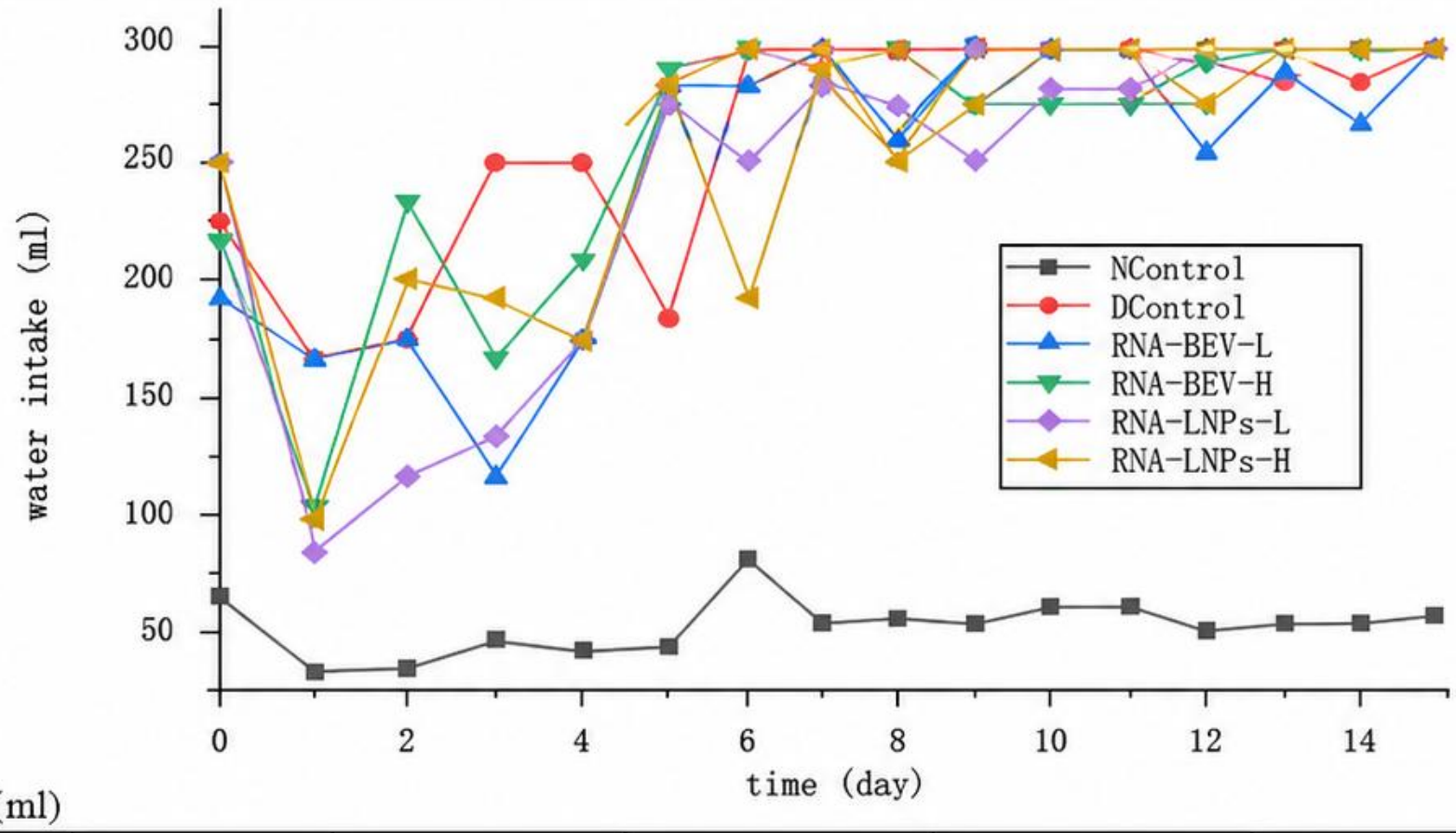

water intake (ml)

Group	0	1	2	3	4	5	6
NControl	65.83±18.55	33.33±5.16	35±4.47	46.67±5.16	41.67±19.15	44.17±4.92	81.67±29.94
DControl	225±27.39	166.67±40.82	175±41.83	250±0	250±0	183.33±25.82	300±0
RNA-BEV-L	191.67±37.64	166.67±60.55	175±27.39	116.67±68.31	175±61.24	283.33±25.82	283.33±40.82
RNA-BEV-H	216.67±25.82	105±59.58	233.33±25.82	166.67±40.82	208.33±20.41	291.67±20.41	300±0
RNA-LNPs-L	250±0	85±40.37	116.67±51.64	133.33±51.64	175±61.24	275±27.39	250±0
RNA-LNPs-H	250±0	98.33±57.42	200±77.46	191.67±58.45	175±61.24	283.33±25.82	191.67±73.6

Group	7	8	9	10	11	12	13	14
NControl	54.17±24.17	56.67±15.38	54.17±13.57	61.67±13.29	50.83±9.17	54.17±13.57	55±13.78	56.67±12.11
DControl	300±0	300±0	300±0	300±0	291.67±2.41	283.33±25.82	300±0	300±300
RNA-BEV-L	300±0	258.33±6.46	300±0	300±0	255±64.42	291.67±2.41	266.67±5	275±41.83
RNA-BEV-H	291.67±2.41	300±0	275±61.24	275±61.24	275±61.24	300±0	300±0	283.33±40.82
RNA-LNPs-L	283.33±25.82	275±61.24	250±77.46	283.33±40.82	300±0	300±0	300±0	300±0
RNA-LNPs-H	291.67±2.41	250±77.46	275±61.24	300±0	300±0	300±0	300±0	300±0

Supplementary Figure 1d | Daily water intake during the treatment period.
Daily water intake was recorded for 14 days in normal control rat (NControl), diabetic control rat (DControl), and diabetic rat treated with low- or high-dose RNA-BEV (RNA-BEV-L and RNA-BEV-H) or mRNA lipid nanoparticles (RNA-LNPs-L and RNA-LNPs-H). Water consumption was measured daily and expressed in milliliters (ml). Data are presented as mean ± s.d. (n = 6 per group). NControl animals maintained relatively stable and lower water intake throughout the study. In contrast, diabetic control rat exhibited markedly elevated water consumption compared with NControl animals, consistent with diabetic polydipsia. rat treated with RNA-BEV or RNA-LNPs showed water intake levels comparable to diabetic controls with moderate fluctuations during the treatment period. Source data corresponding to the graph are provided in the accompanying table.

Animal 1

Supplementary Figure 2a | Representative H&E staining of skin tissue of control animals / Day 0.
Representative hematoxylin and eosin (H&E)–stained section of skin tissue collected from experimental rat. The image shows the typical architecture of the skin, including the epidermal layer with stratified keratinized epithelium and the underlying dermis composed of dense collagen fibers and connective tissue structures.
(n = 6 per group).

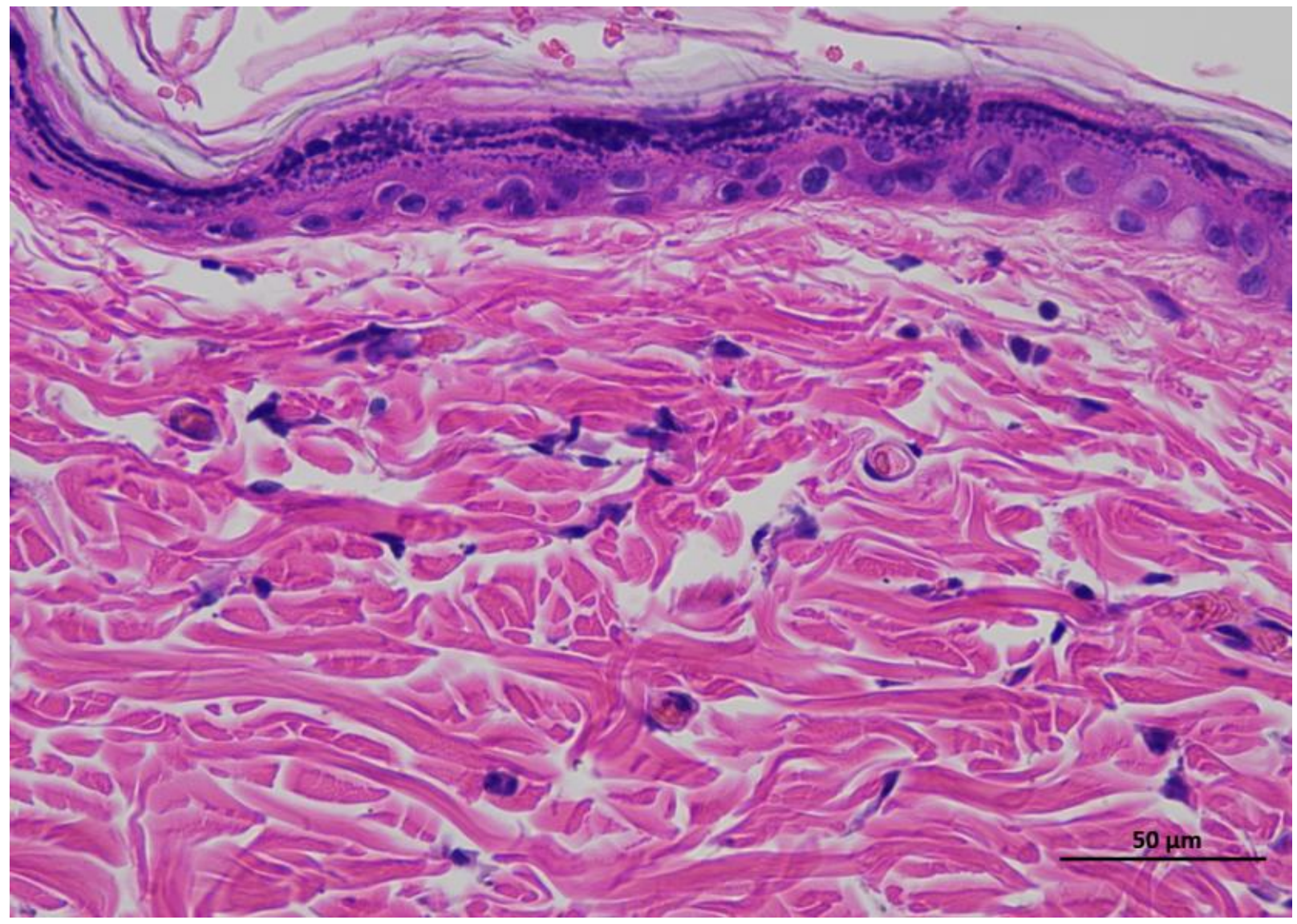

1 NControl 0day 400-1

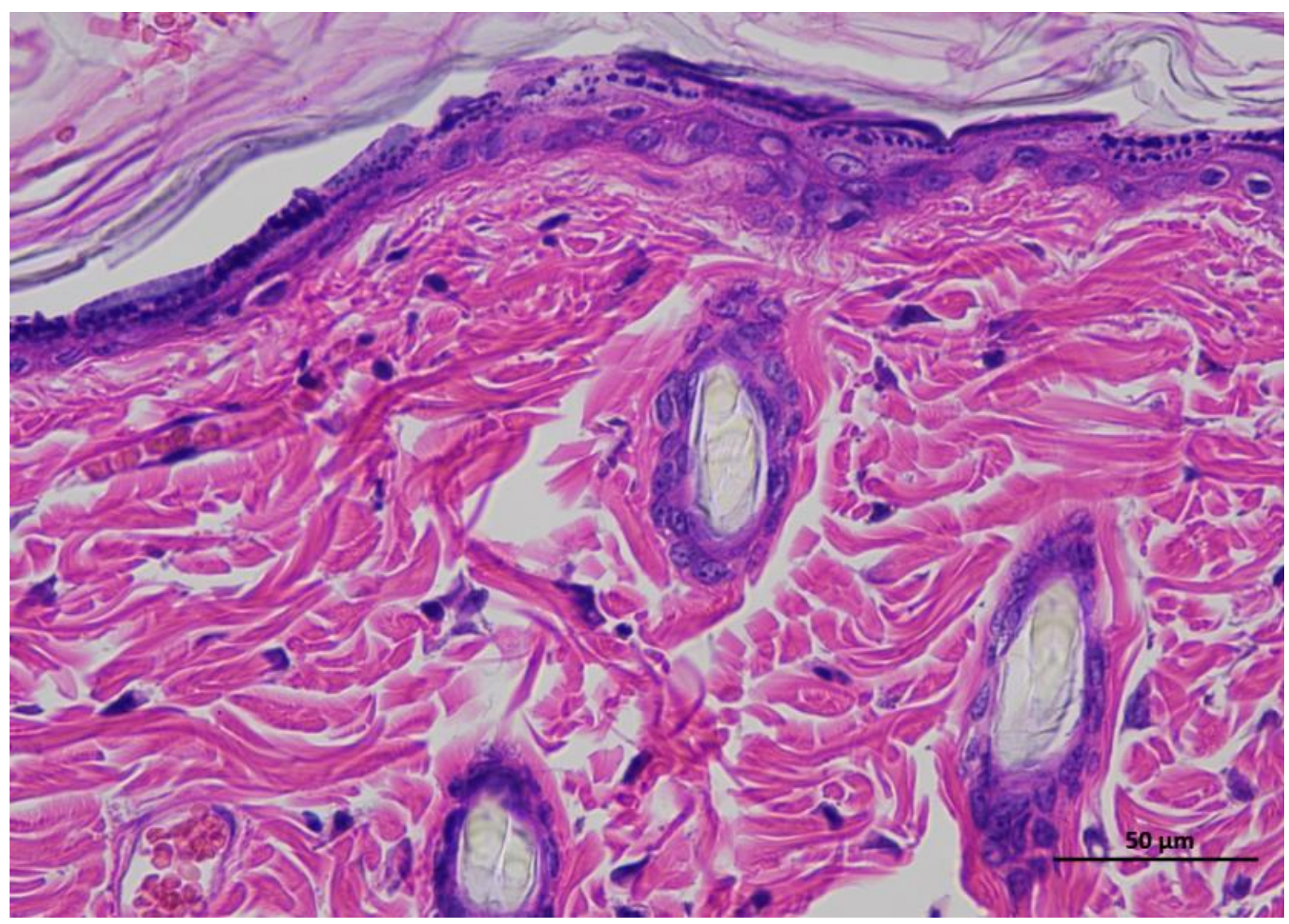

1 NControl 0day 400-2

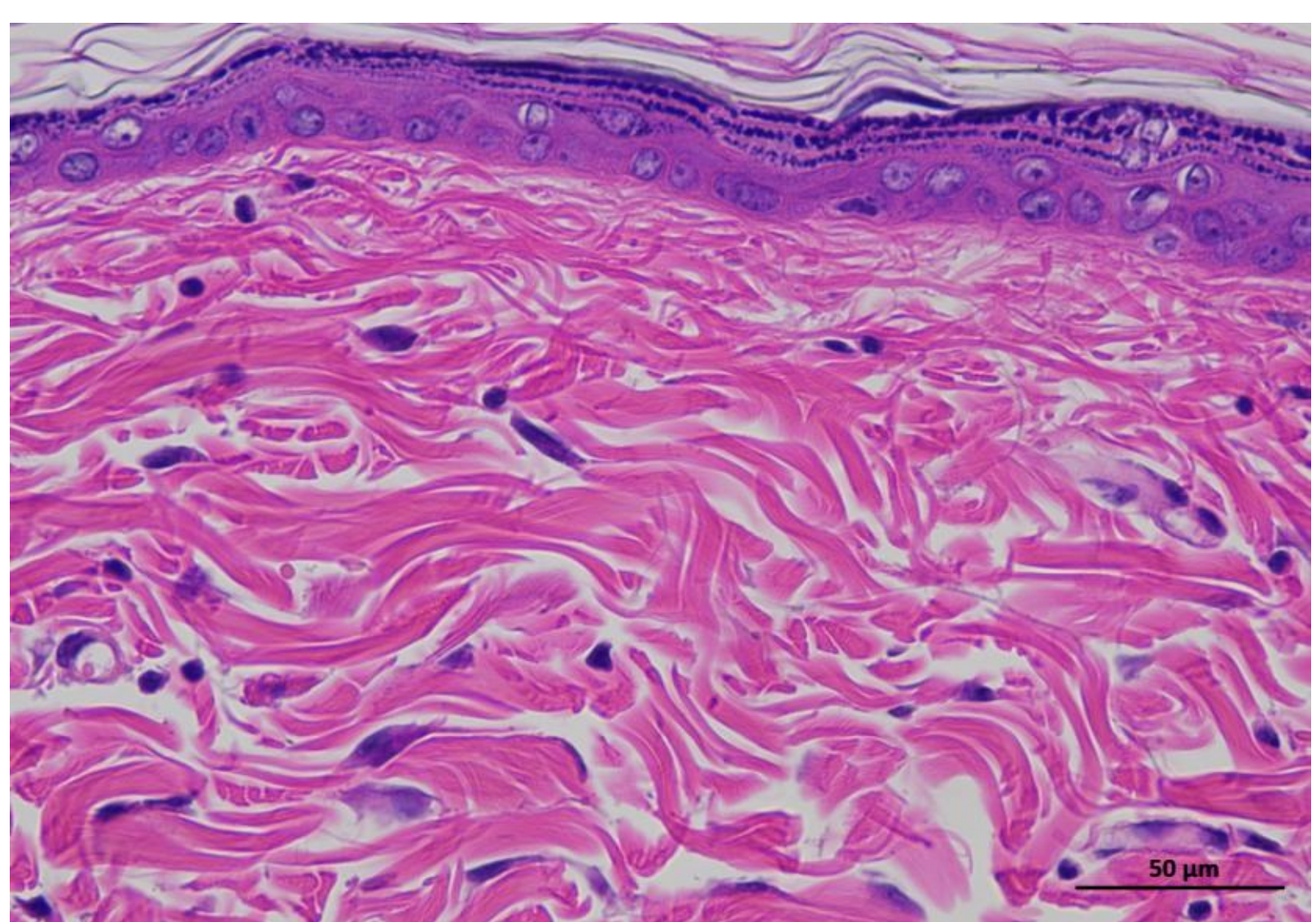

1 NControl 0day 400-3

Animal 2

Supplementary Figure 2a | Representative H&E staining of skin tissue of control animals / Day 0.

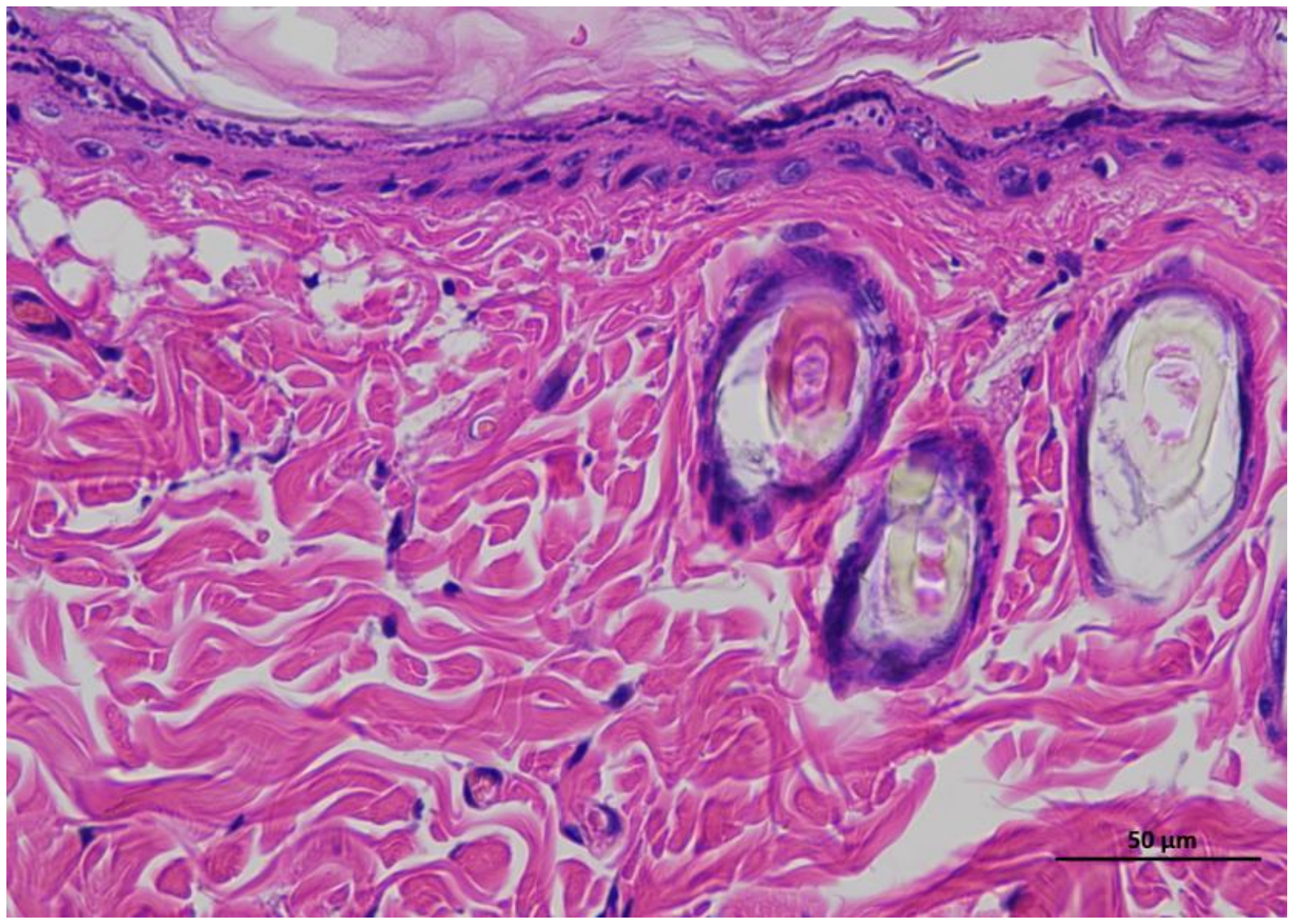

2 NControl 0day 400-1

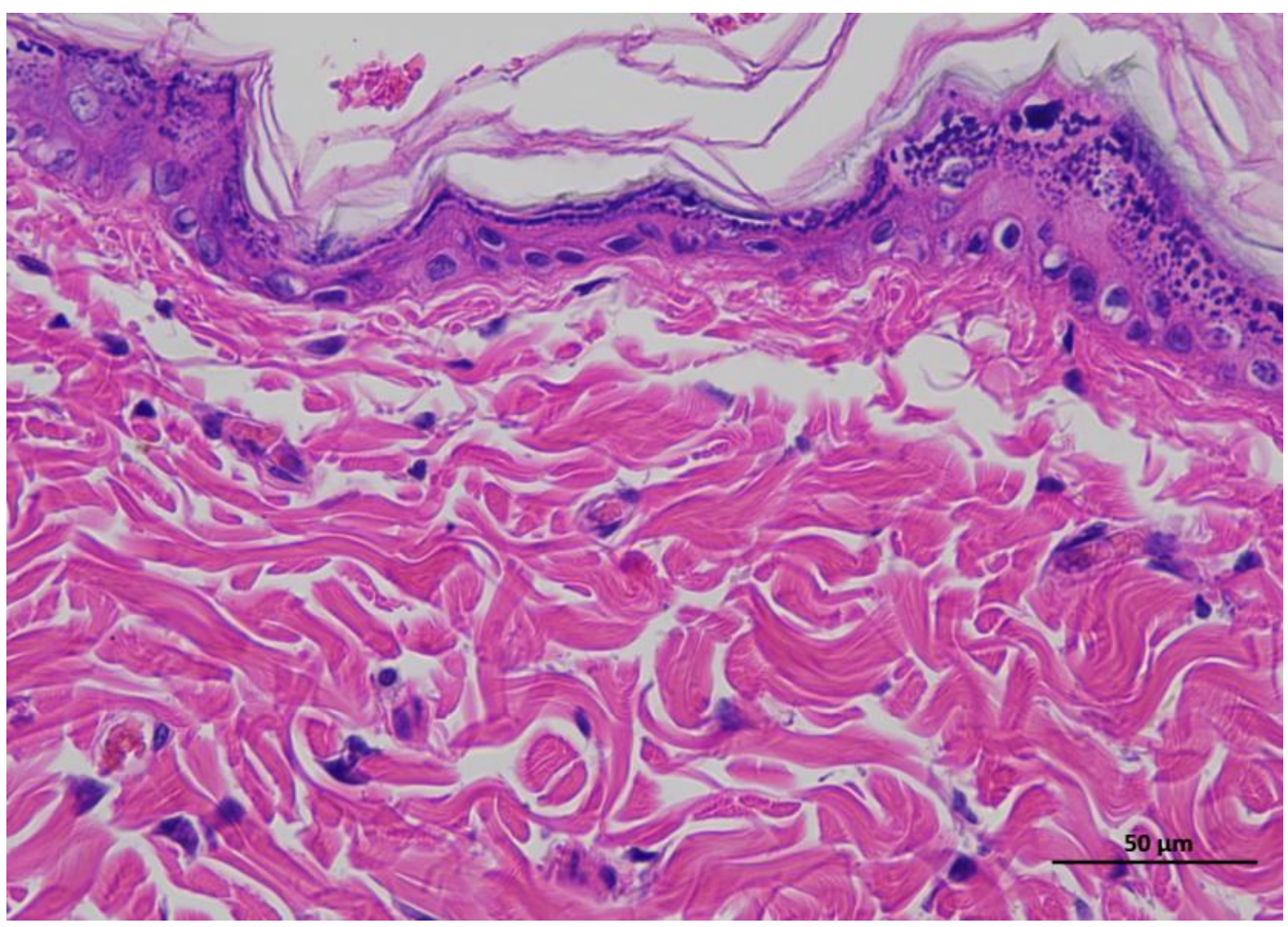

2 NControl 0day 400-2

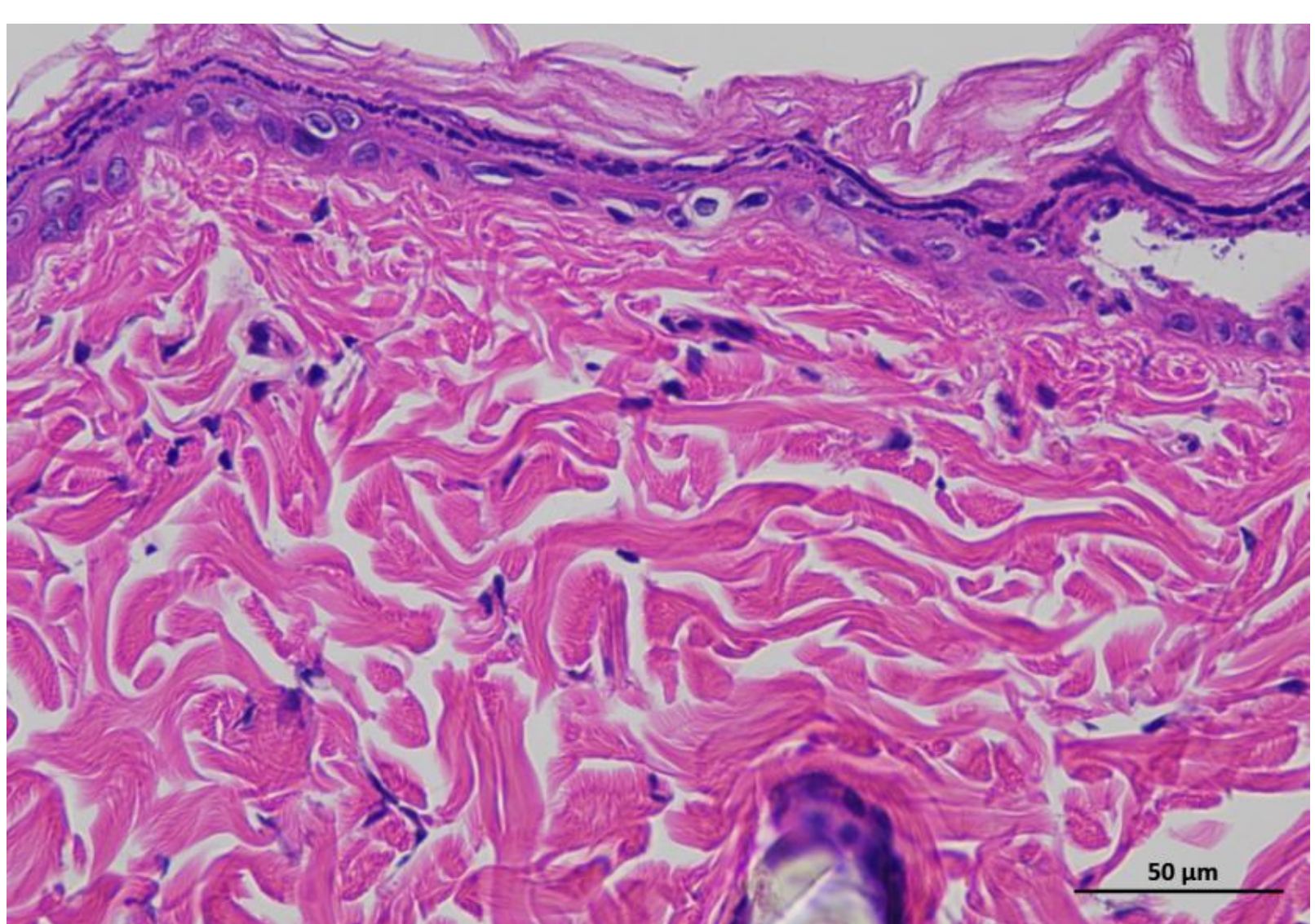

2 NControl 0day 400-3

Animal 3

Supplementary Figure 2a | Representative H&E staining of skin tissue of control animals / Day 0.

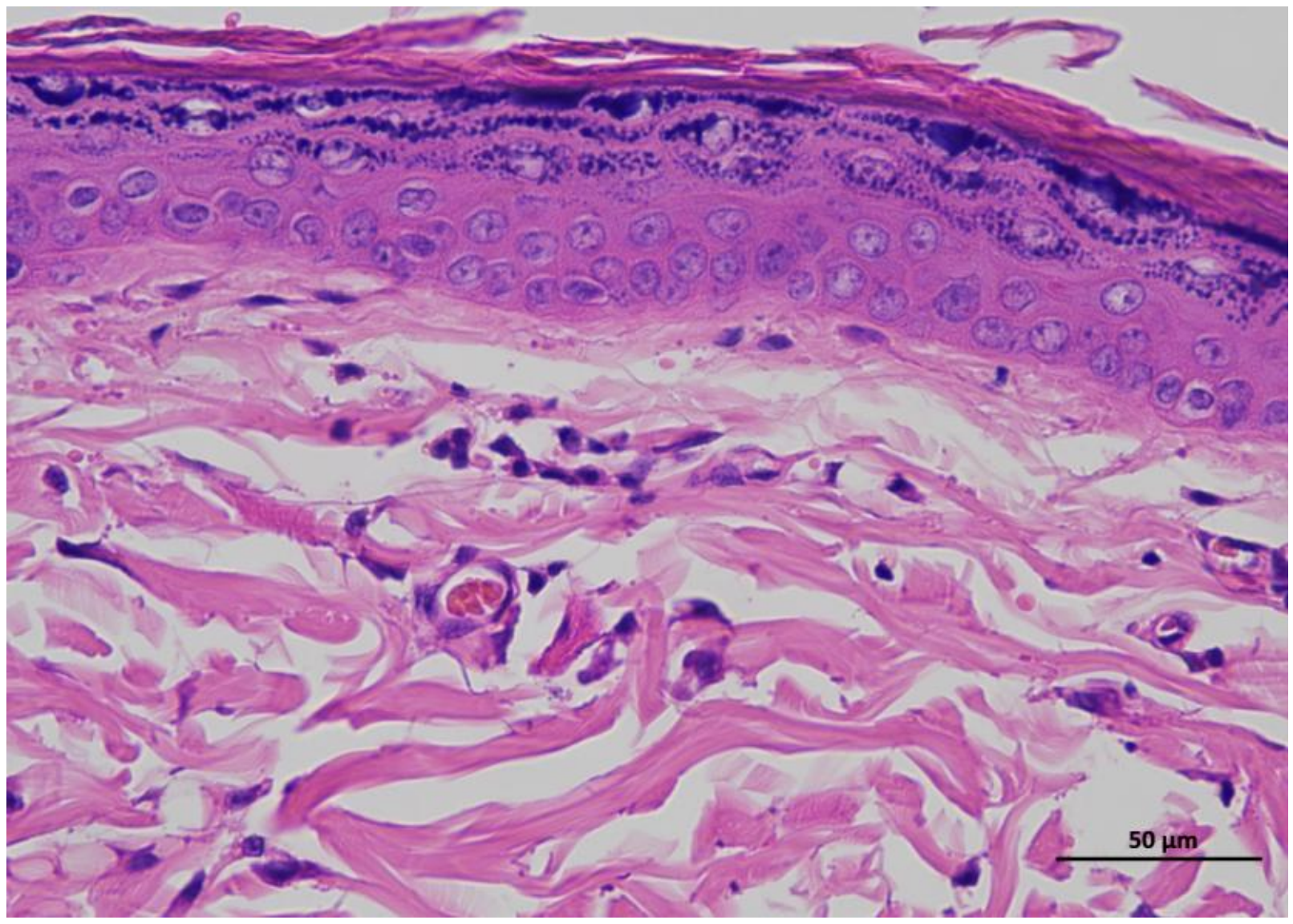

3 NControl 0day 400-1

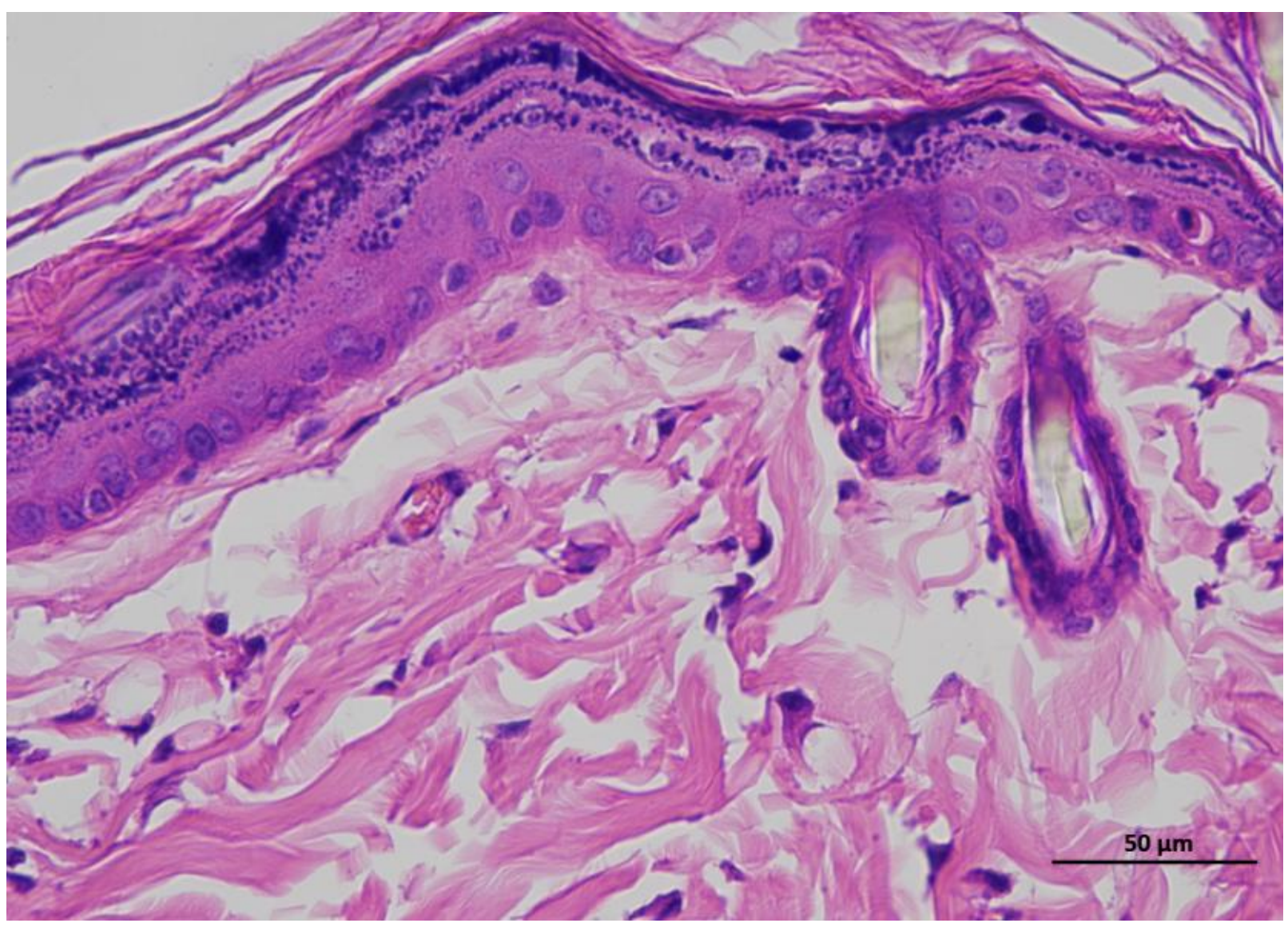

3 NControl 0day 400-2

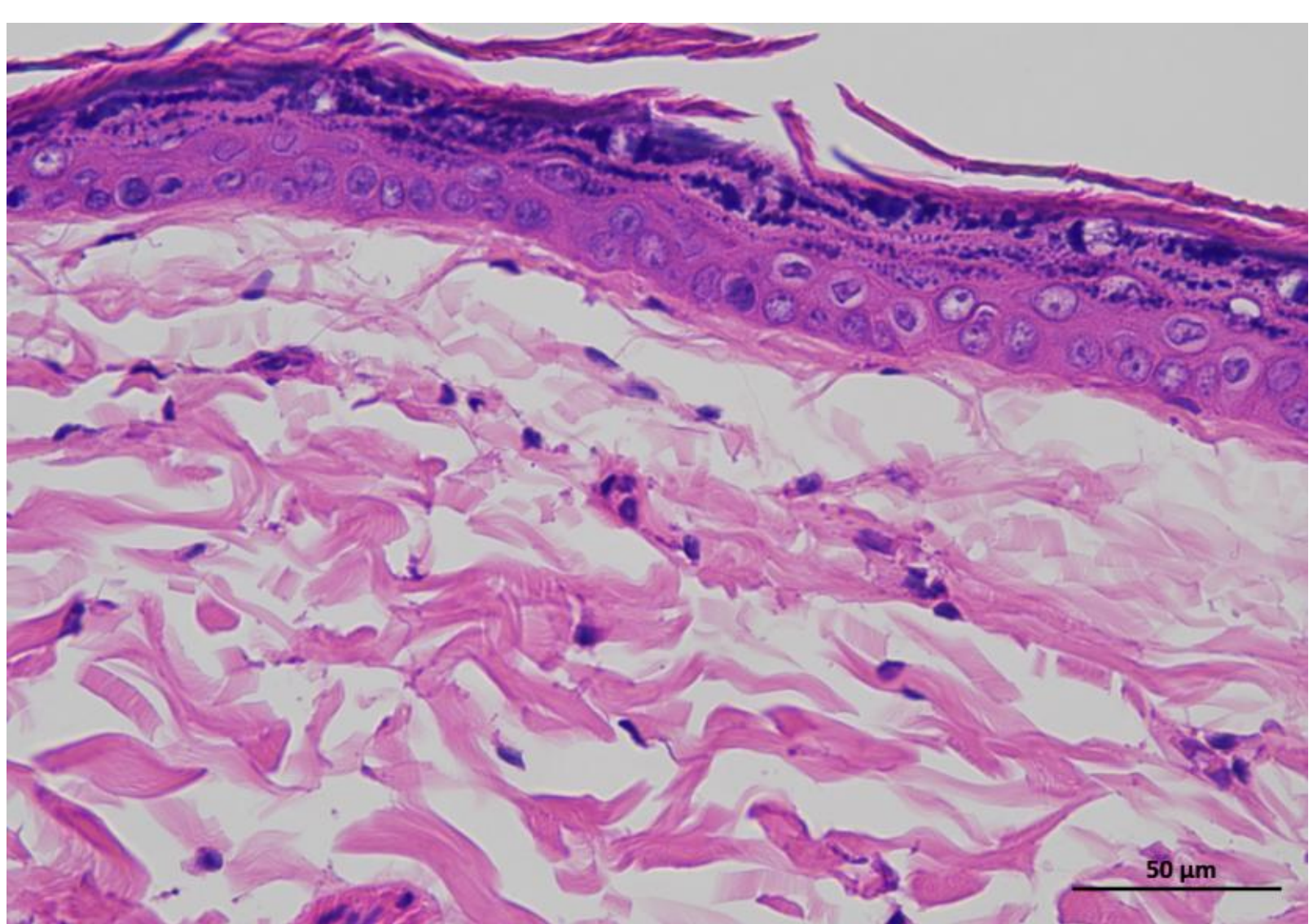

3 NControl 0day 400-3

Animal 4

Supplementary Figure 2a | Representative H&E staining of skin tissue of control animals / Day 0.

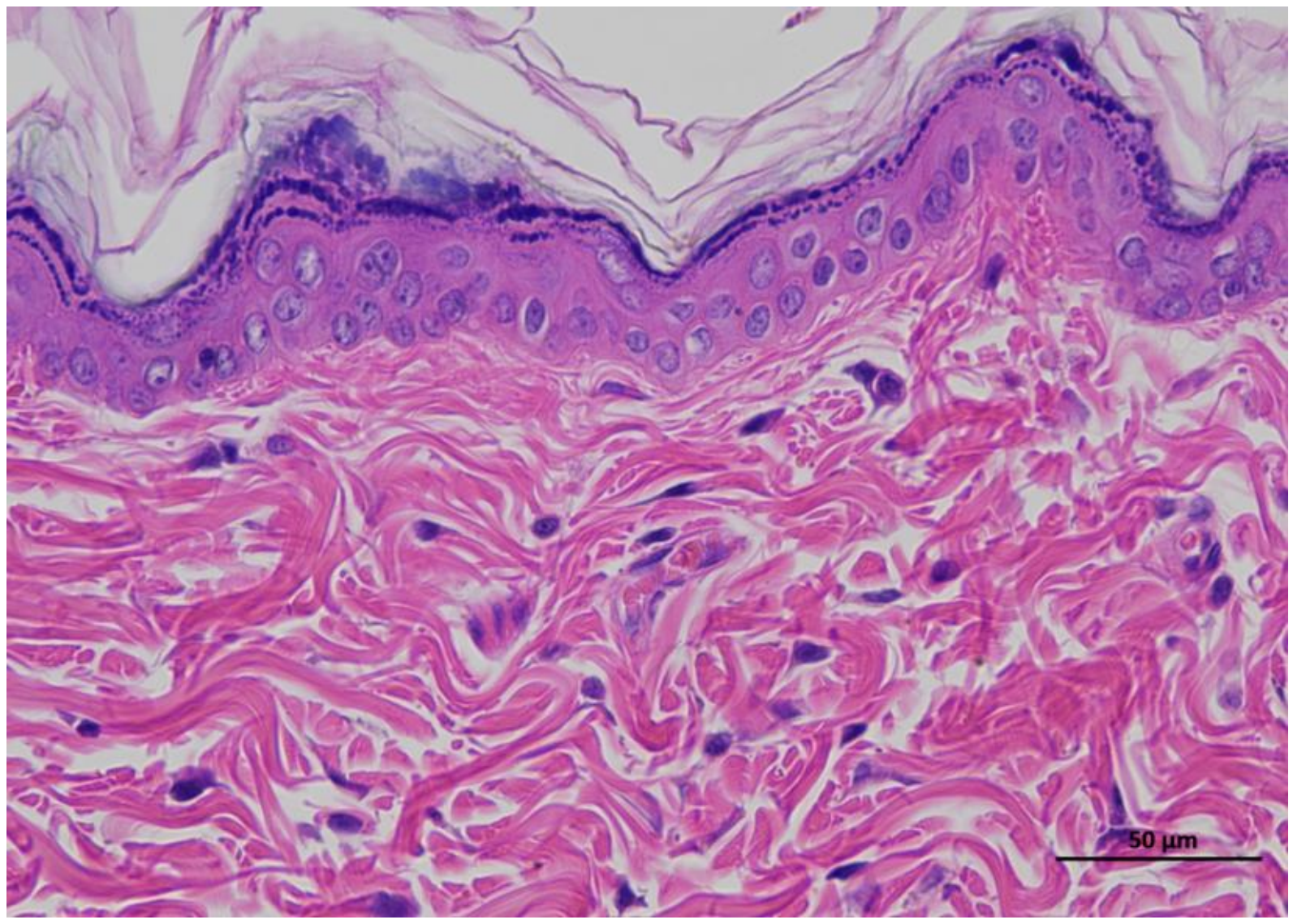

4 NControl 0day 400-1

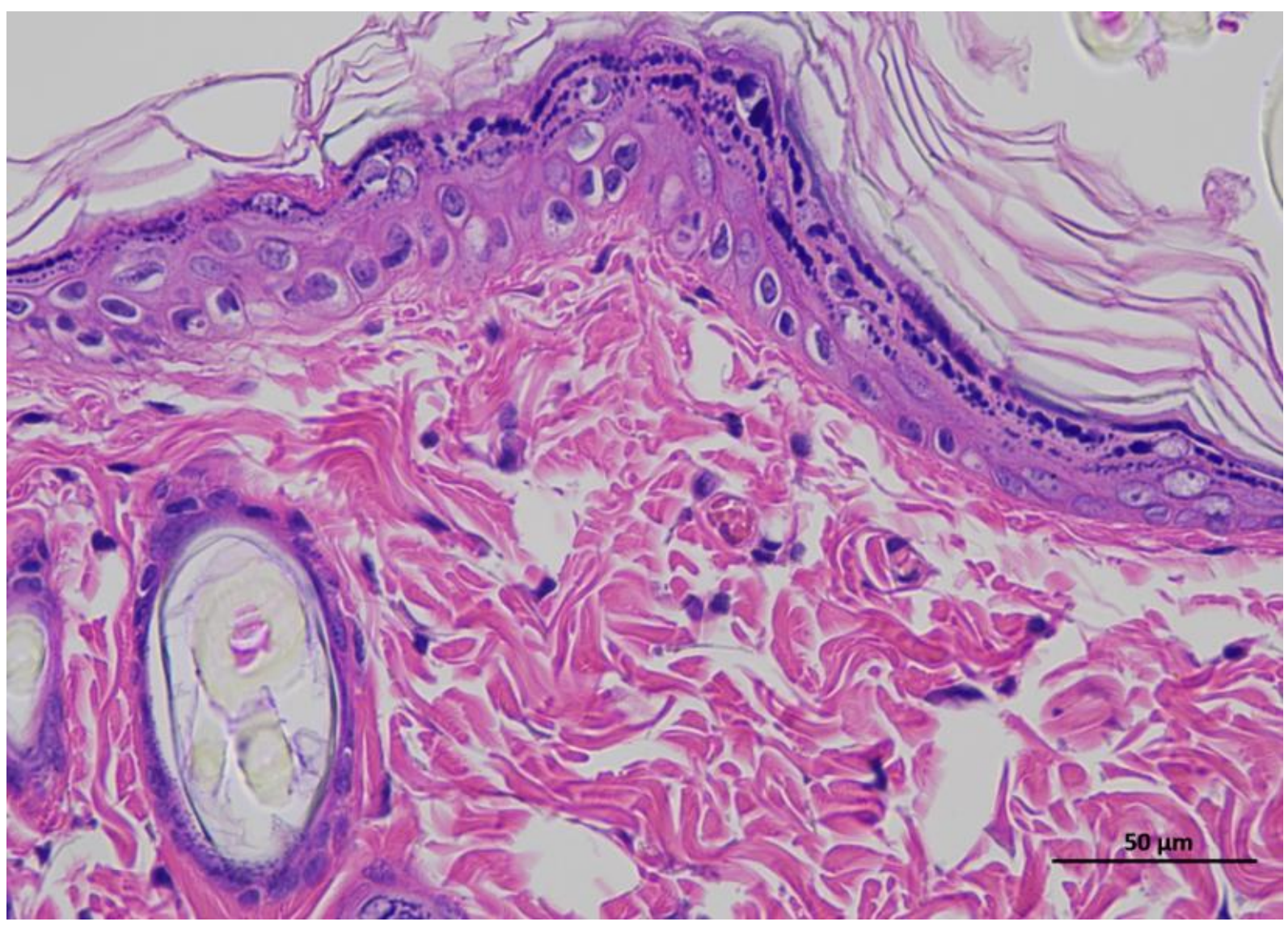

4 NControl 0day 400-2

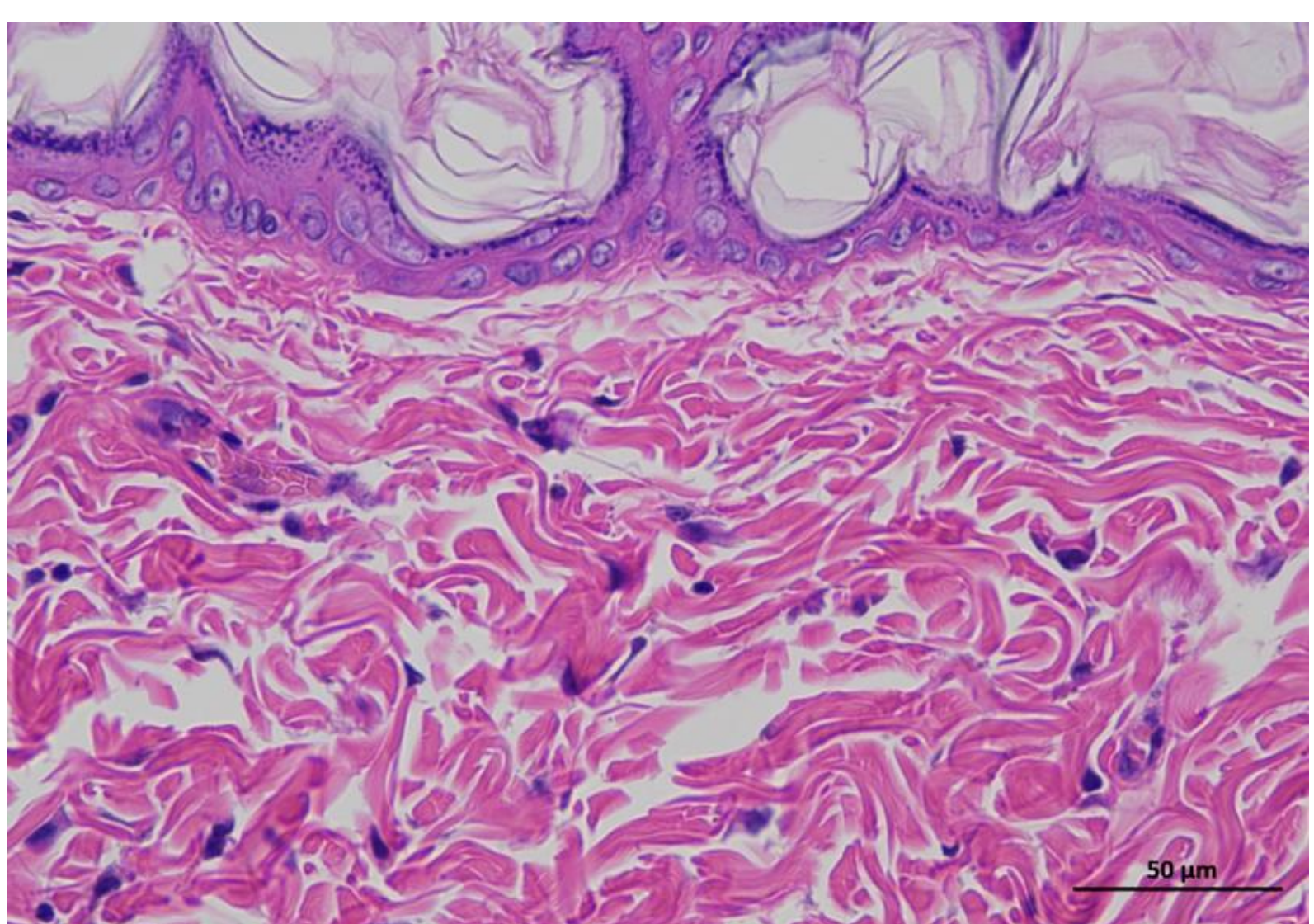

4 NControl 0day 400-3

Animal 5

Supplementary Figure 2a | Representative H&E staining of skin tissue of control animals / Day 0.

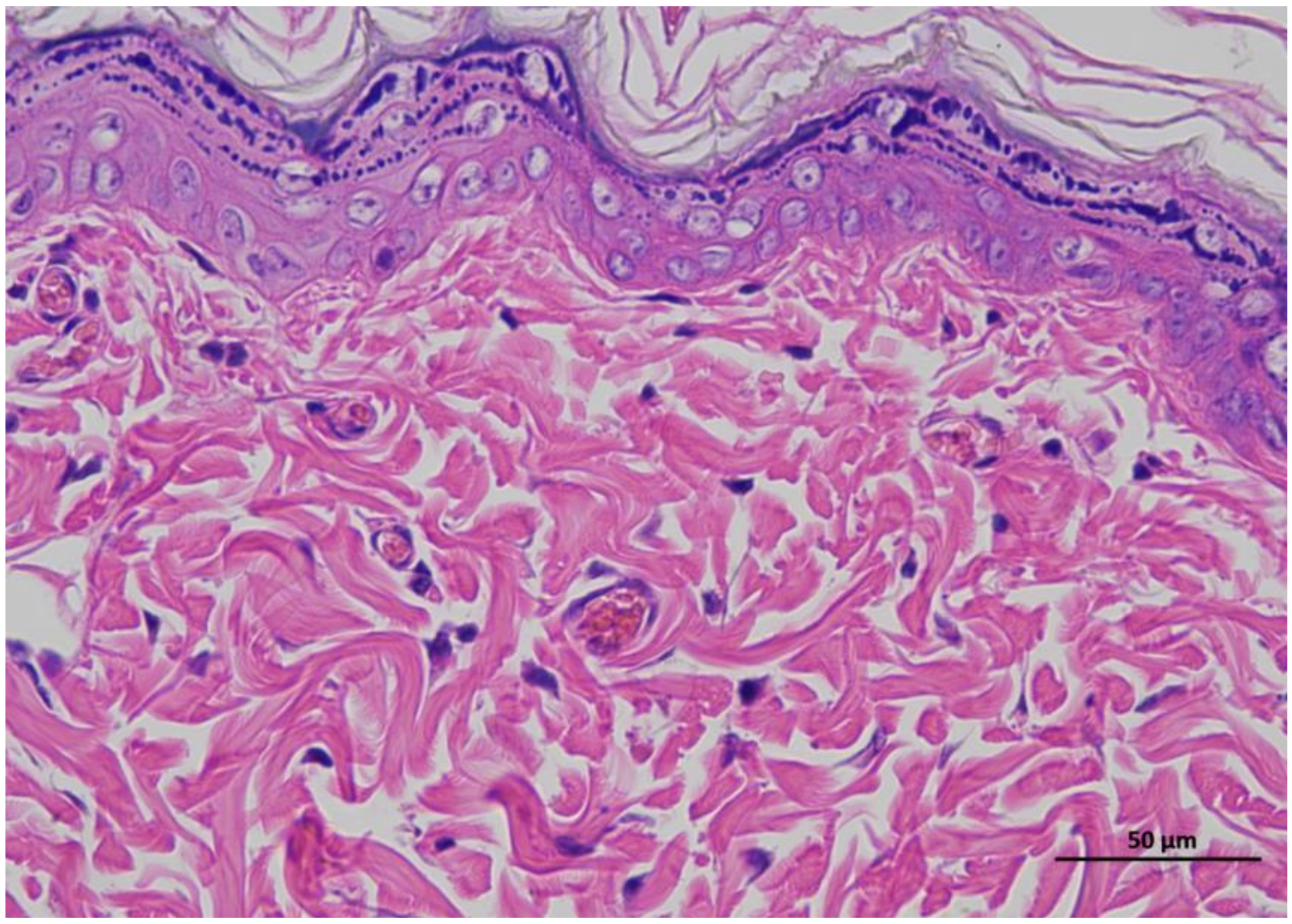

5 NControl 0day 400-1

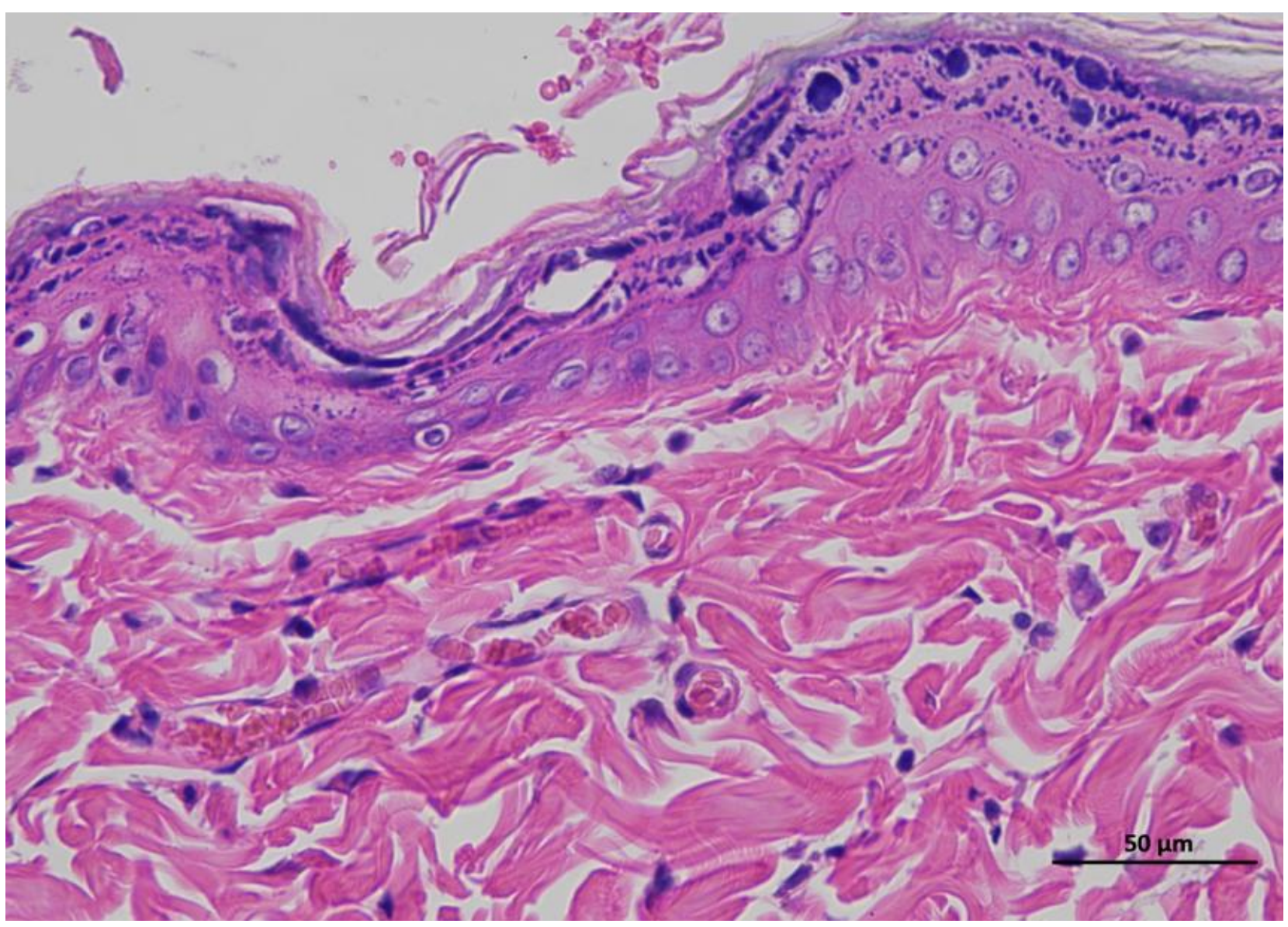

5 NControl 0day 400-2

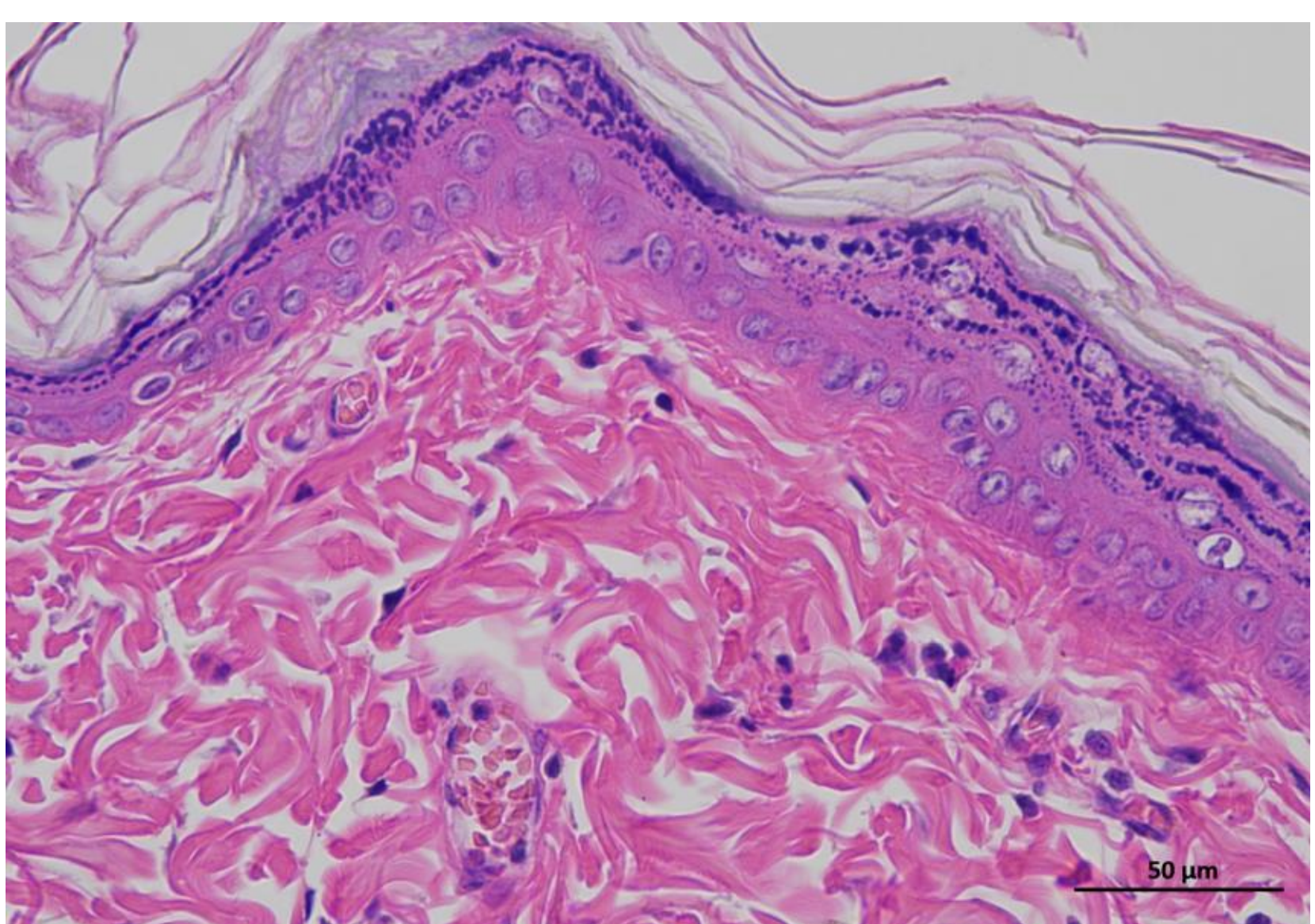

5 NControl 0day 400-3

Supplementary Figure 2a | Representative H&E staining of skin tissue of control animals / Day 0.

Animal 6

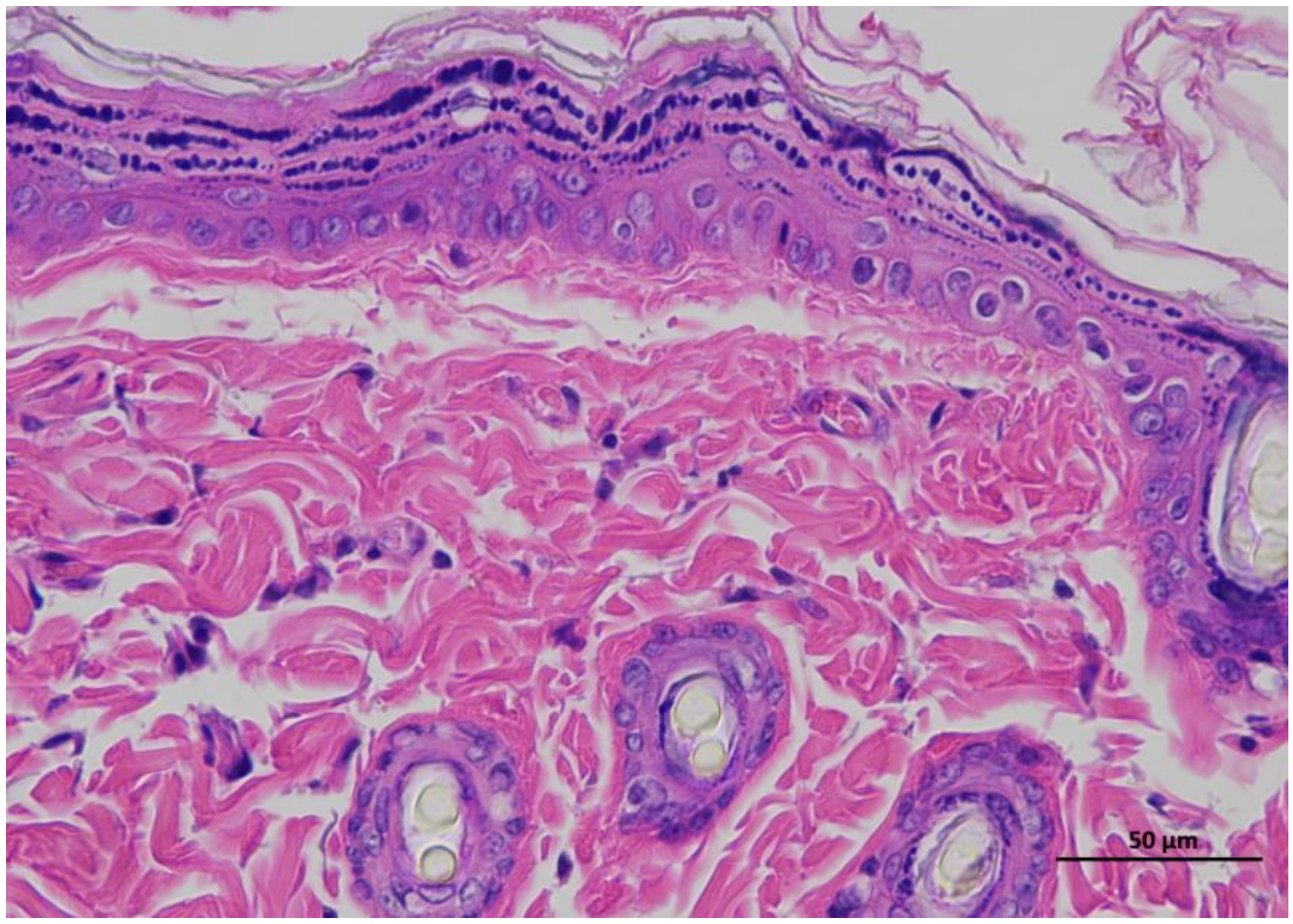

6 NControl 0day 400-1

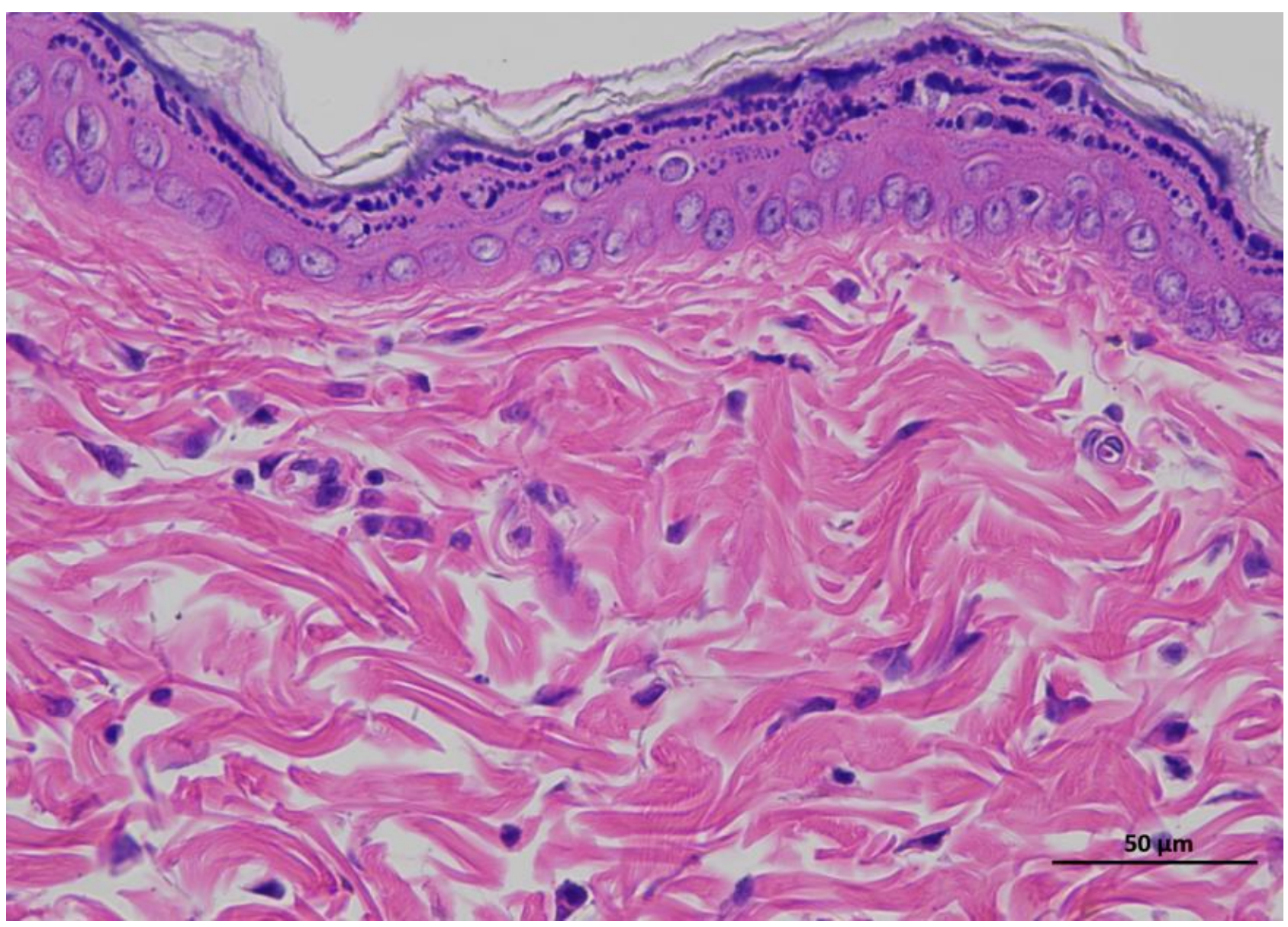

6 NControl 0day 400-2

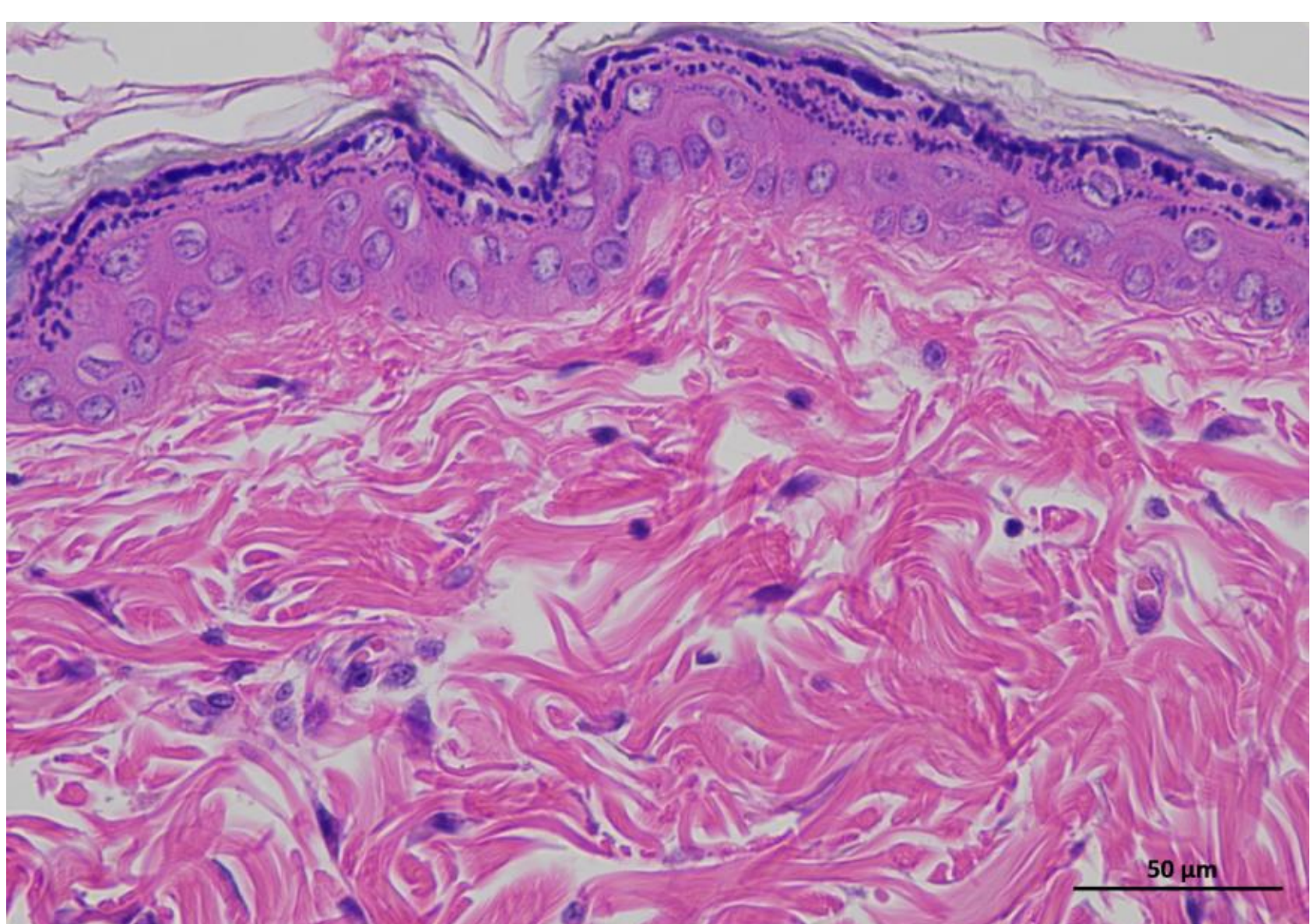

6 NControl 0day 400-3

Animal 1

Supplementary Figure 3a | Representative H&E staining of skin tissue of control animals / Day 14. Representative hematoxylin and eosin (H&E)–stained section of skin tissue collected from experimental rat. The image shows the typical architecture of the skin, including the epidermal layer with stratified keratinized epithelium and the underlying dermis composed of dense collagen fibers and connective tissue structures.
(n = 6 per group).

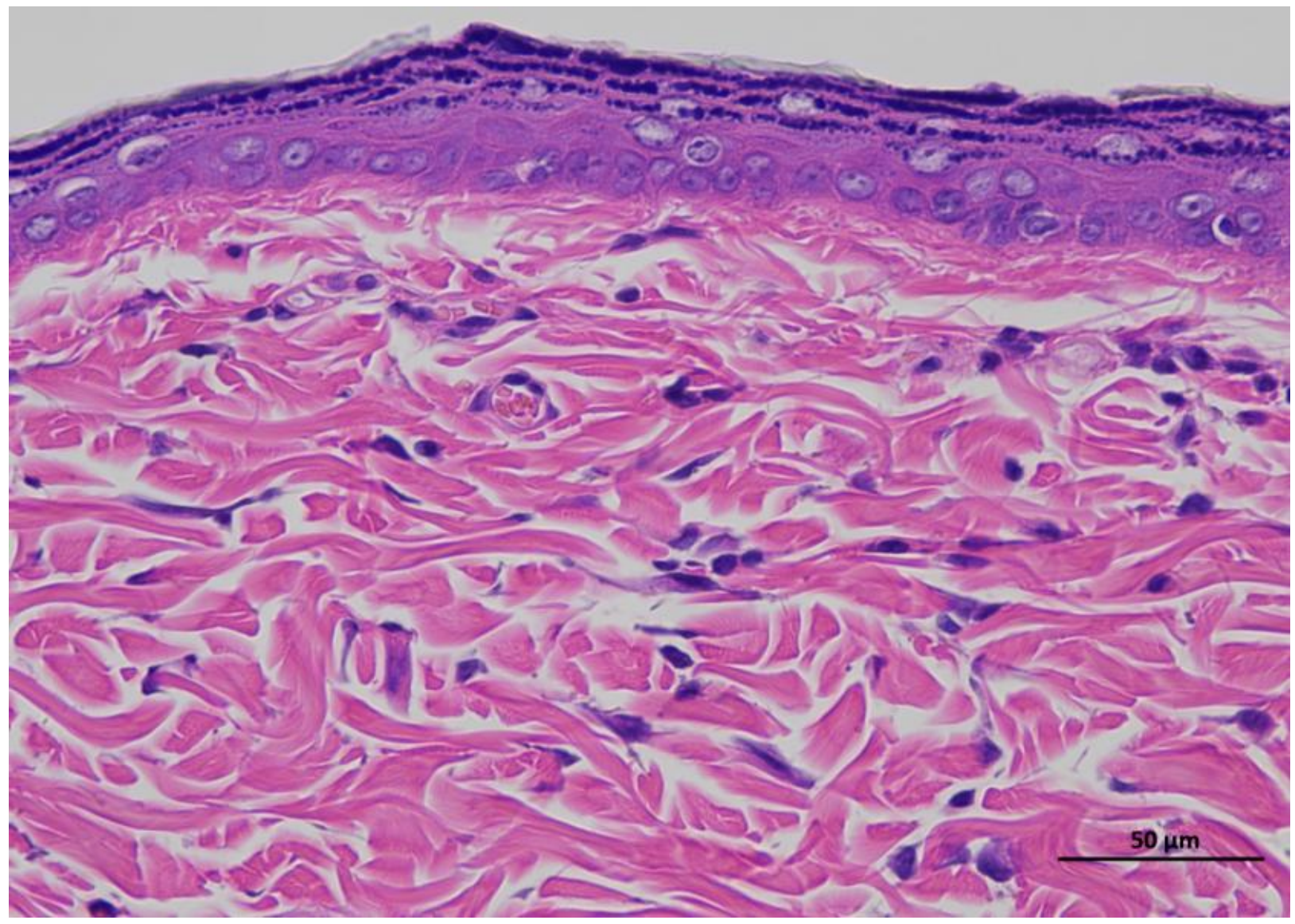

1 NControl 14day 400-1

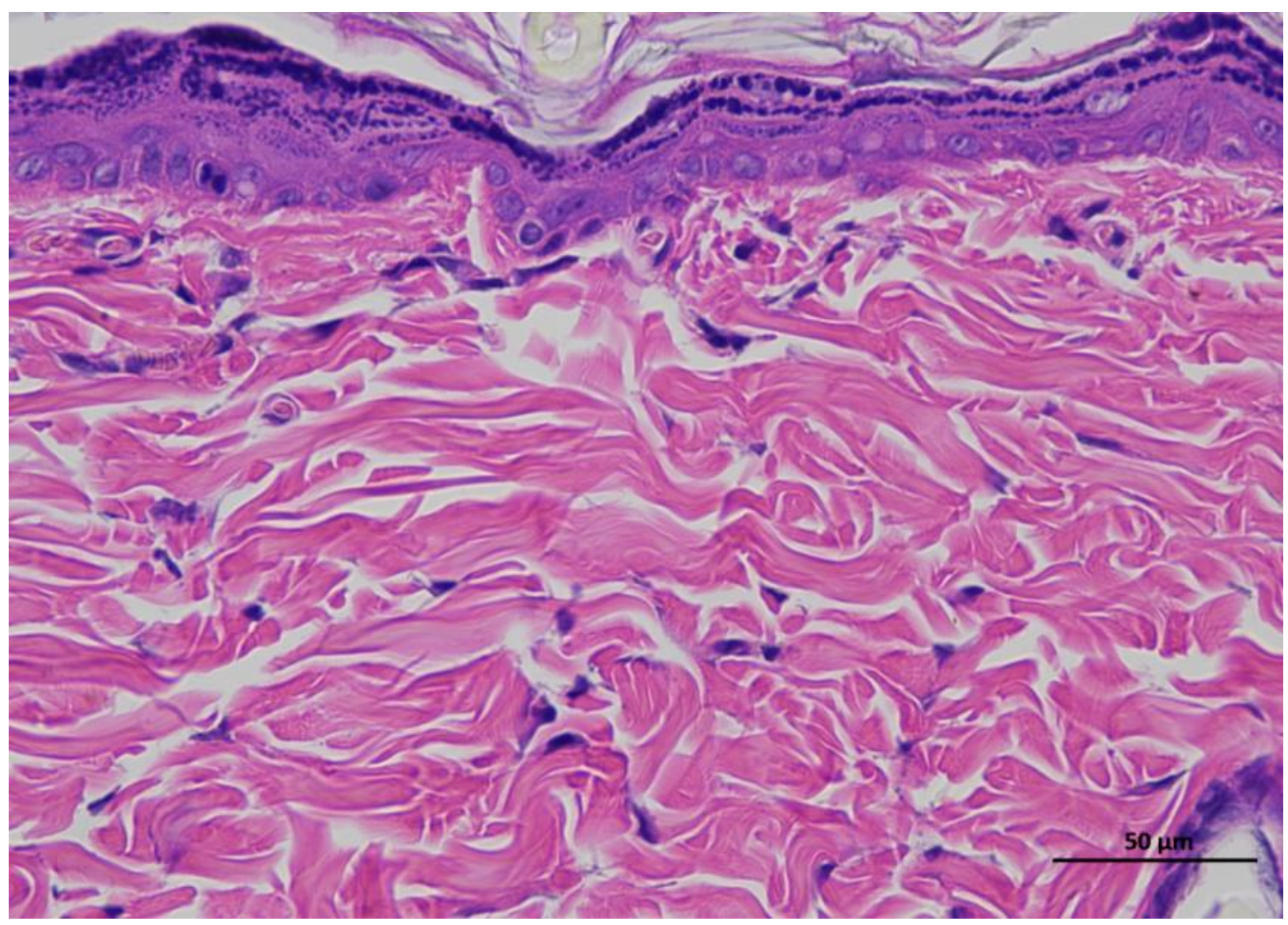

1 NControl 14day 400-2

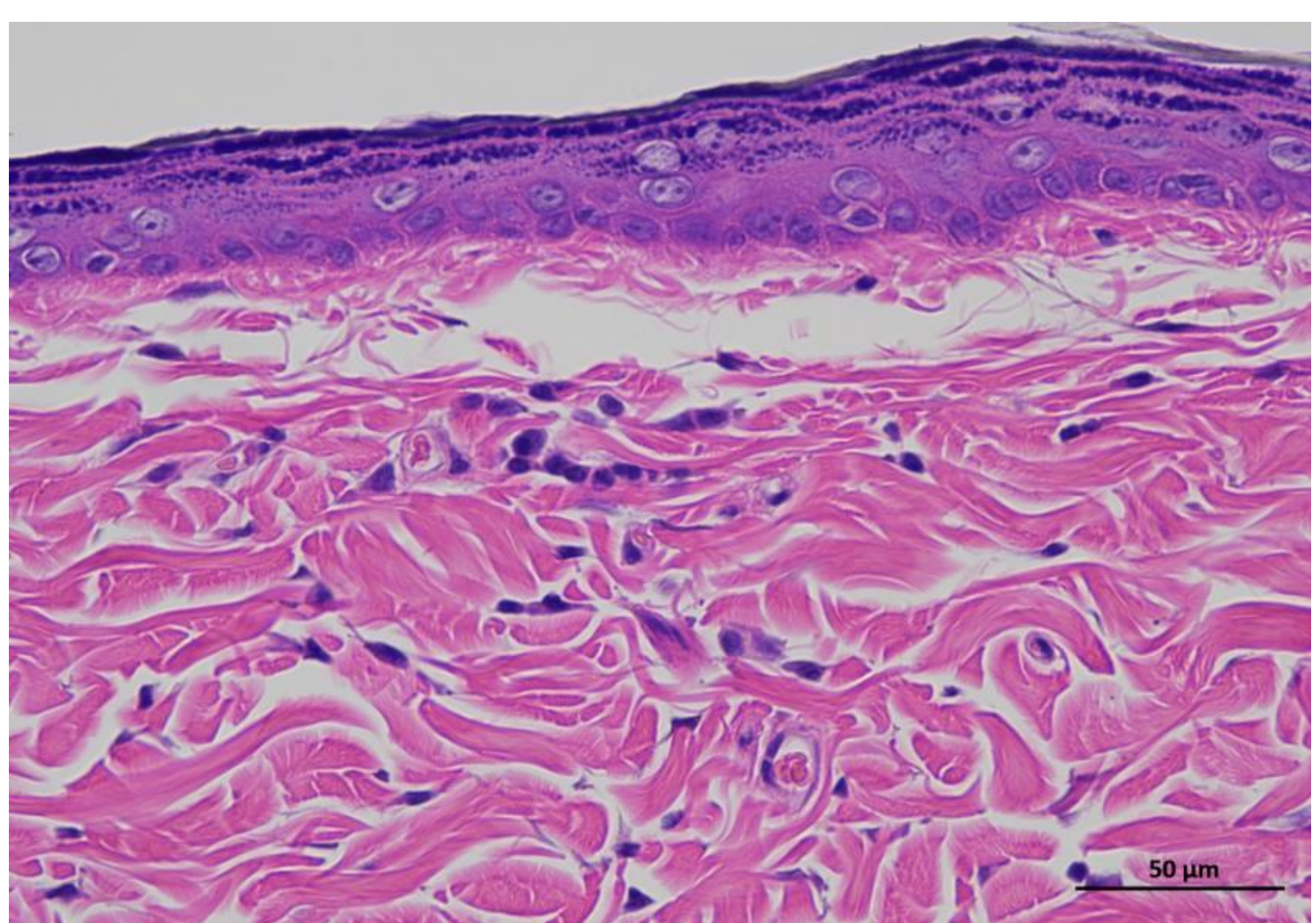

1 NControl 14day 400-3

Animal 2

Supplementary Figure 3a | Representative H&E staining of skin tissue of control animals / Day 14.

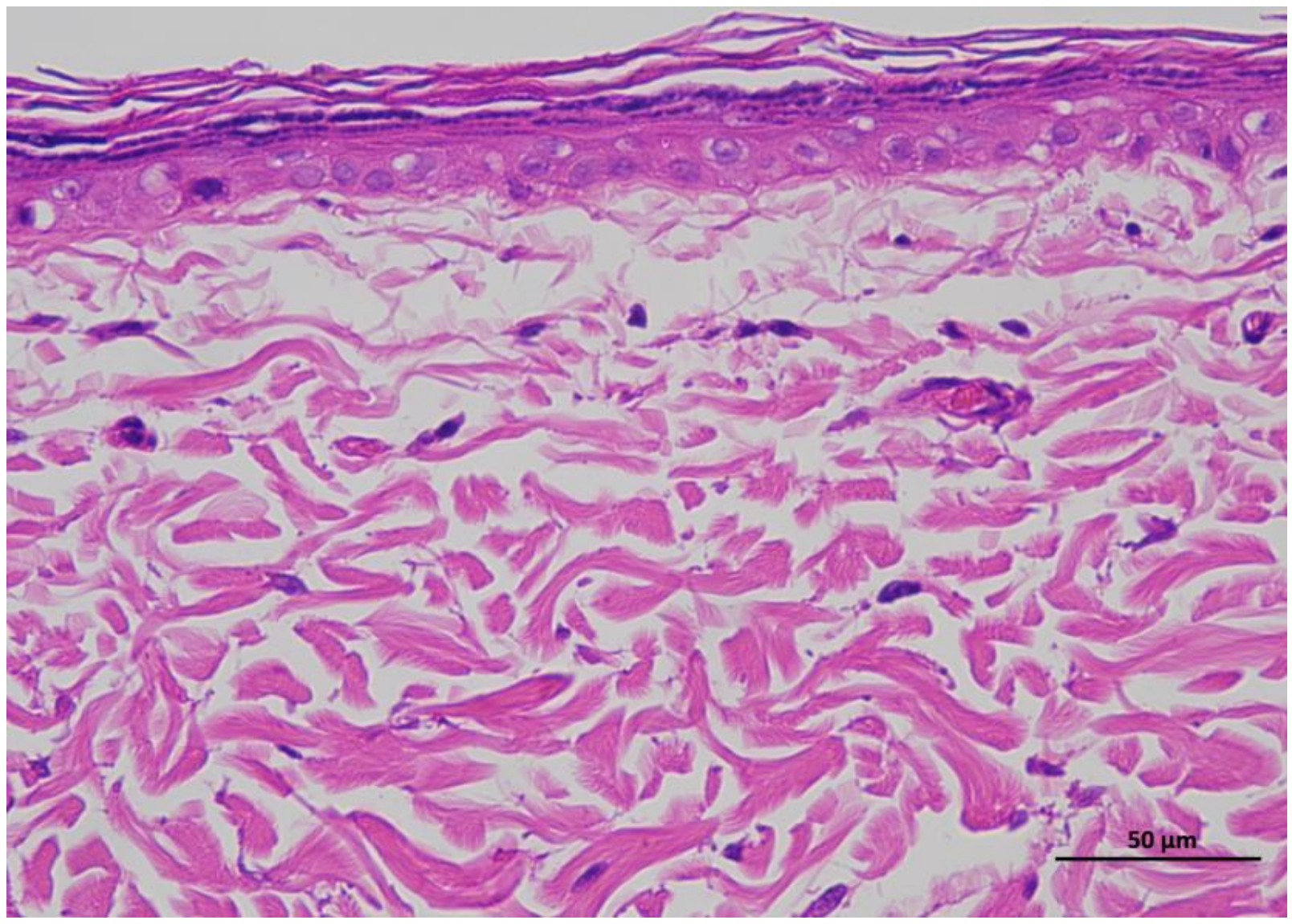

2 NControl 14day 400-1

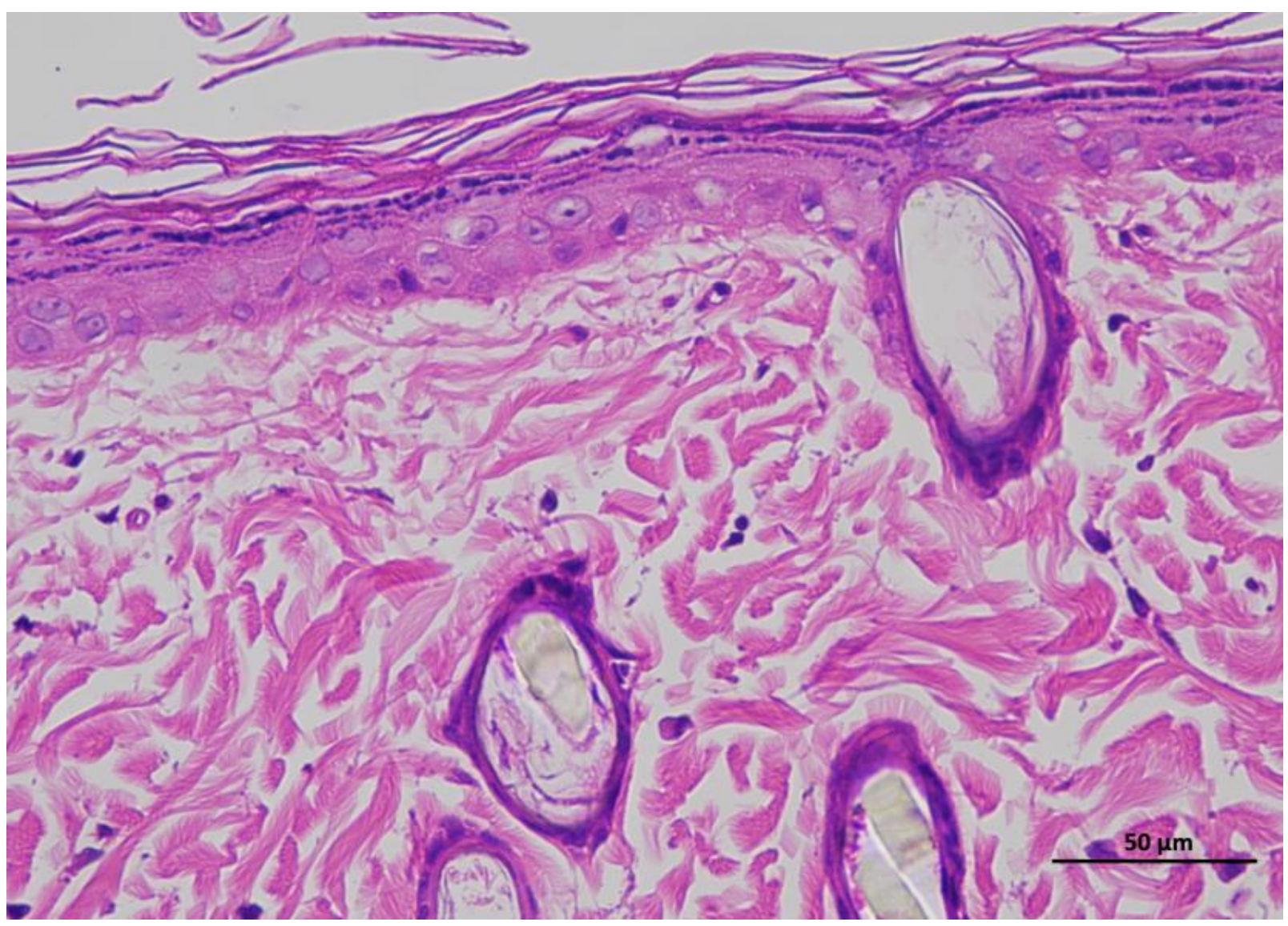

2 NControl 14day 400-2

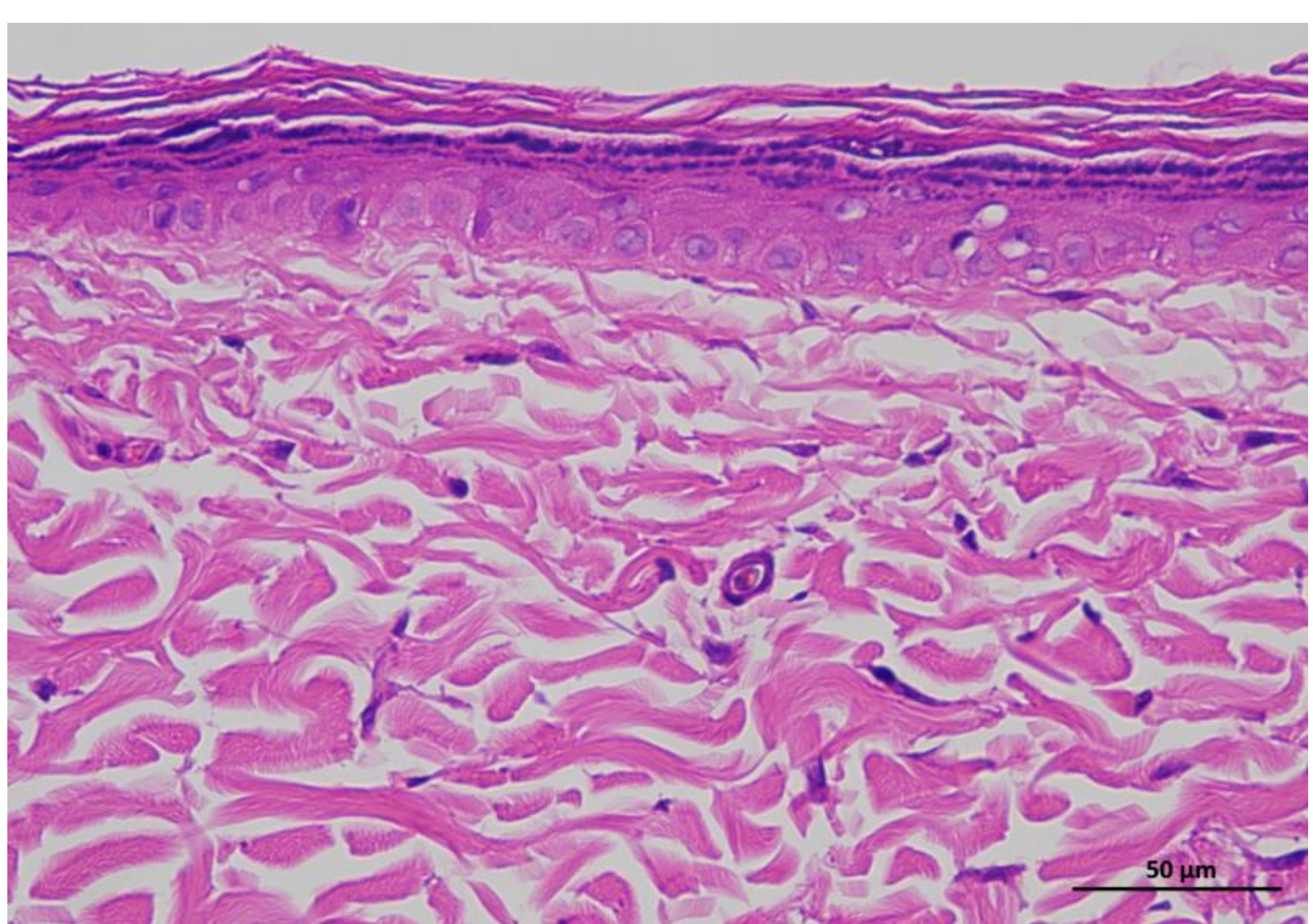

2 NControl 14day 400-3

Animal 3

Supplementary Figure 3a | Representative H&E staining of skin tissue of control animals / Day 14.

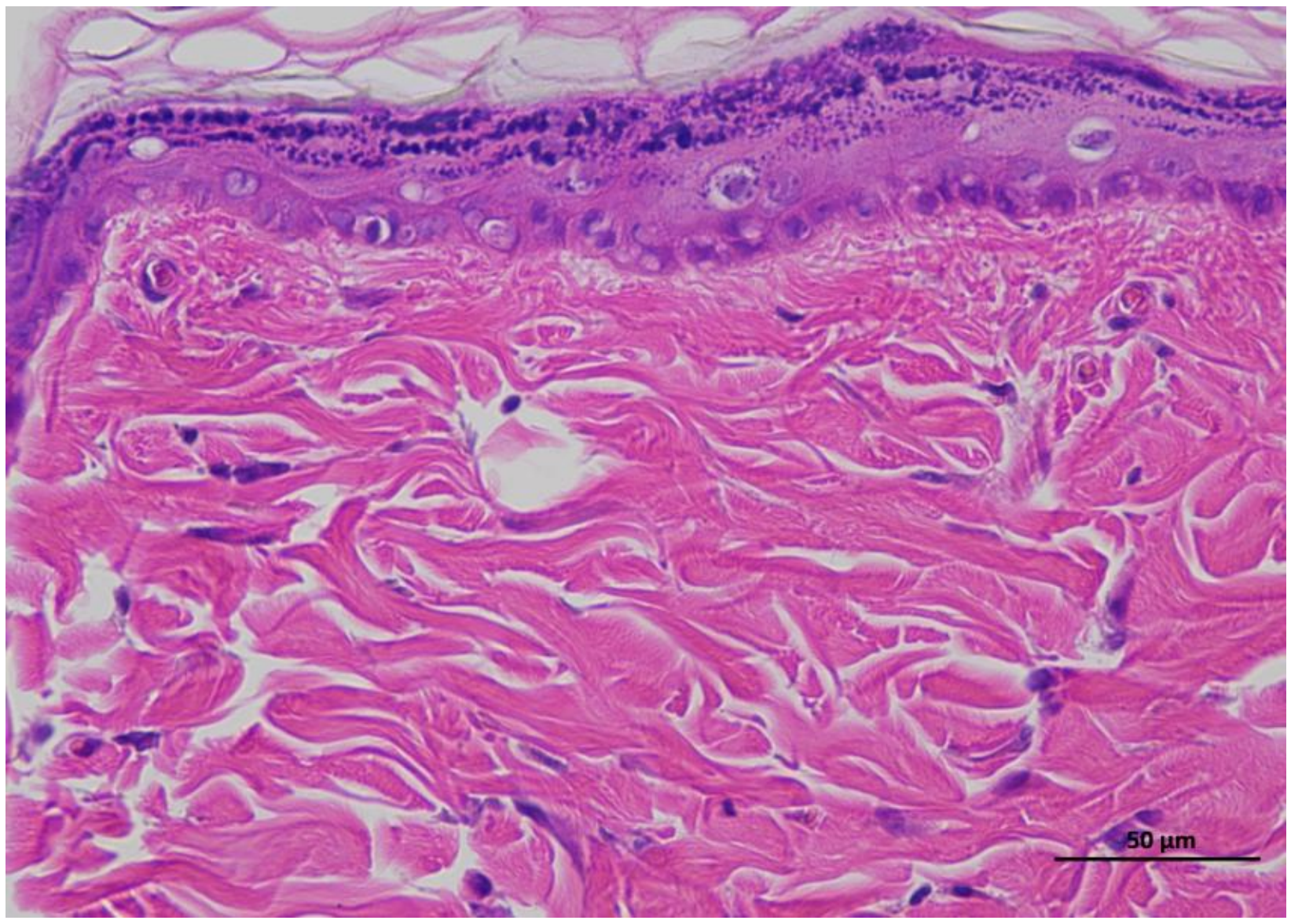

3 NControl 14day 400-1

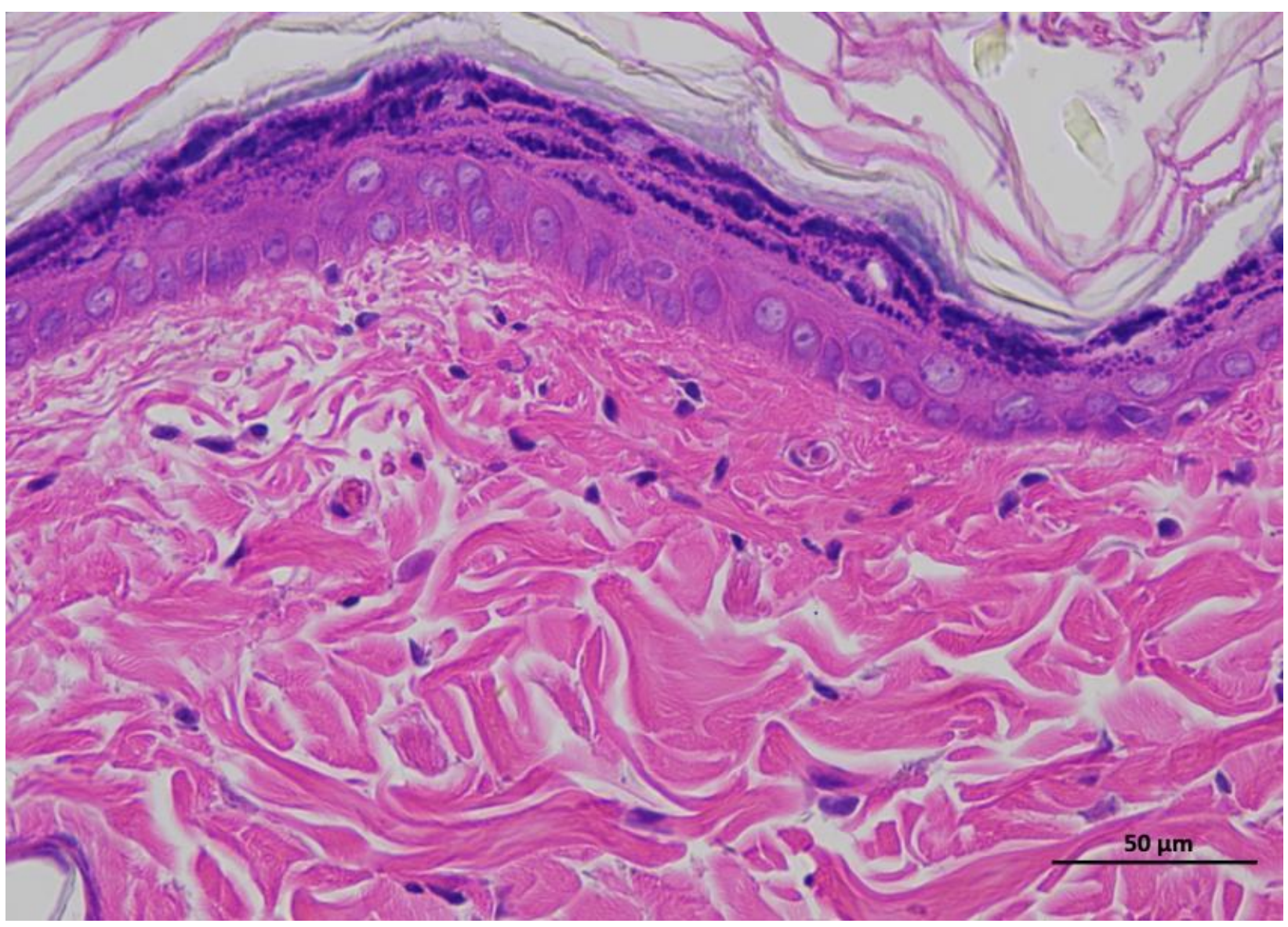

3 NControl 14day 400-2

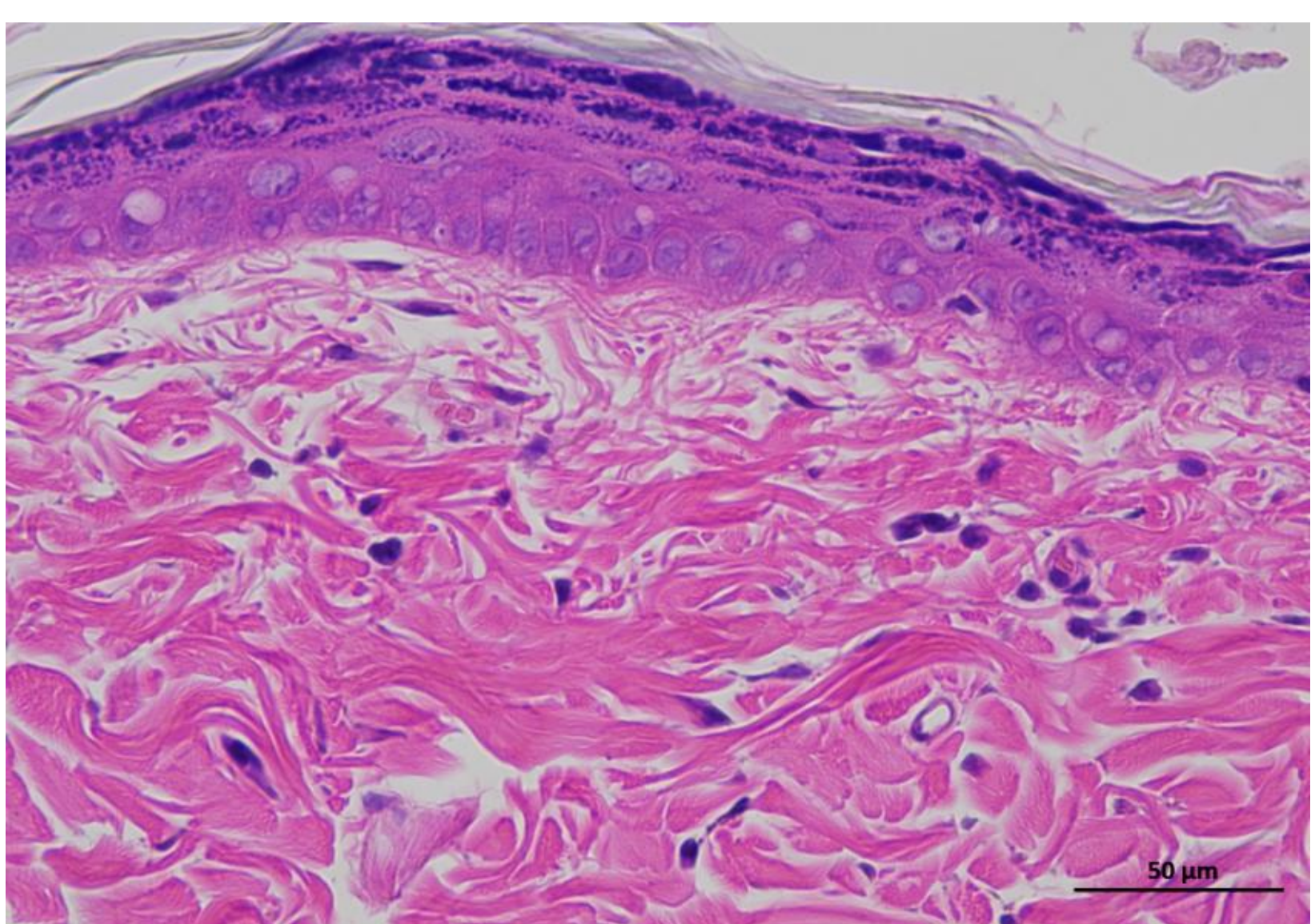

3 NControl 14day 400-3

Animal 4

Supplementary Figure 3a | Representative H&E staining of skin tissue of control animals / Day 14.

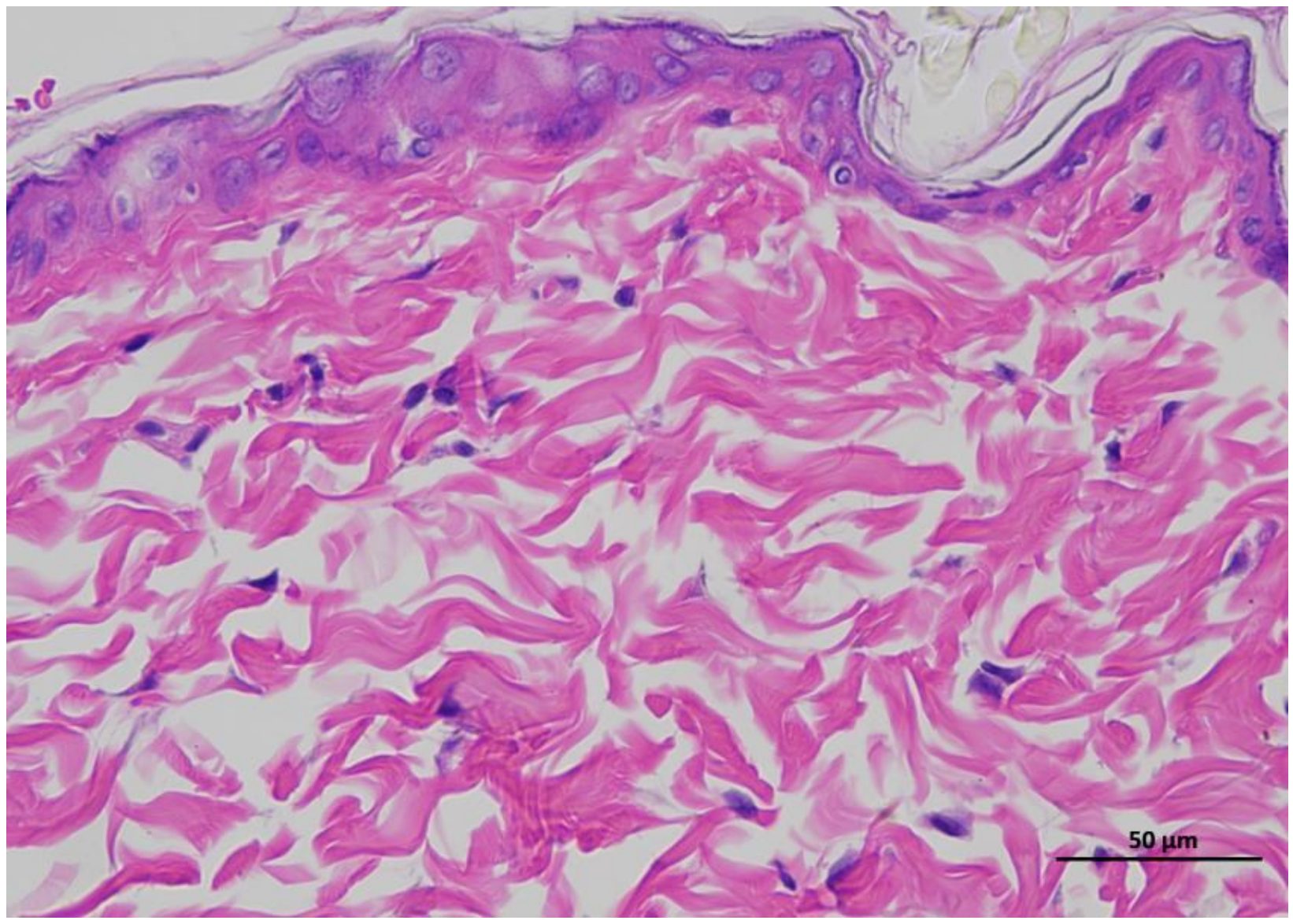

4 NControl 14day 400-1

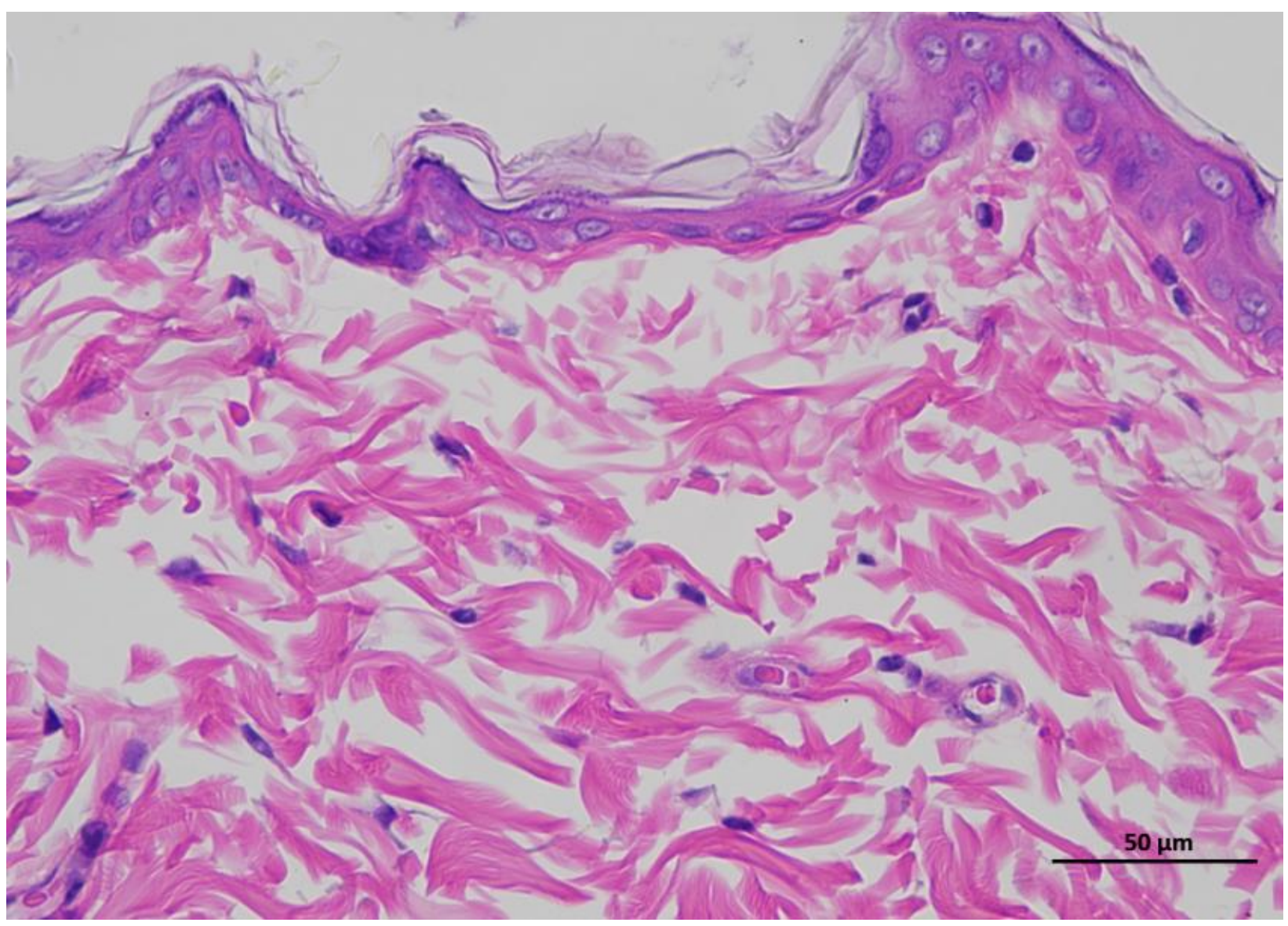

4 NControl 14day 400-2

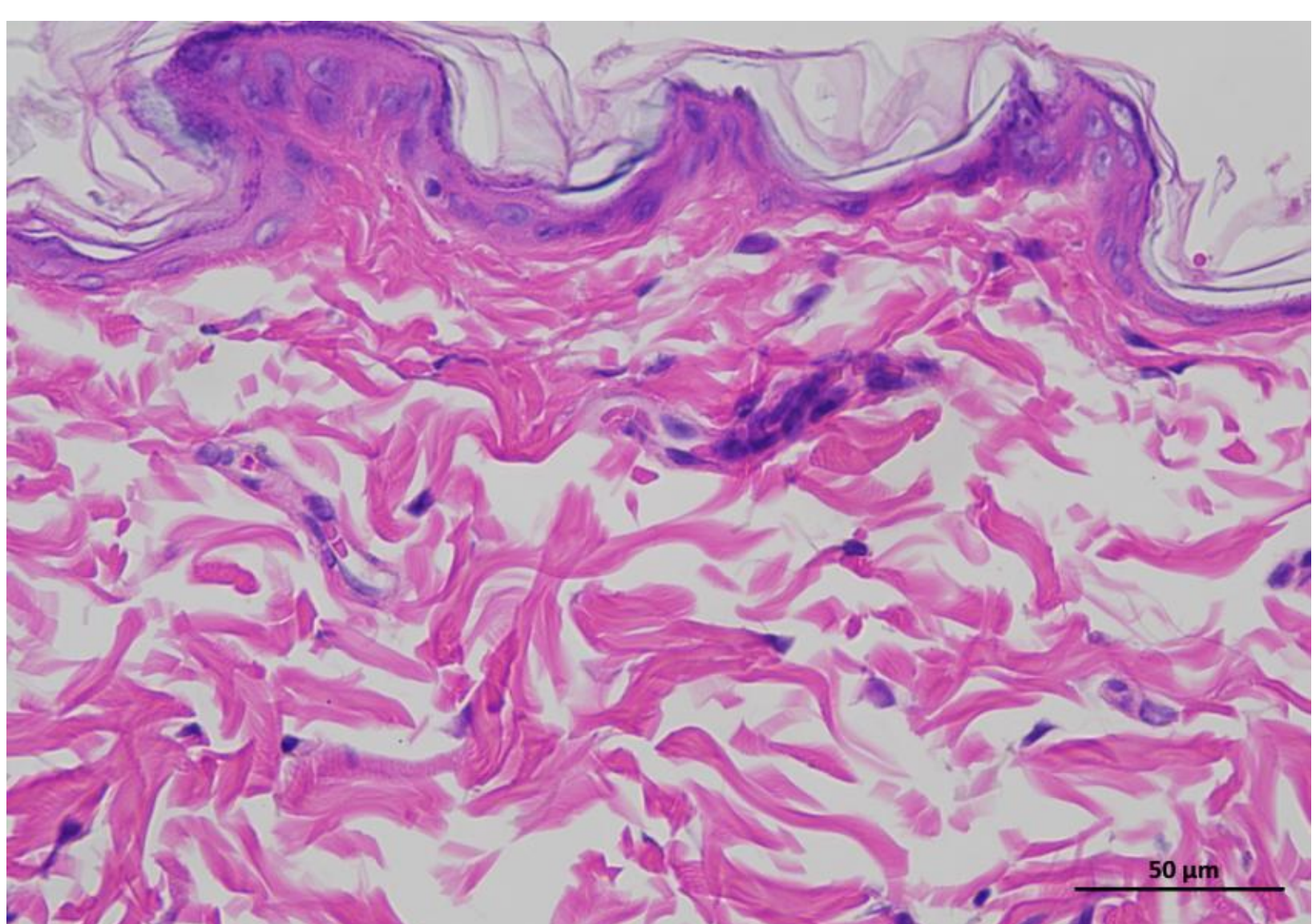

4 NControl 14day 400-3

Animal 5

Supplementary Figure 3a | Representative H&E staining of skin tissue of control animals / Day 14.

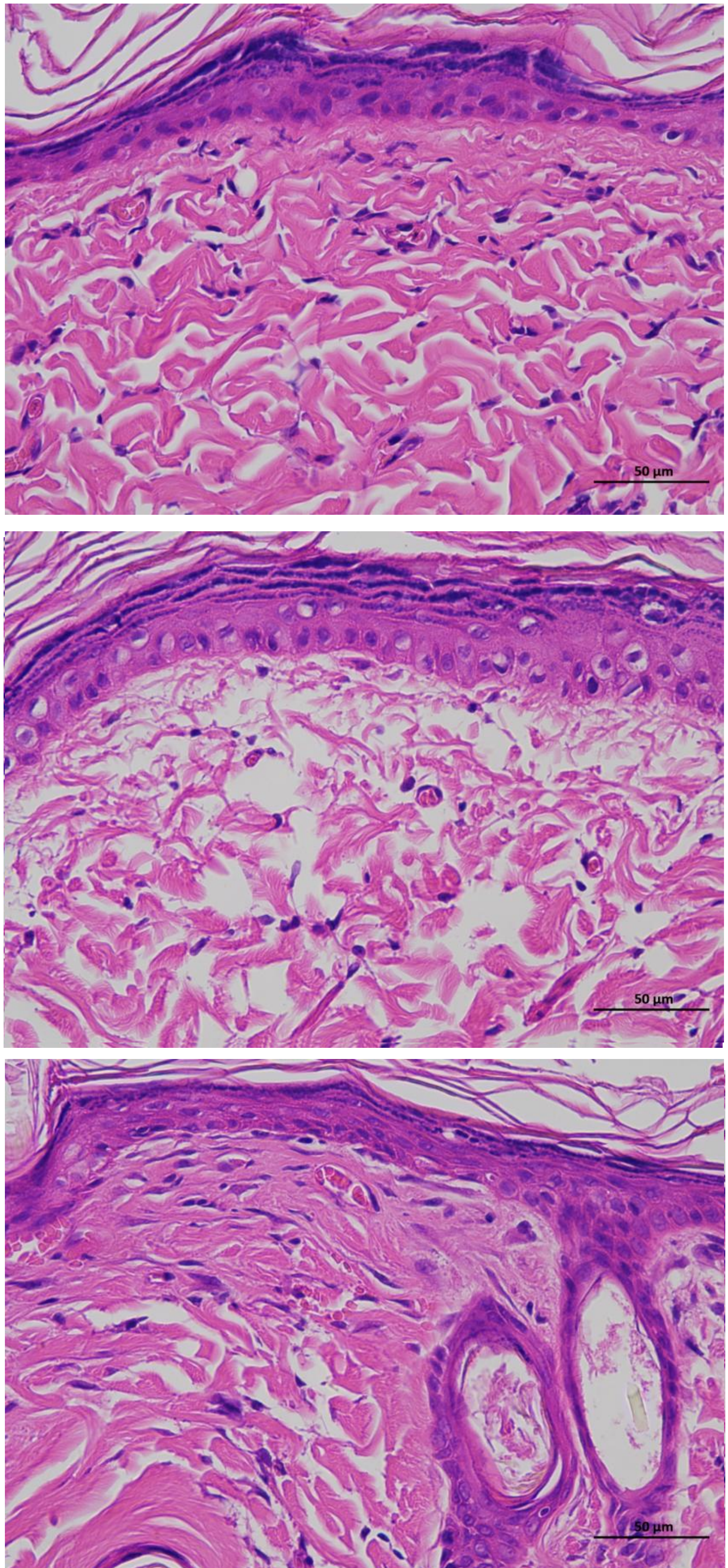

5 NControl 14day 400-1

5 NControl 14day 400-2

5 NControl 14day 400-3

Animal 6

Supplementary Figure 3A | Representative H&E staining of skin tissue of control animals / Day 14.

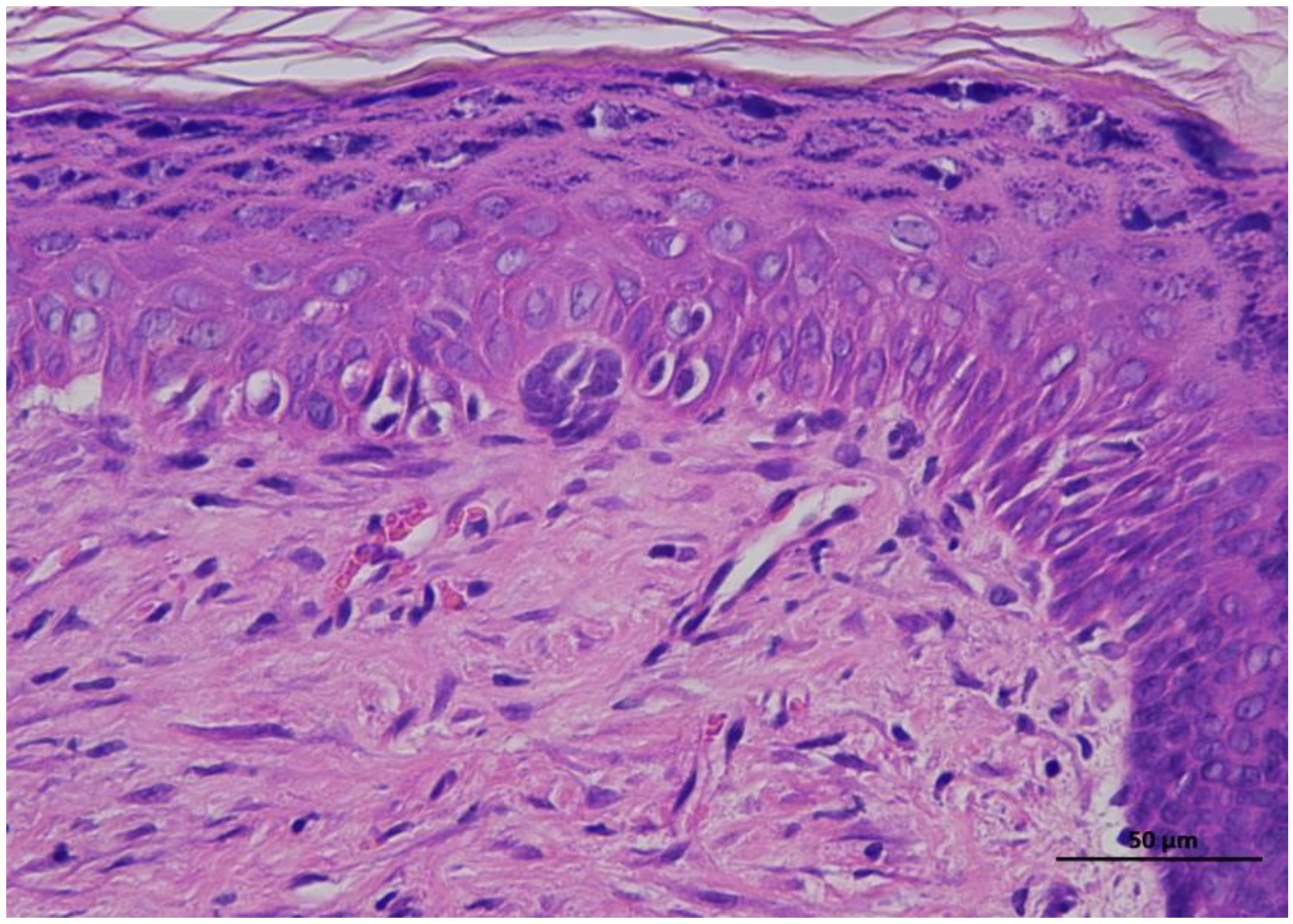

6 NControl 14day 400-1

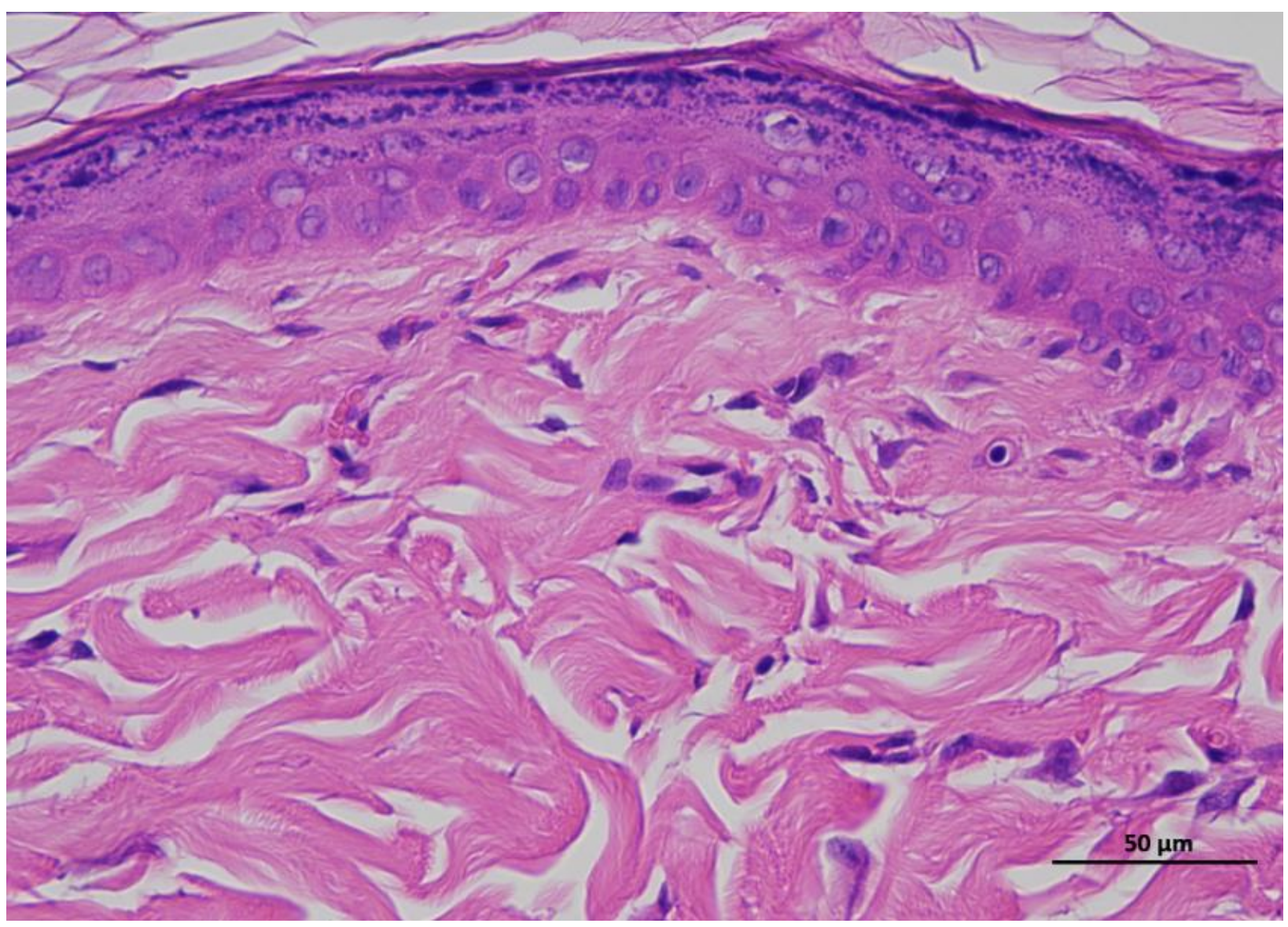

6 NControl 14day 400-2

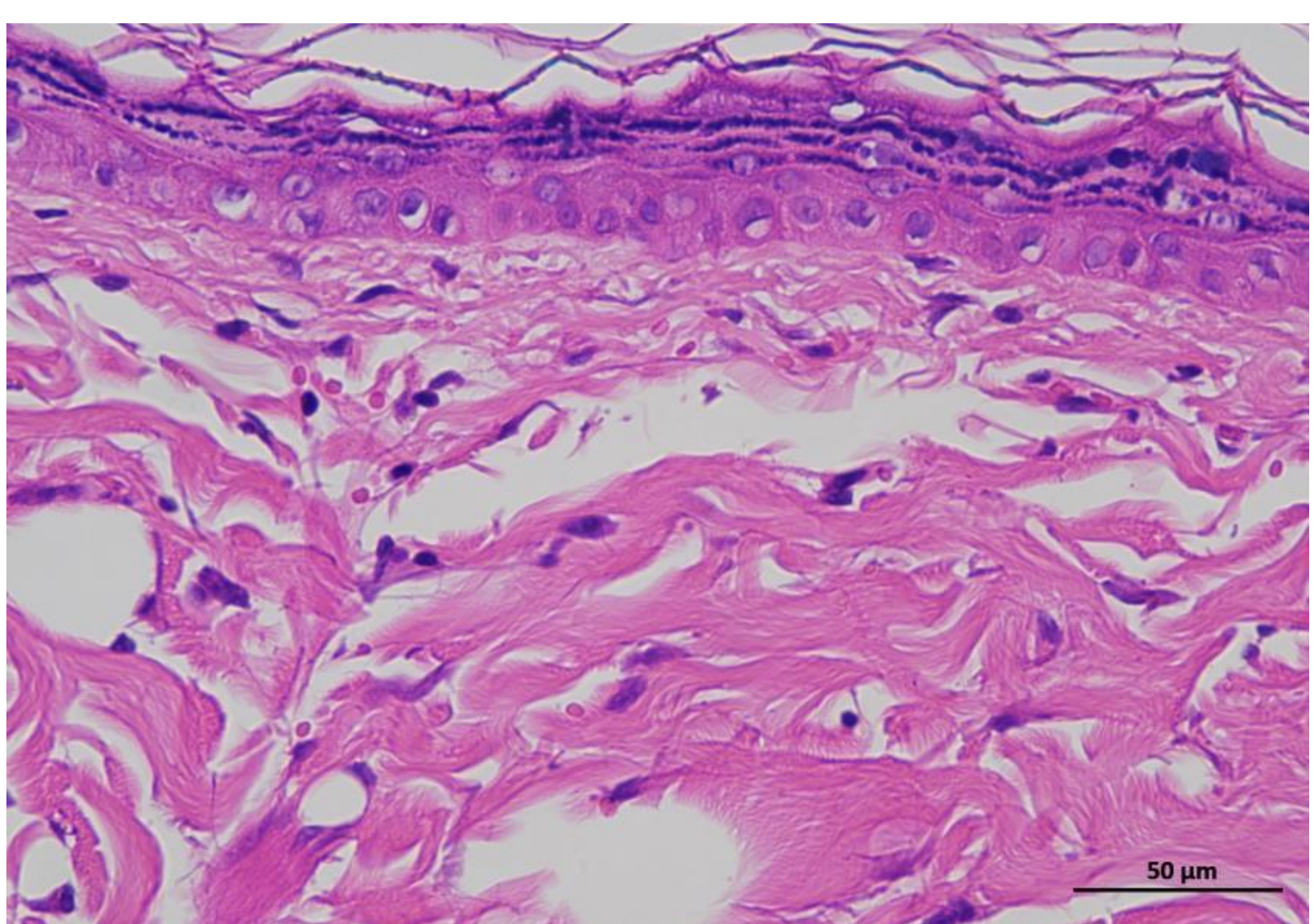

6 NControl 14day 400-3

Animal 1

Supplementary Figure 2b | Representative H&E staining of skin tissue of diabetic control animals / Day 0. Representative hematoxylin and eosin (H&E)–stained section of skin tissue collected from experimental rat. The image shows the typical architecture of the skin, including the epidermal layer with stratified keratinized epithelium and the underlying dermis composed of dense collagen fibers and connective tissue structures.
(n = 6 per group).

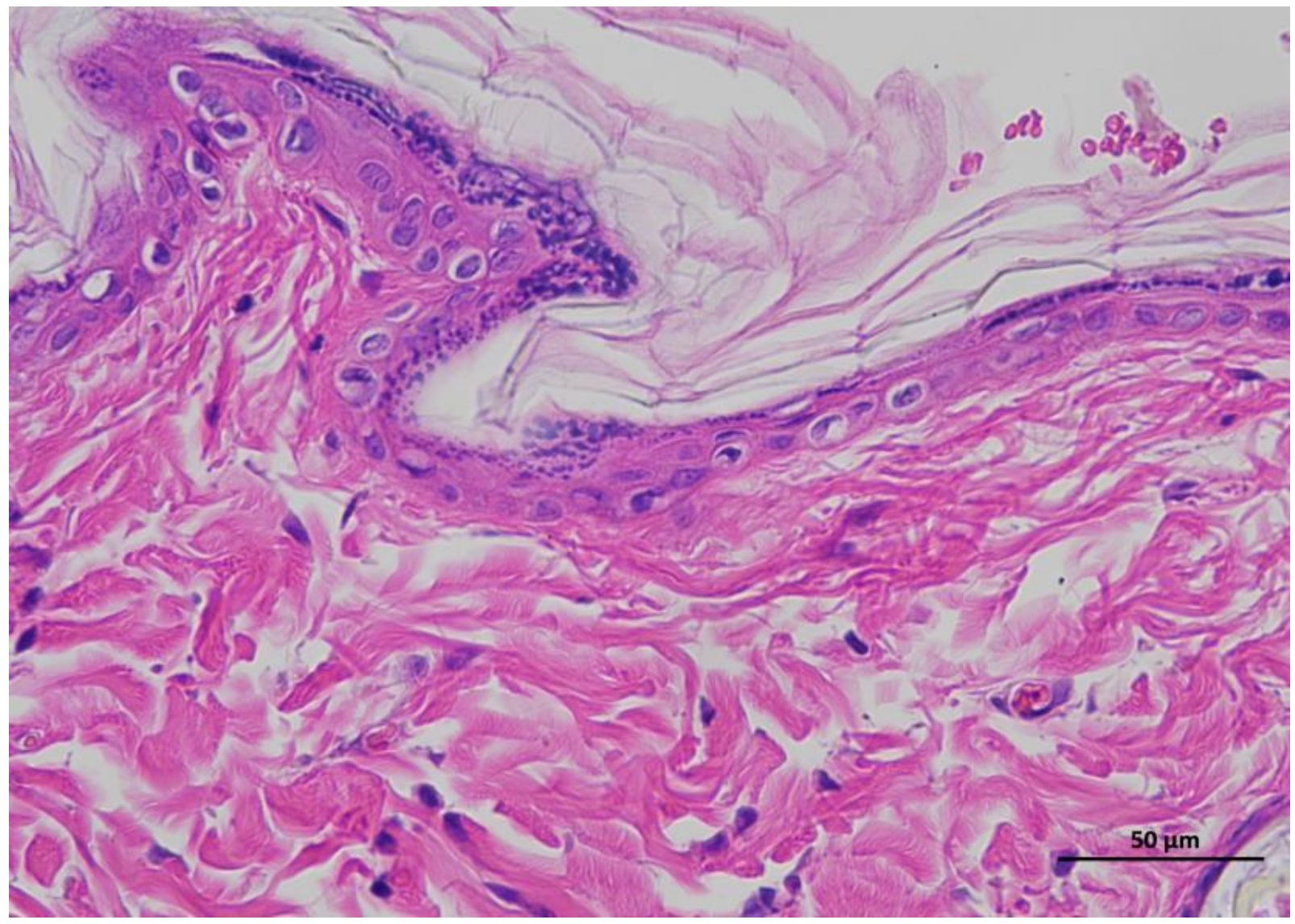

1 DControl 0day 400-1

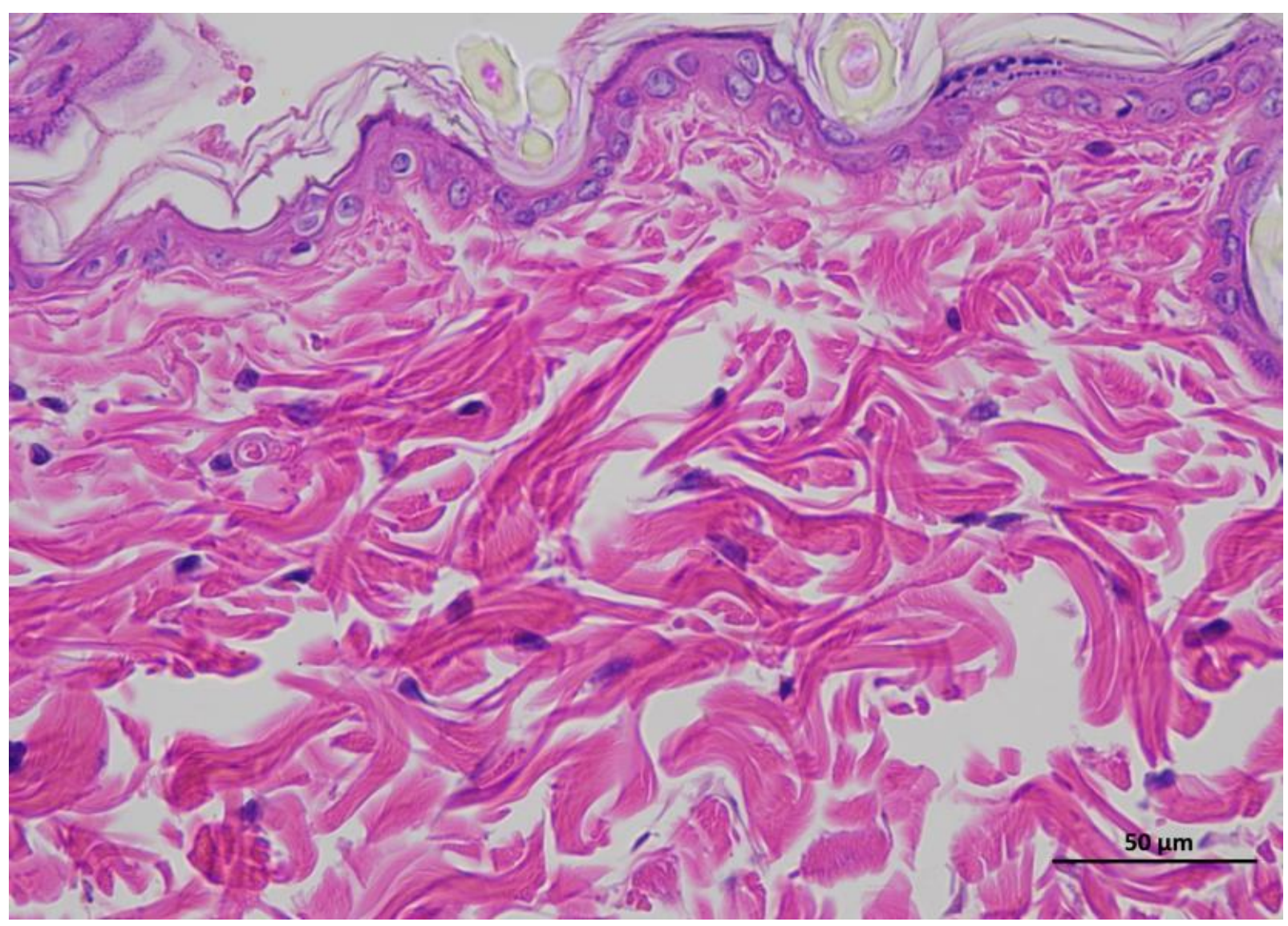

1 DControl 0day 400-2

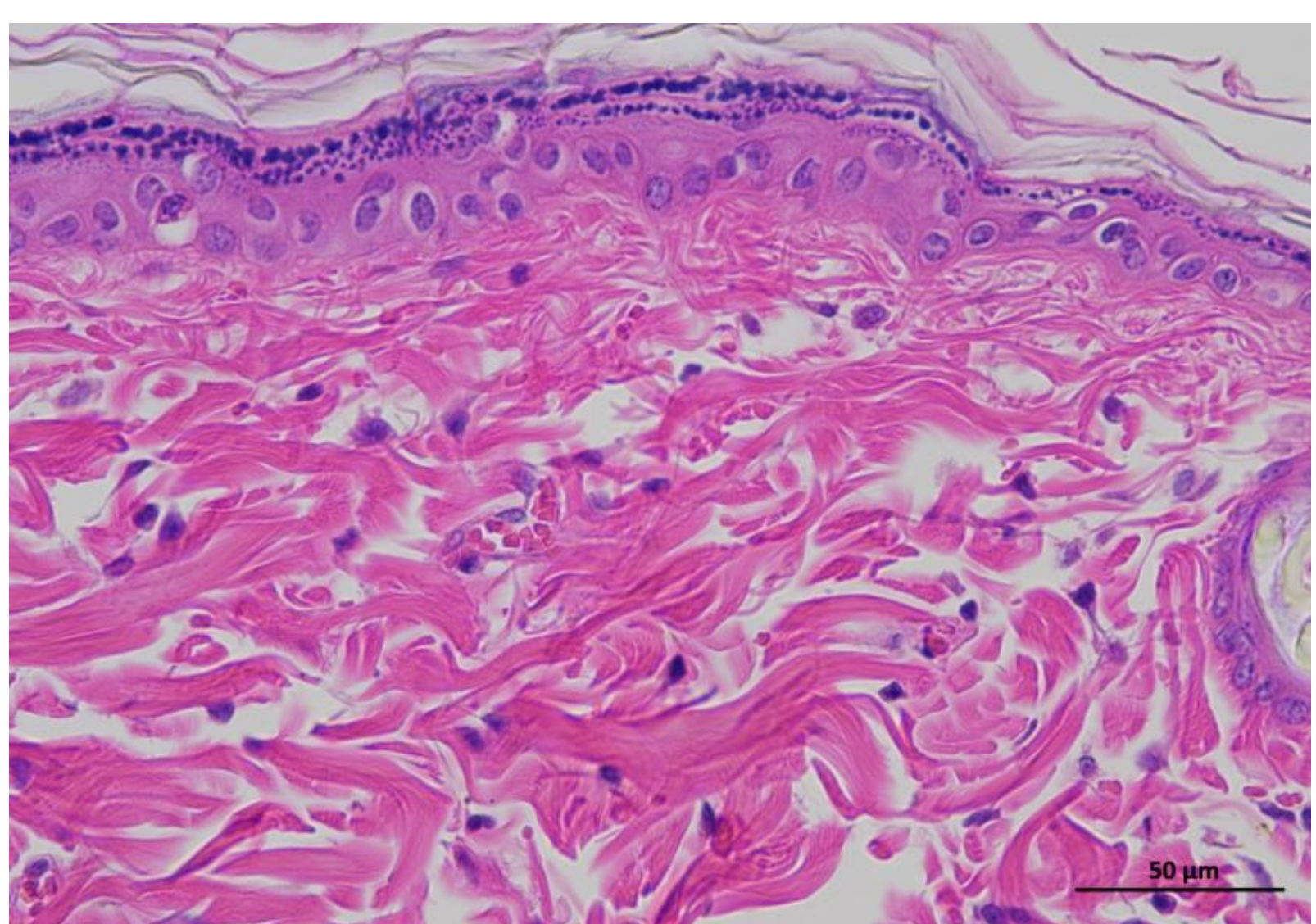

1 DControl 0day 400-3

Animal 2

Supplementary Figure 2b | Representative H&E staining of skin tissue of diabetic control animals / Day 0.

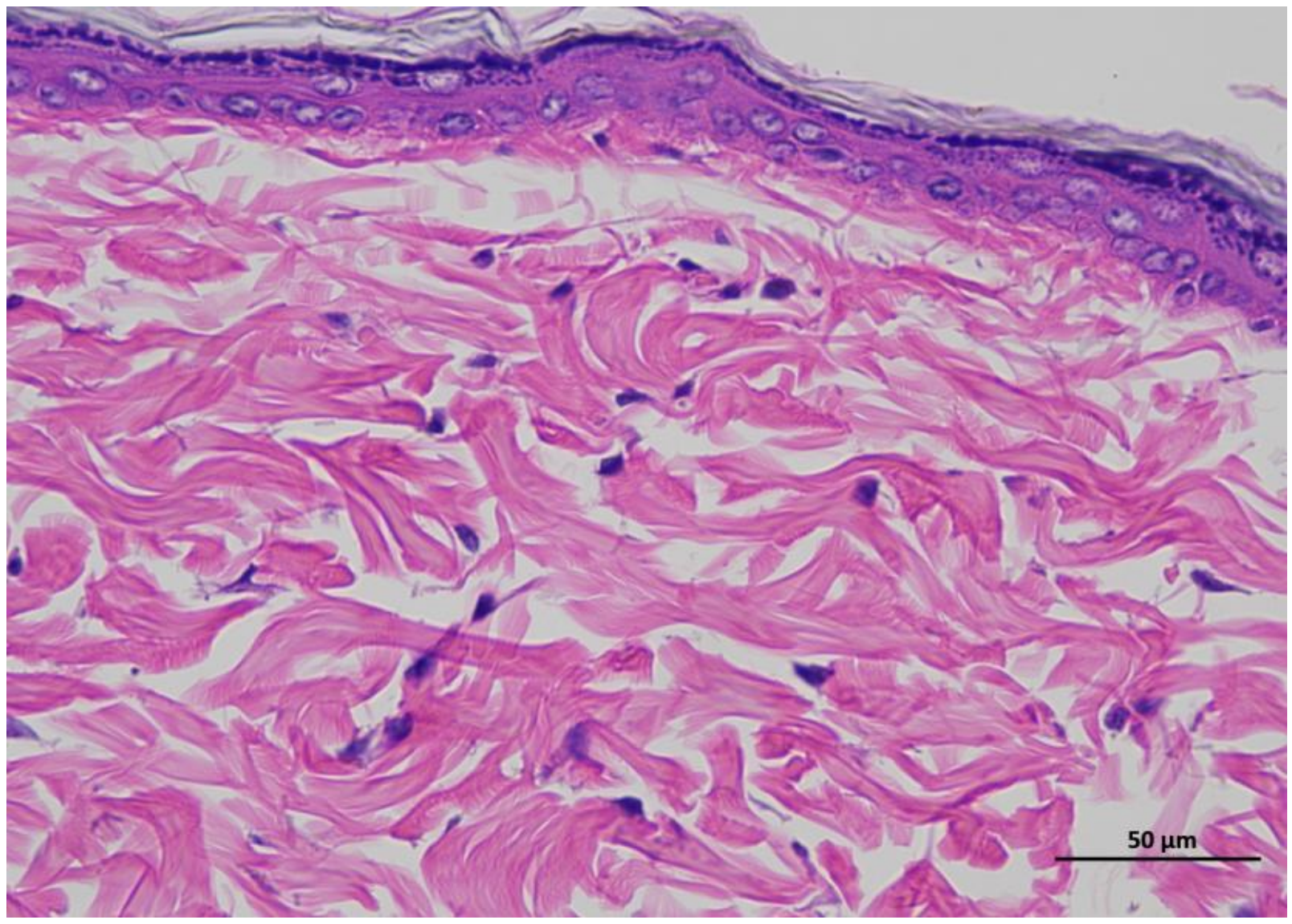

2 DControl 0day 400-1

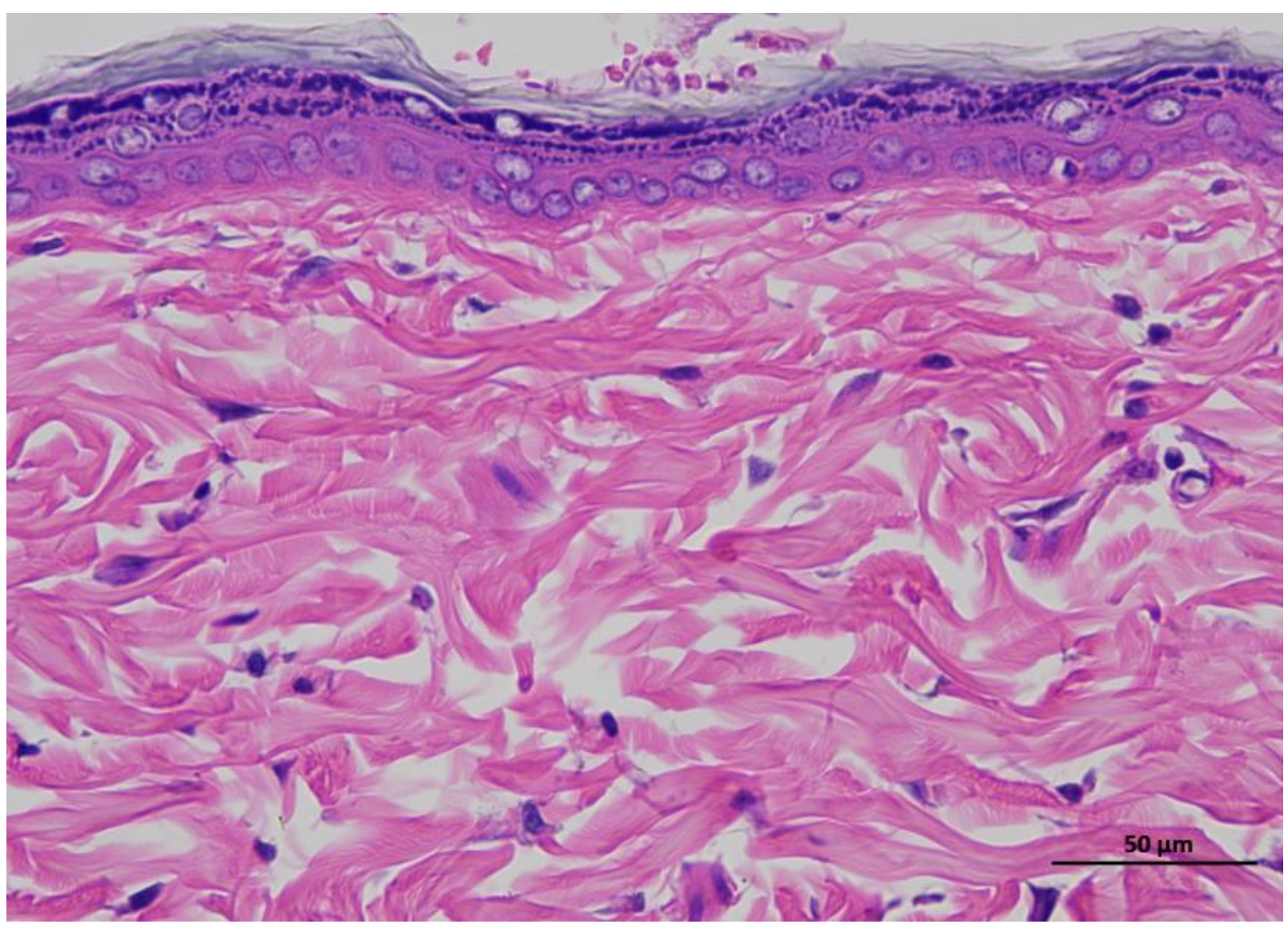

2 DControl 0day 400-2

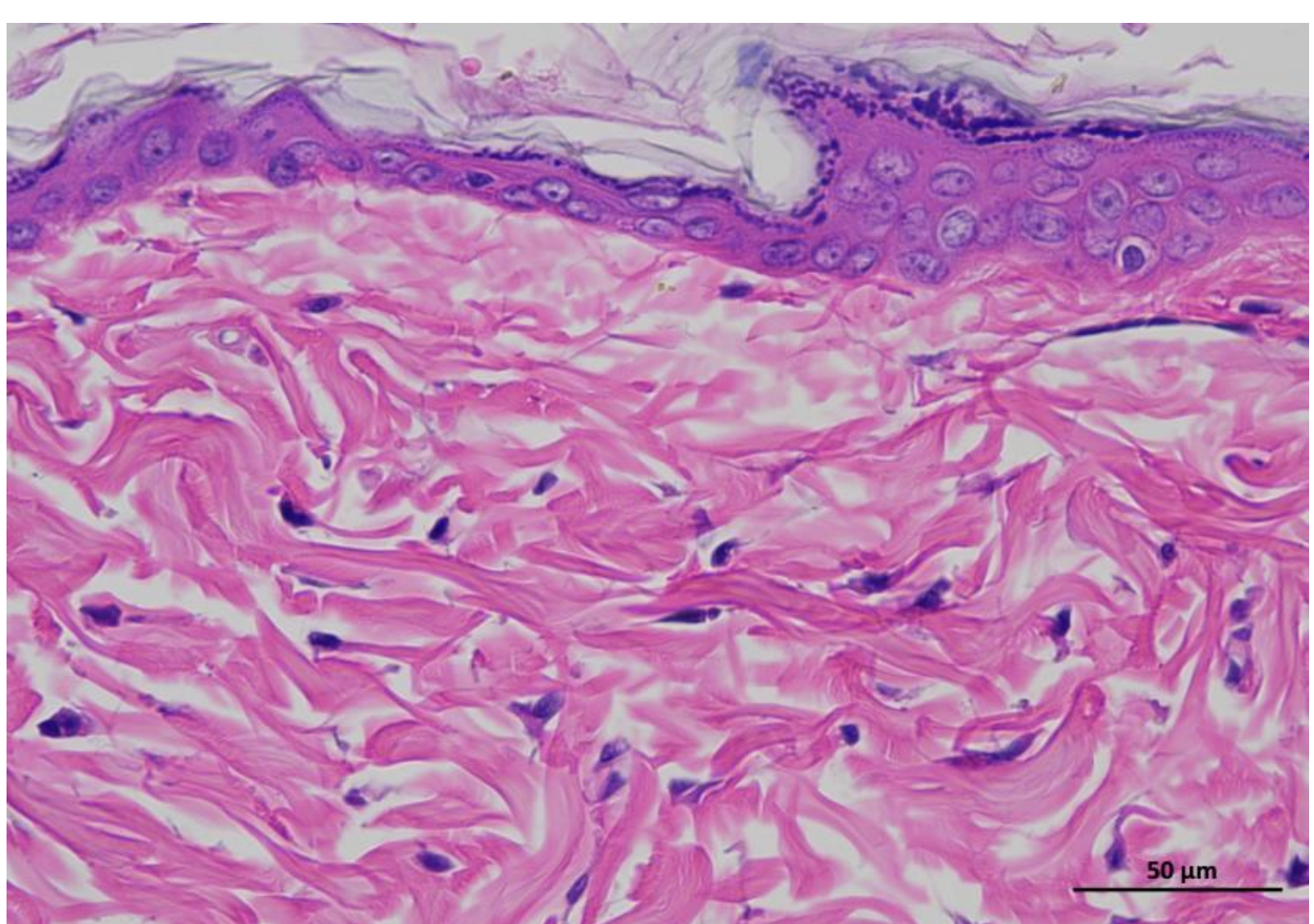

2 DControl 0day 400-3

Animal 3

Supplementary Figure 2b | Representative H&E staining of skin tissue of diabetic control animals / Day 0.

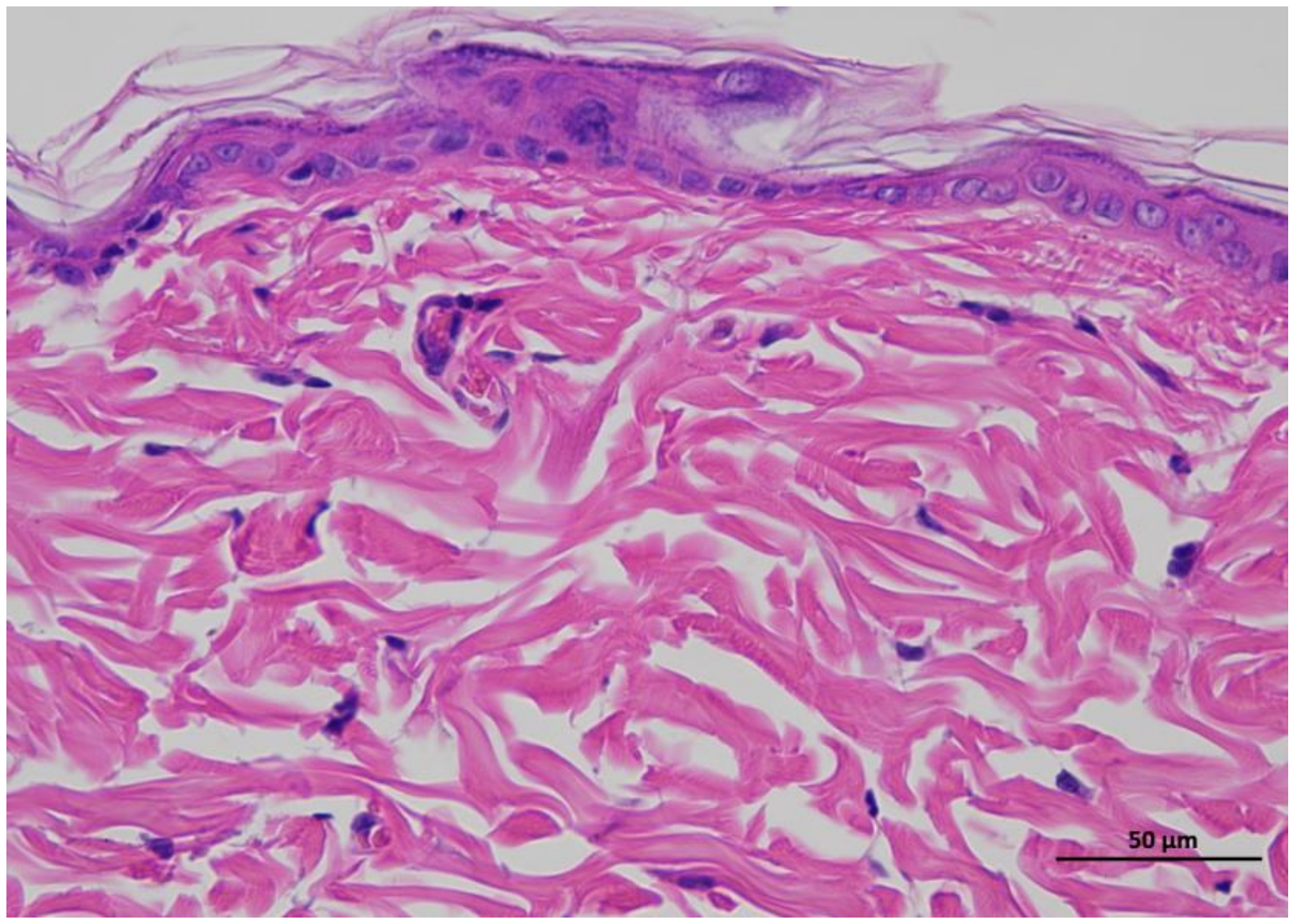

3 DControl 0day 400-1

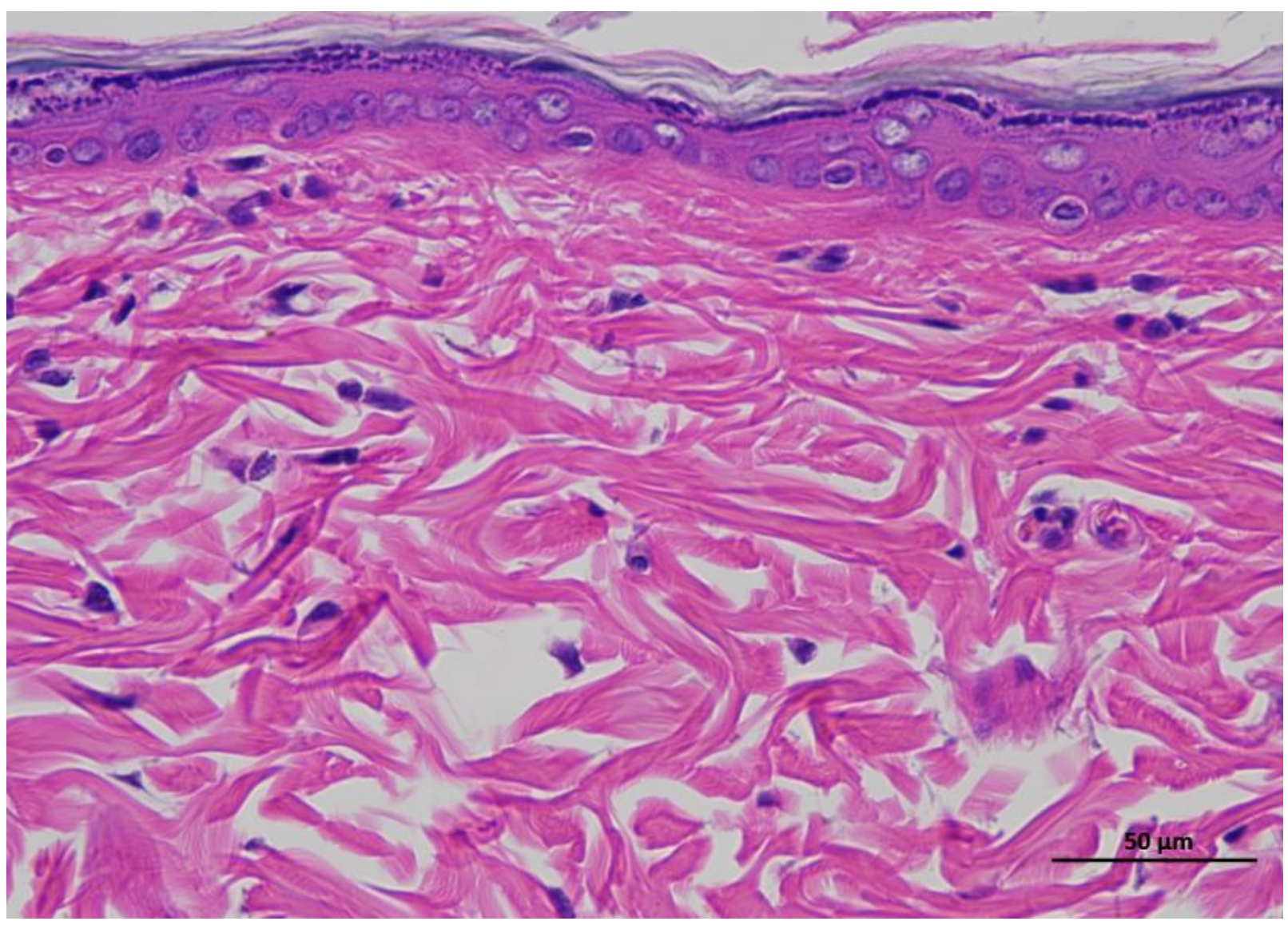

3 DControl 0day 400-2

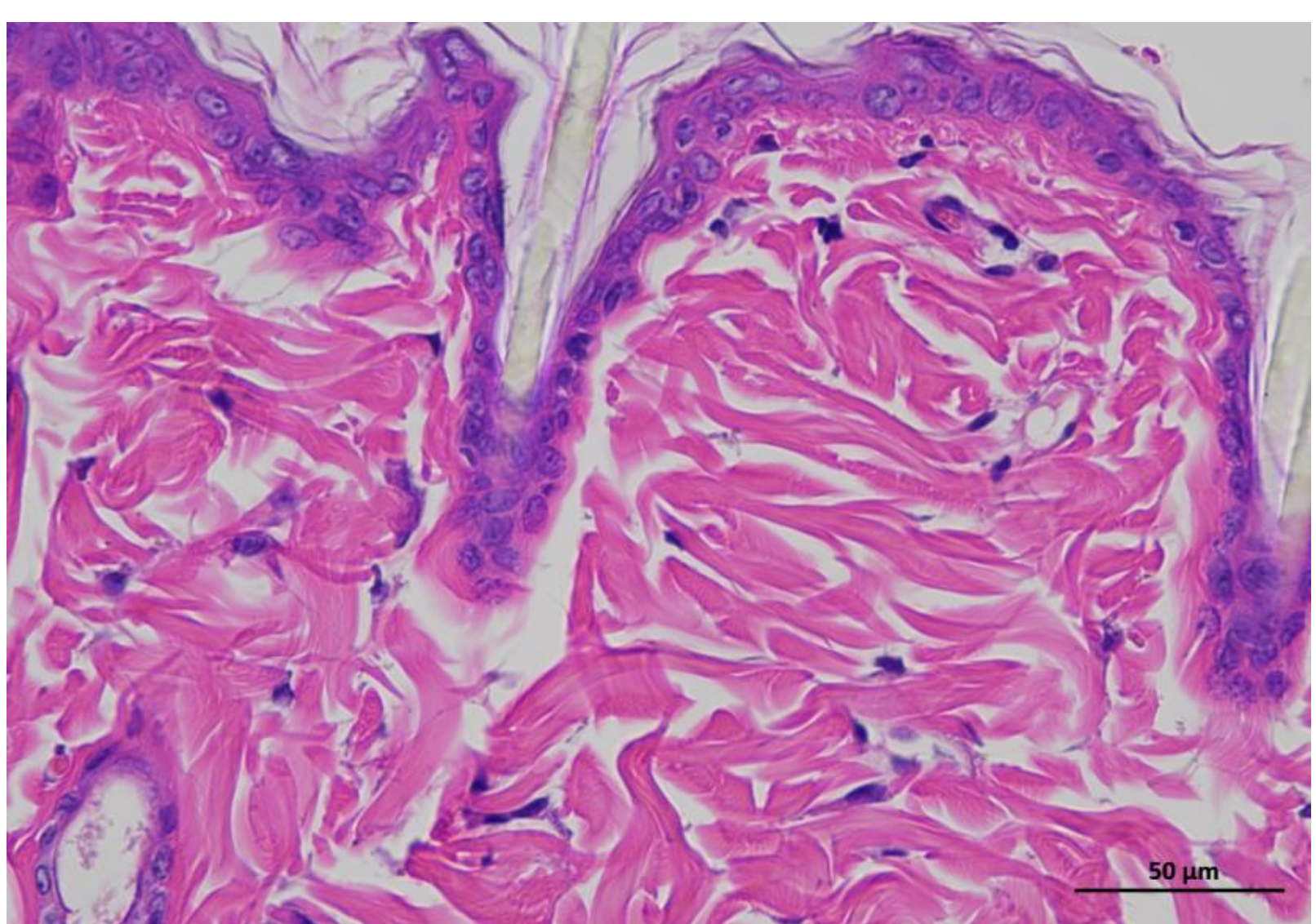

3 DControl 0day 400-3

Animal 4

Supplementary Figure 2b | Representative H&E staining of skin tissue of diabetic control animals / Day 0.

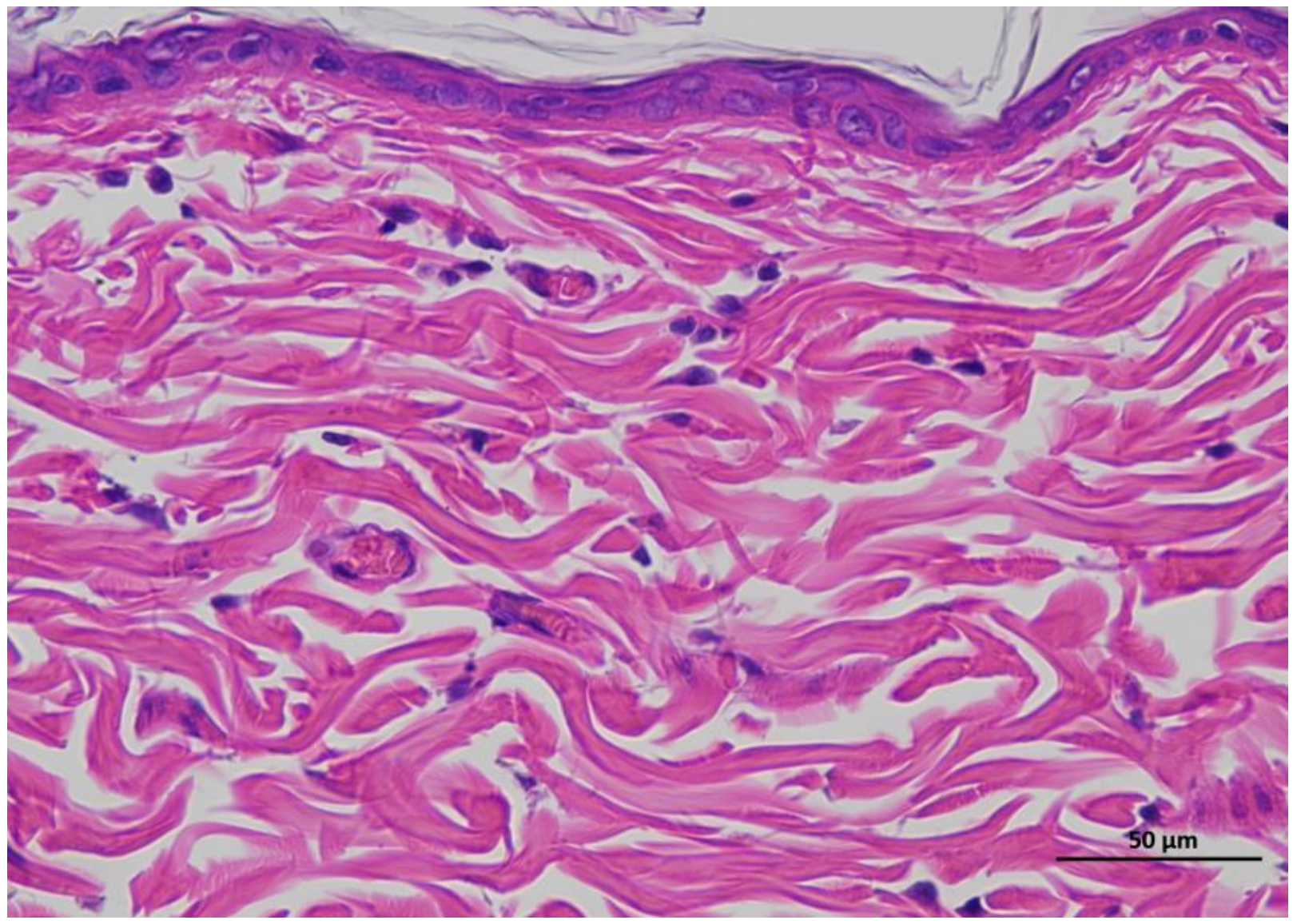

4 DControl 0day 400-1

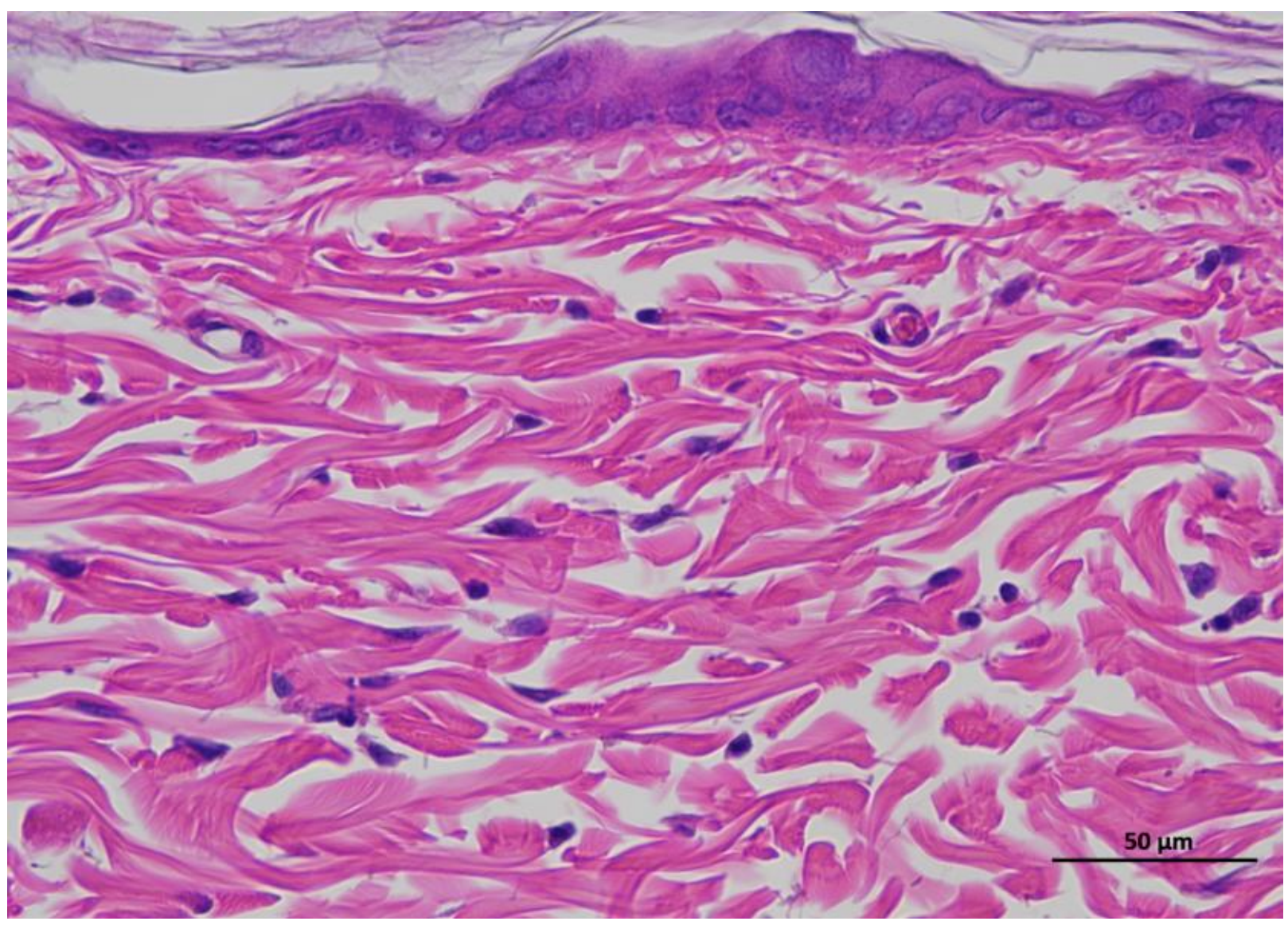

4 DControl 0day 400-2

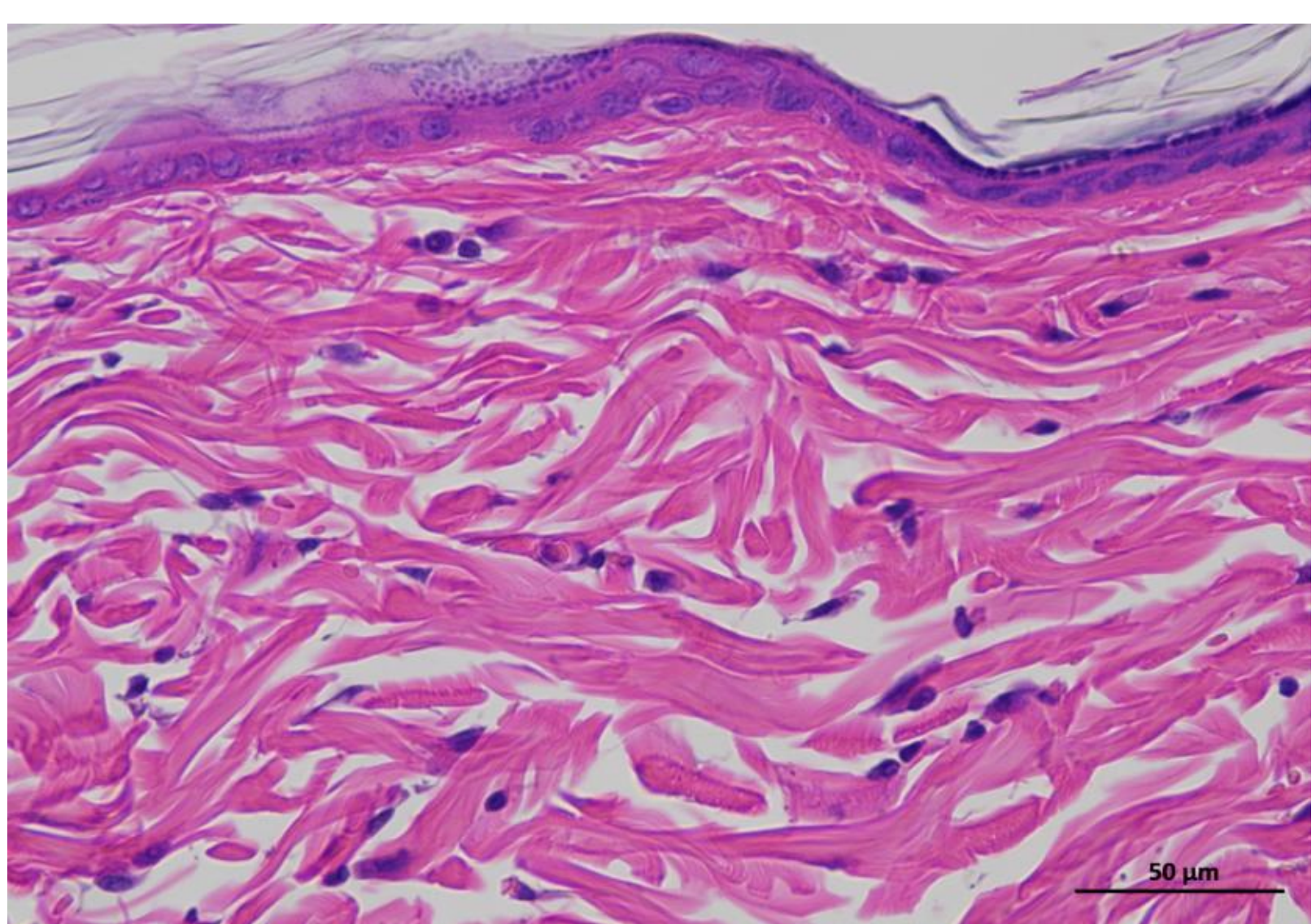

4 DControl 0day 400-3

Animal 5

Supplementary Figure 2b | Representative H&E staining of skin tissue of diabetic control animals / Day 0.

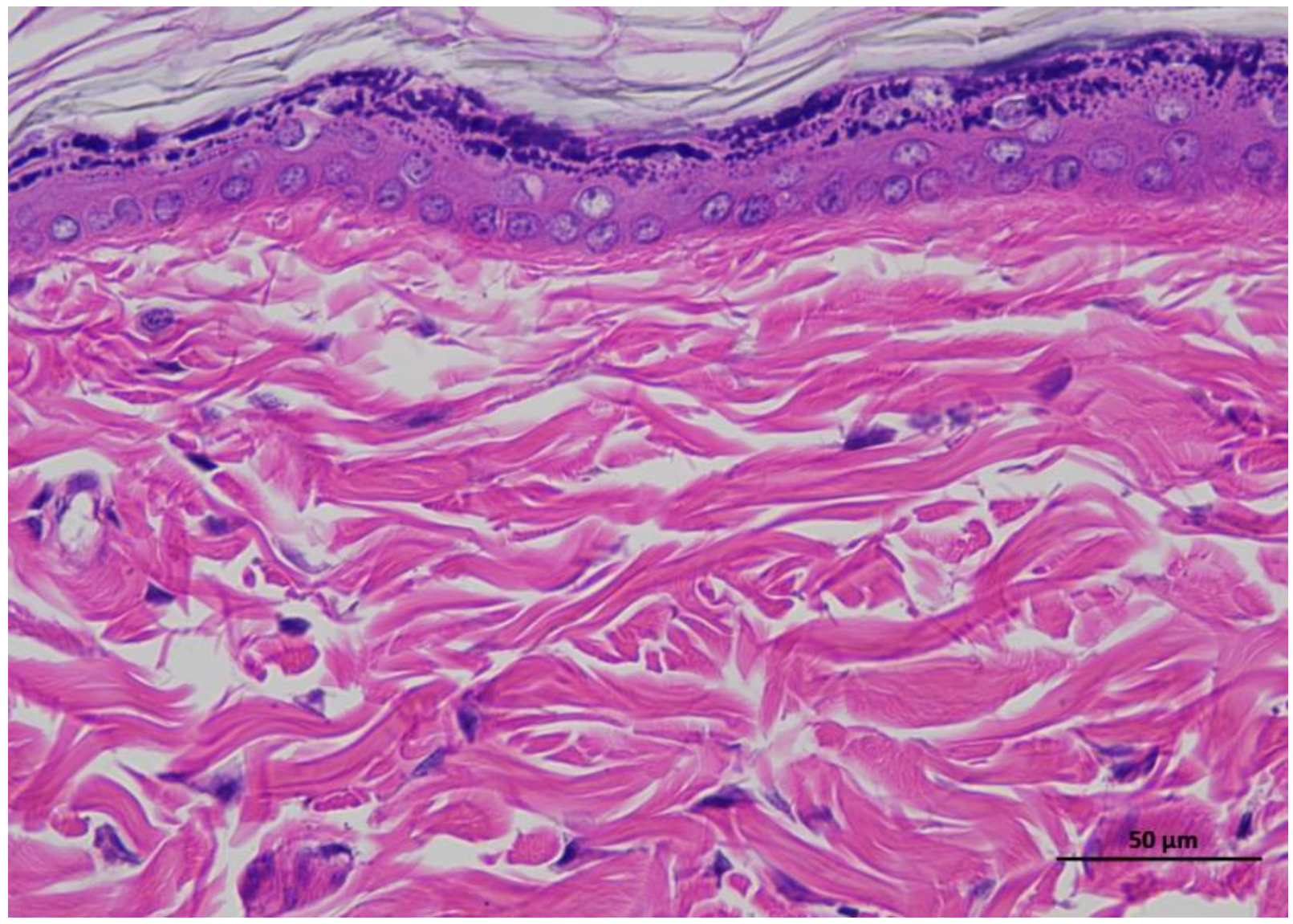

5 DControl 0day 400-1

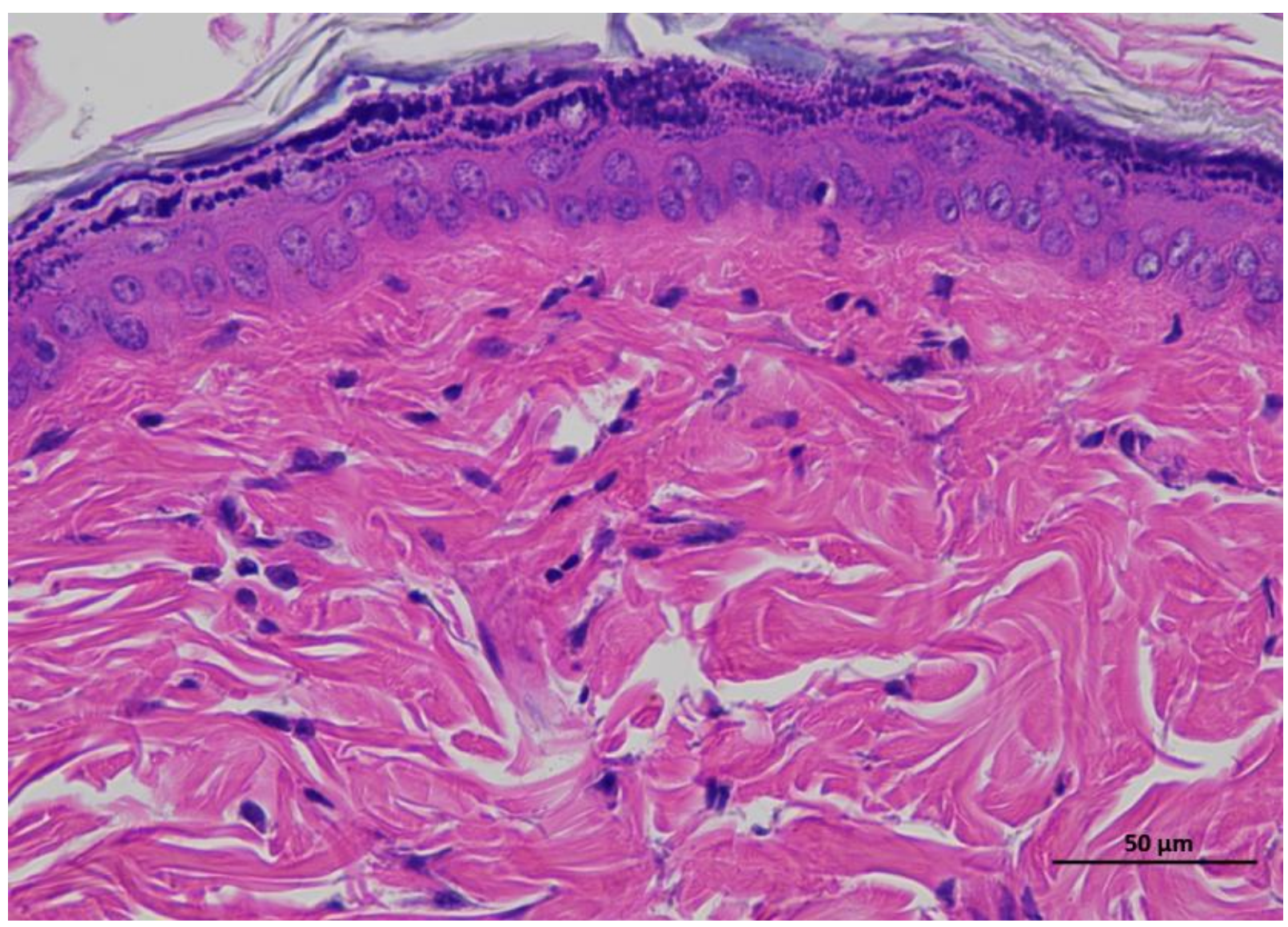

5 DControl 0day 400-2

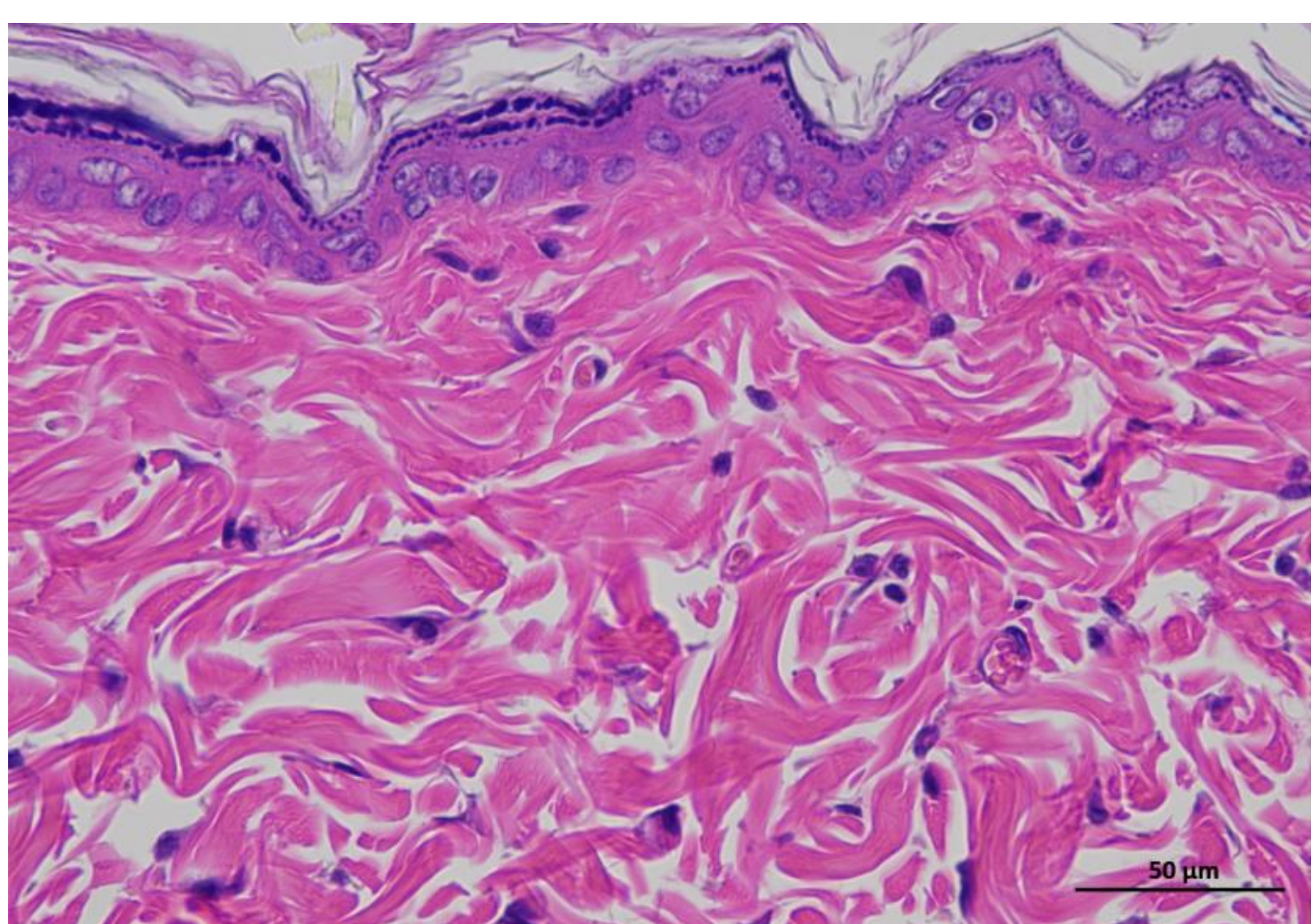

5 DControl 0day 400-3

Animal 6

Supplementary Figure 2b | Representative H&E staining of skin tissue of diabetic control animals / Day 0.

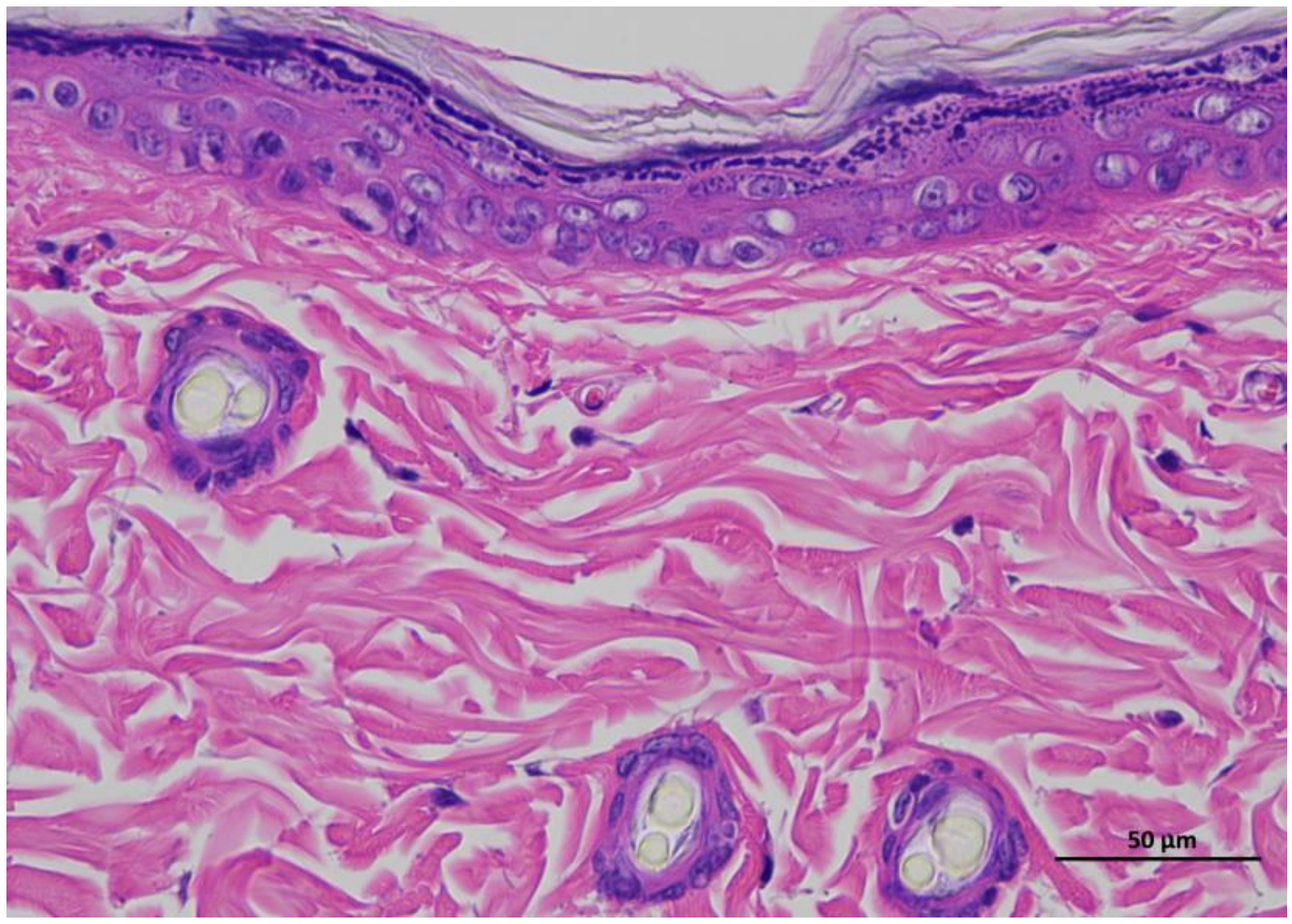

6 DControl 0day 400-1

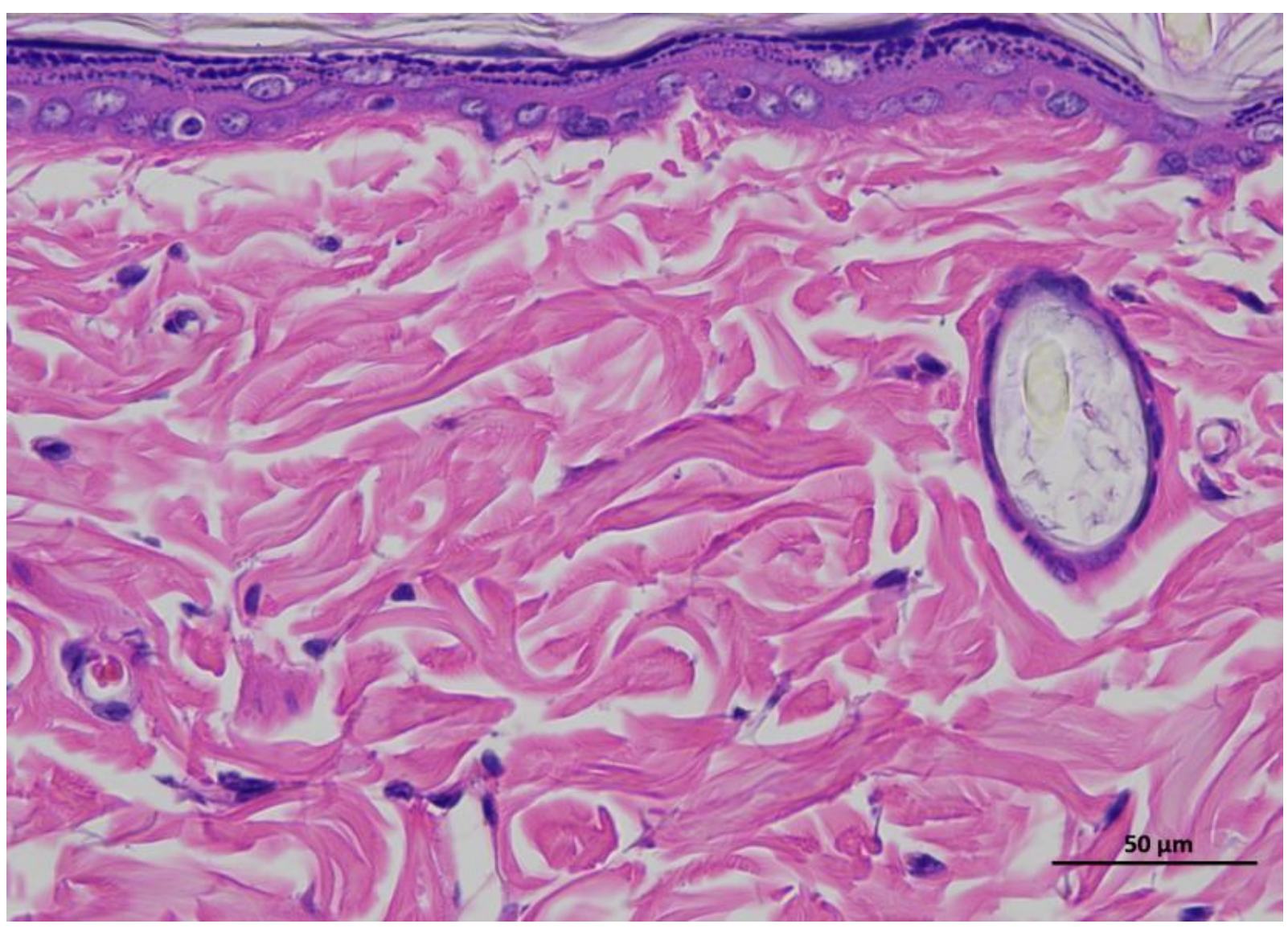

6 DControl 0day 400-2

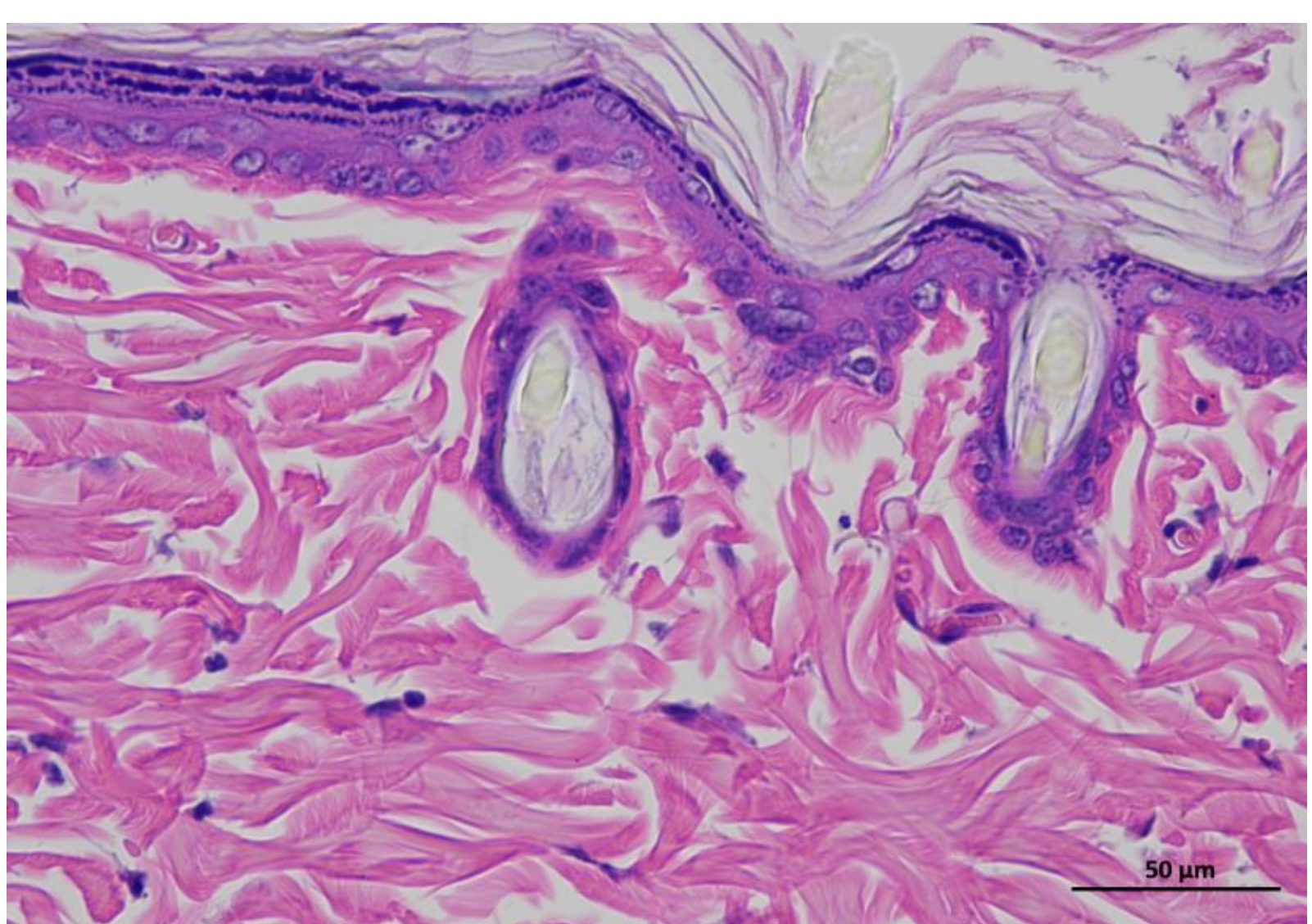

6 DControl 0day 400-3

Supplementary Figure 3b | Representative H&E staining of skin tissue of diabetic control animals / Day 14. Representative hematoxylin and eosin (H&E)–stained section of skin tissue collected from experimental rat. The image shows the typical architecture of the skin, including the epidermal layer with stratified keratinized epithelium and the underlying dermis composed of dense collagen fibers and connective tissue structures.
(n = 6 per group).

Animal 1

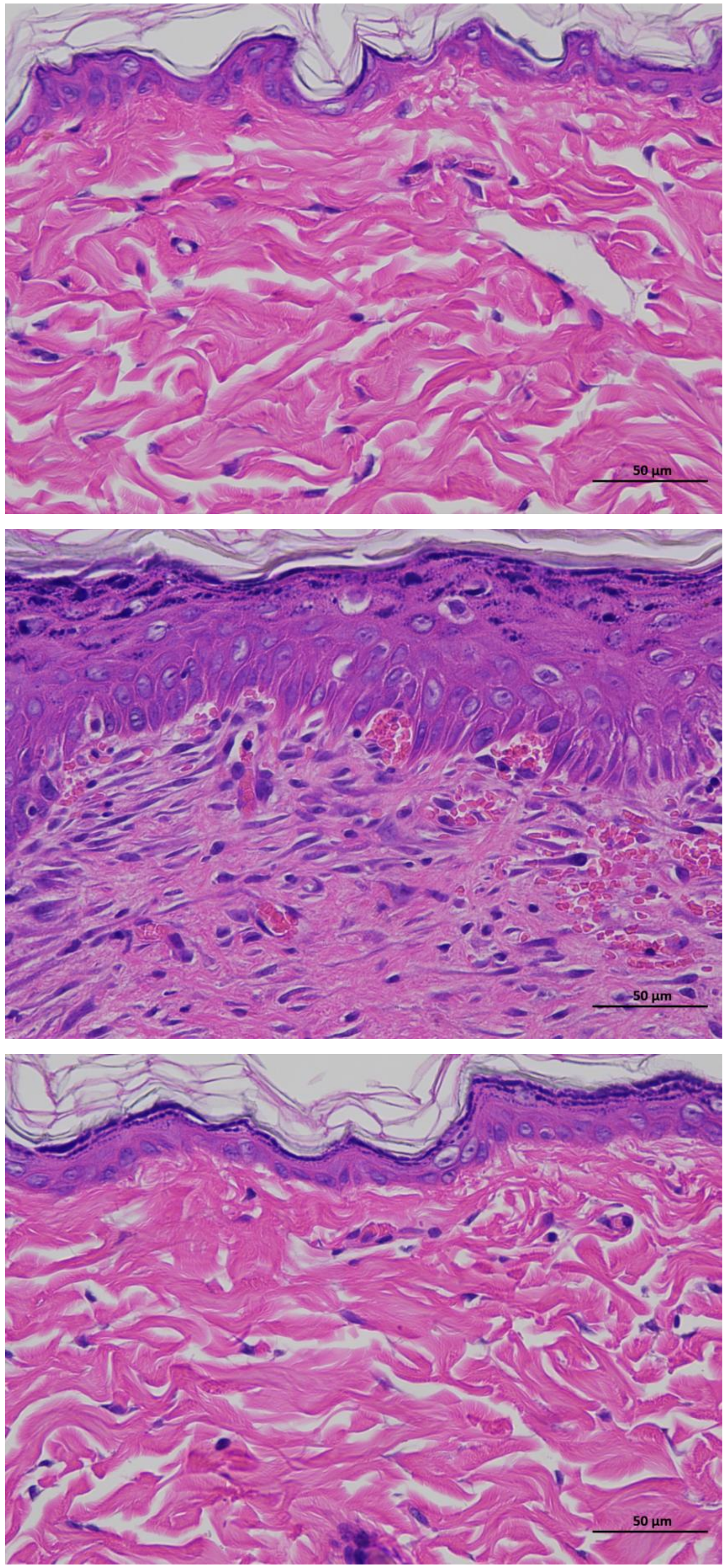

1 DControl 14day 400-1

1 DControl 14day 400-2

1 DControl 14day 400-3

Animal 2

Supplementary Figure 3b | Representative H&E staining of skin tissue of diabetic control animals / Day 14.

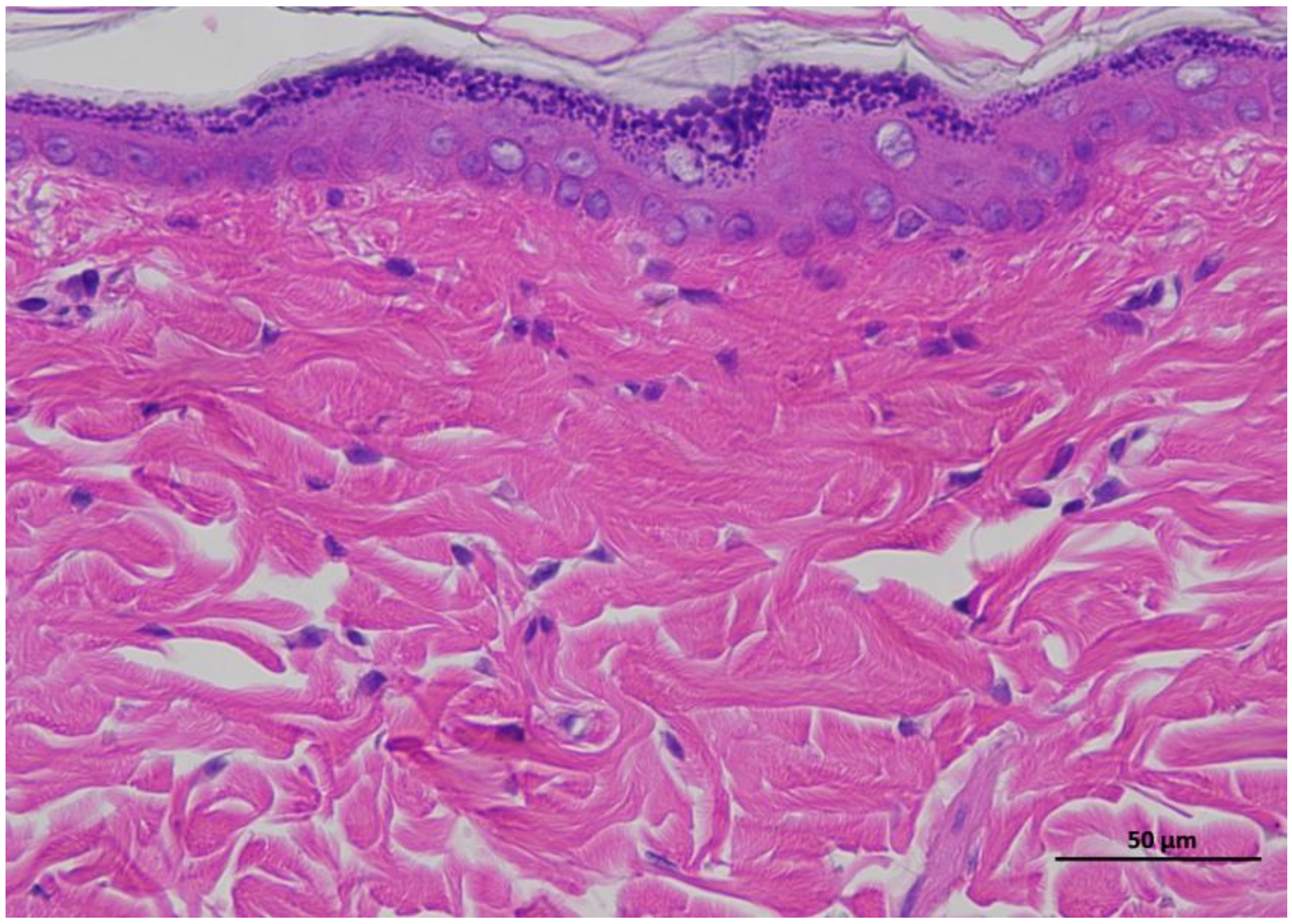

2 DControl 14day 400-1

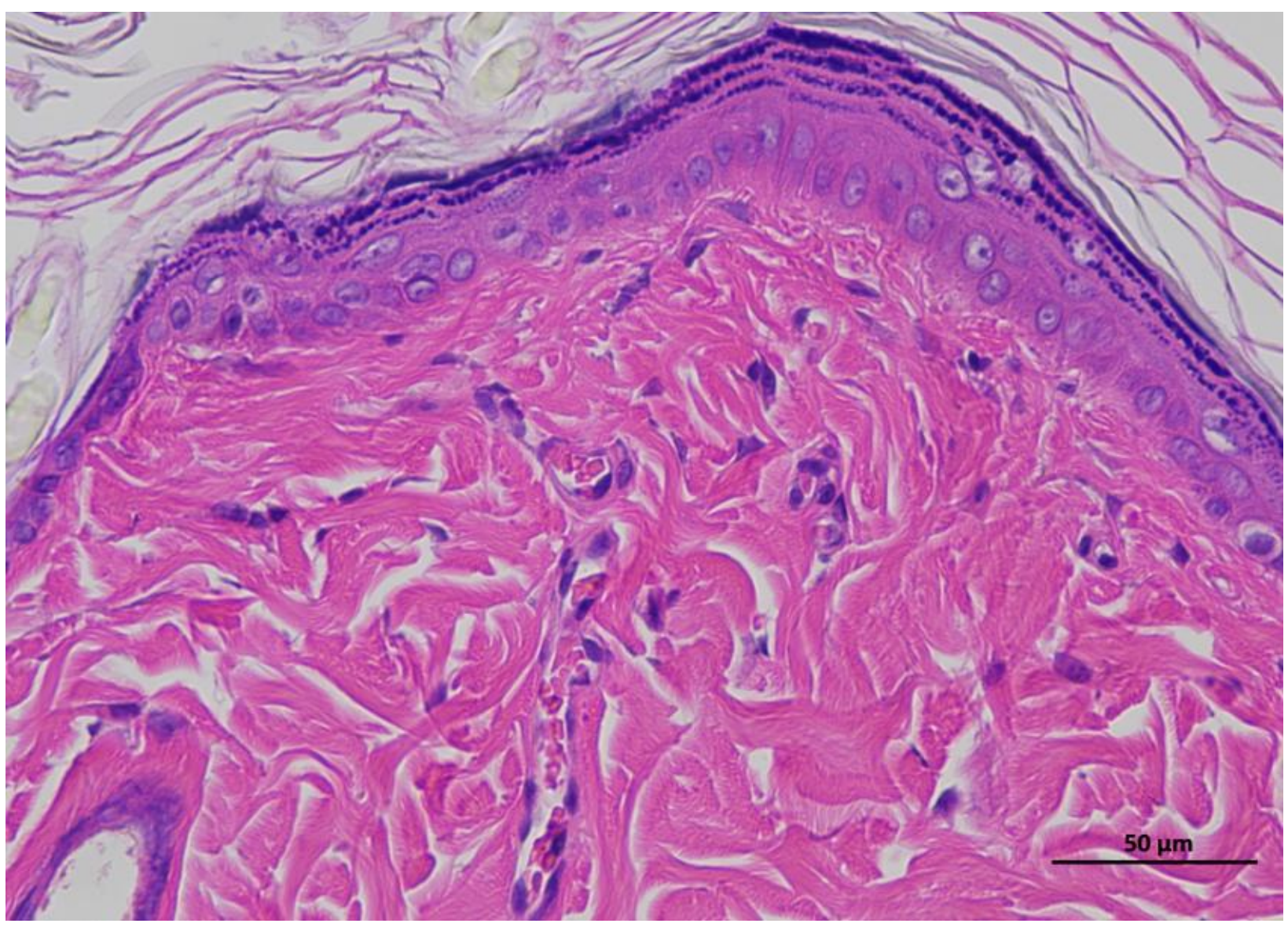

2 DControl 14day 400-2

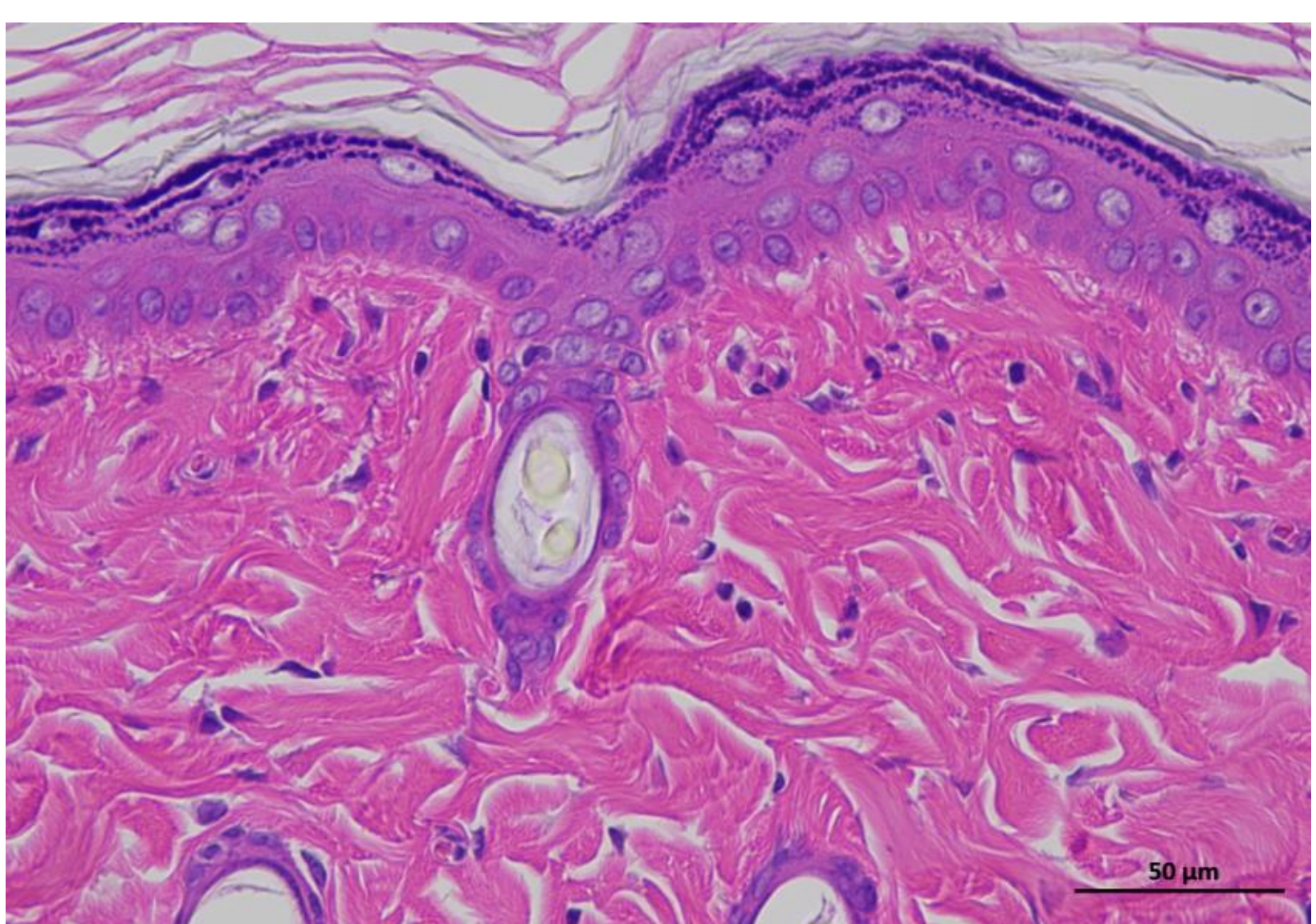

2 DControl 14day 400-3

Animal 3

Supplementary Figure 3b | Representative H&E staining of skin tissue of diabetic control animals / Day 14.

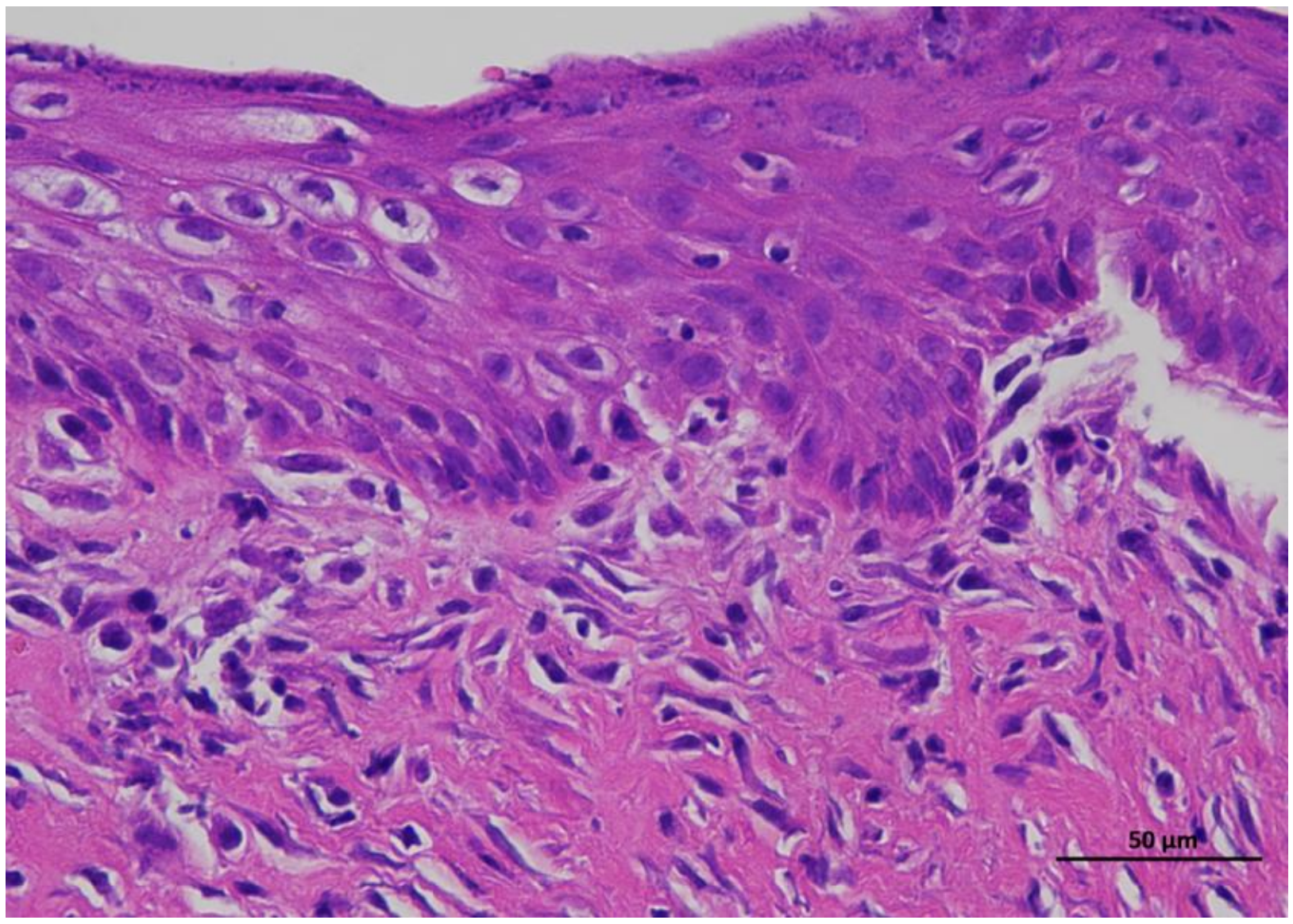

3 DControl 14day 400-1

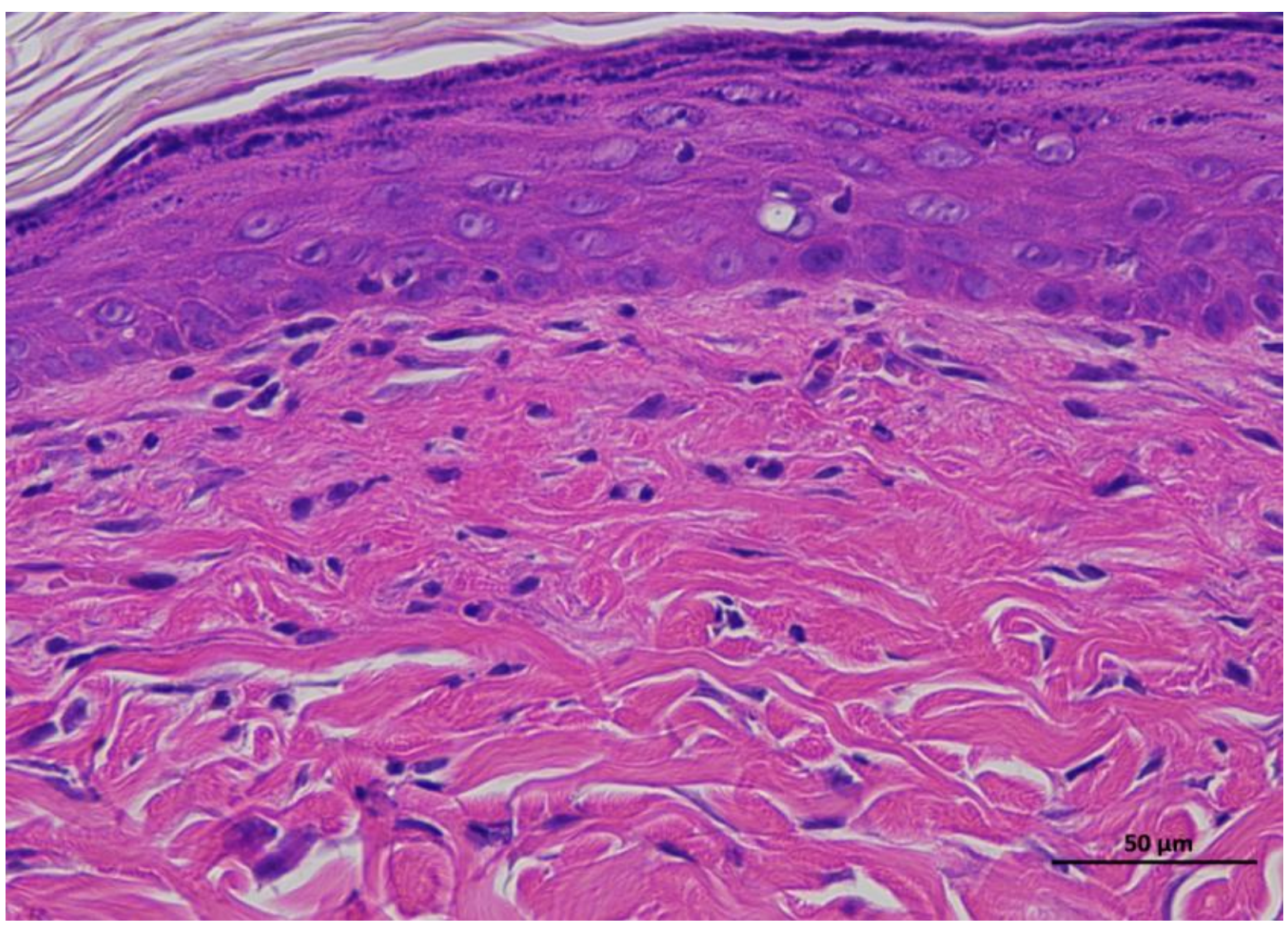

3 DControl 14day 400-2

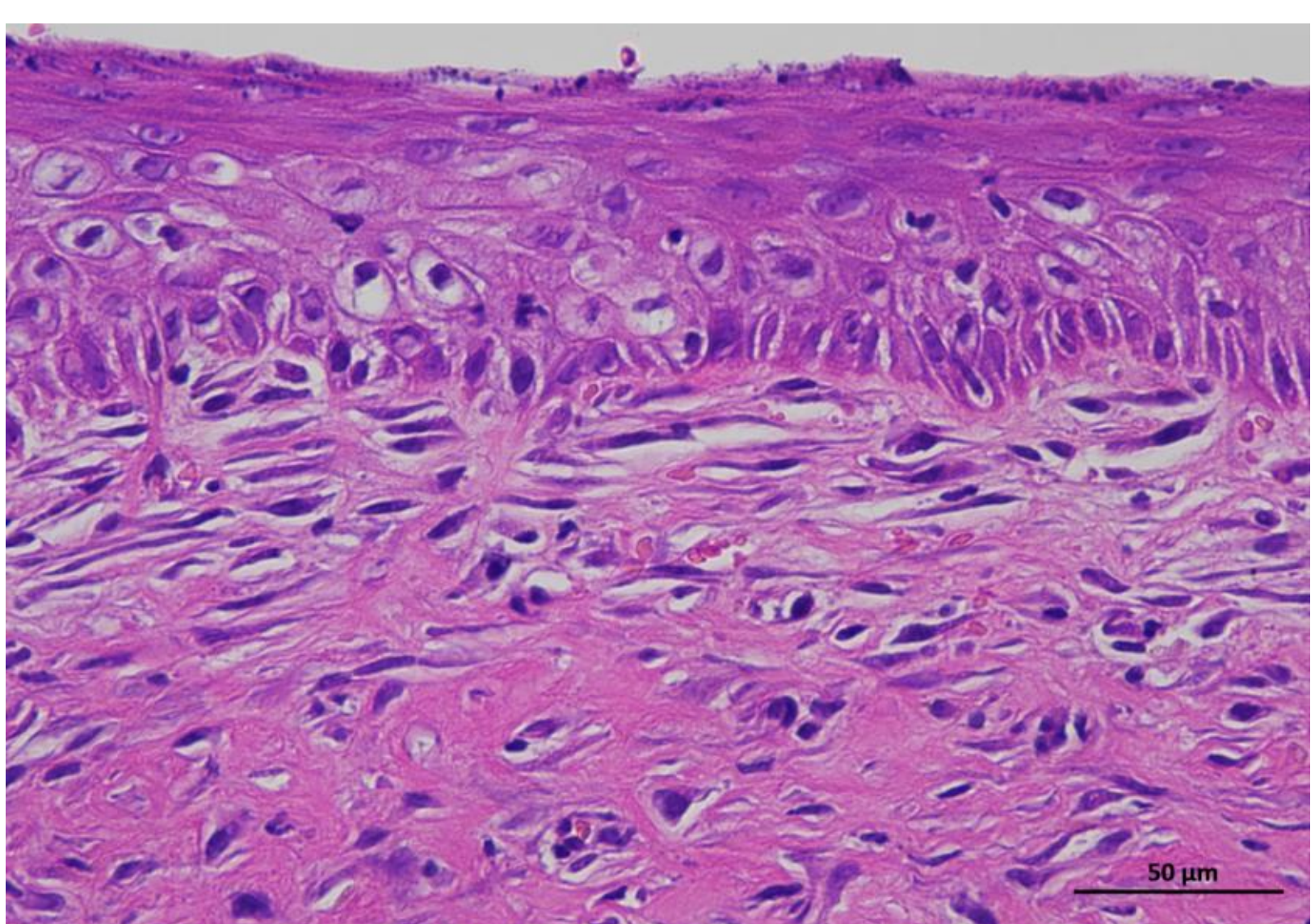

3 DControl 14day 400-3

Animal 5

Supplementary Figure 3b | Representative H&E staining of skin tissue of diabetic control animals / Day 14.

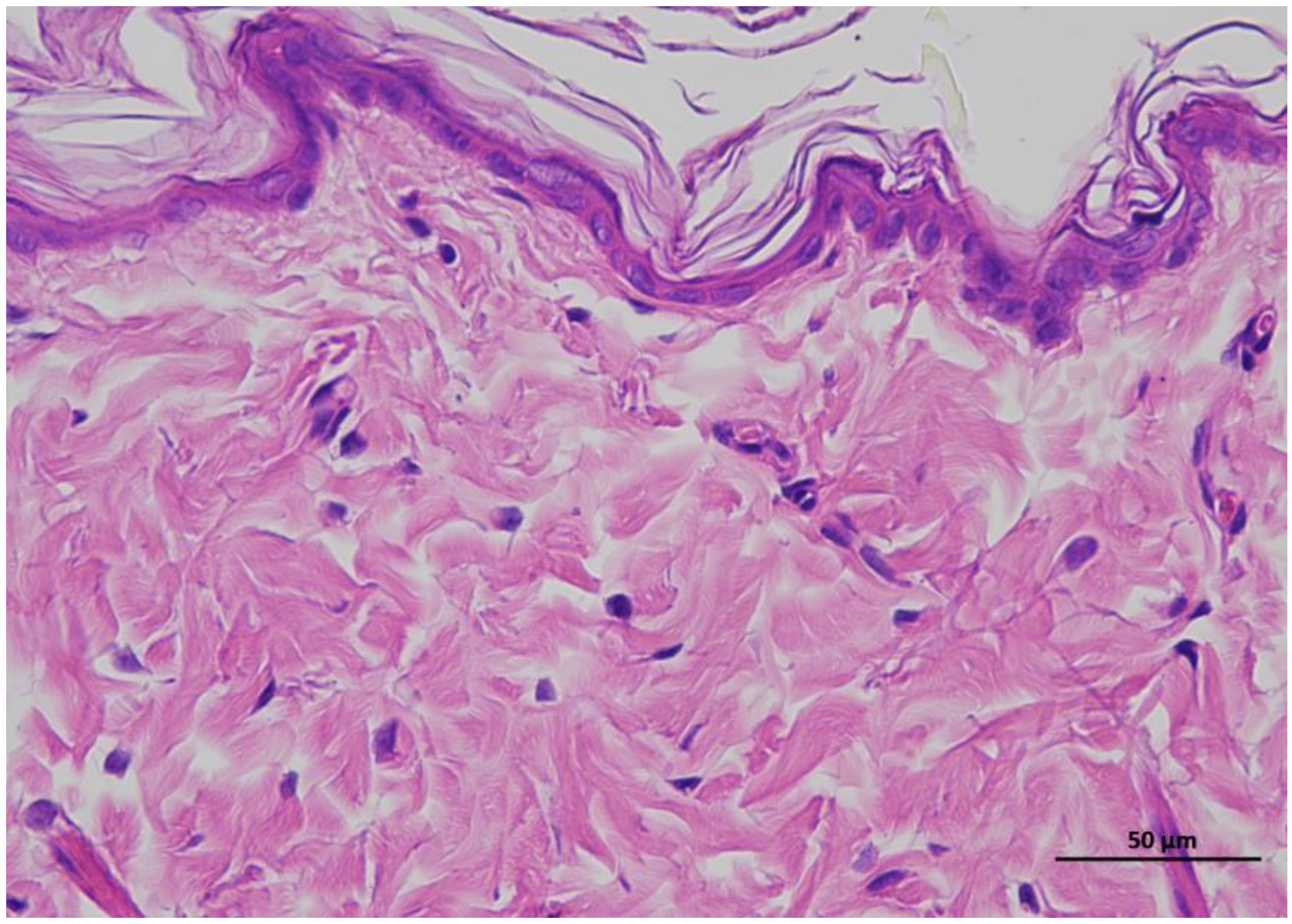

5 DControl 14day 400-1

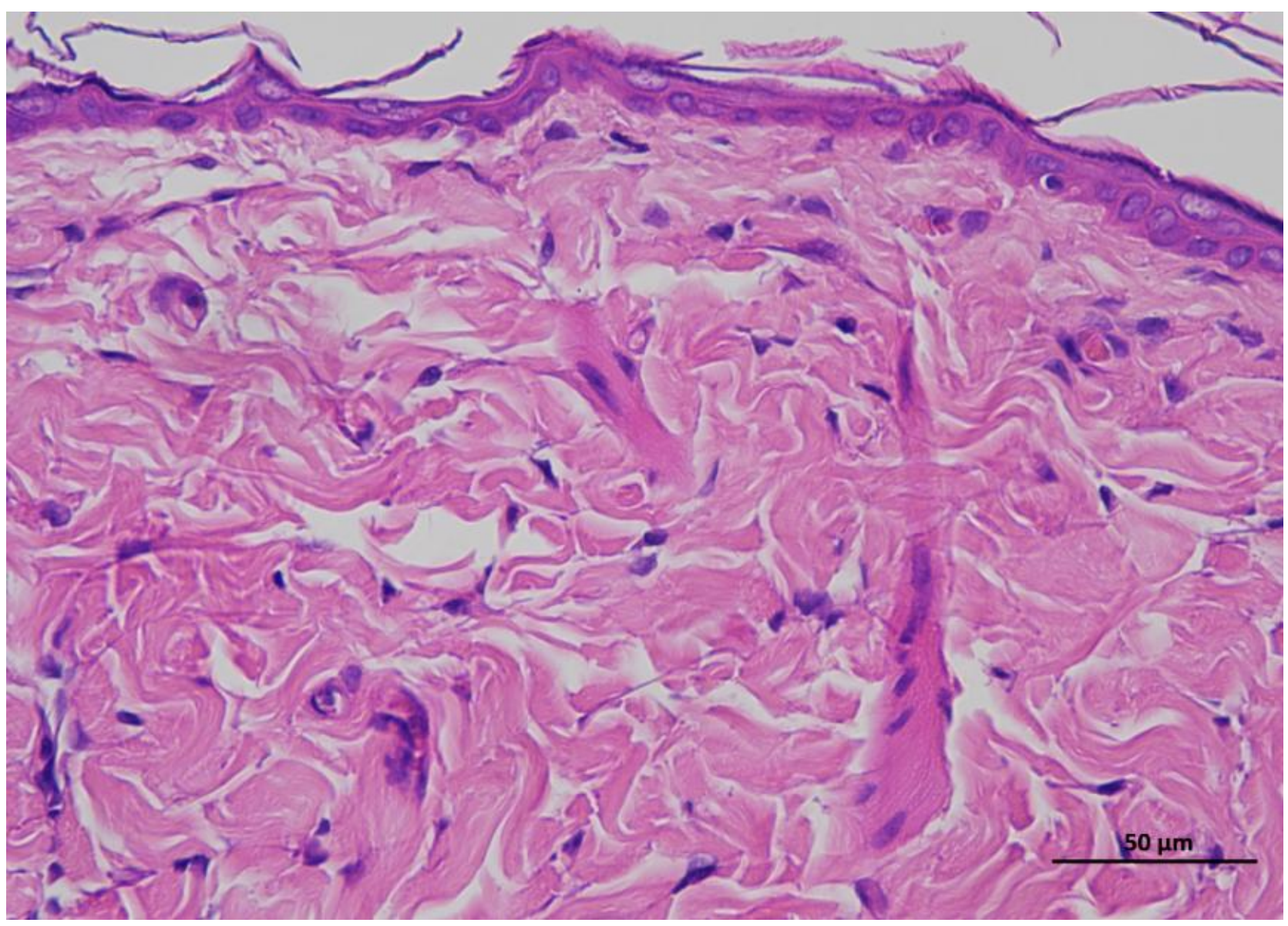

5 DControl 14day 400-2

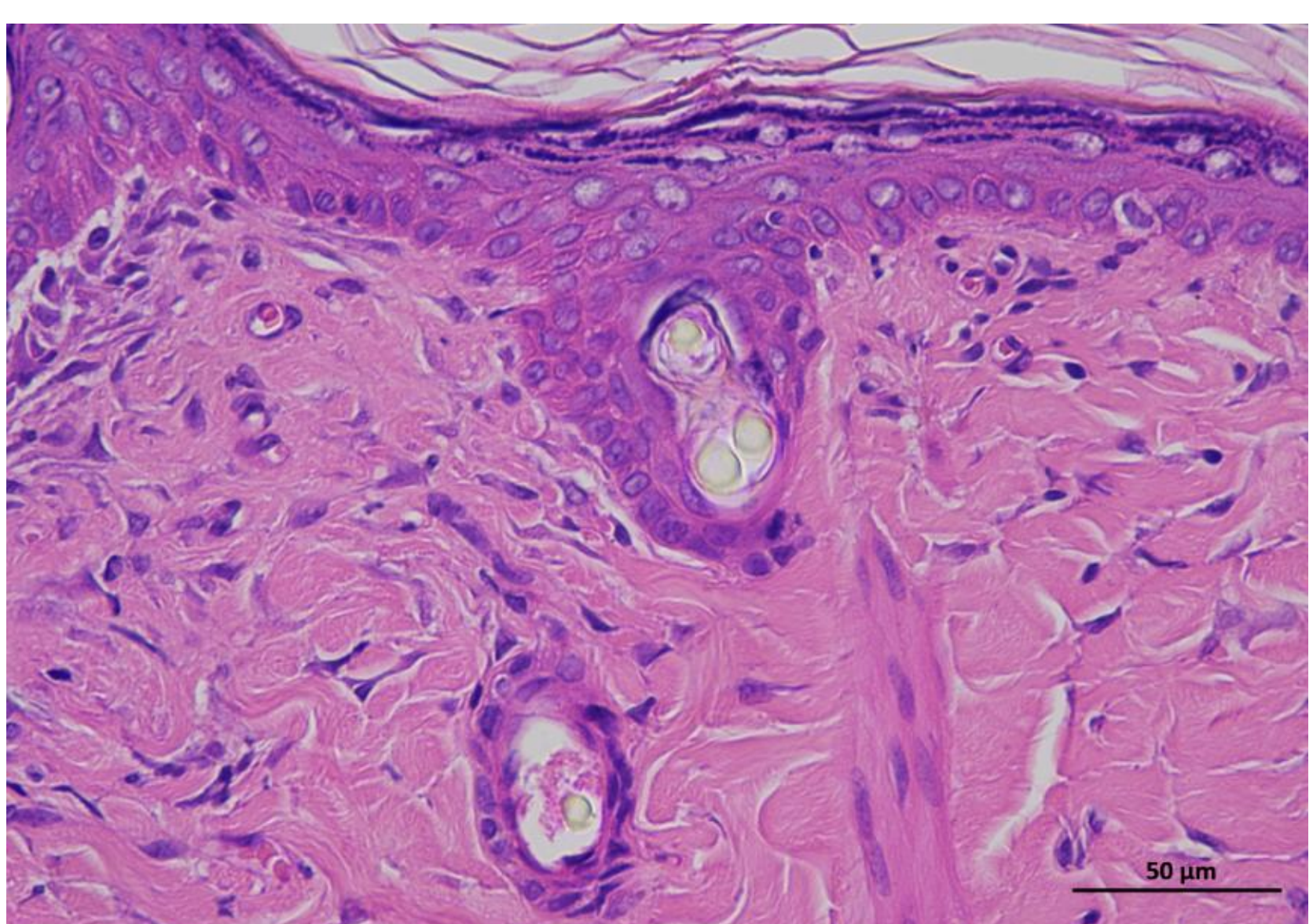

5 DControl 14day 400-3

Supplementary Figure 3b | Representative H&E staining of skin tissue of diabetic control animals / Day 14.

Animal 6

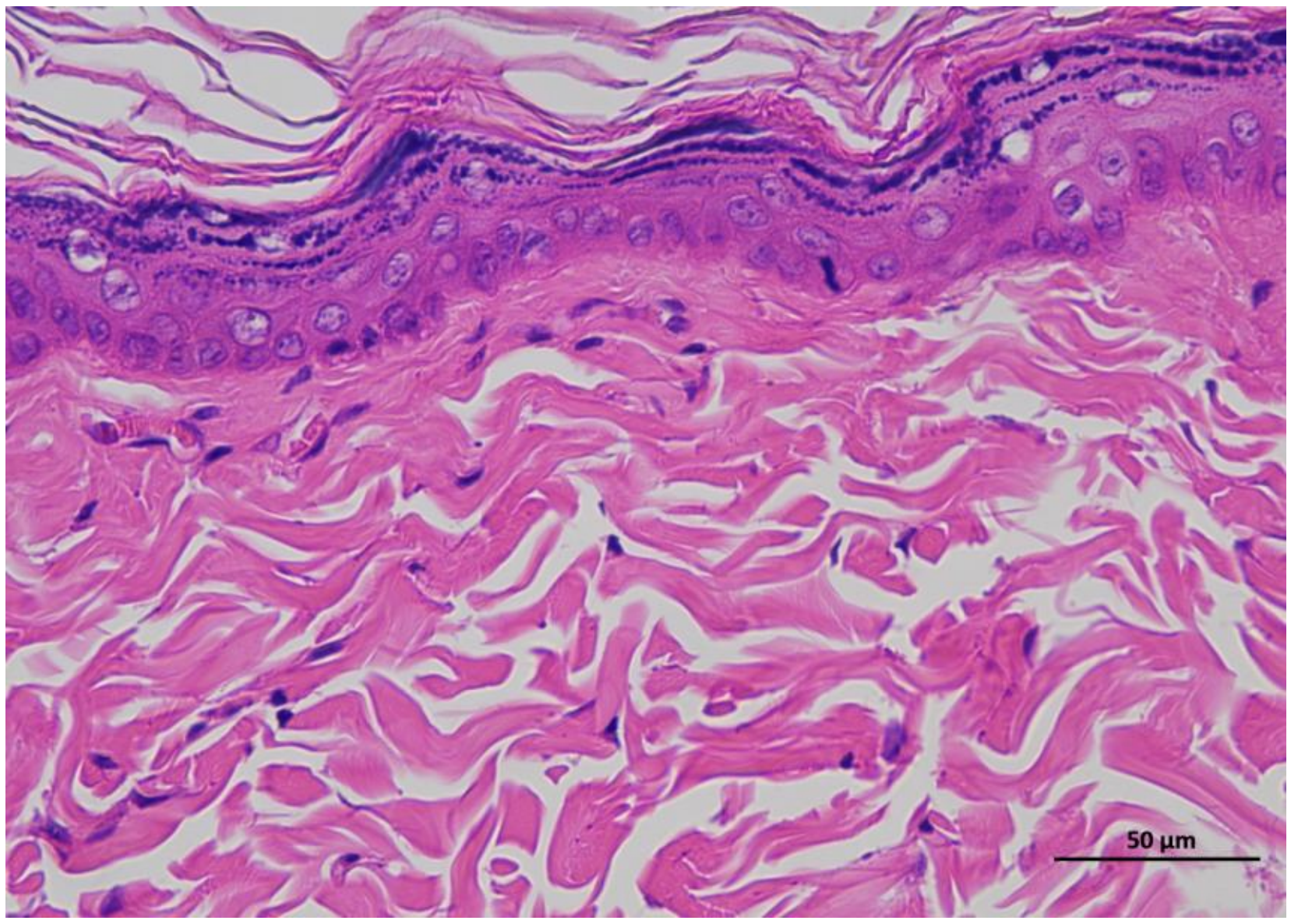

6 DControl 14day 400-1

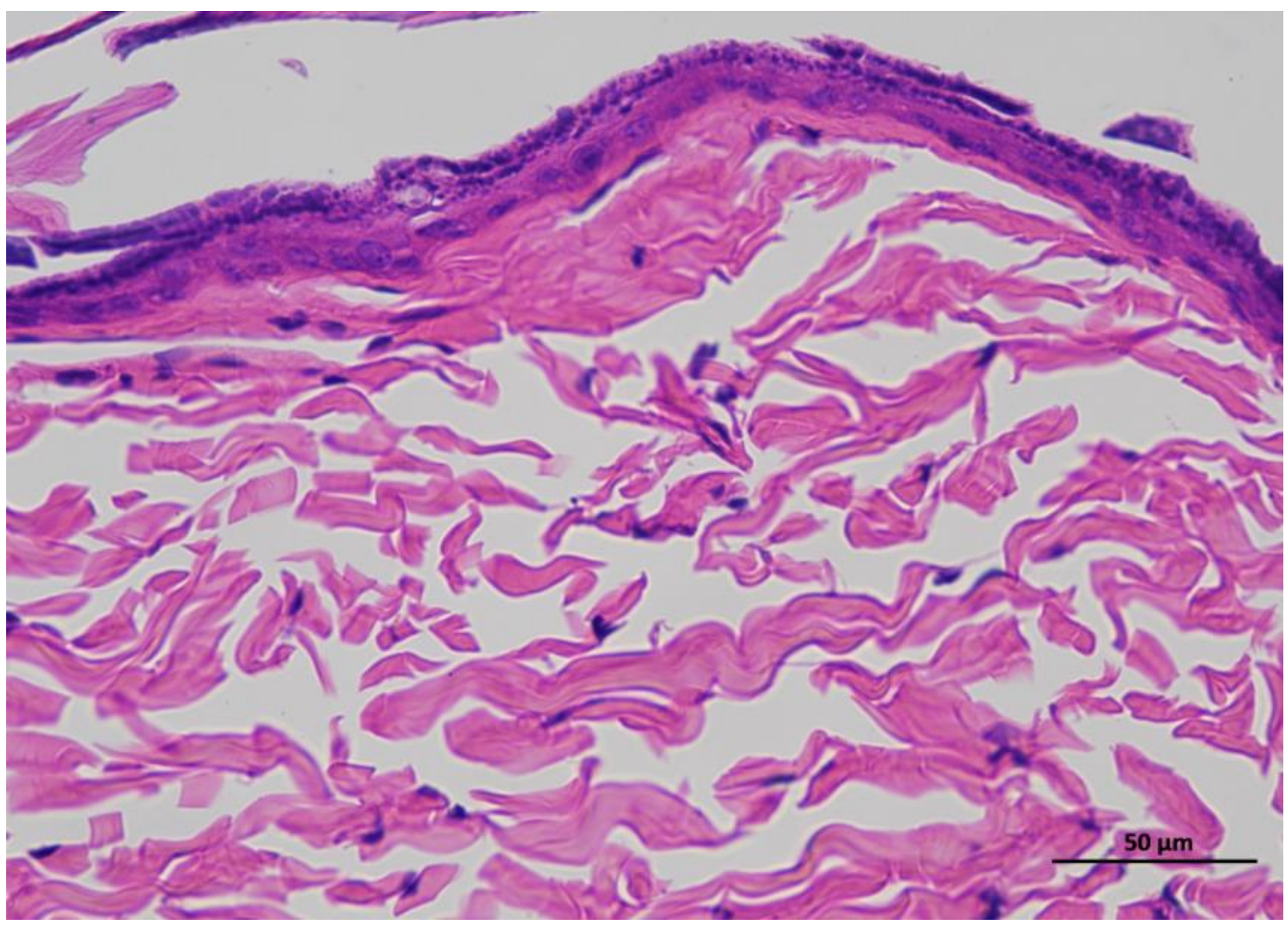

6 DControl 14day 400-2

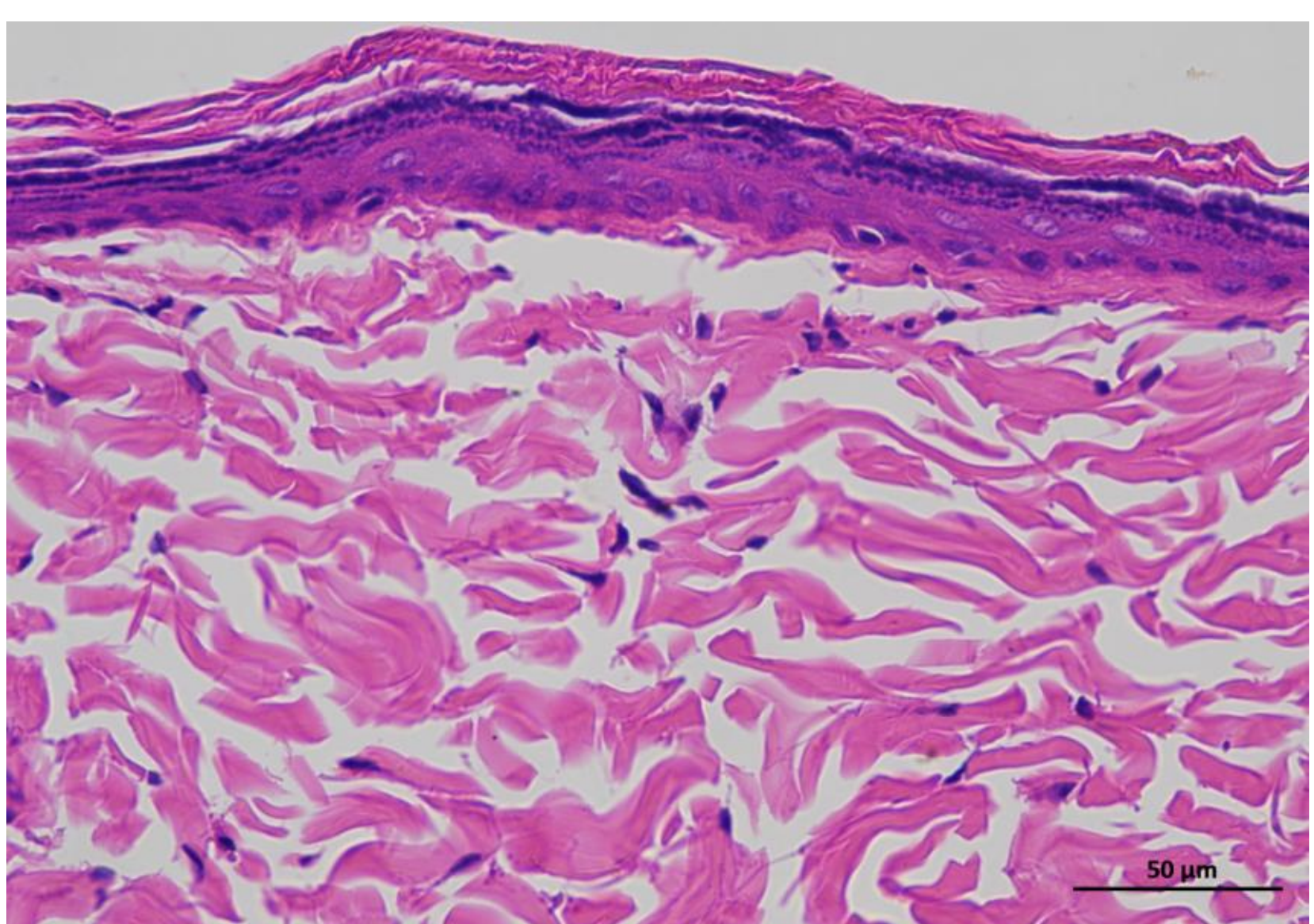

6 DControl 14day 400-3

Animal 1

Supplementary Figure 2c | Representative H&E staining of skin tissue of RNA-LNPs-L animals / Day 0. Representative hematoxylin and eosin (H&E)–stained section of skin tissue collected from experimental rat. The image shows the typical architecture of the skin, including the epidermal layer with stratified keratinized epithelium and the underlying dermis composed of dense collagen fibers and connective tissue structures.
(n = 6 per group).

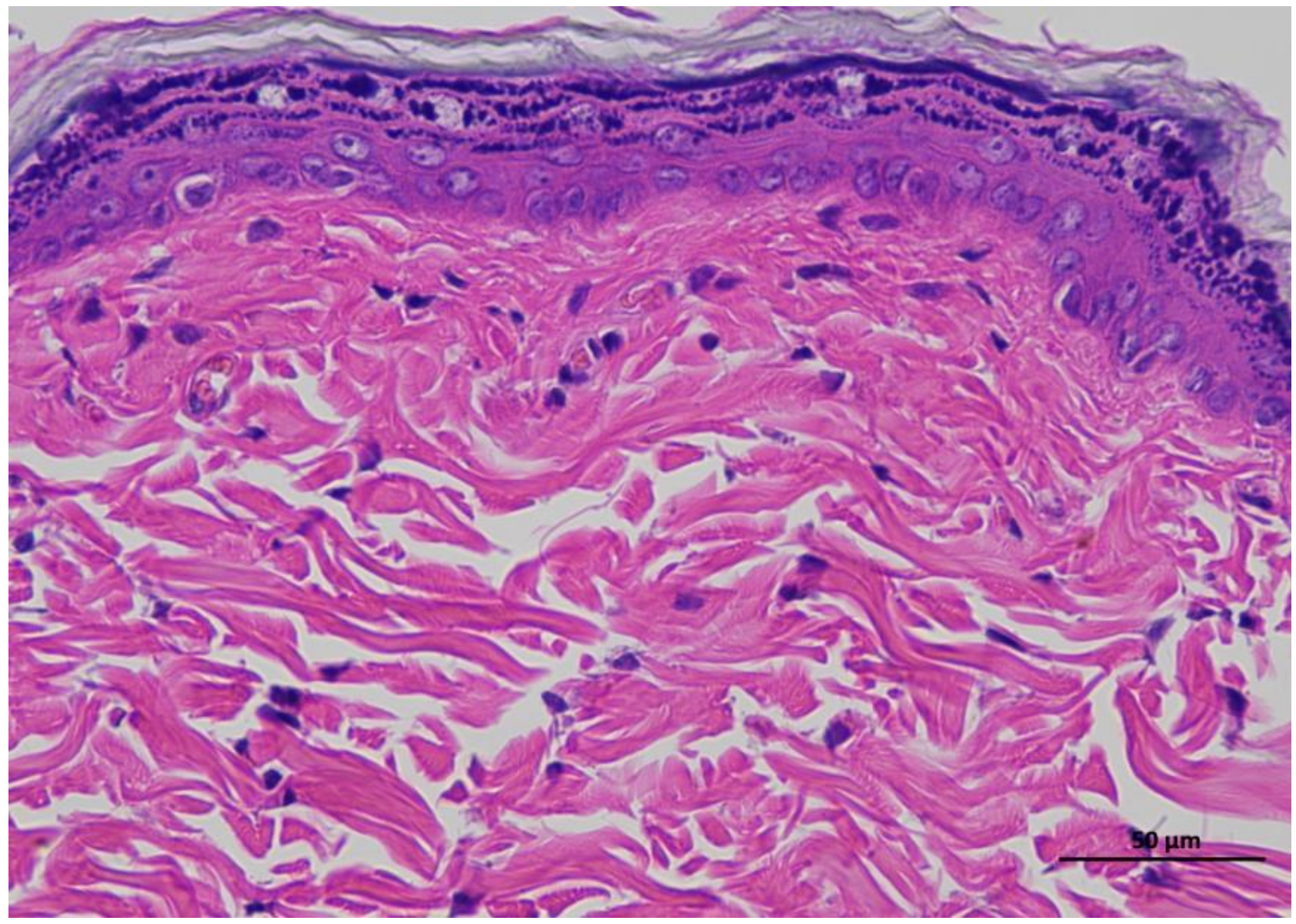

1 RNA-LNPs-L 0day 400-1

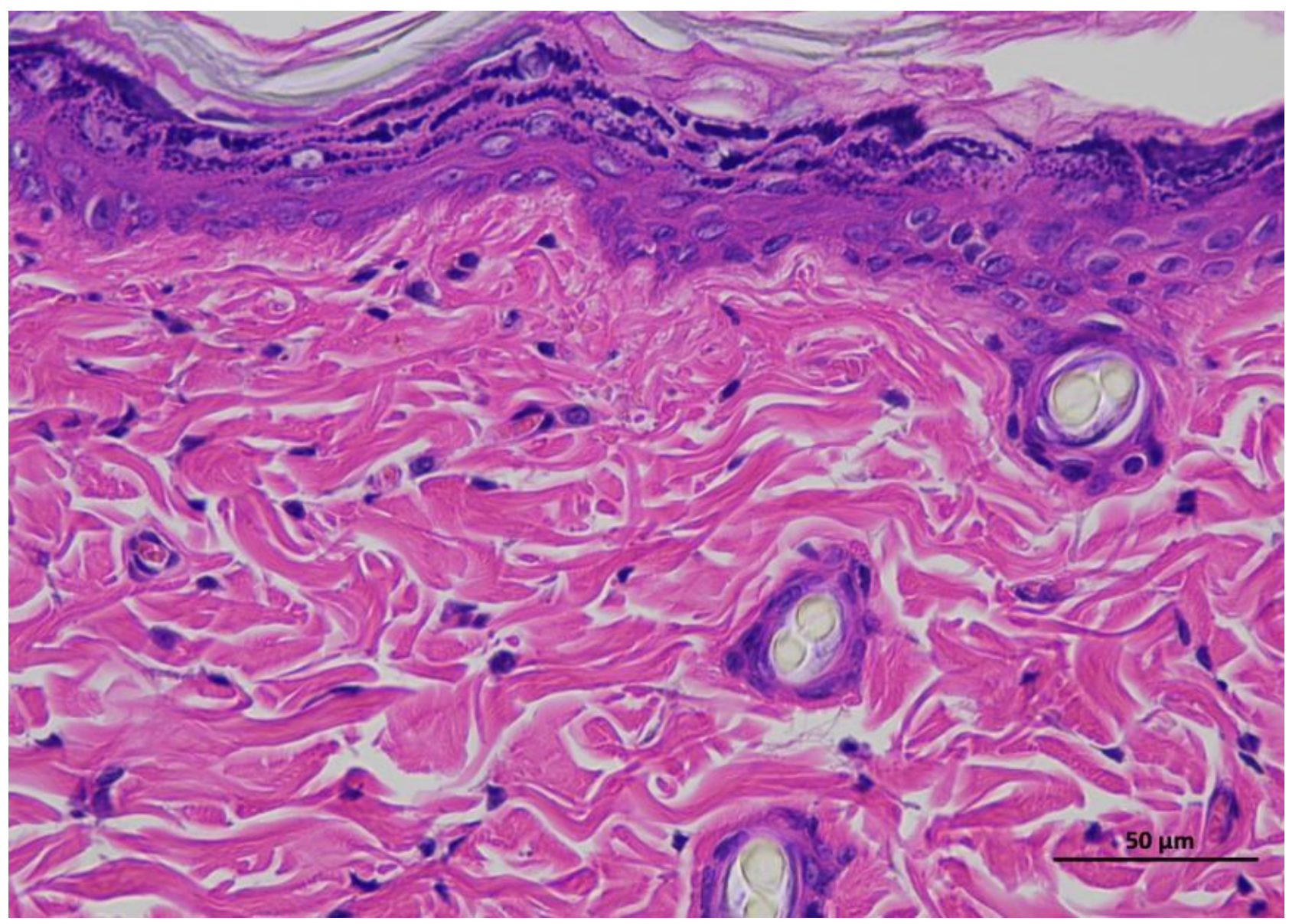

1 RNA-LNPs-L 0day 400-2

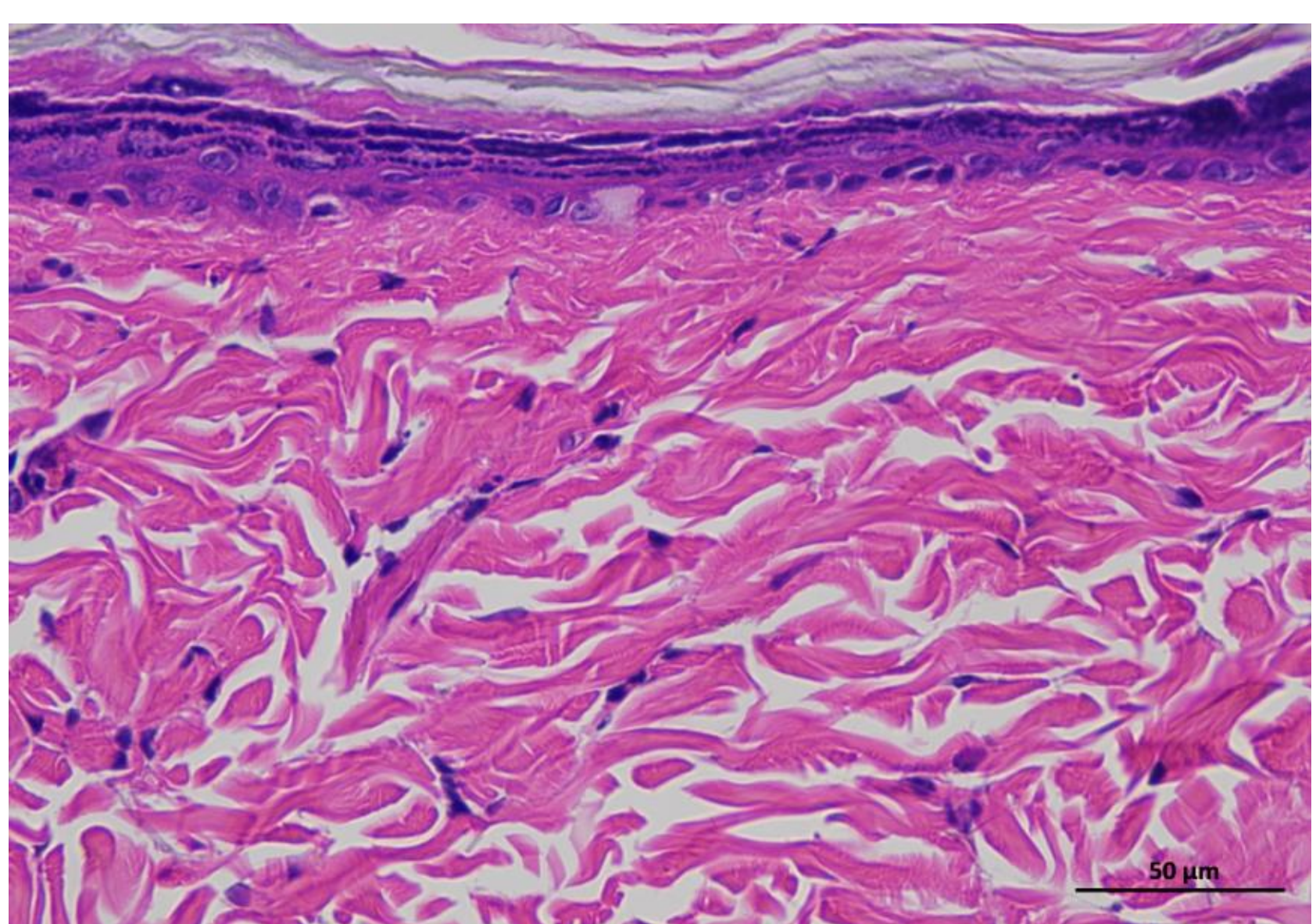

1 RNA-LNPs-L 0day 400-3

Animal 2

Supplementary Figure 2c | Representative H&E staining of skin tissue of RNA-LNPs-L animals / Day 0.

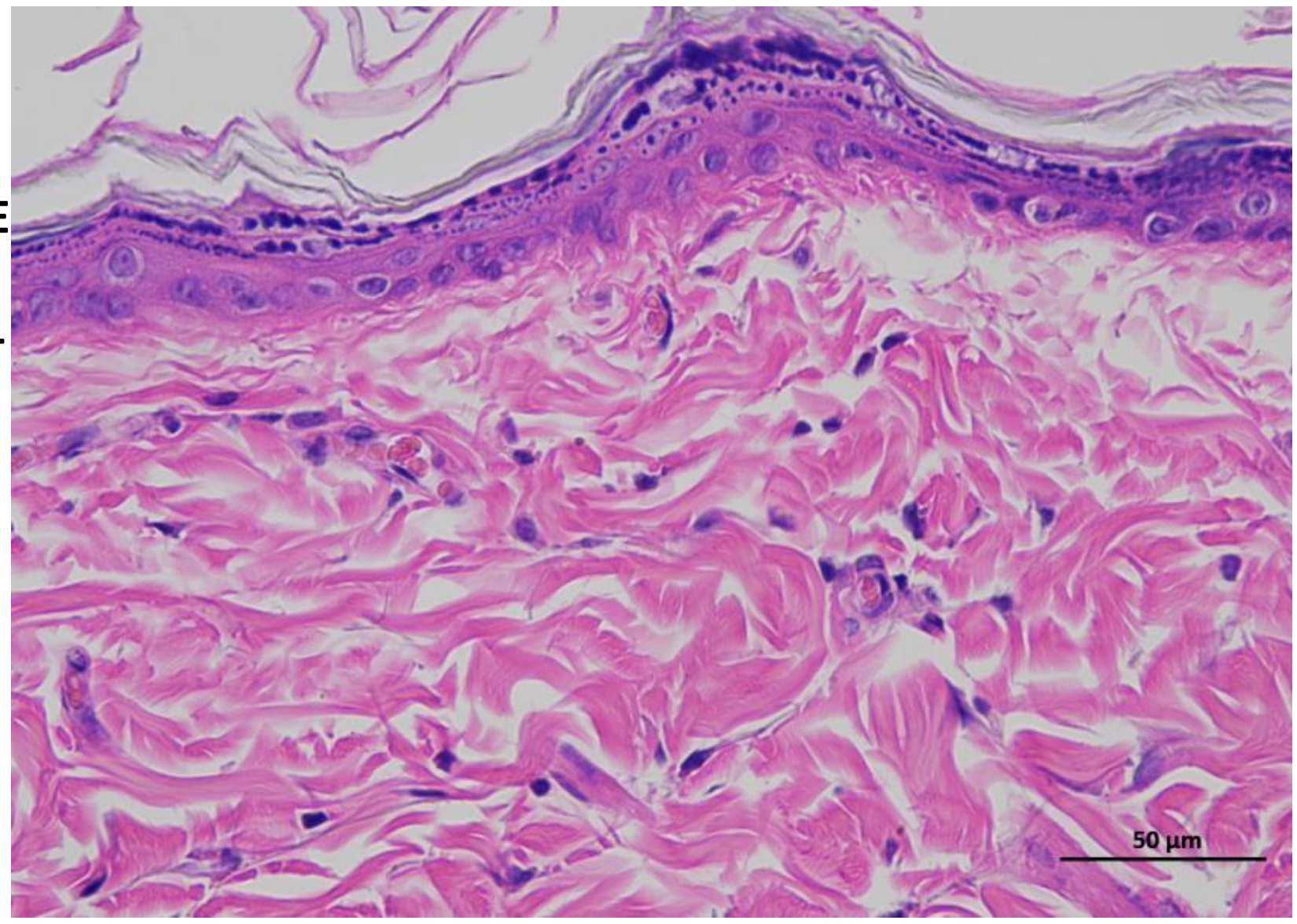

2 RNA-LNPs-L 0day 400-1

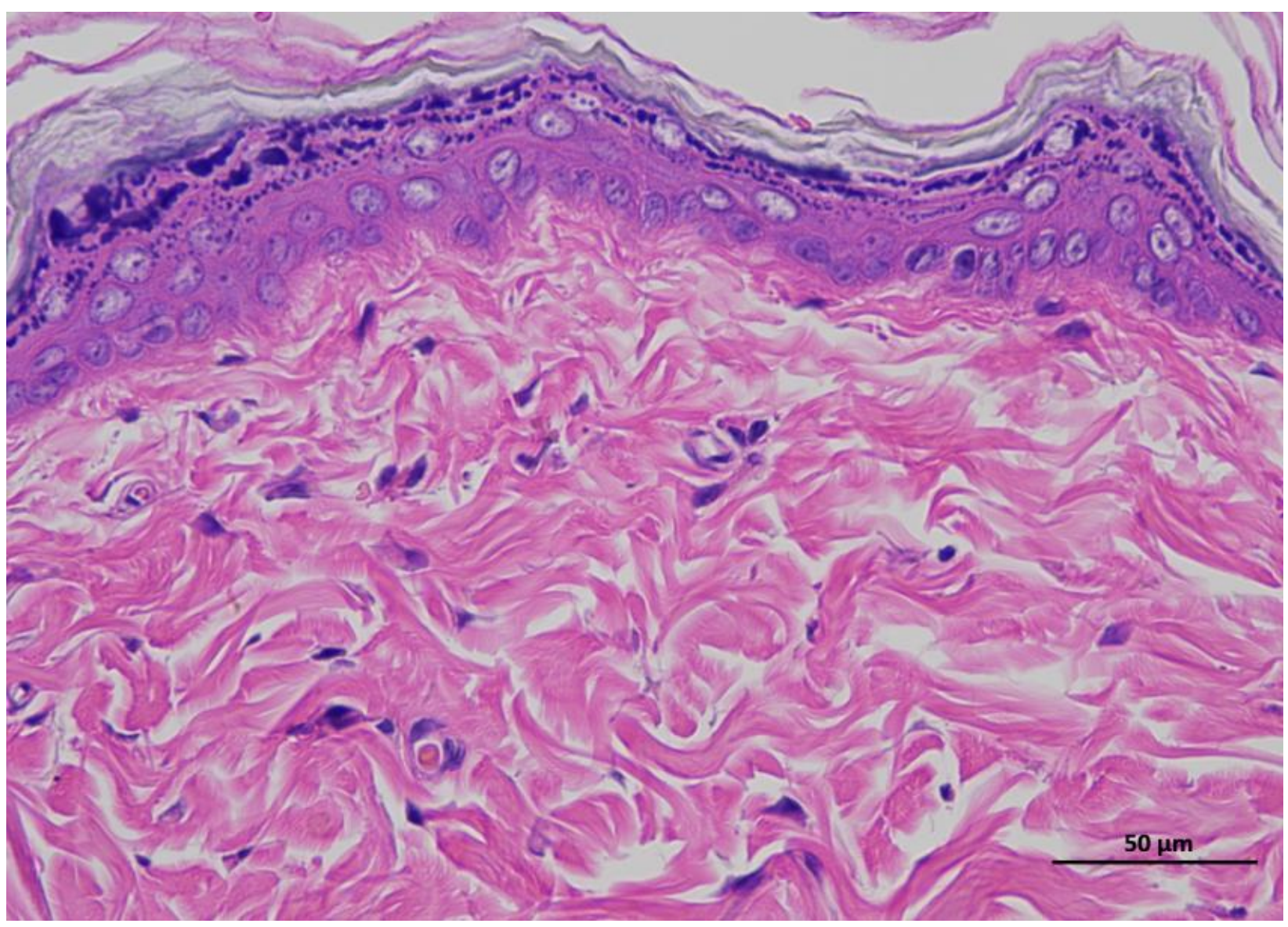

2 RNA-LNPs-L 0day 400-2

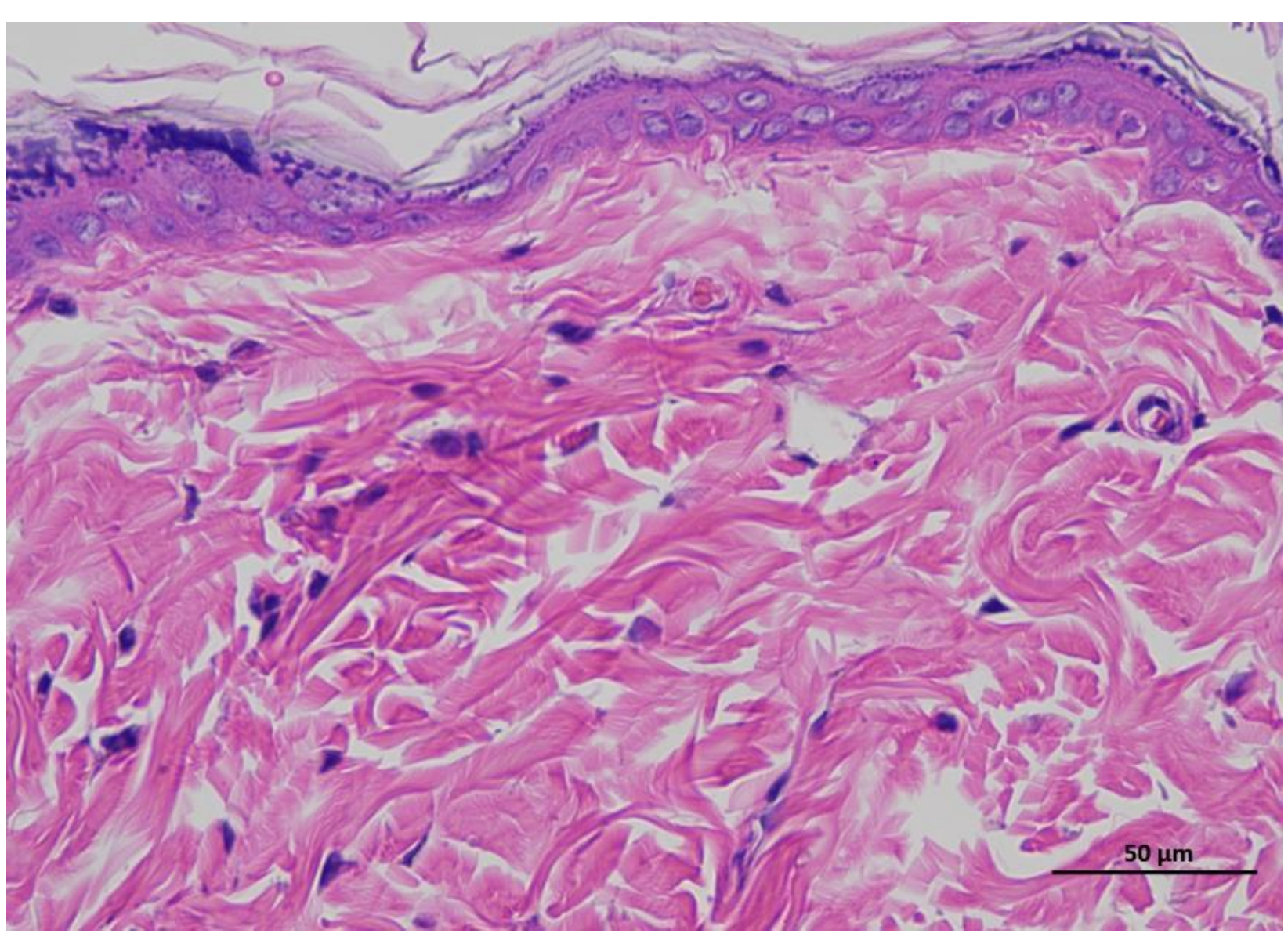

2 RNA-LNPs-L 0day 400-3

Animal 3

Supplementary Figure 2c | Representative H&E staining of skin tissue of RNA-LNPs-L animals / Day 0.

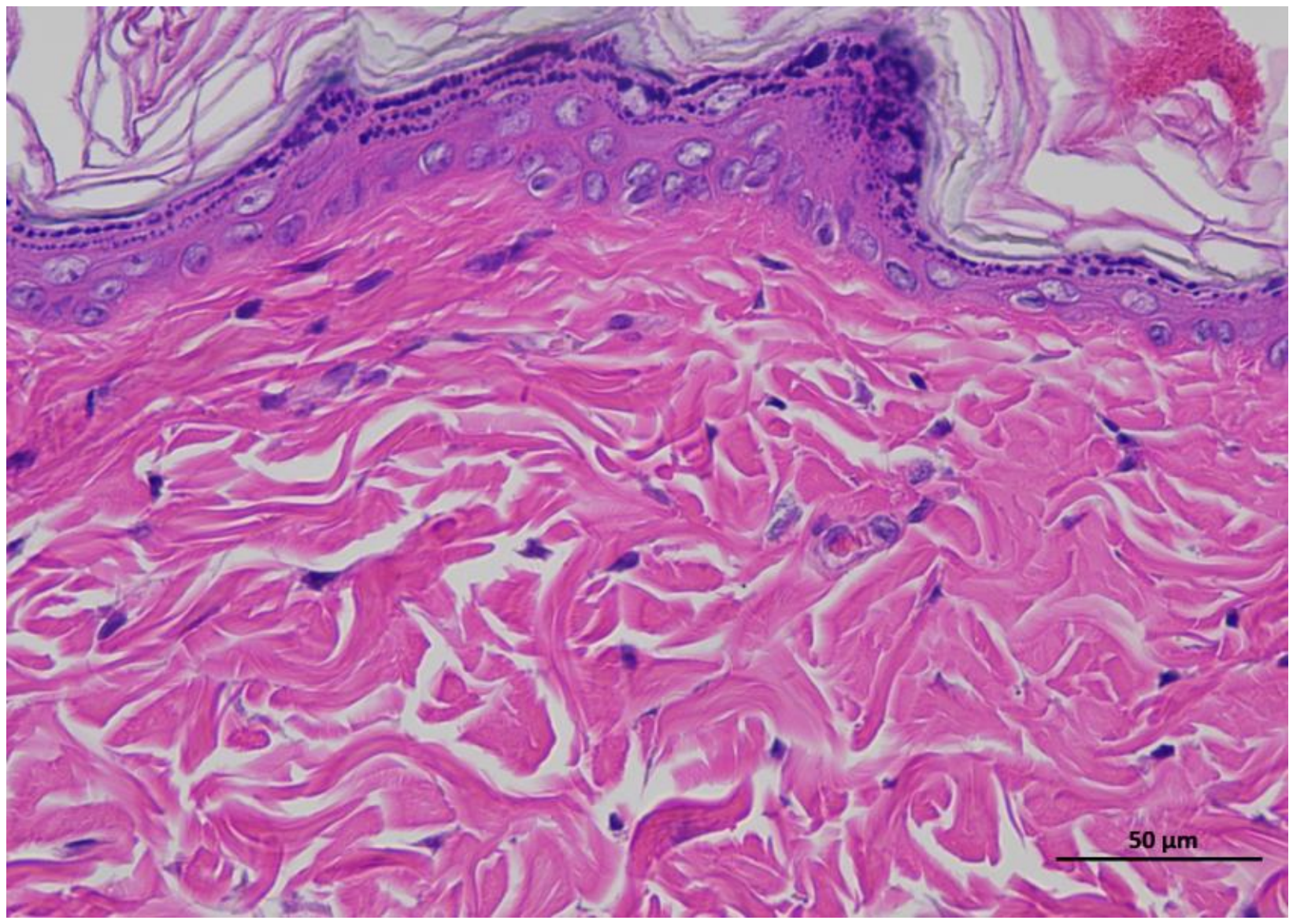

3 RNA-LNPs-L 0day 400-1

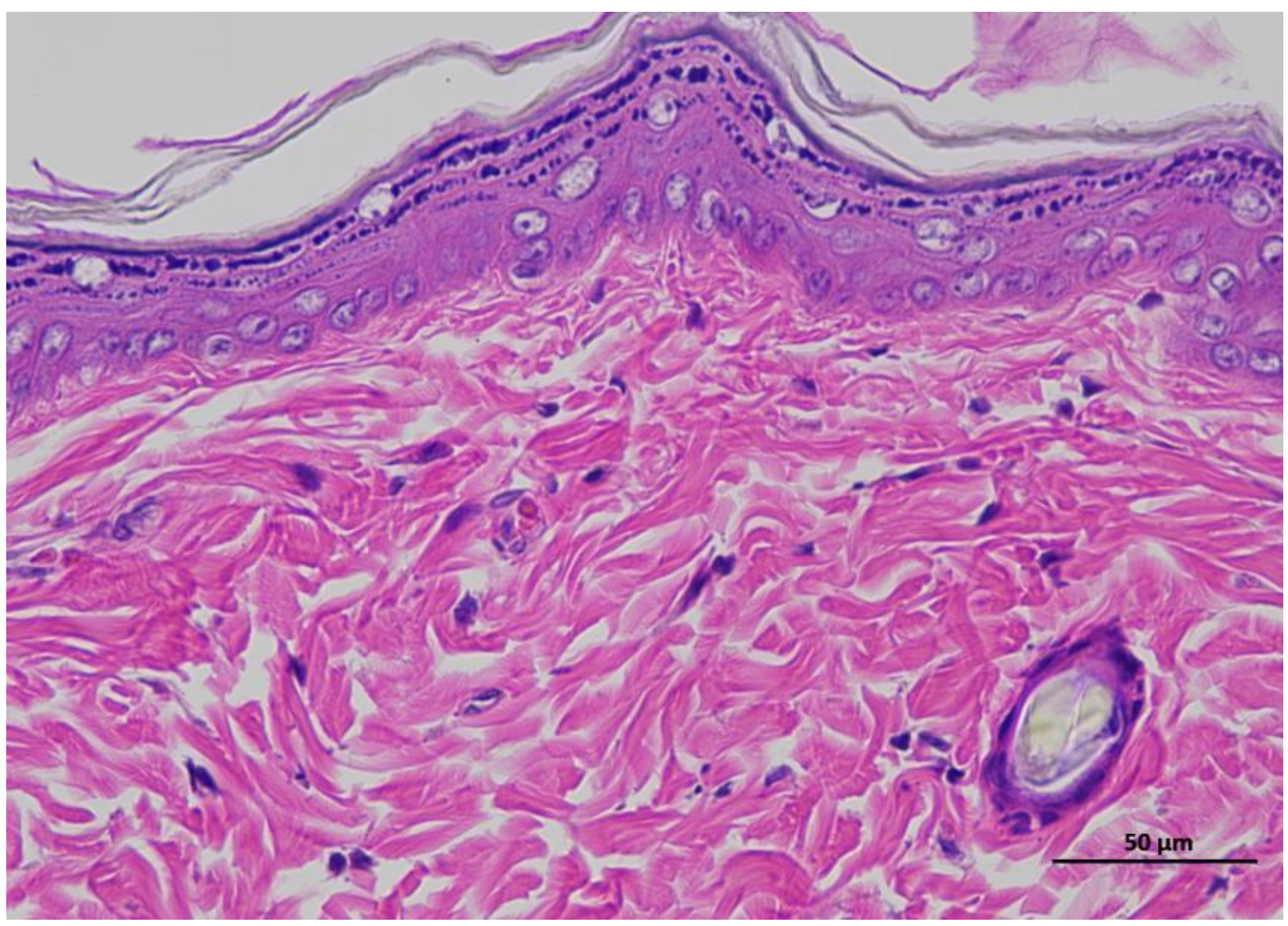

3 RNA-LNPs-L 0day 400-2

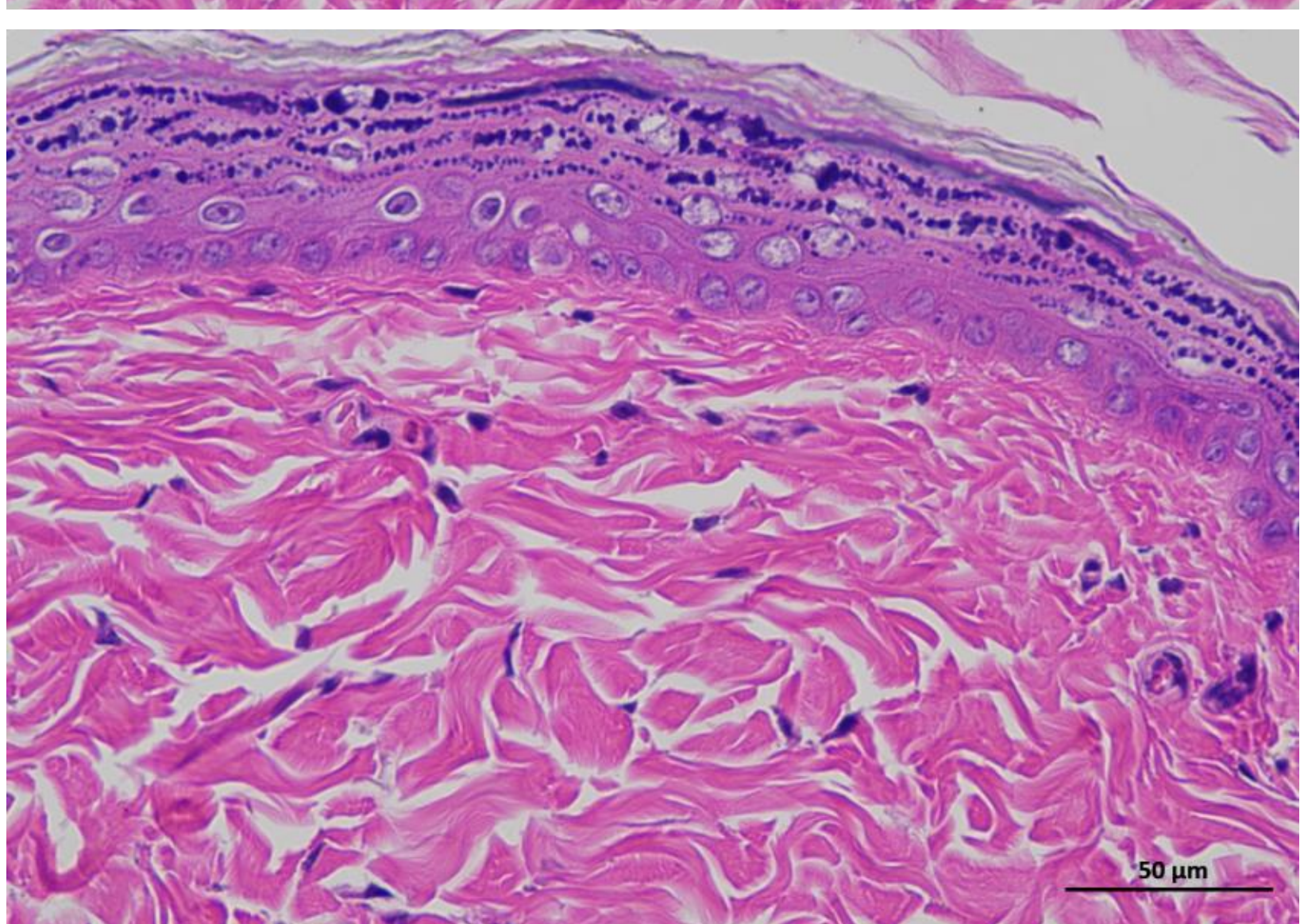

3 RNA-LNPs-L 0day 400-3

Animal 4

Supplementary Figure 2c | Representative H&E staining of skin tissue of RNA-LNPs-L animals / Day 0.

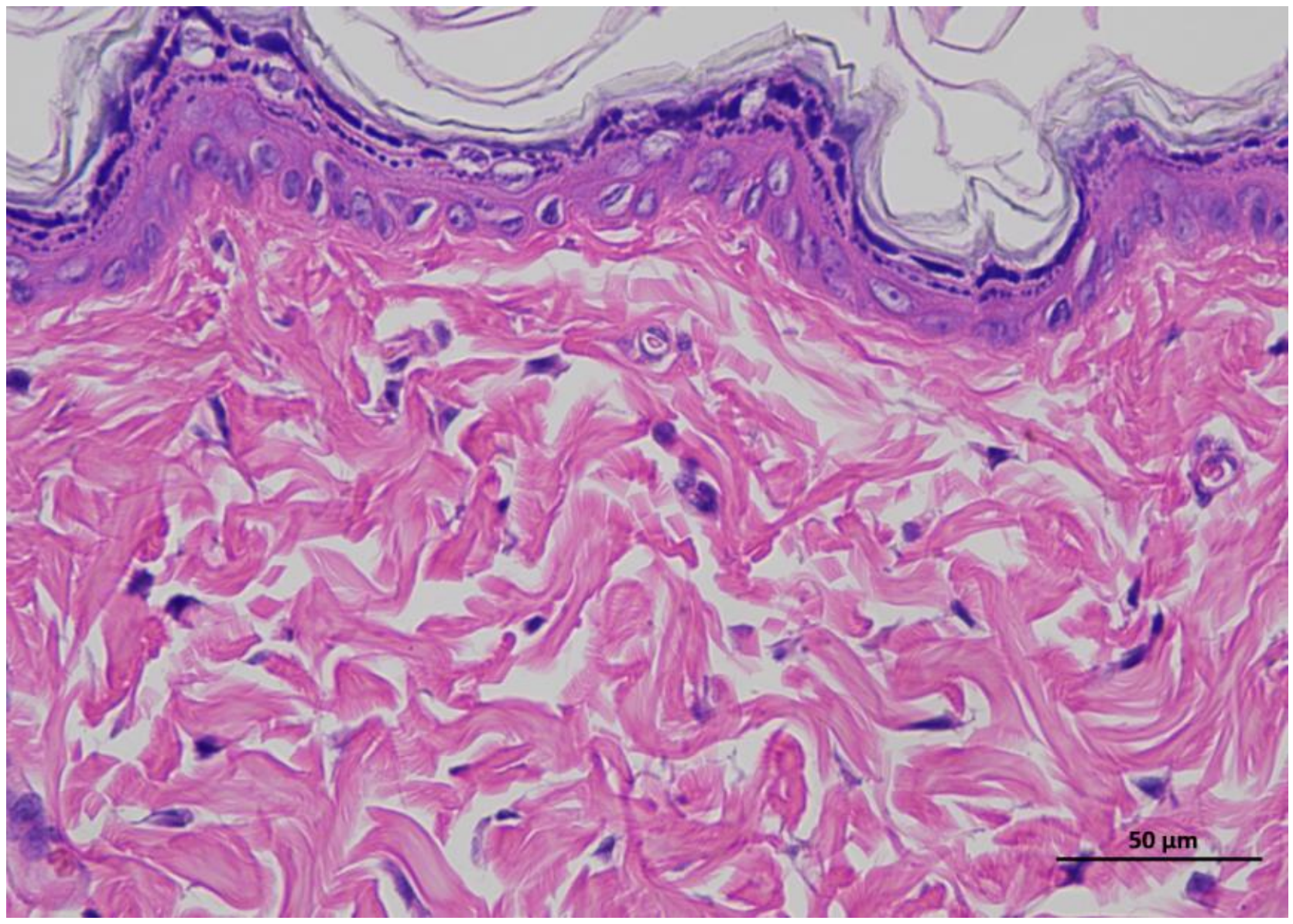

4 RNA-LNPs-L 0day 400-1

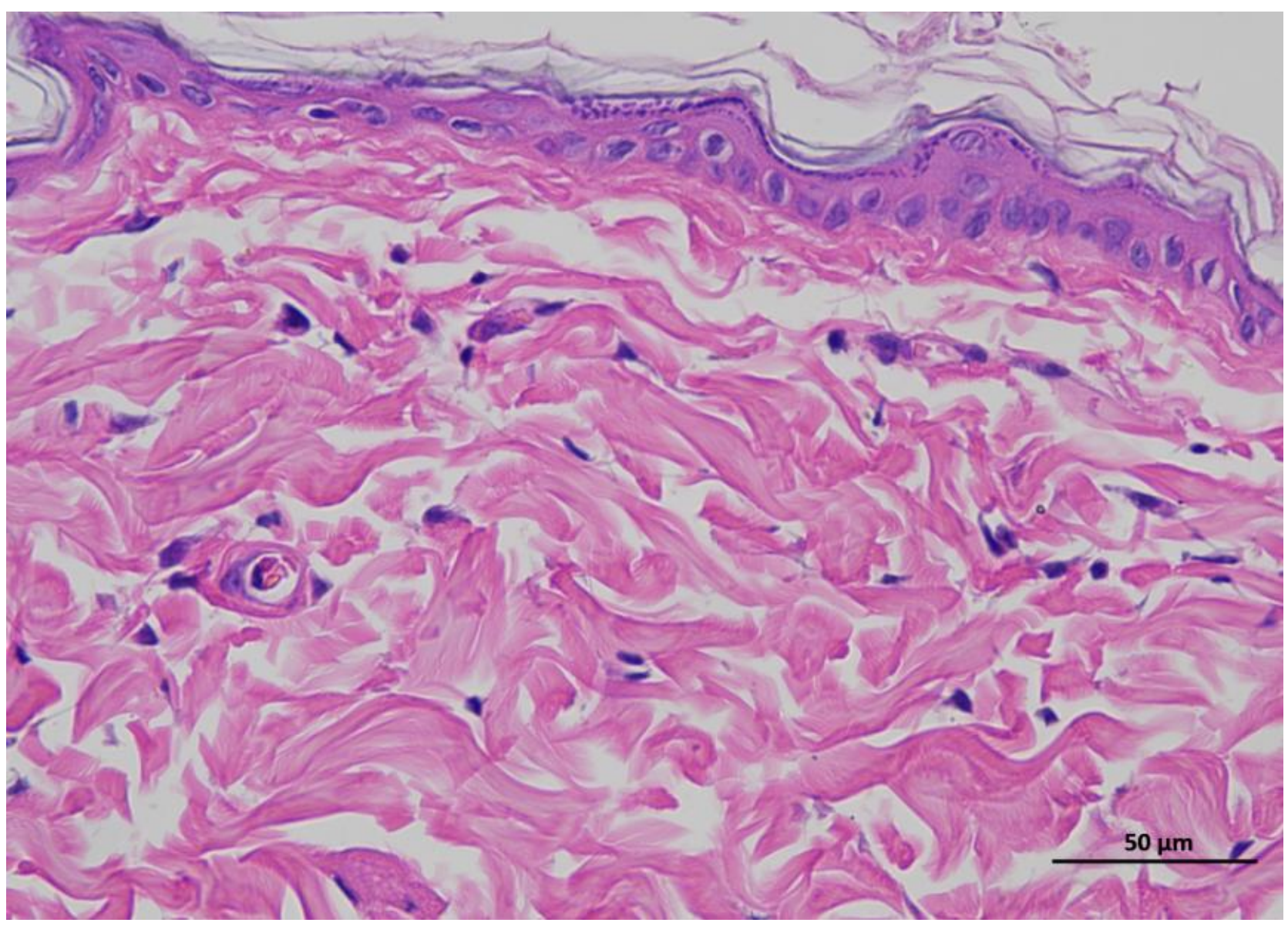

4 RNA-LNPs-L 0day 400-2

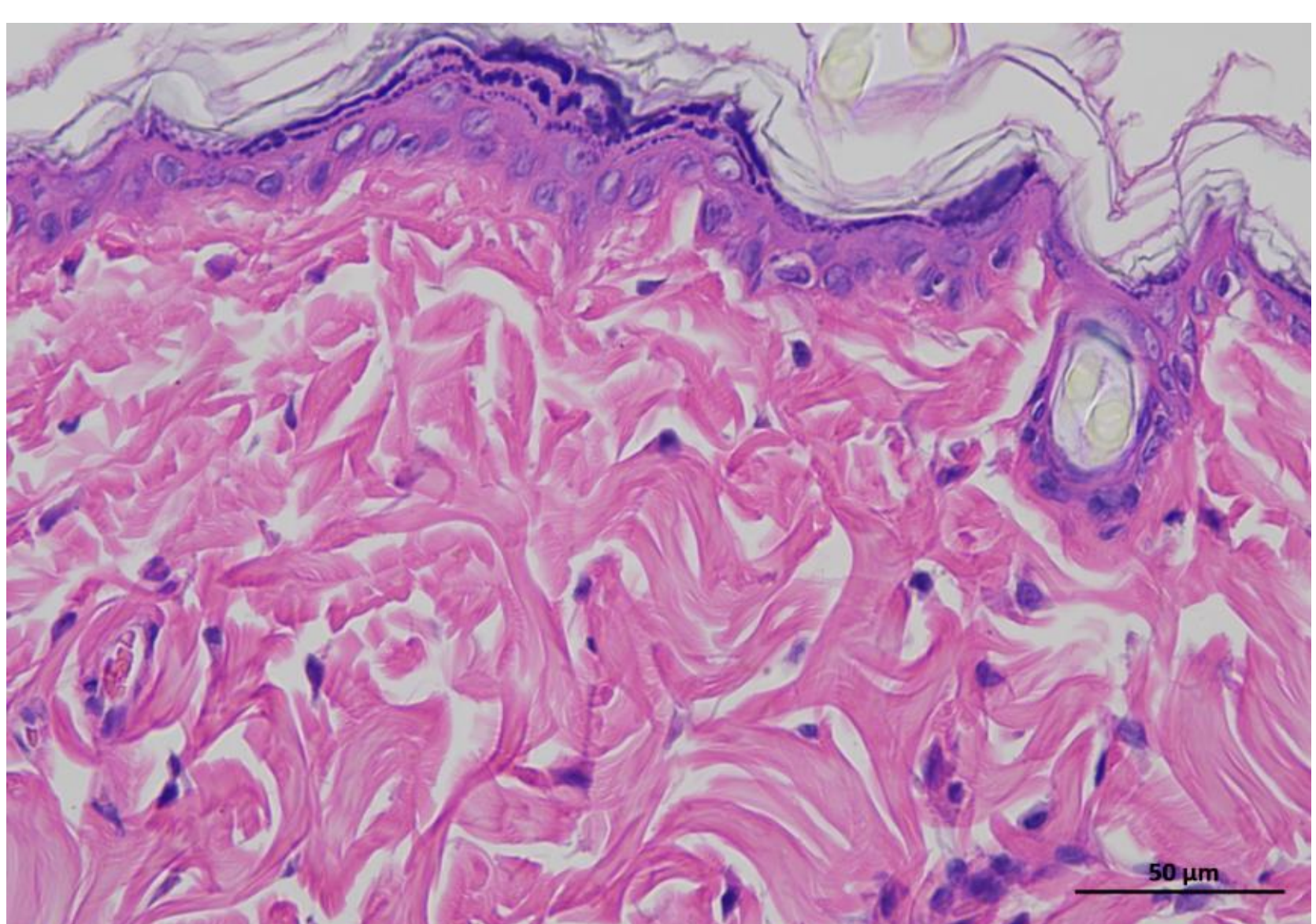

4 RNA-LNPs-L 0day 400-3

Animal 5

Supplementary Figure 2c | Representative H&E staining of skin tissue of RNA-LNPs-L animals / Day 0.

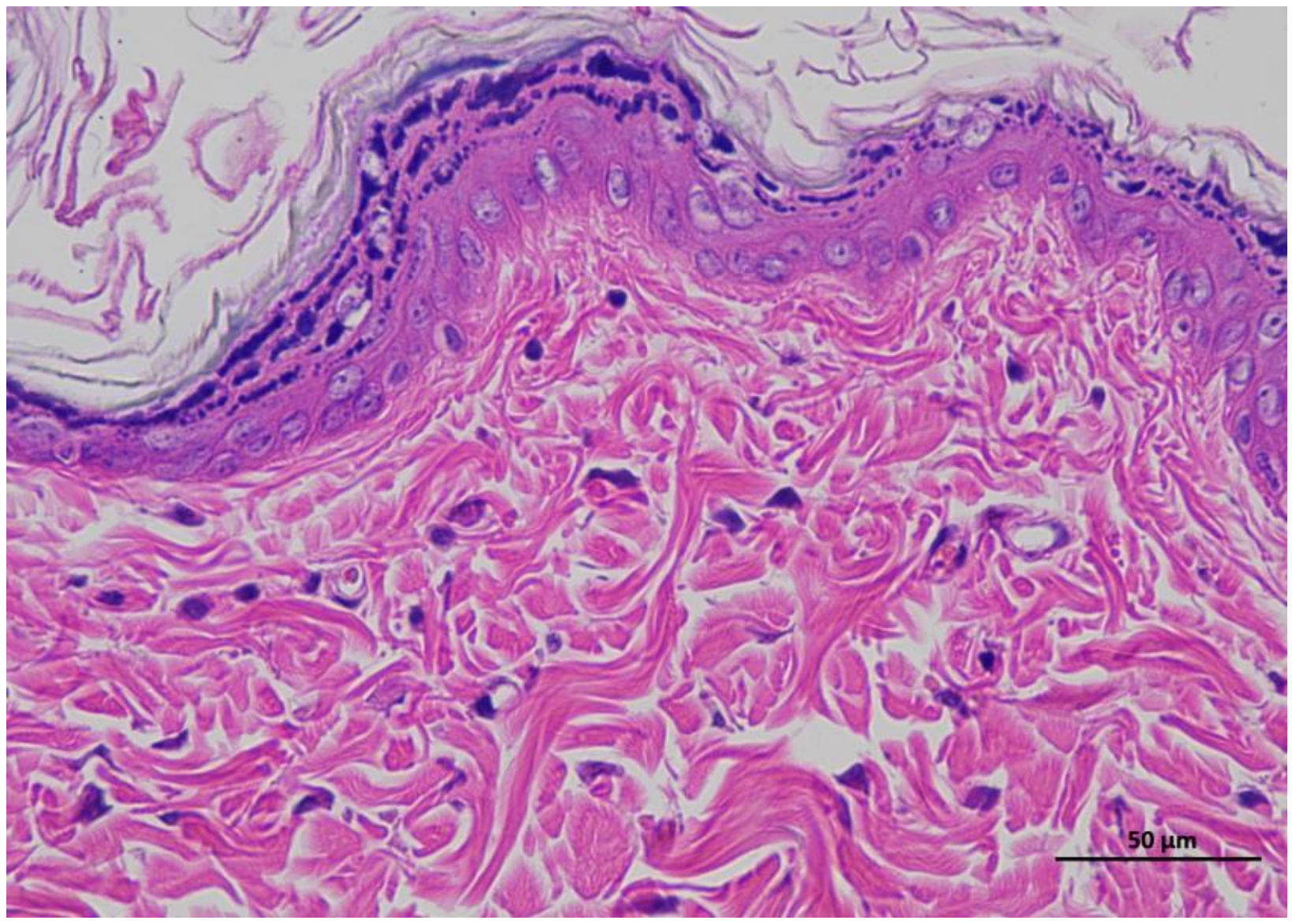

5 RNA-LNPs-L 0day 400-1

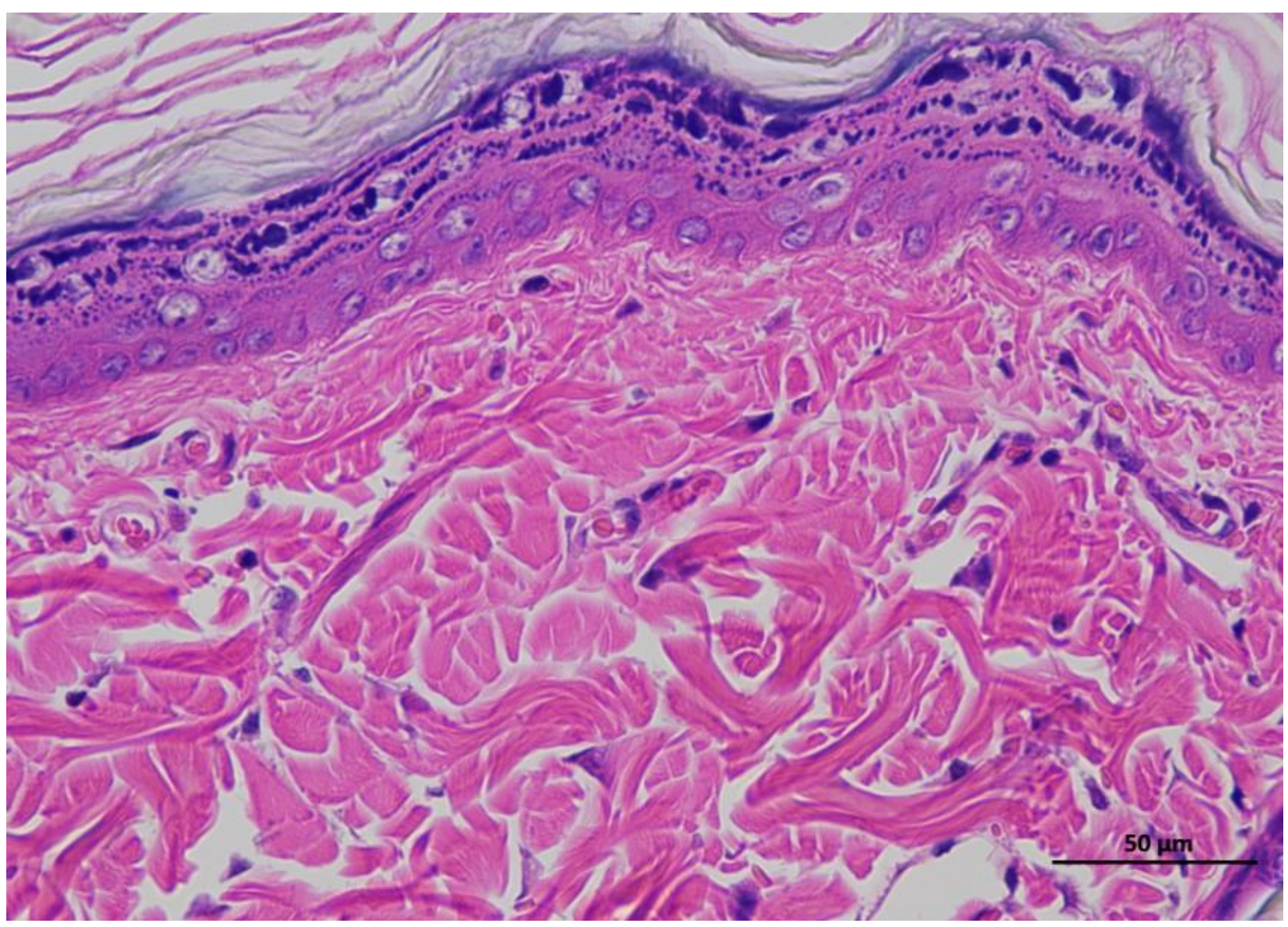

5 RNA-LNPs-L 0day 400-2

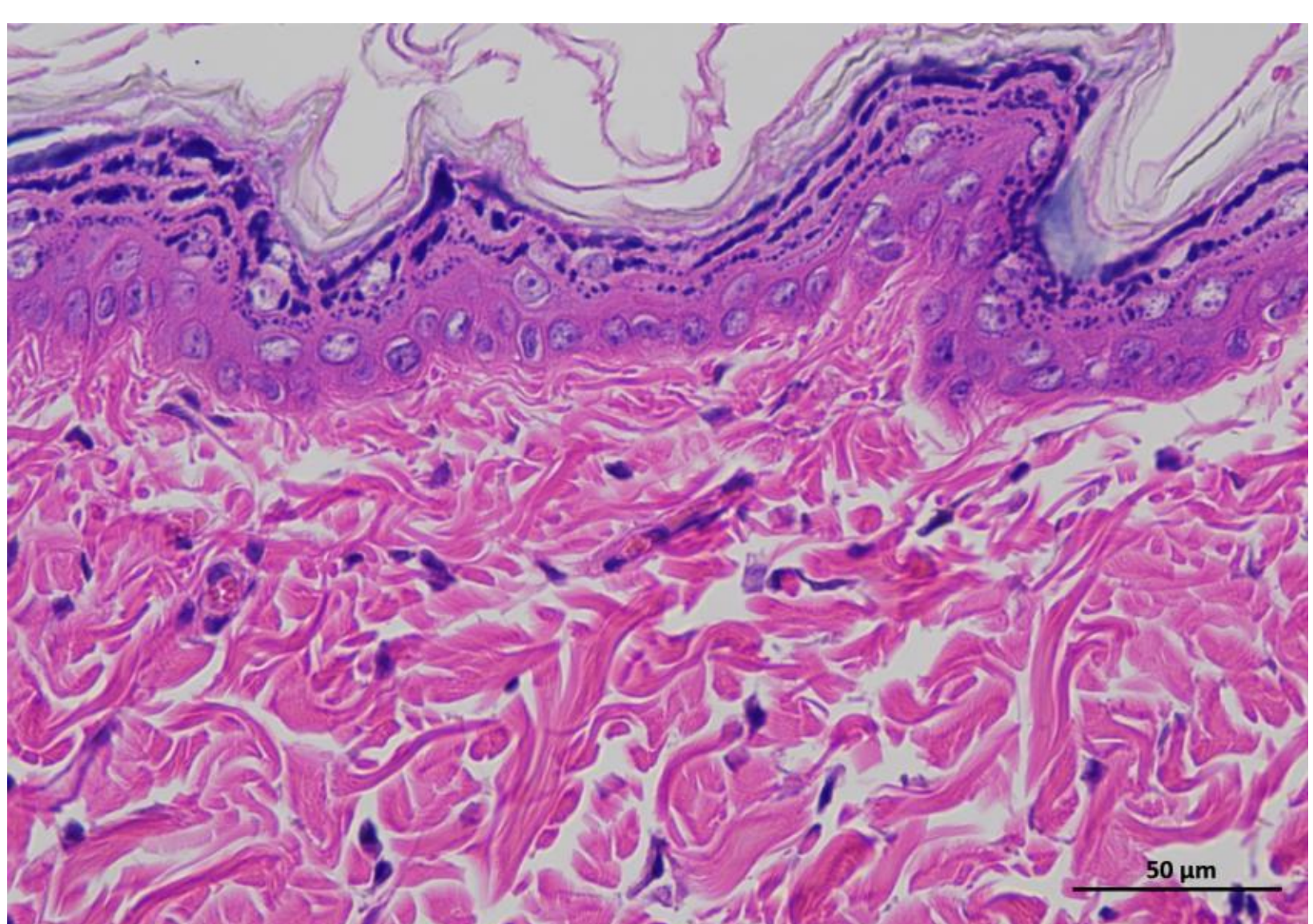

5 RNA-LNPs-L 0day 400-3

Animal 6

Supplementary Figure 2c | Representative H&E staining of skin tissue of RNA-LNPs-L animals / Day 0.

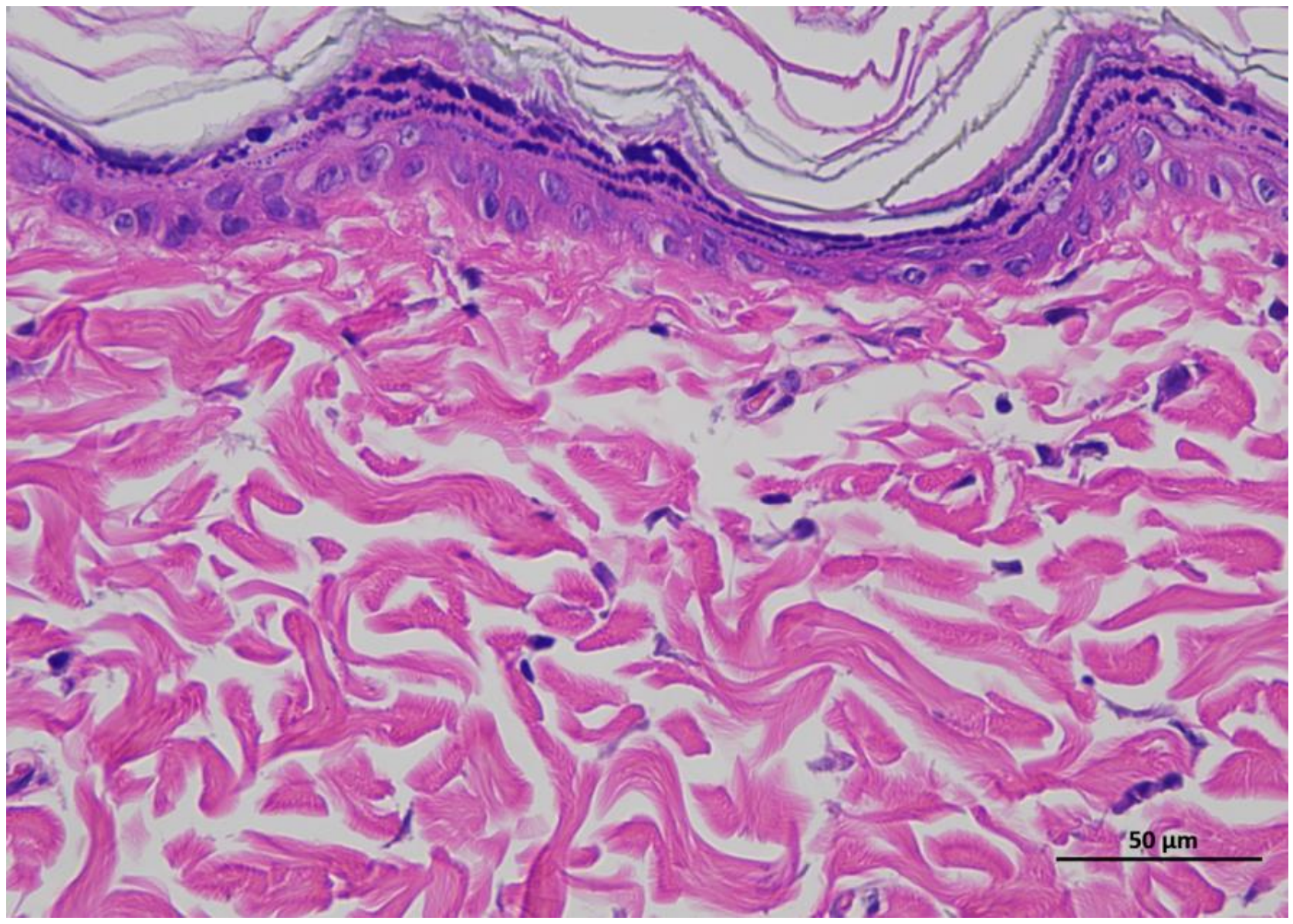

6 RNA-LNPs-L 0day 400-1

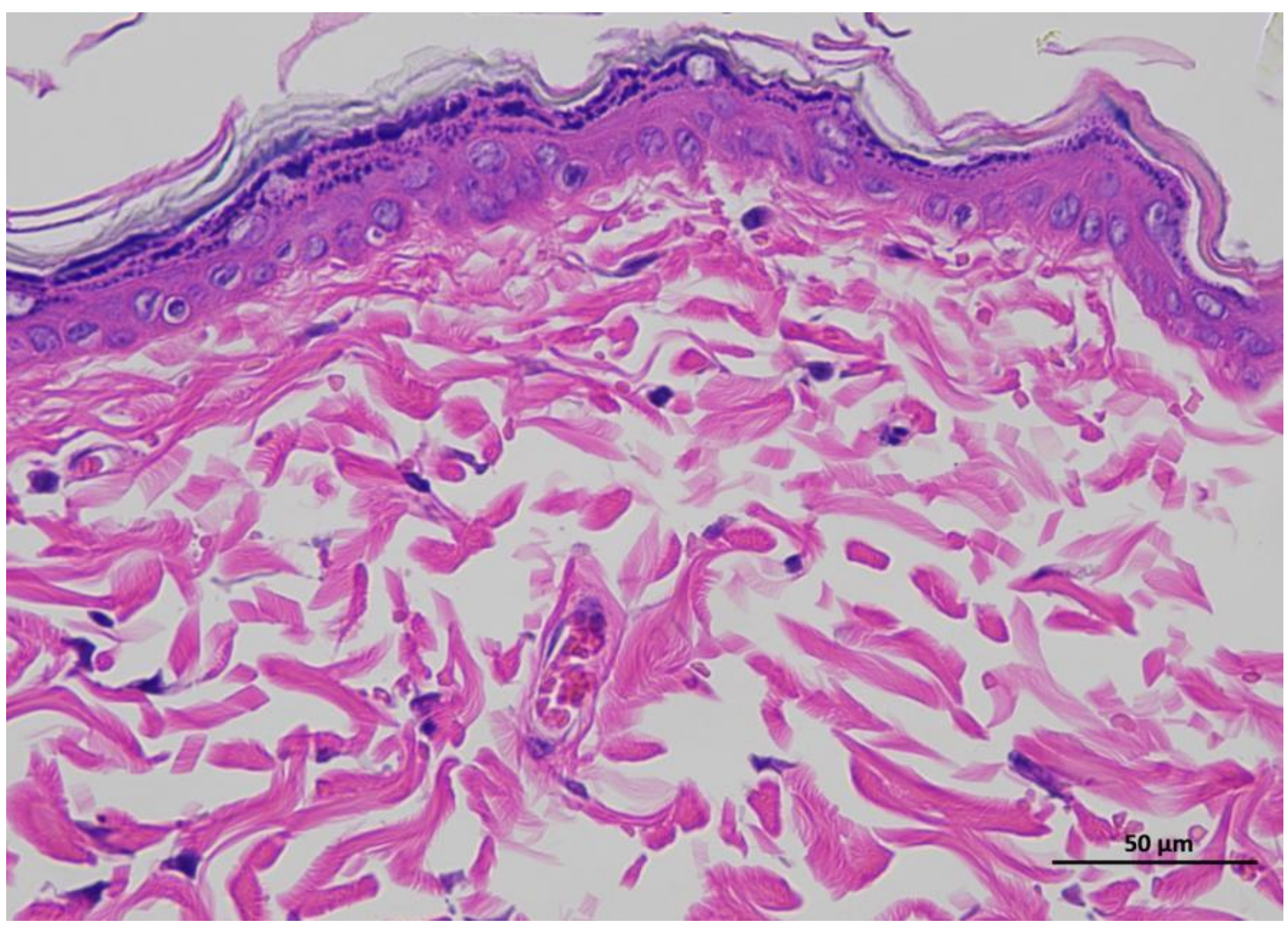

6 RNA-LNPs-L 0day 400-2

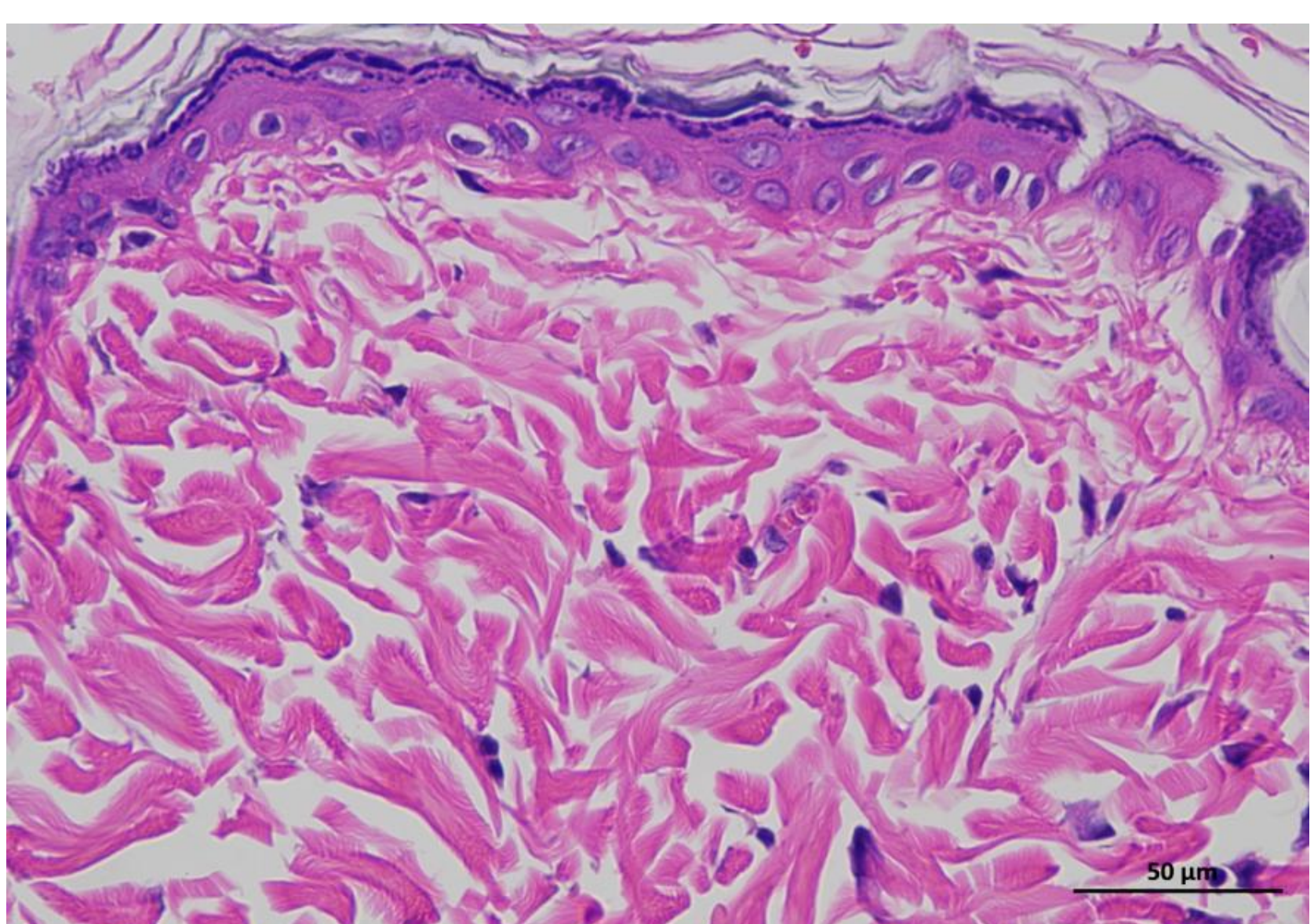

6 RNA-LNPs-L 0day 400-3

Supplementary Figure 3c | Representative H&E staining of skin tissue of RNA-LNPs-L animals / Day 14.
Representative hematoxylin and eosin (H&E)–stained section of skin tissue collected from experimental rat. The image shows the typical architecture of the skin, including the epidermal layer with stratified keratinized epithelium and the underlying dermis composed of dense collagen fibers and connective tissue structures.
(n = 6 per group).

Animal 1

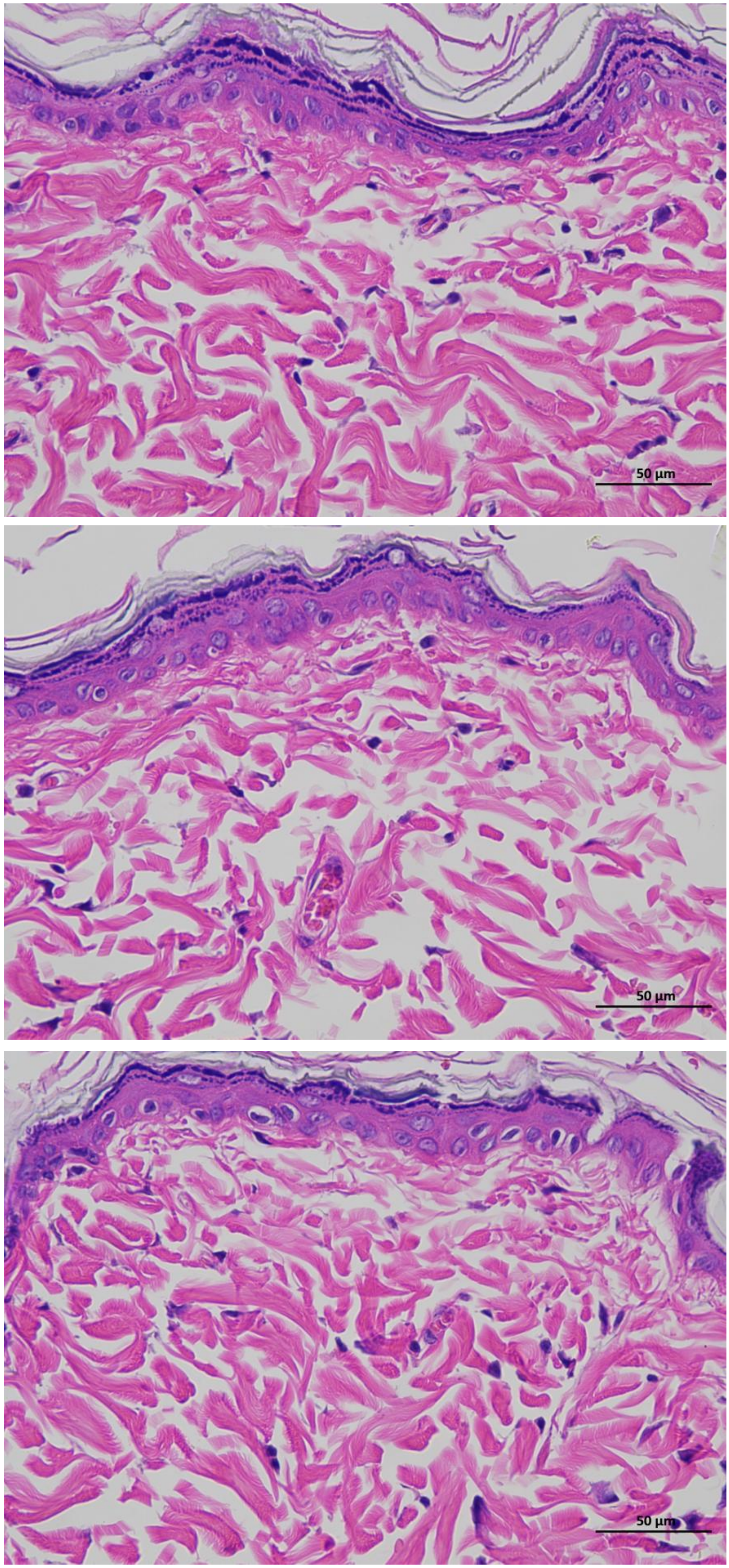

1 RNA-LNPs-L 14day 400-1

1 RNA-LNPs-L 14day 400-2

1 RNA-LNPs-L 14day 400-3

Animal 2

Supplementary Figure 3c | Representative H&E staining of skin tissue of RNA-LNPs-L animals / Day 14.

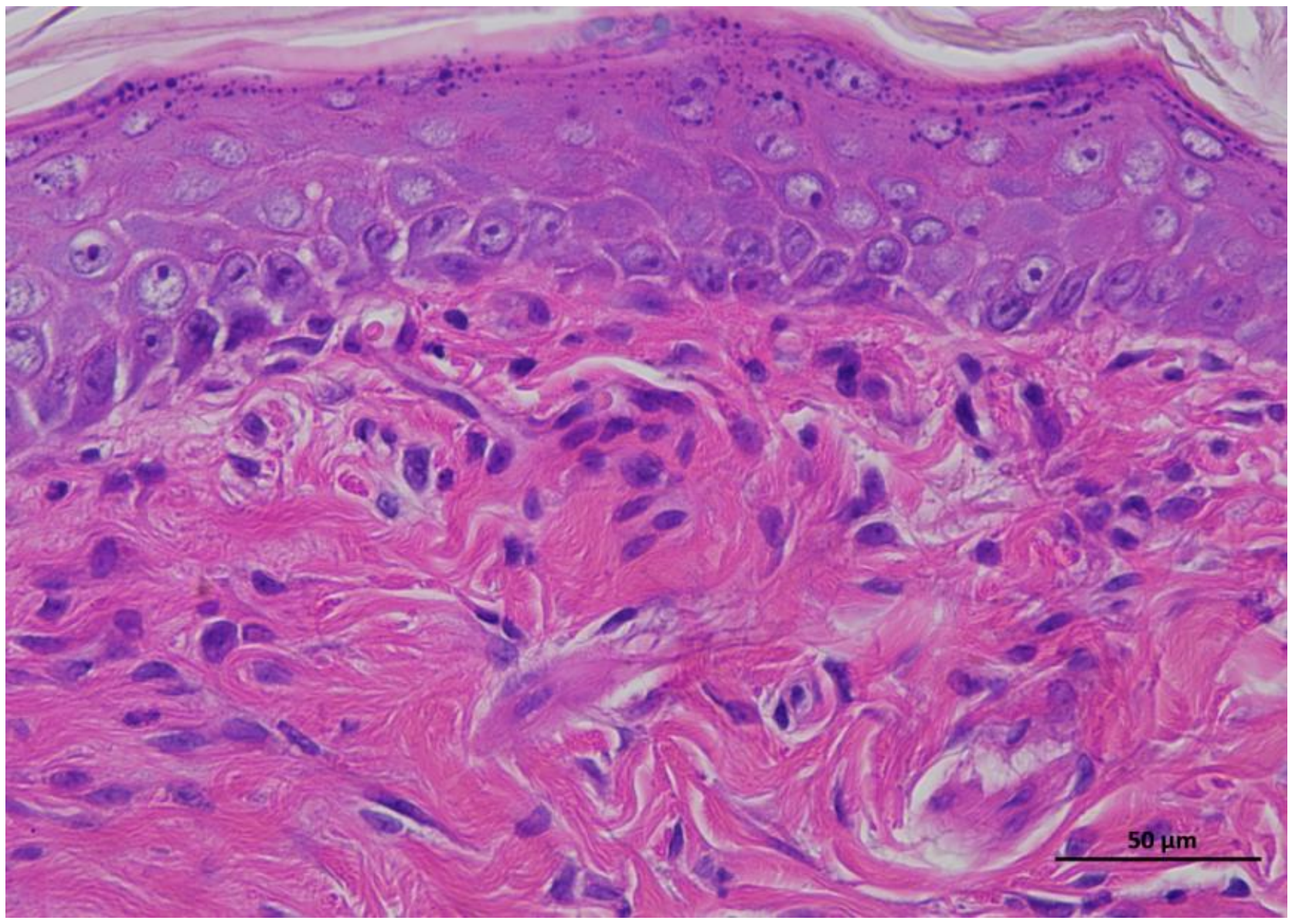

2 RNA-LNPs-L 14day 400-1

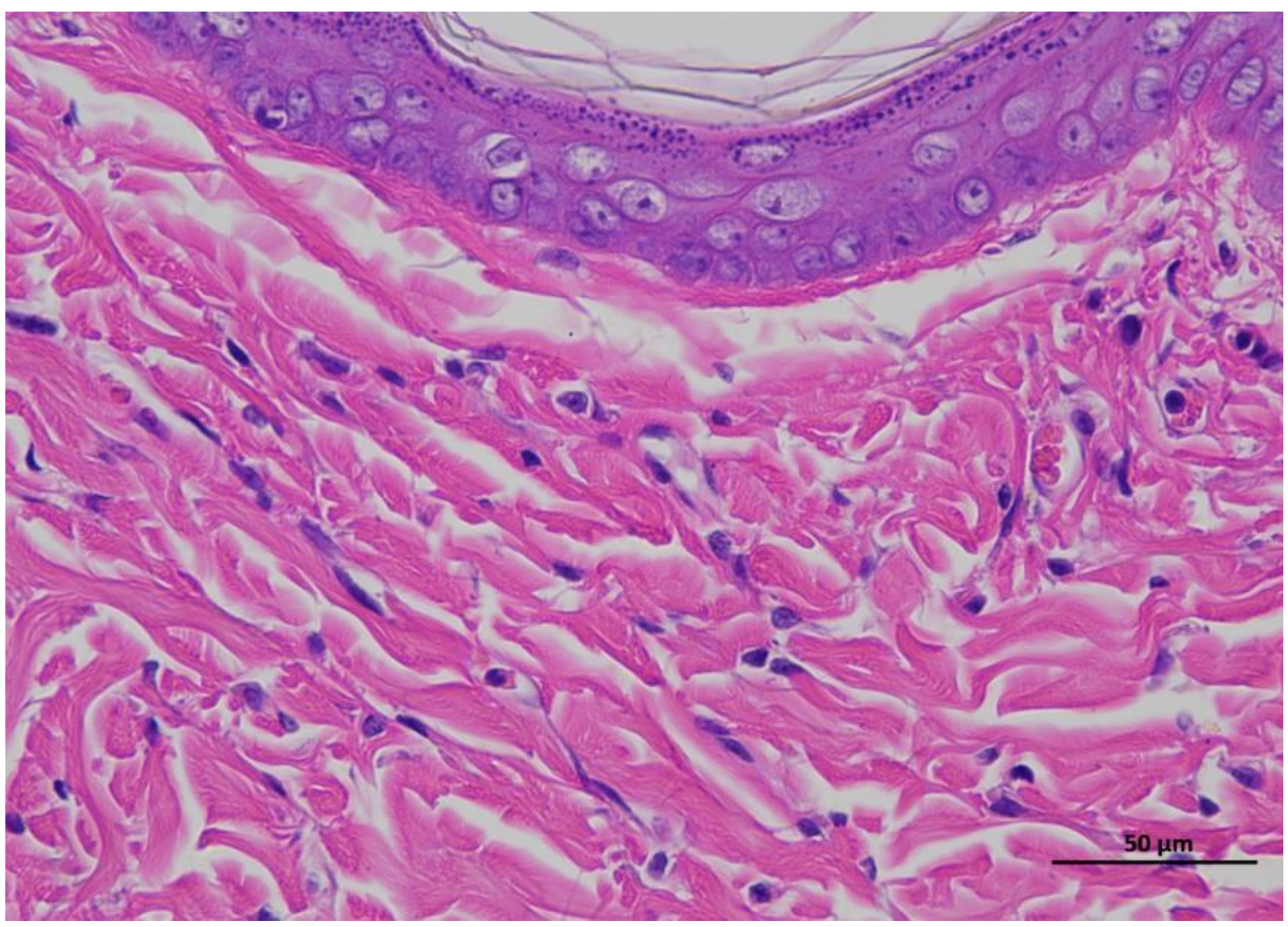

2 RNA-LNPs-L 14day 400-2

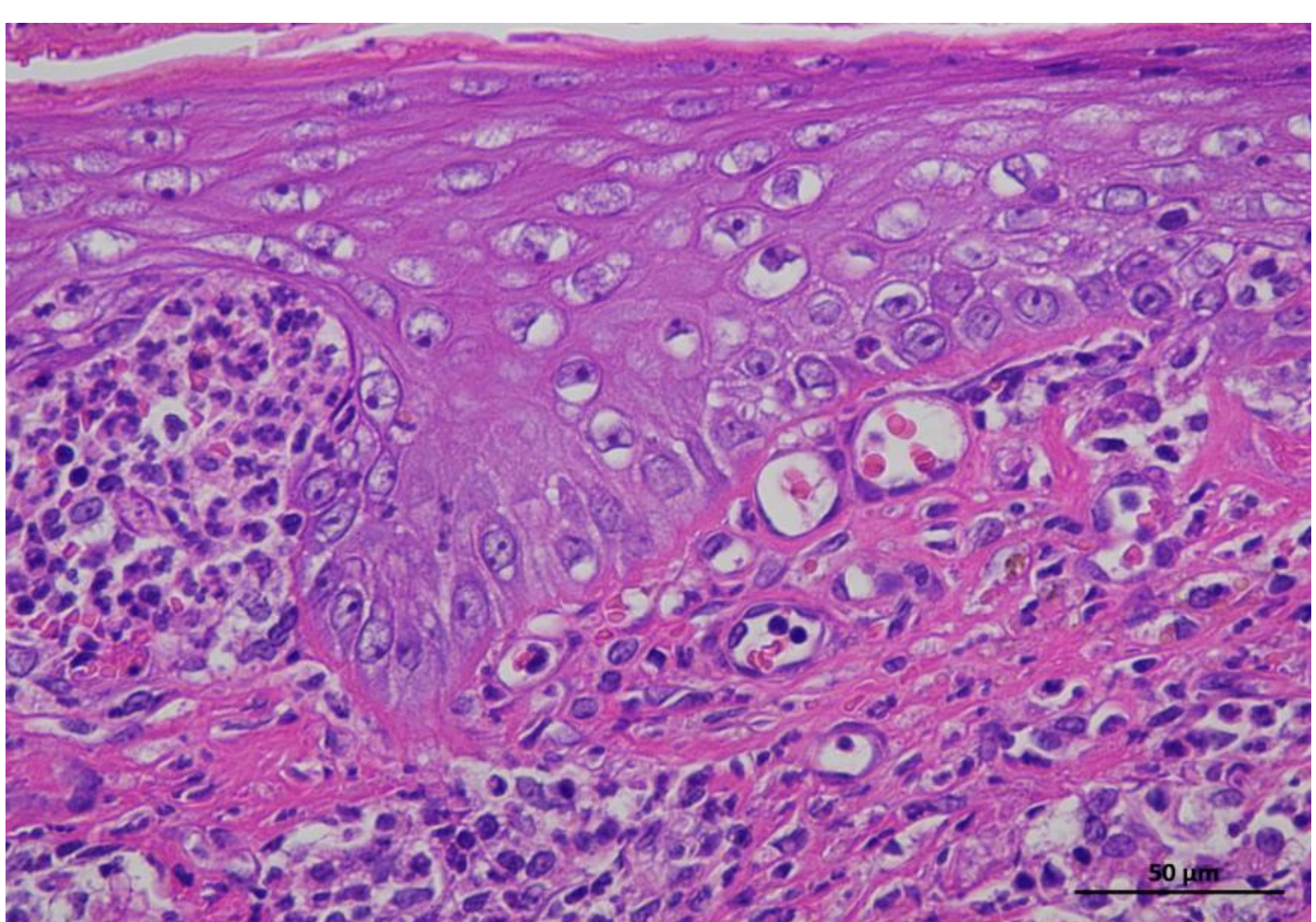

2 RNA-LNPs-L 14day 400-3

Animal 3

Supplementary Figure 3c | Representative H&E staining of skin tissue of RNA-LNPs-L animals / Day 14.

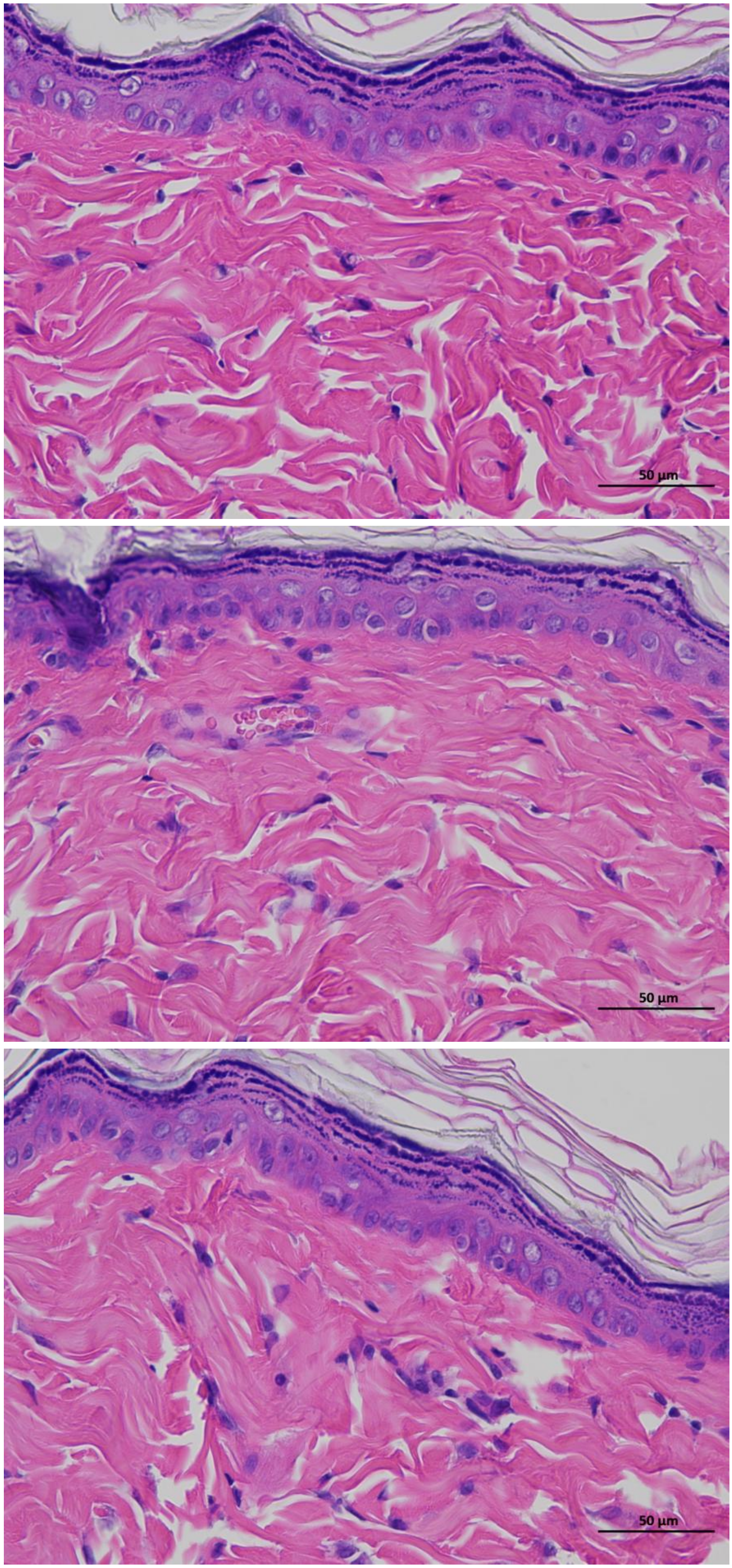

3 RNA-LNPs-L 14day 400-1

3 RNA-LNPs-L 14day 400-2

3 RNA-LNPs-L 14day 400-3

Supplementary Figure 3c | Representative H&E staining of skin tissue of RNA-LNPs-L animals / Day 14.

Animal 4

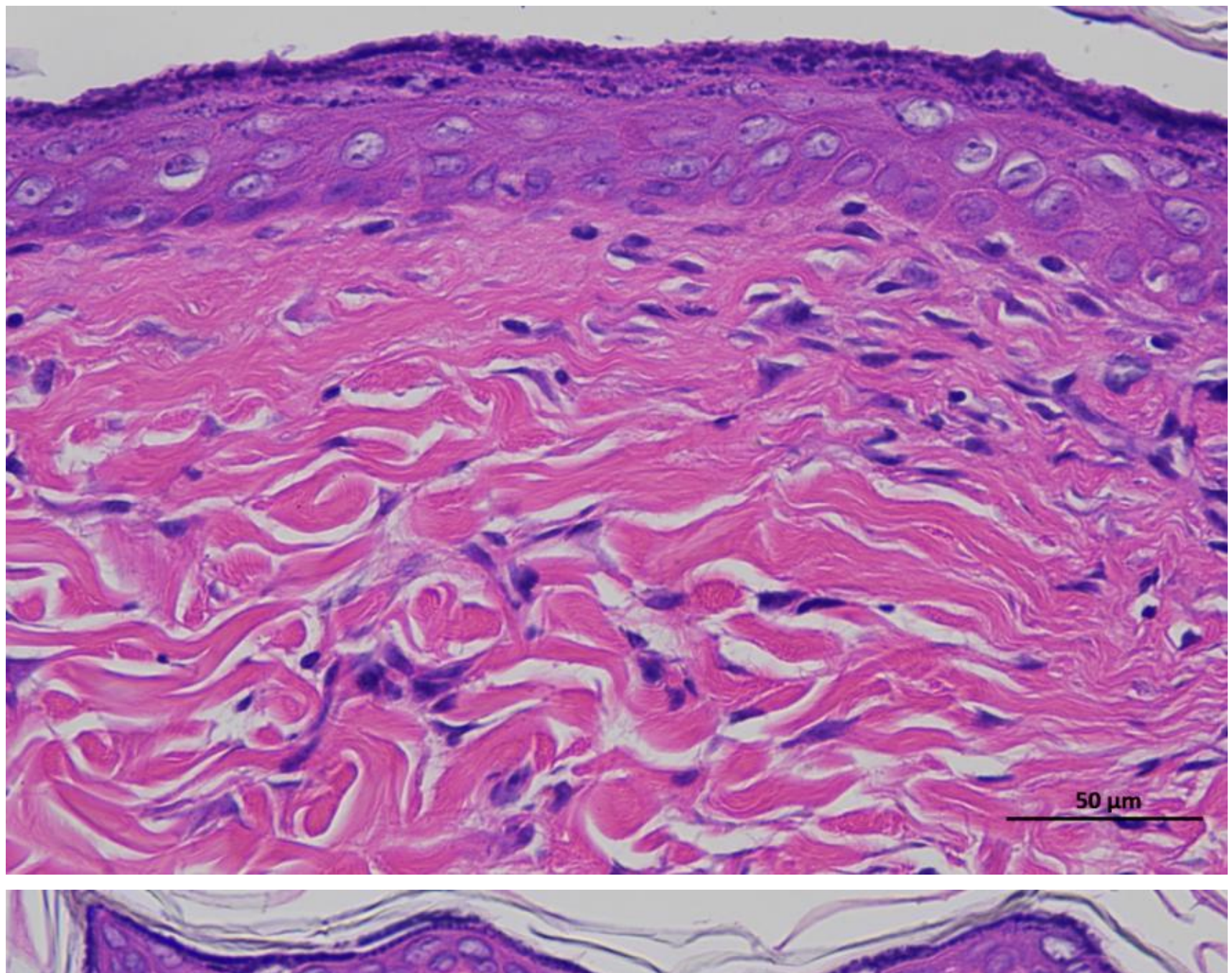

4 RNA-LNPs-L 14day 400-1

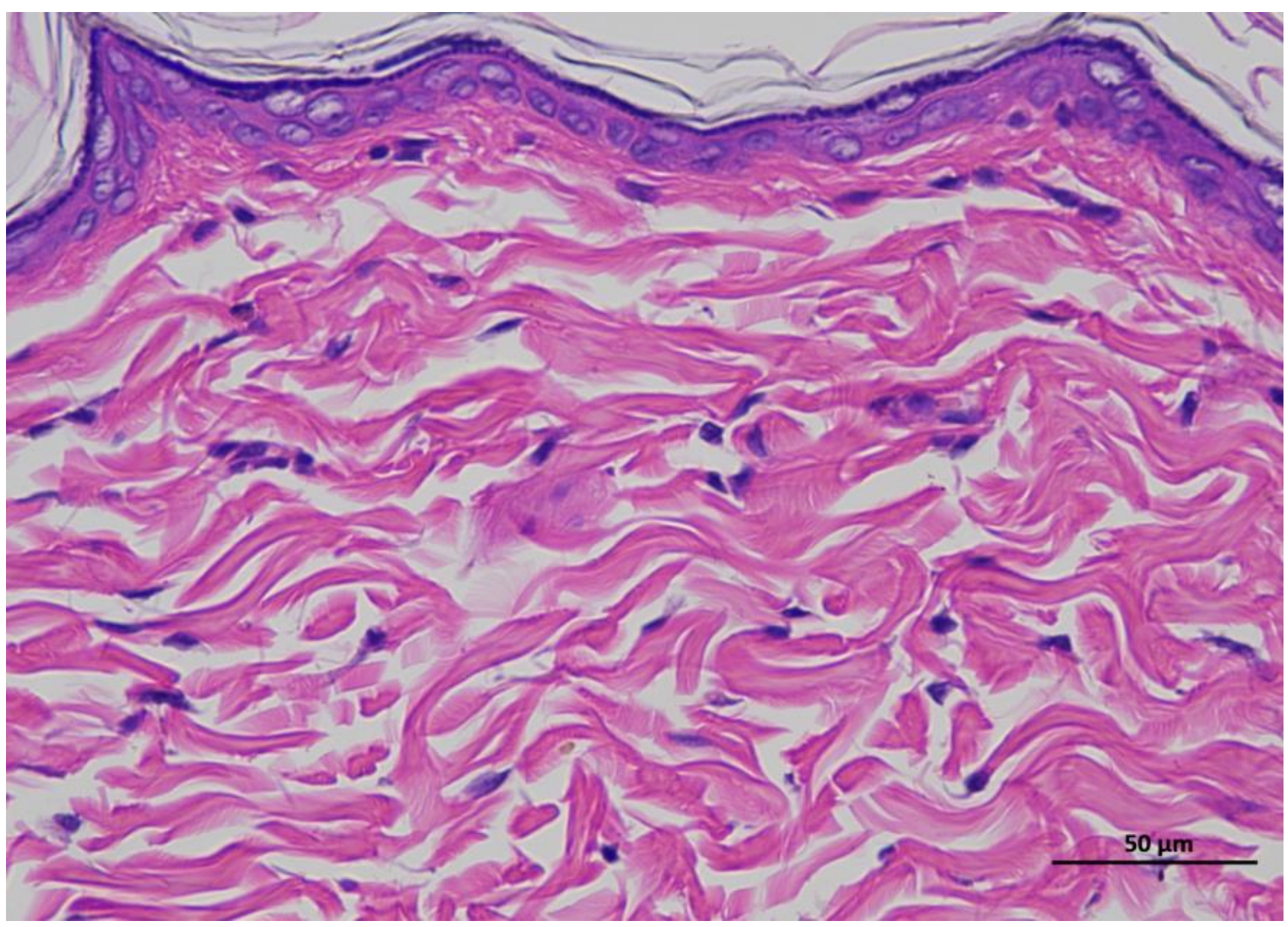

4 RNA-LNPs-L 14day 400-2

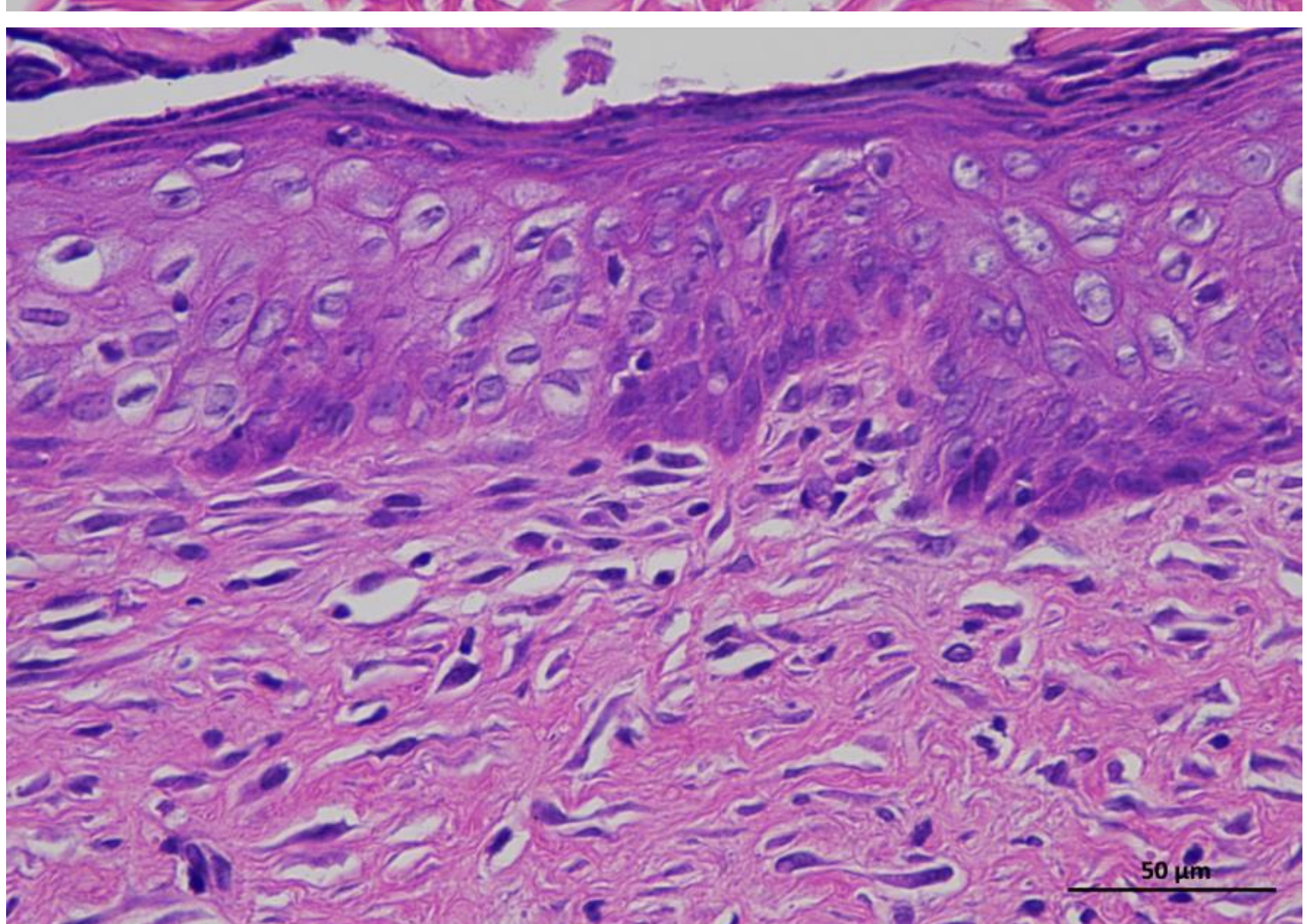

4 RNA-LNPs-L 14day 400-3

Animal 5

Supplementary Figure 3c | Representative H&E staining of skin tissue of RNA-LNPs-L animals / Day 14.

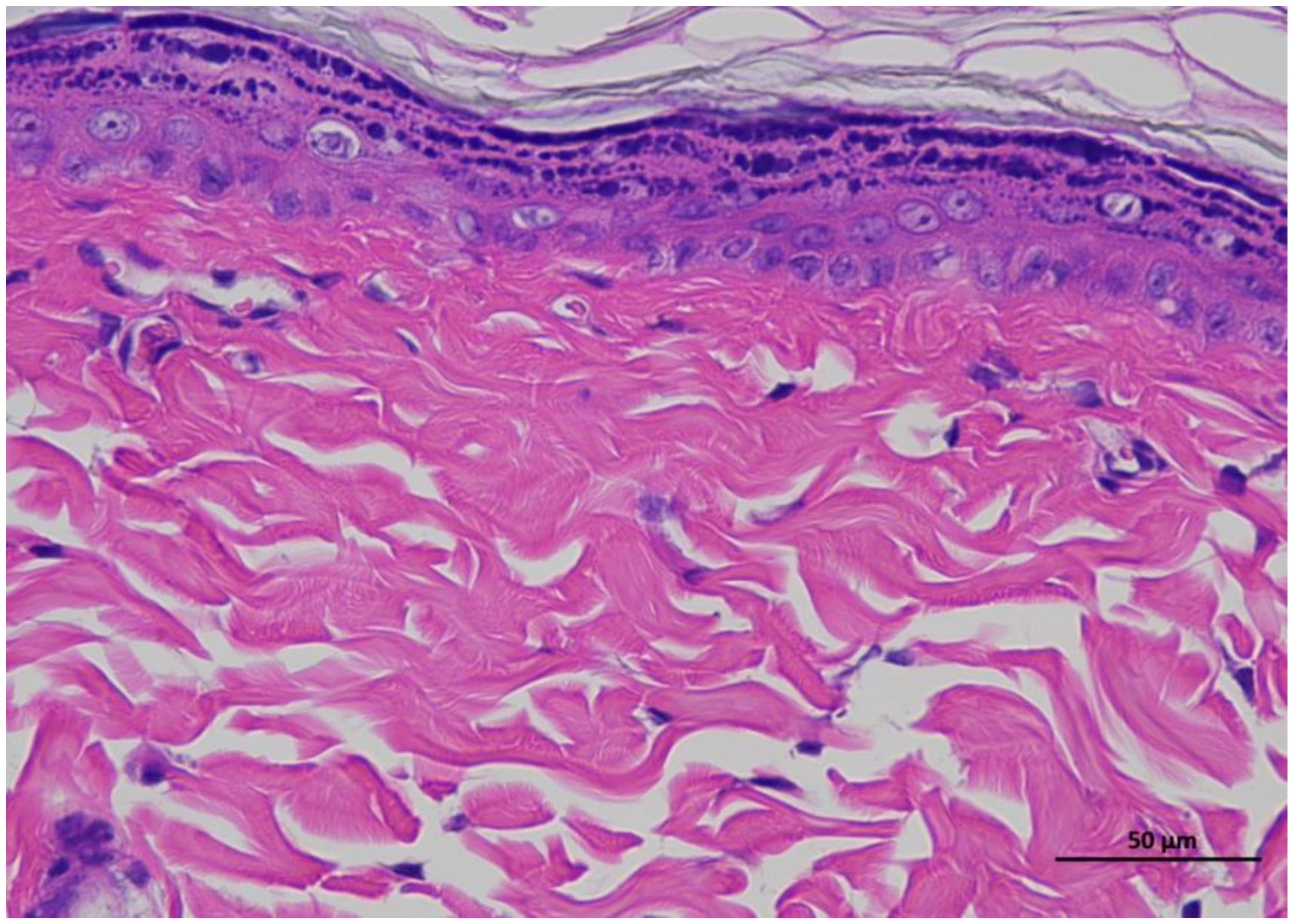

5 RNA-LNPs-L 14day 400-1

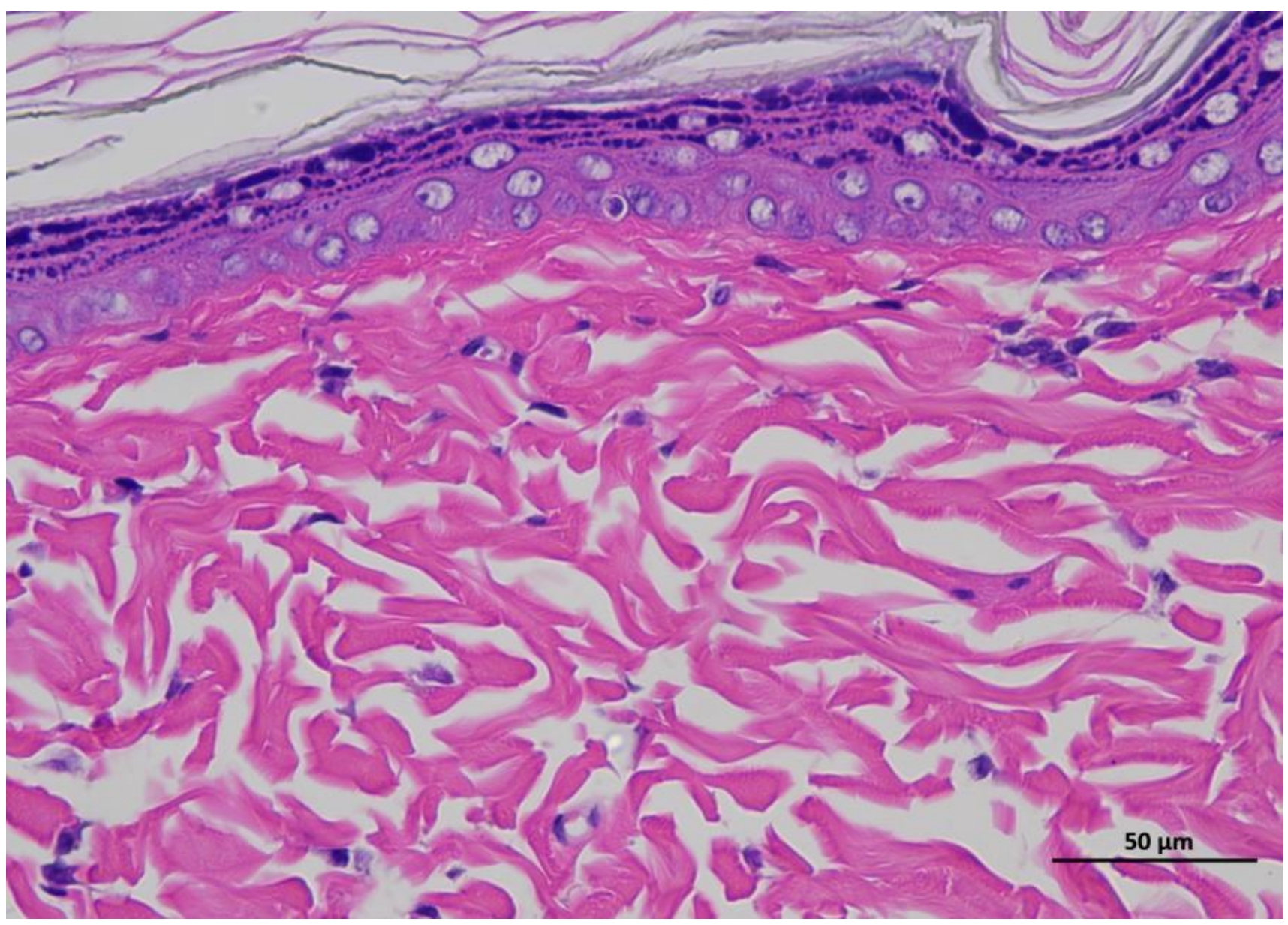

5 RNA-LNPs-L 14day 400-2

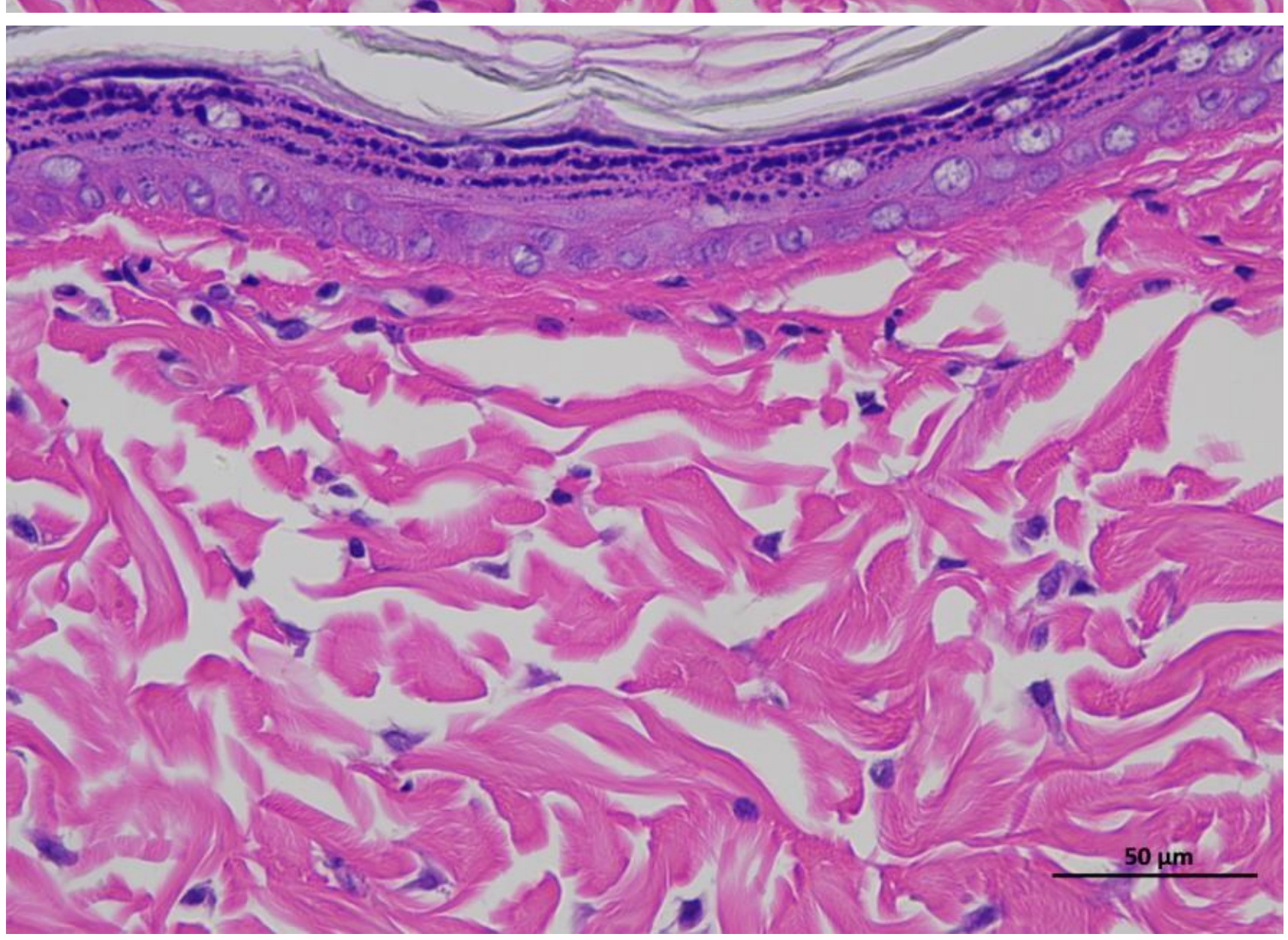

5 RNA-LNPs-L 14day 400-3

Supplementary Figure 3c | Representative H&E staining of skin tissue of RNA-LNPs-L animals / Day 14.

Animal 6

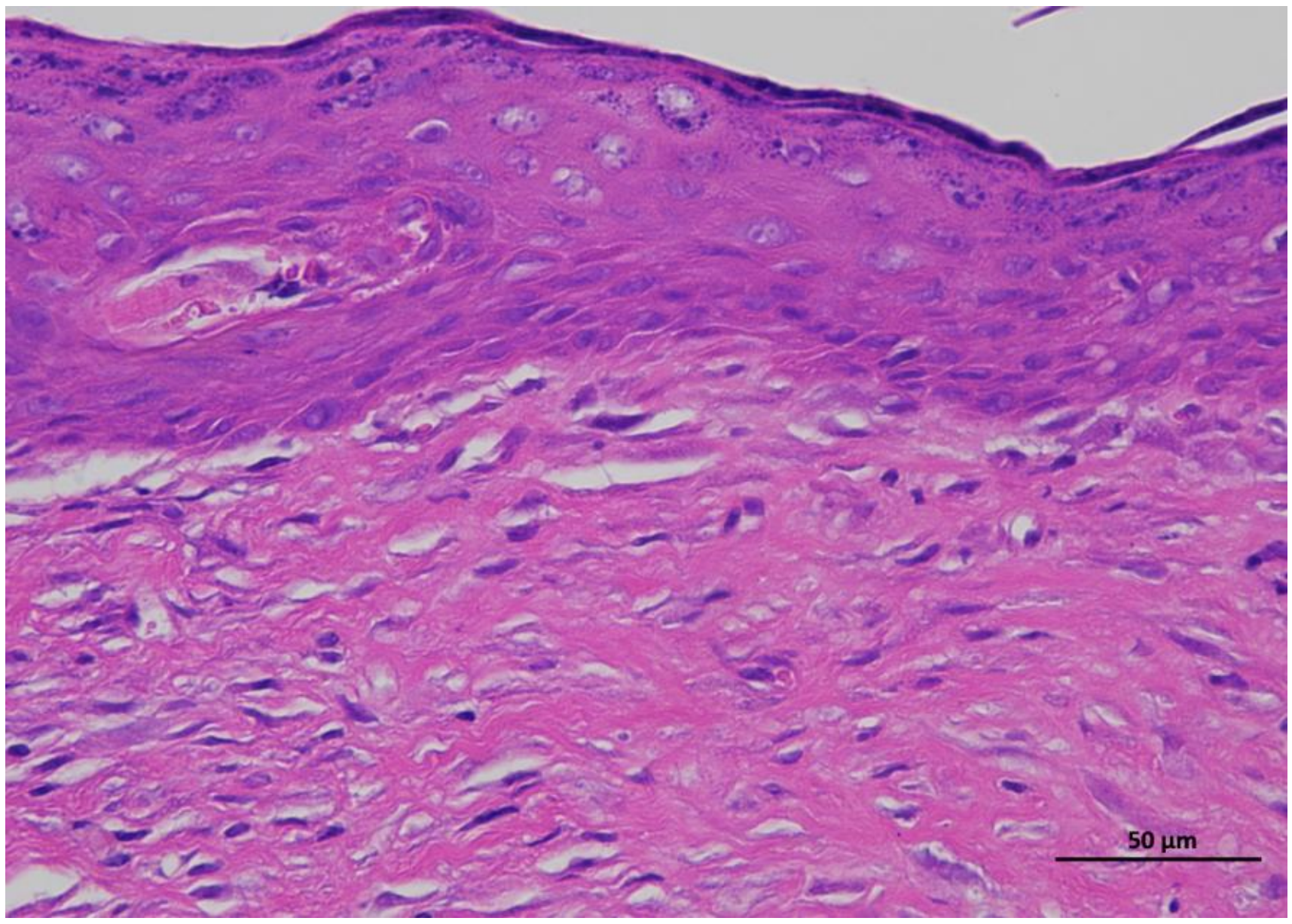

6 RNA-LNPs-L 14day 400-1

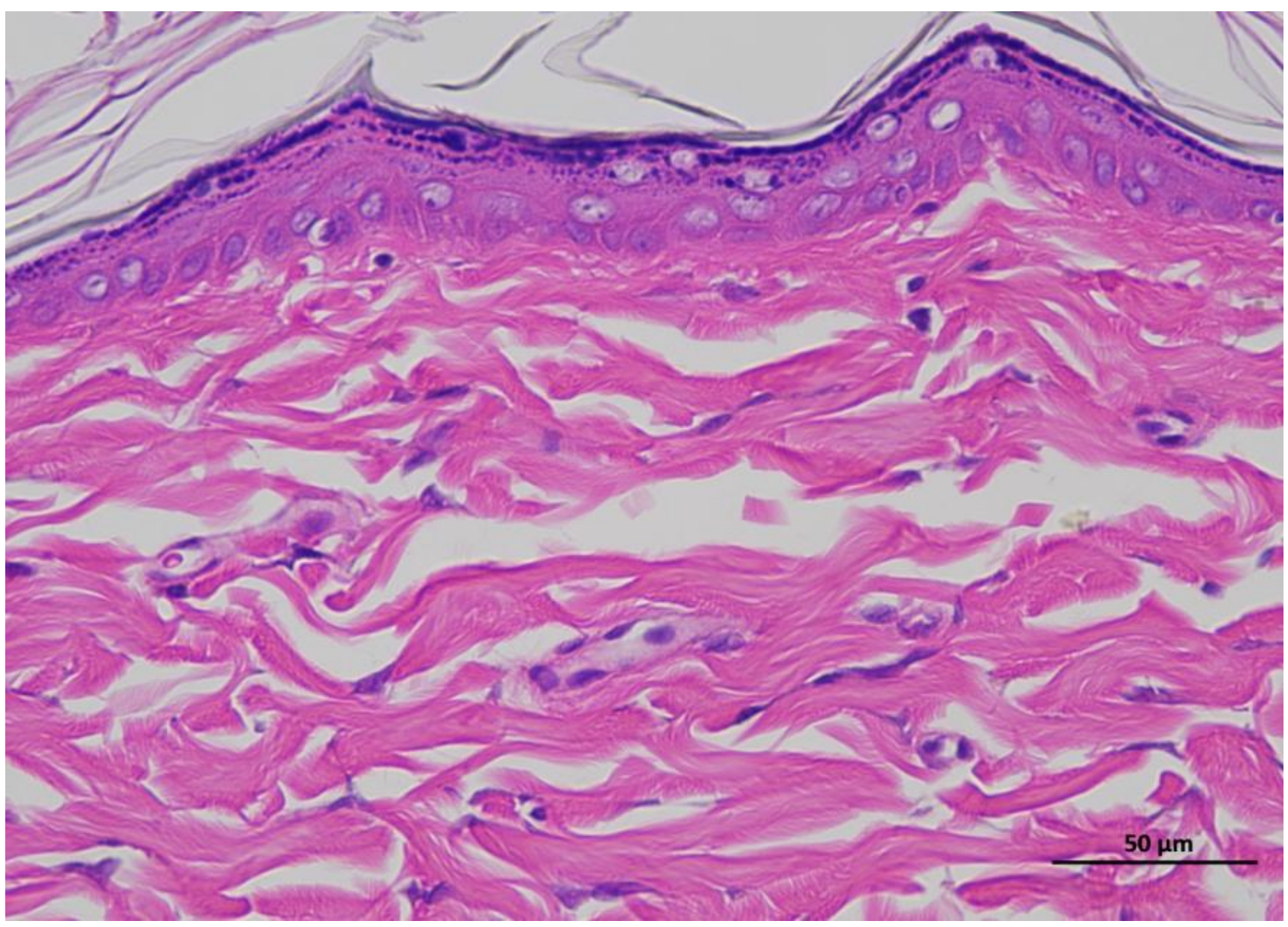

6 RNA-LNPs-L 14day 400-2

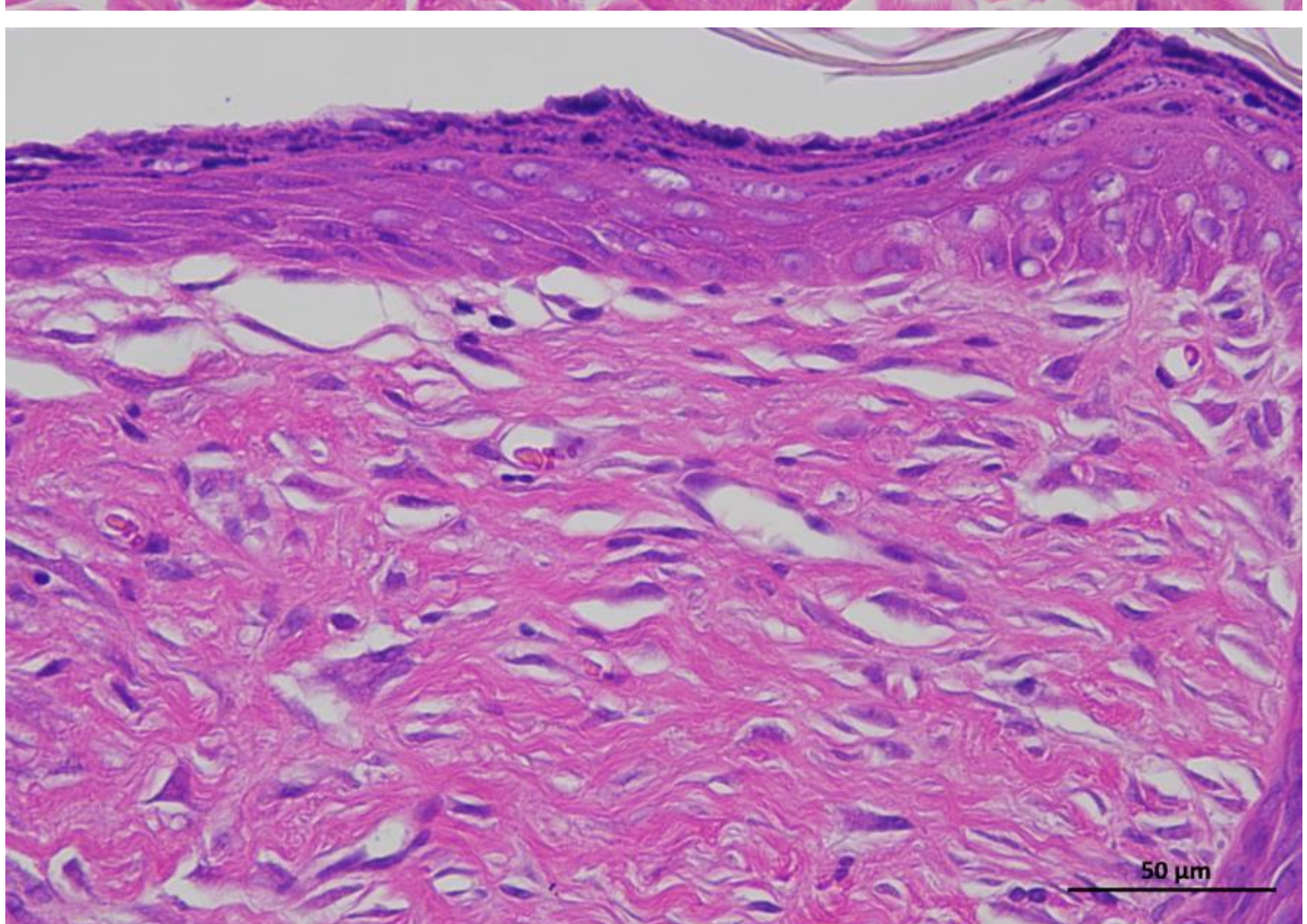

6 RNA-LNPs-L 14day 400-3

Supplementary Figure 2d | Representative H&E staining of skin tissue of RNA-LNPs-H animals / Day 0.
Representative hematoxylin and eosin (H&E)–stained section of skin tissue collected from experimental rat. The image shows the typical architecture of the skin, including the epidermal layer with stratified keratinized epithelium and the underlying dermis composed of dense collagen fibers and connective tissue structures.
(n = 6 per group).

Animal 1

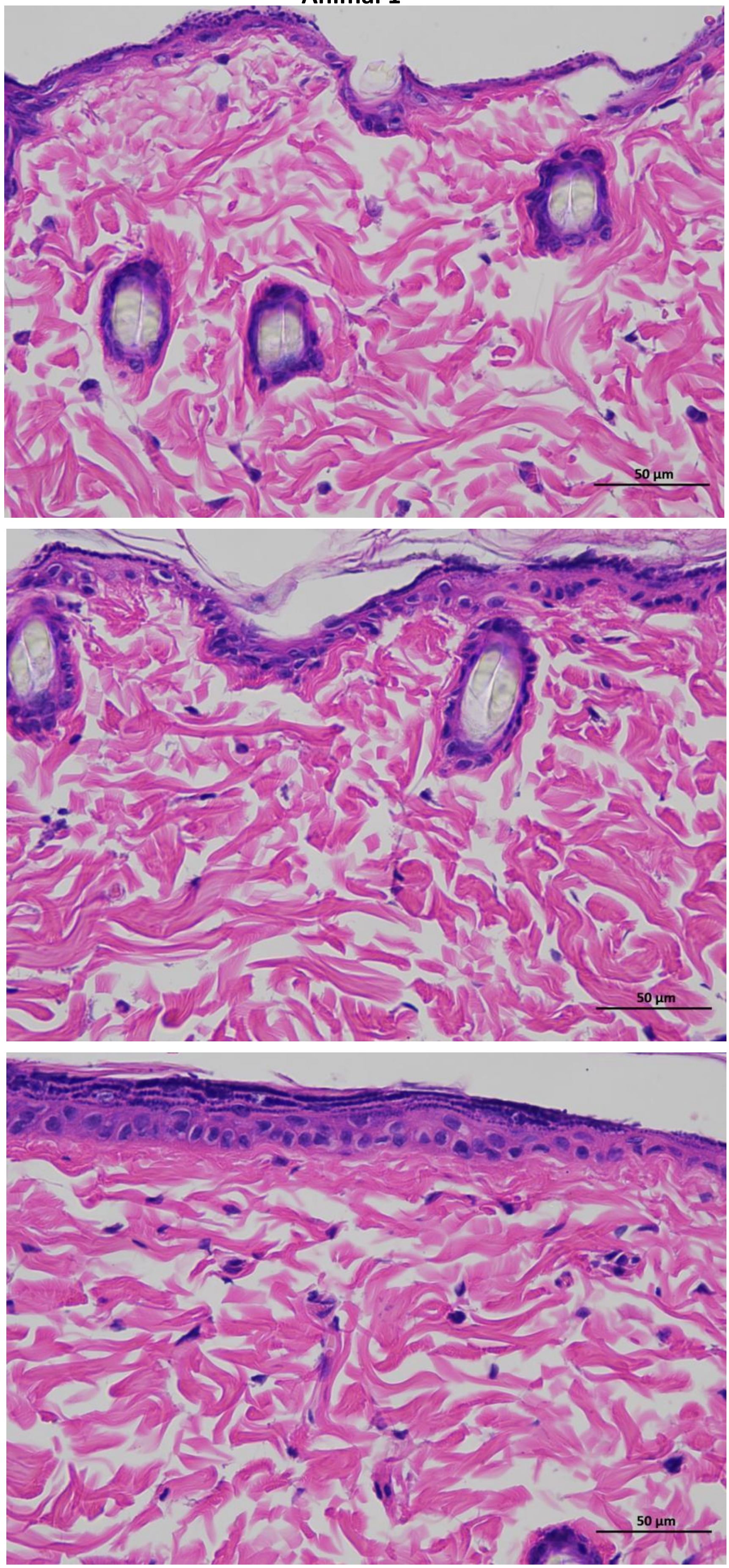

1 RNA-LNPs-H 0day 400-1

1 RNA-LNPs-H 0day 400-2

1 RNA-LNPs-H 0day 400-3

Animal 2

Supplementary Figure 2d| Representative H&I staining of skin tissue of RNA-LNPs-H animals / Day 0.

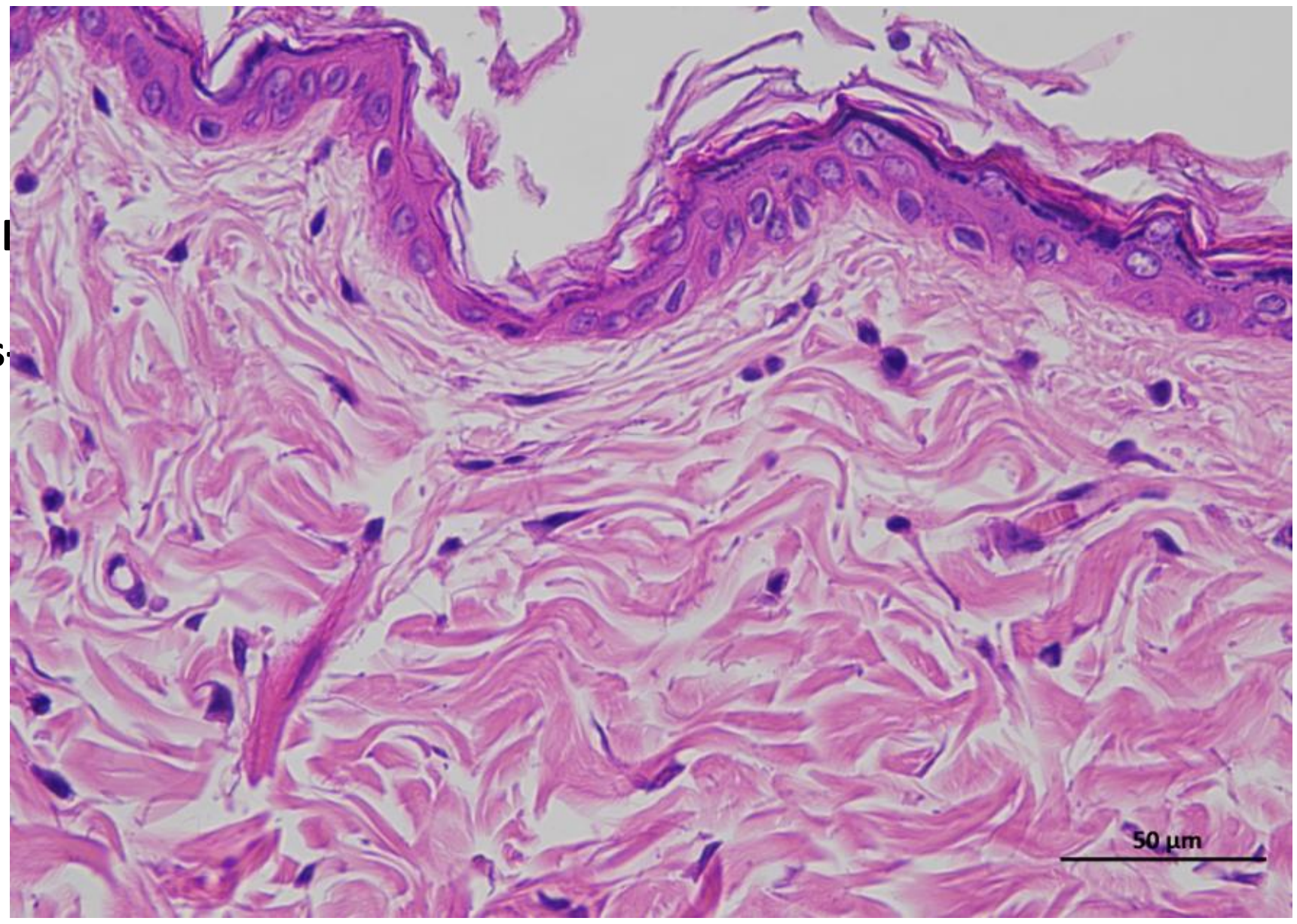

2 RNA-LNPs-H 0day 400-1

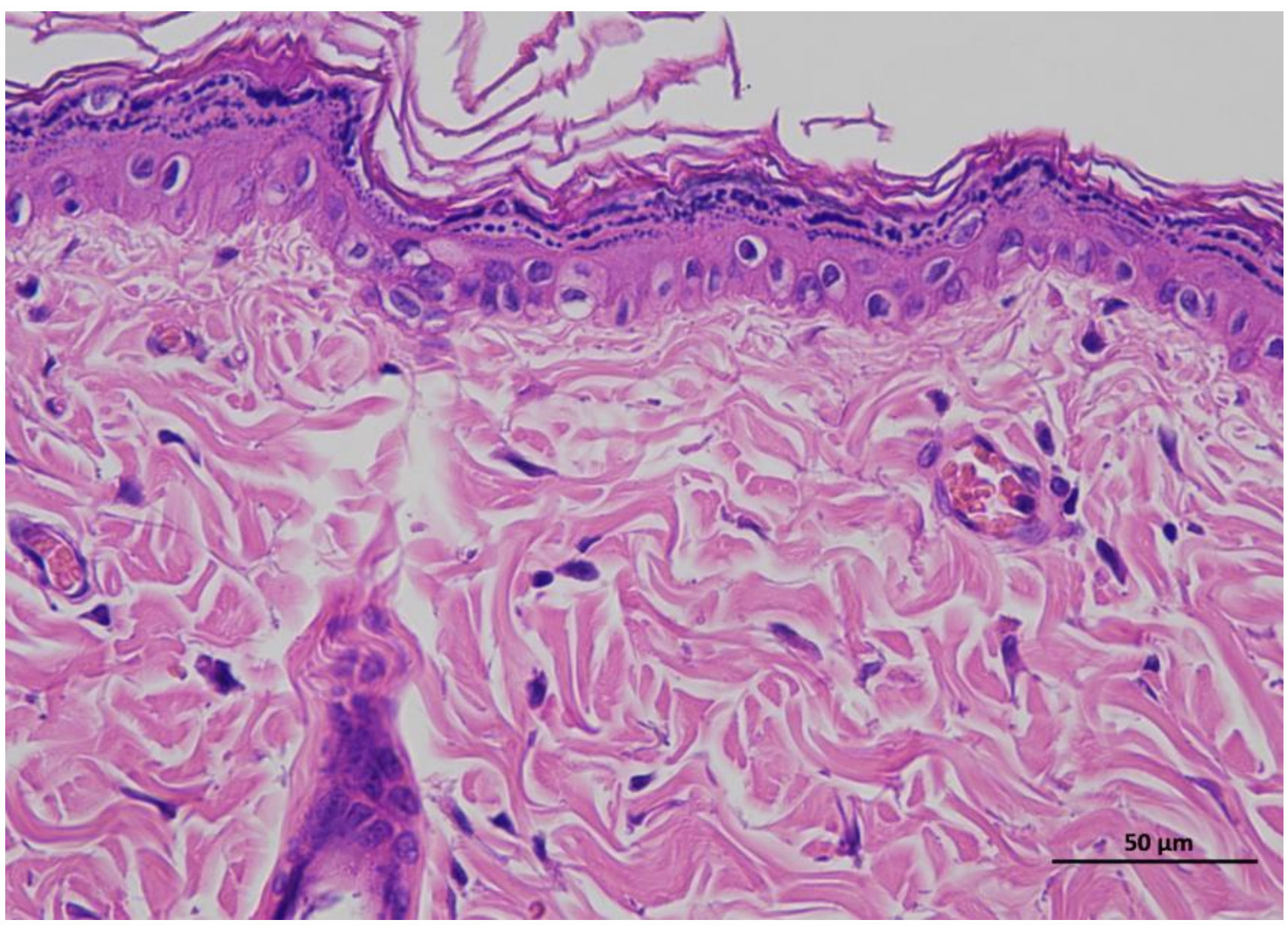

2 RNA-LNPs-H 0day 400-2

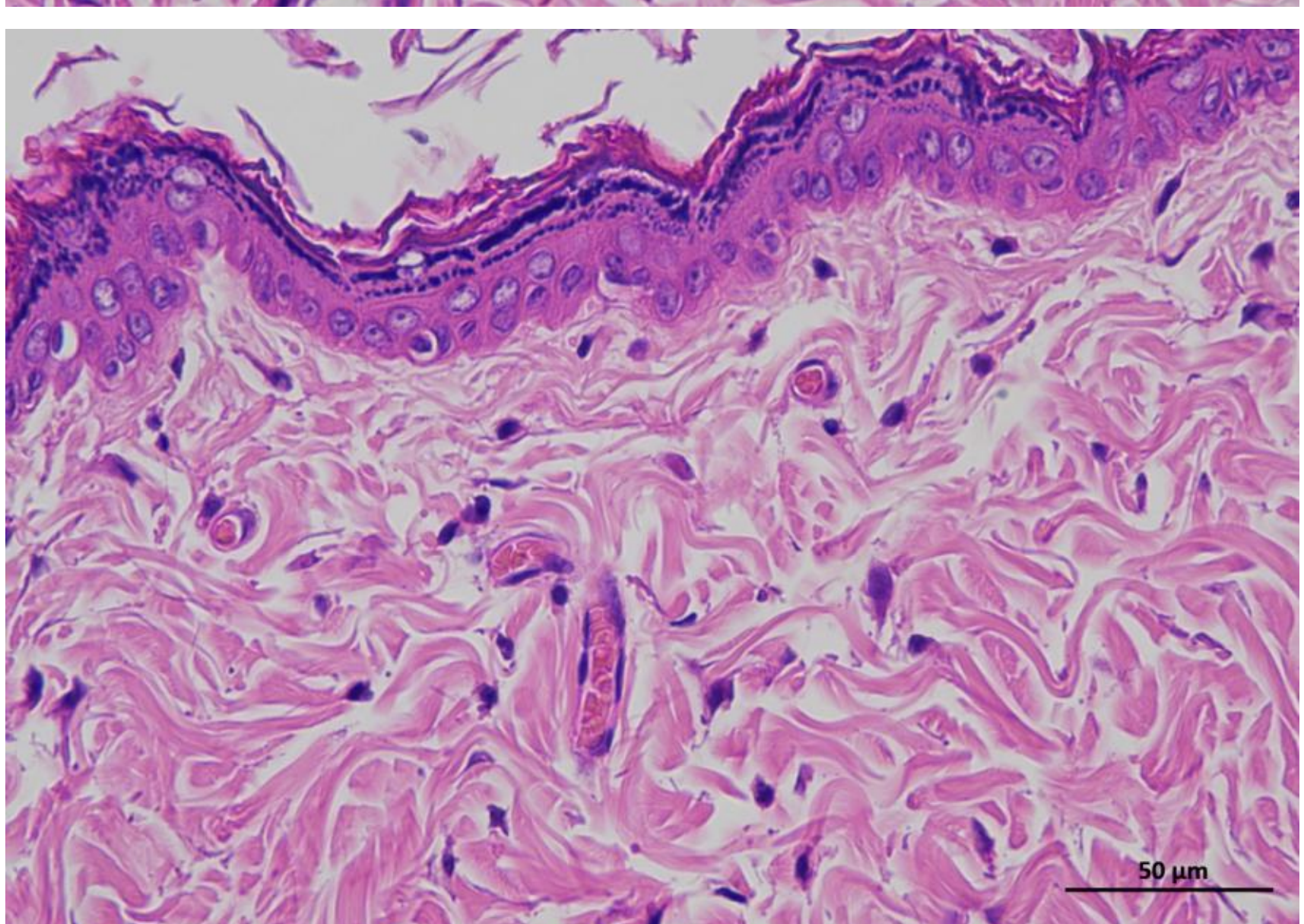

2 RNA-LNPs-H 0day 400-3

Animal 3

Supplementary Figure 2d| Representative H&E staining of skin tissue of RNA-LNPs-H animals / Day 0.

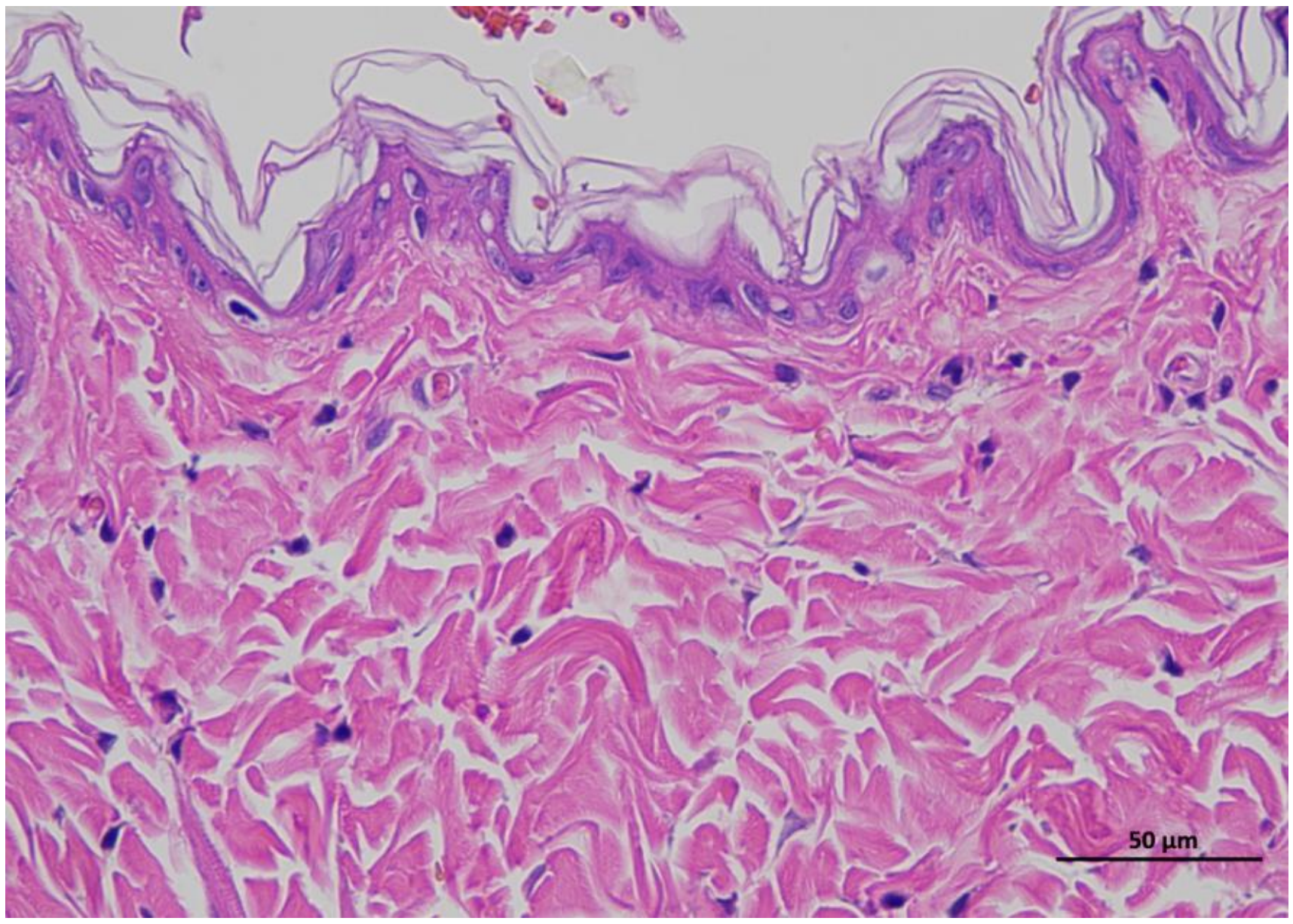

3 RNA-LNPs-H 0day 400-1

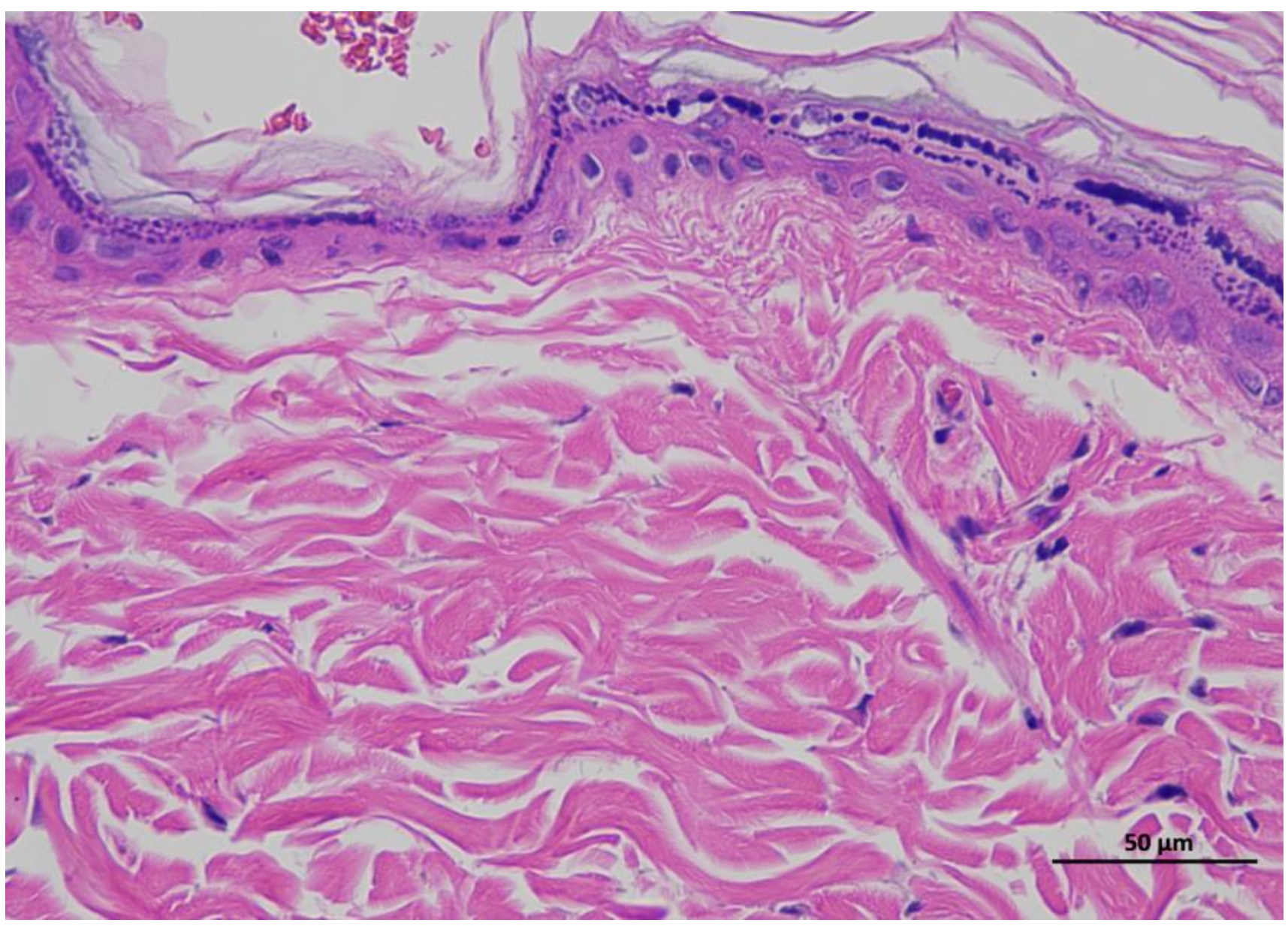

3 RNA-LNPs-H 0day 400-2

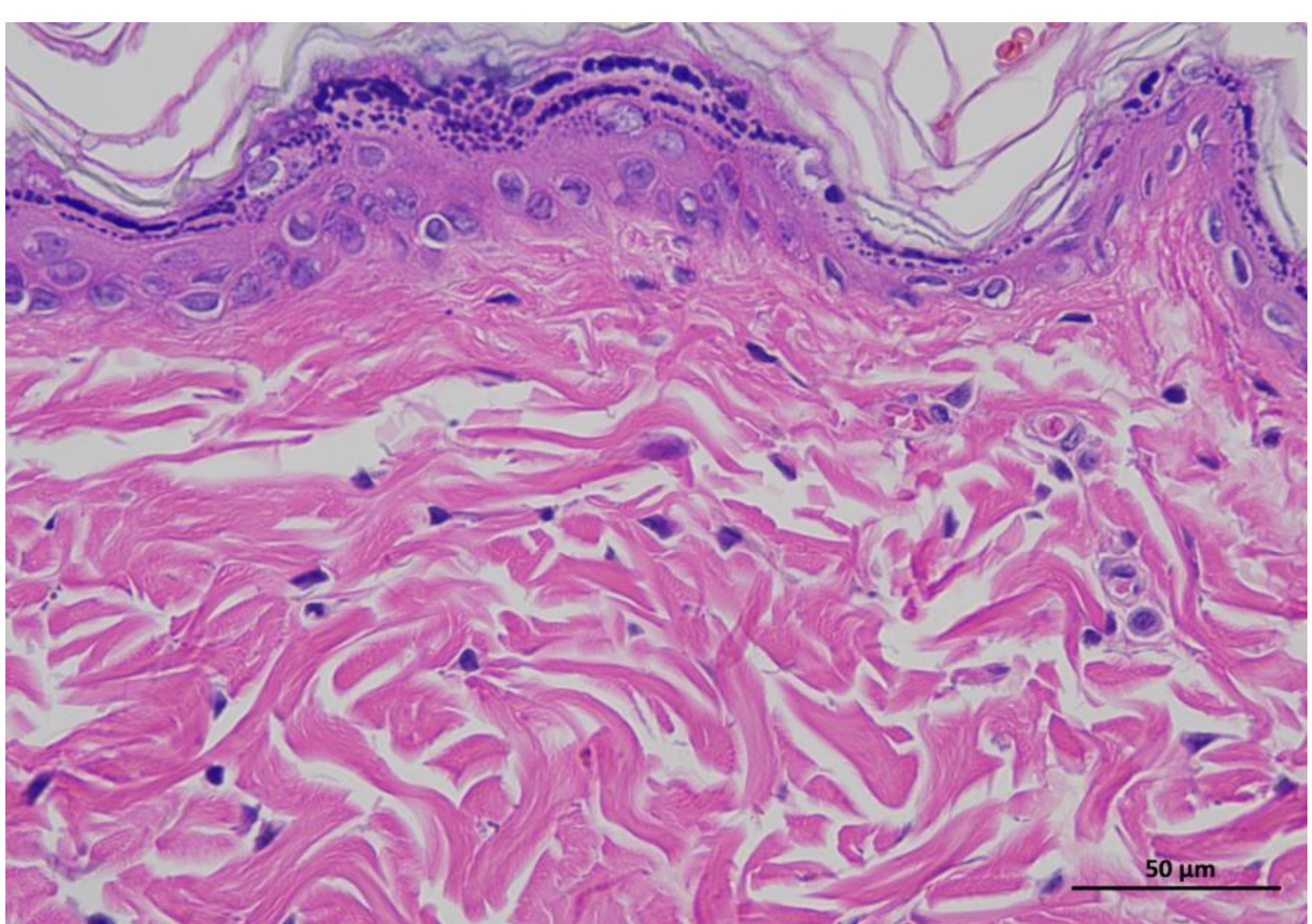

3 RNA-LNPs-H 0day 400-3

Animal 4

Supplementary Figure 2d| Representative H&E staining of skin tissue of RNA-LNPs-H animals / Day 0.

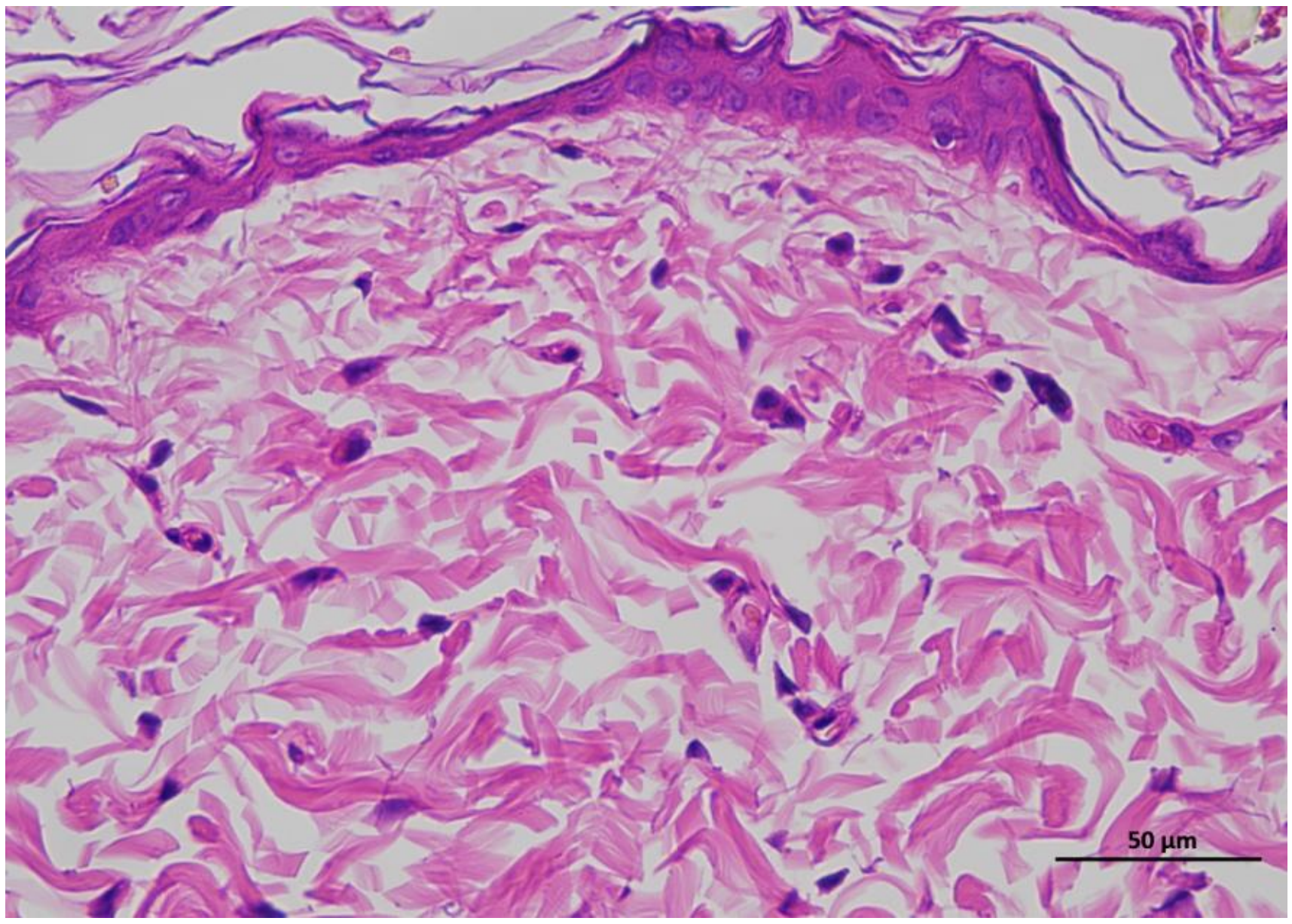

4 RNA-LNPs-H 0day 400-1

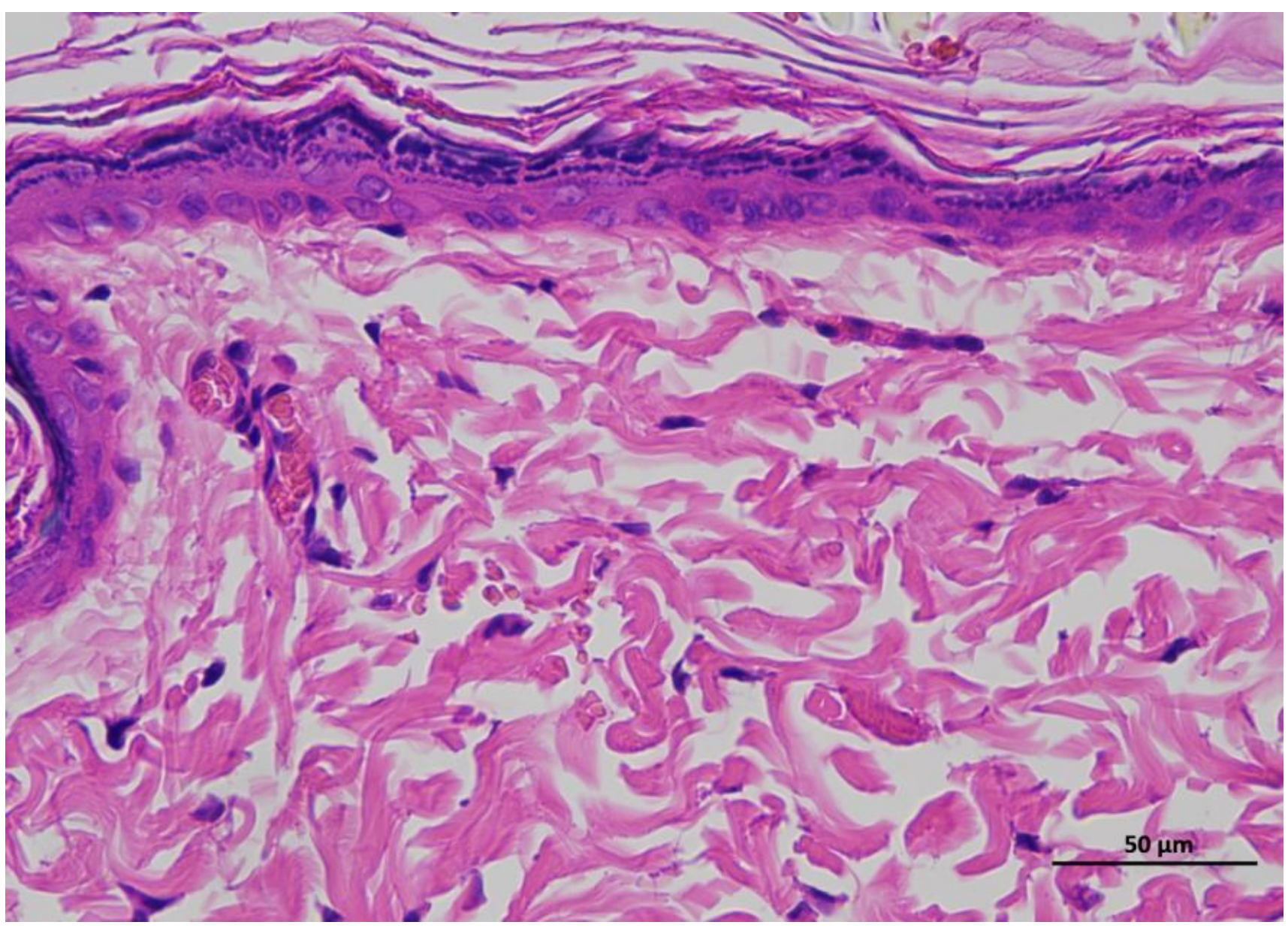

4 RNA-LNPs-H 0day 400-2

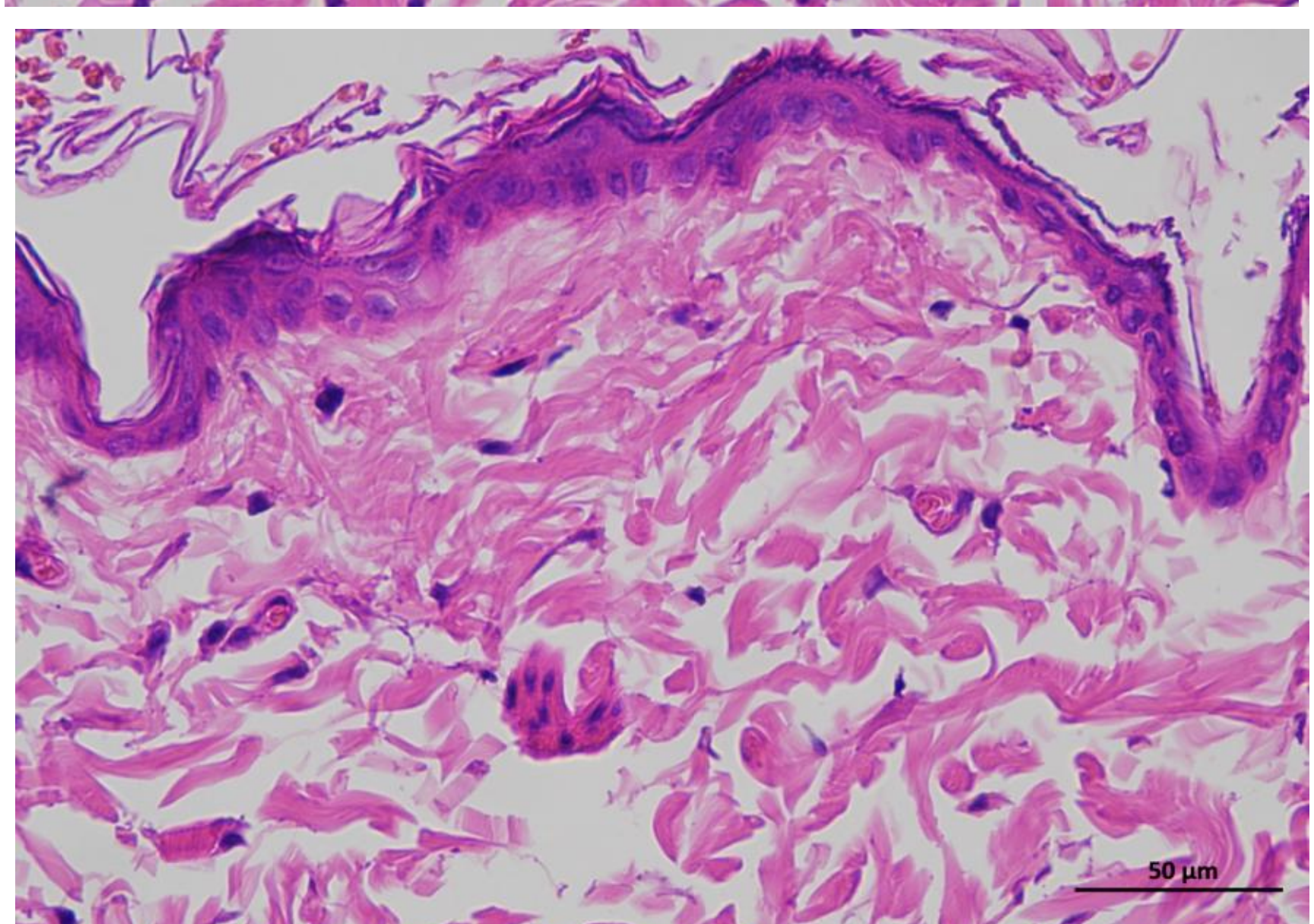

4 RNA-LNPs-H 0day 400-3

Animal 5

Supplementary Figure 2d| Representative H&E staining of skin tissue of RNA-LNPs-H animals / Day 0.

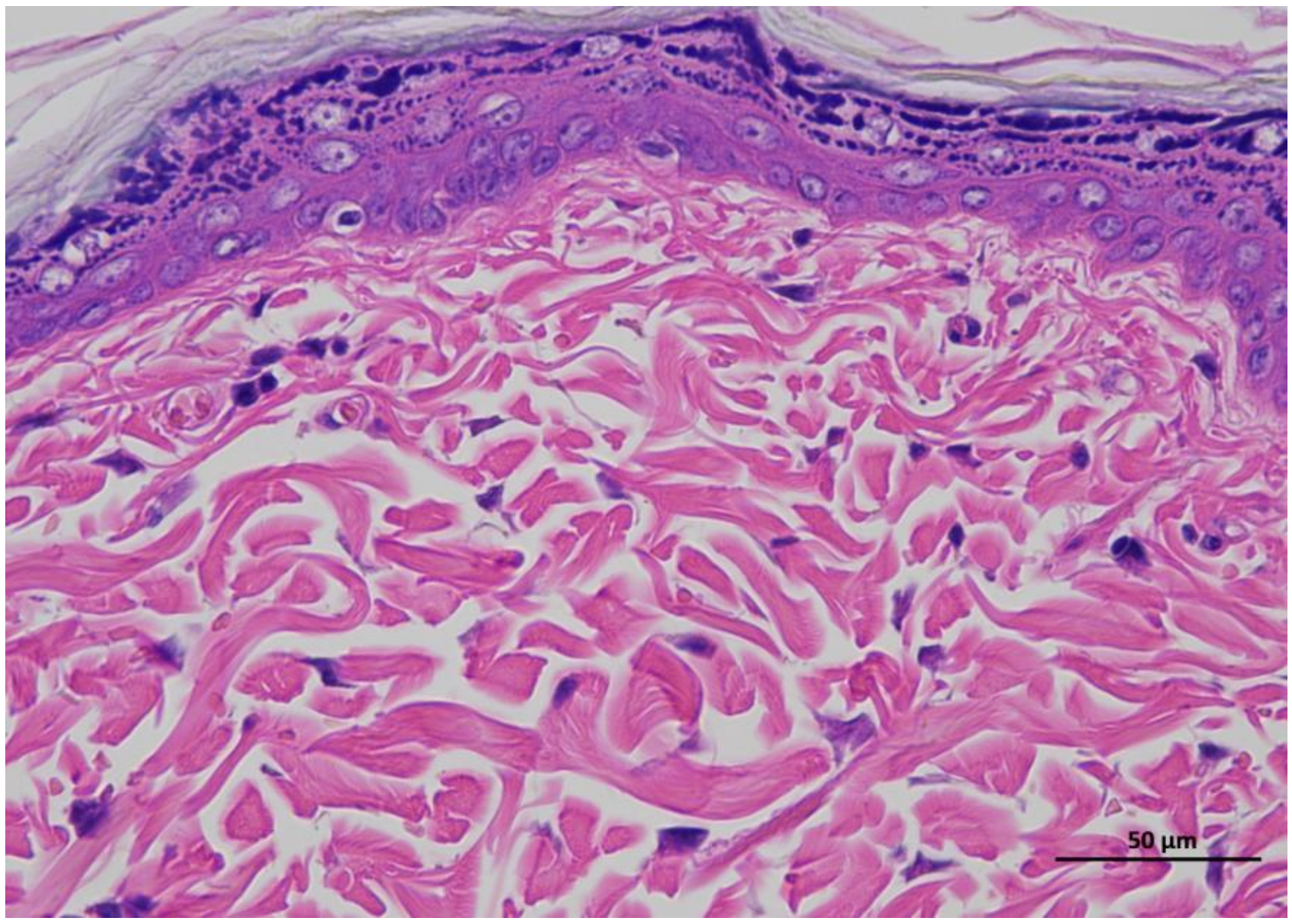

5 RNA-LNPs-H 0day 400-1

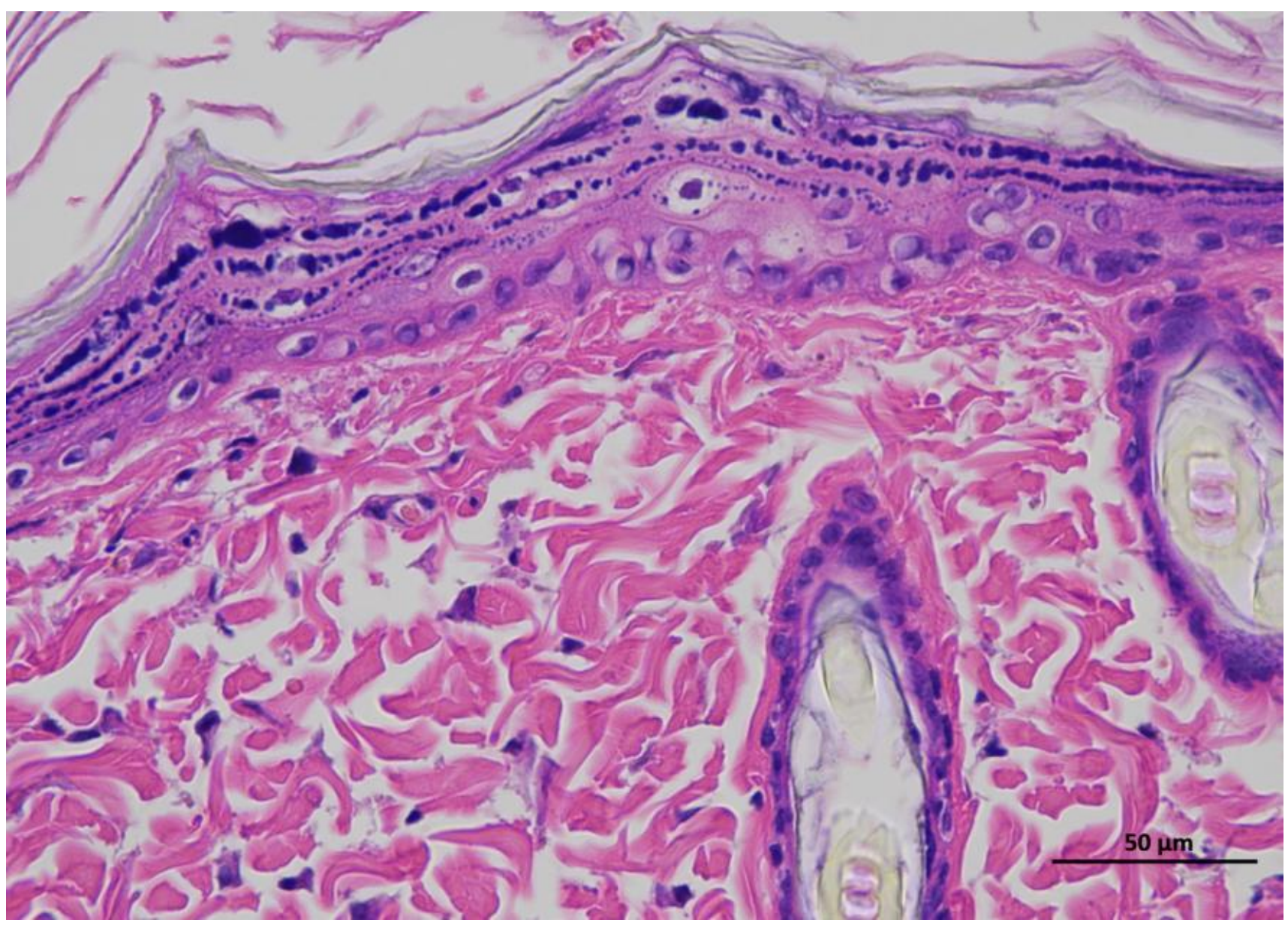

5 RNA-LNPs-H 0day 400-2

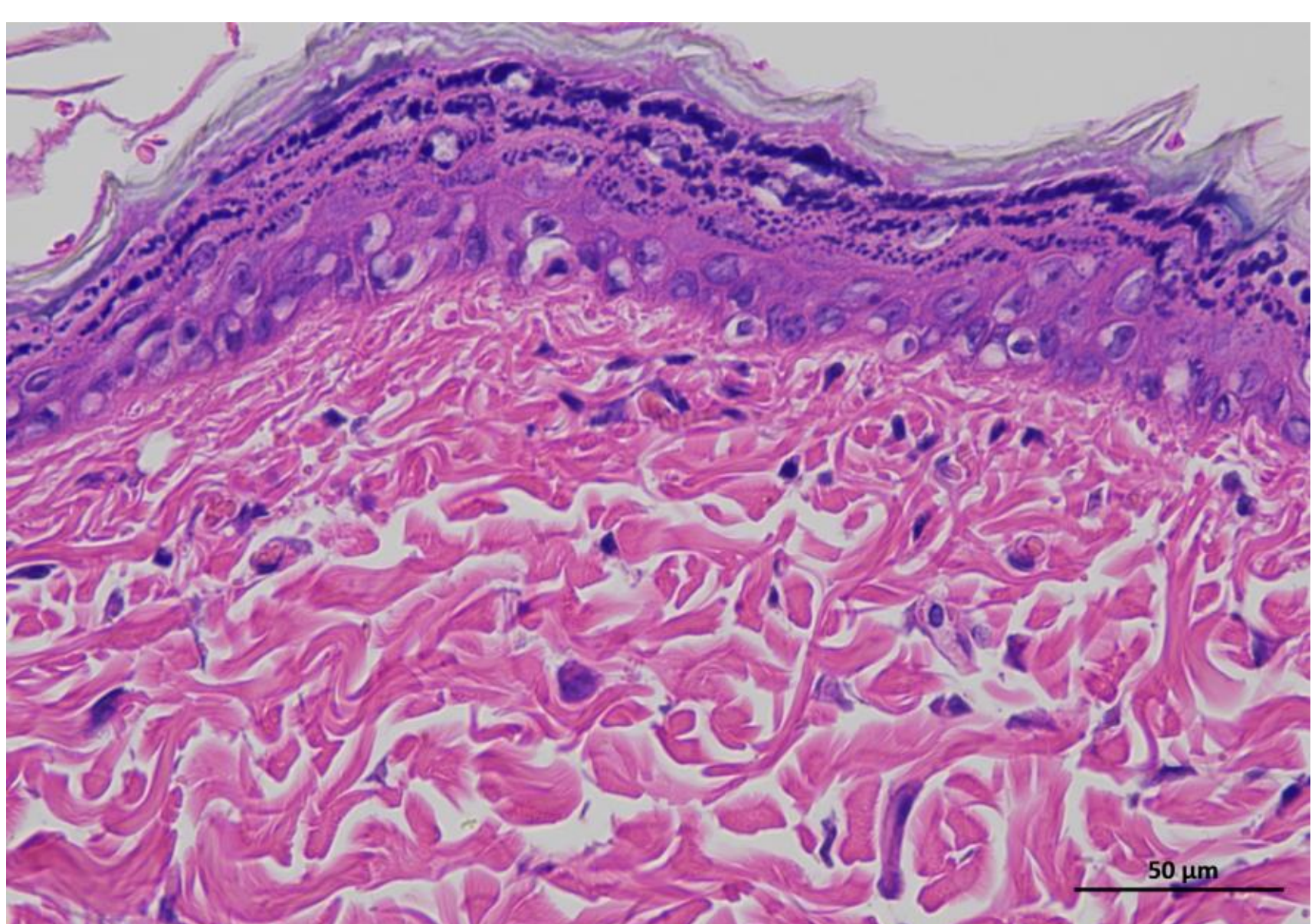

5 RNA-LNPs-H 0day 400-3

Animal 6

Supplementary Figure 2d| Representative H&E staining of skin tissue of RNA-LNPs-H animals / Day 0.

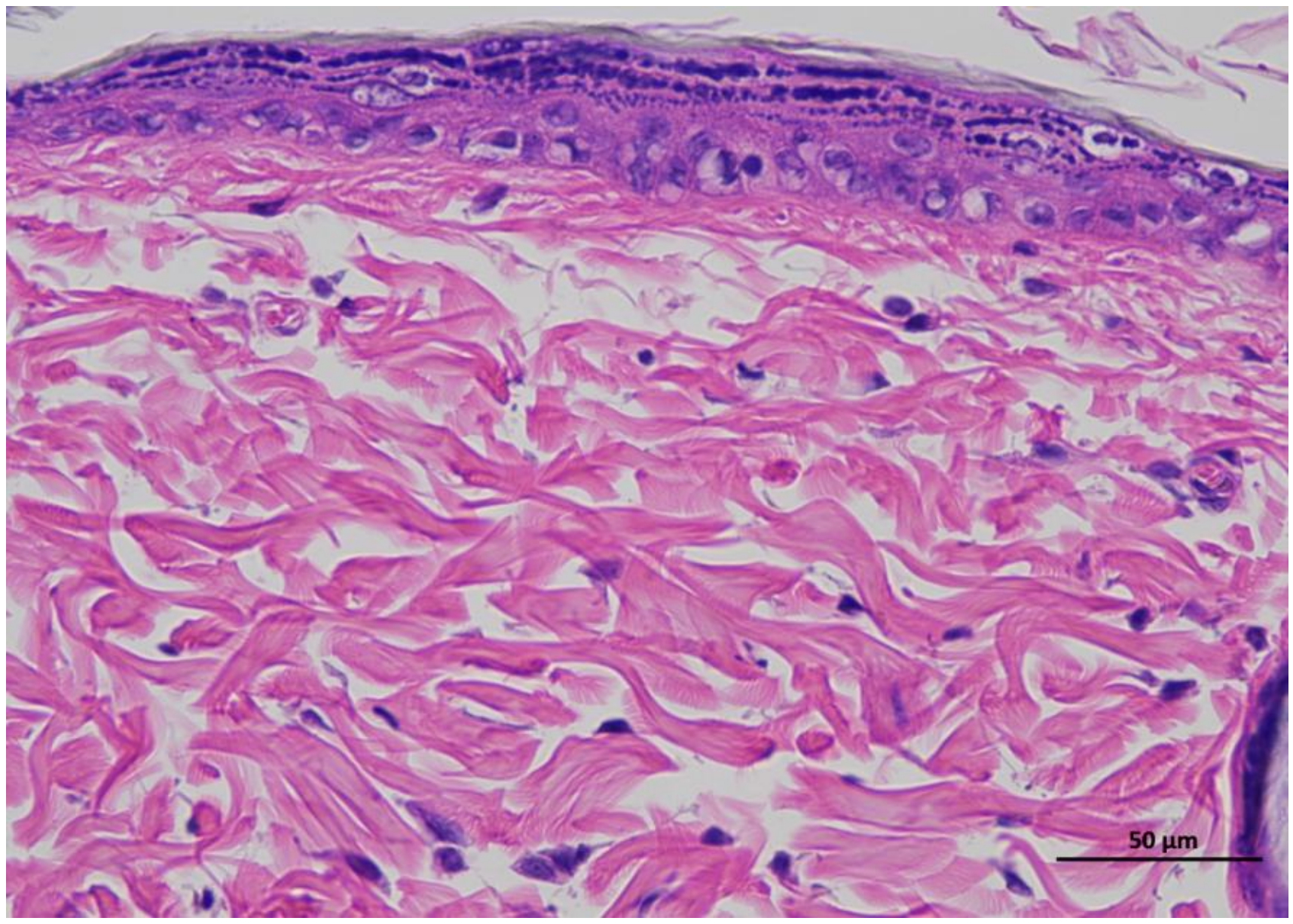

6 RNA-LNPs-H 0day 400-1

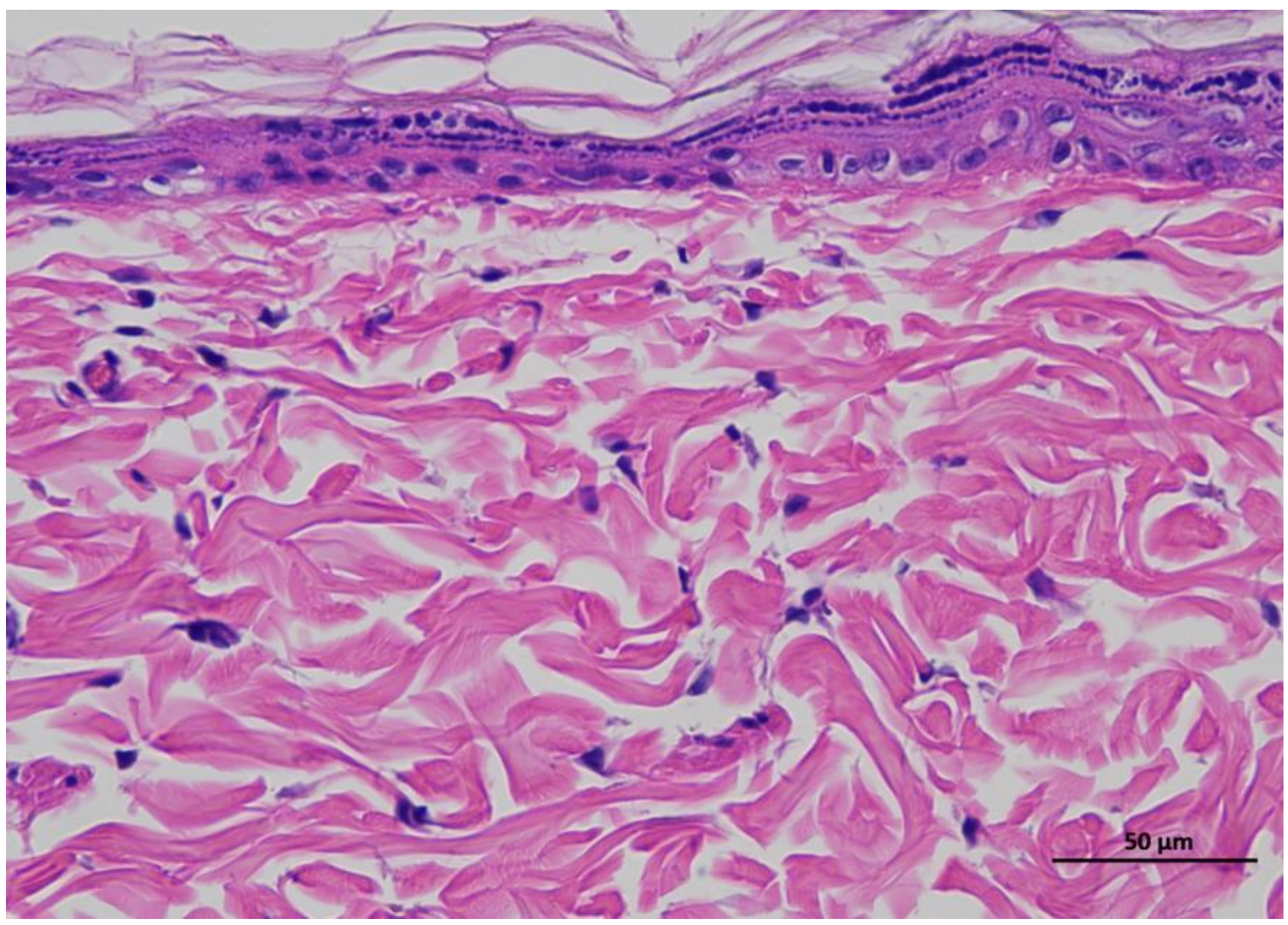

6 RNA-LNPs-H 0day 400-2

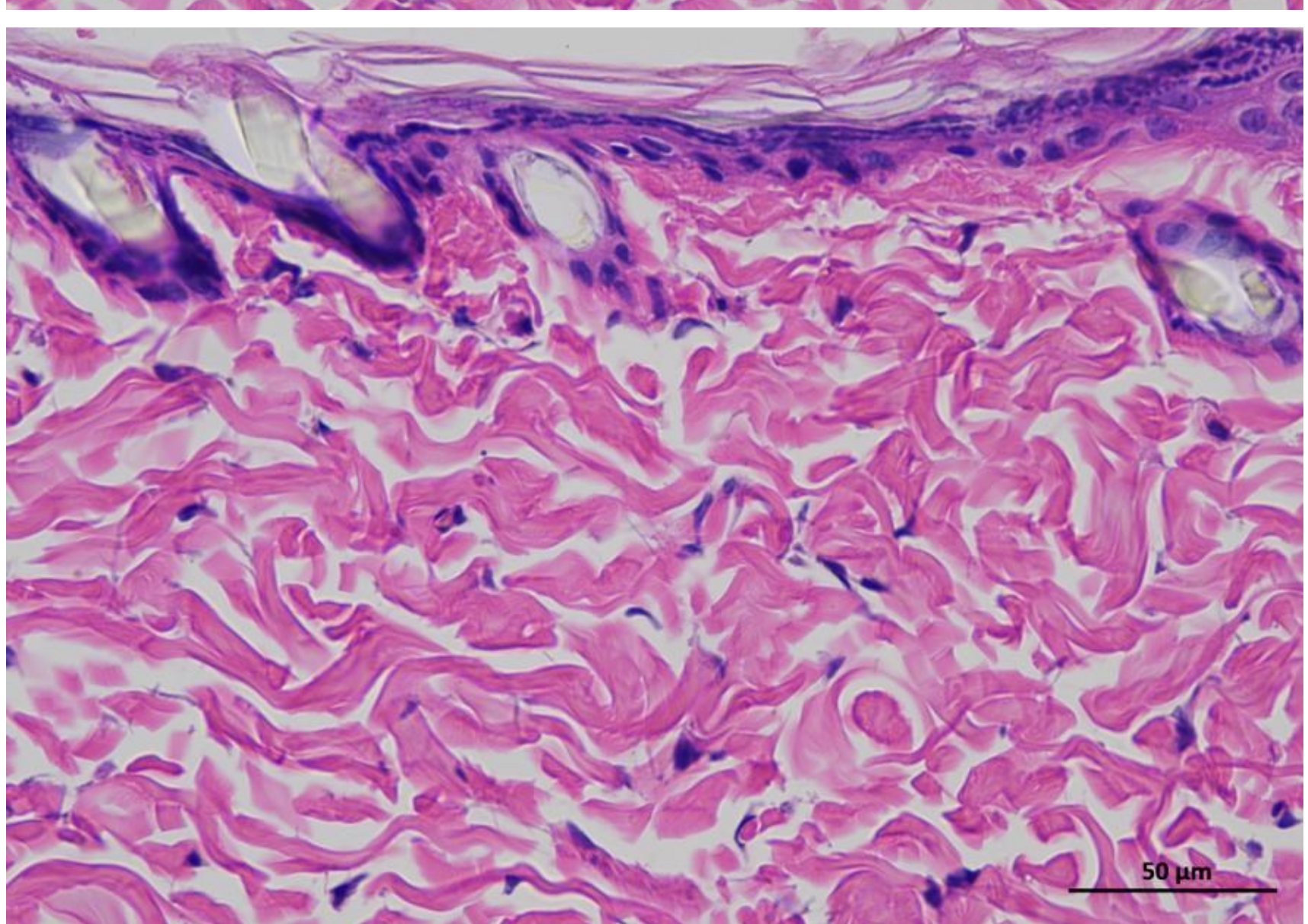

6 RNA-LNPs-H 0day 400-3

Supplementary Figure 3d | Representative H&E staining of skin tissue of RNA-LNPs-H animals / Day 14.
Representative hematoxylin and eosin (H&E)–stained section of skin tissue collected from experimental rat. The image shows the typical architecture of the skin, including the epidermal layer with stratified keratinized epithelium and the underlying dermis composed of dense collagen fibers and connective tissue structures.
(n = 6 per group).

Animal 1

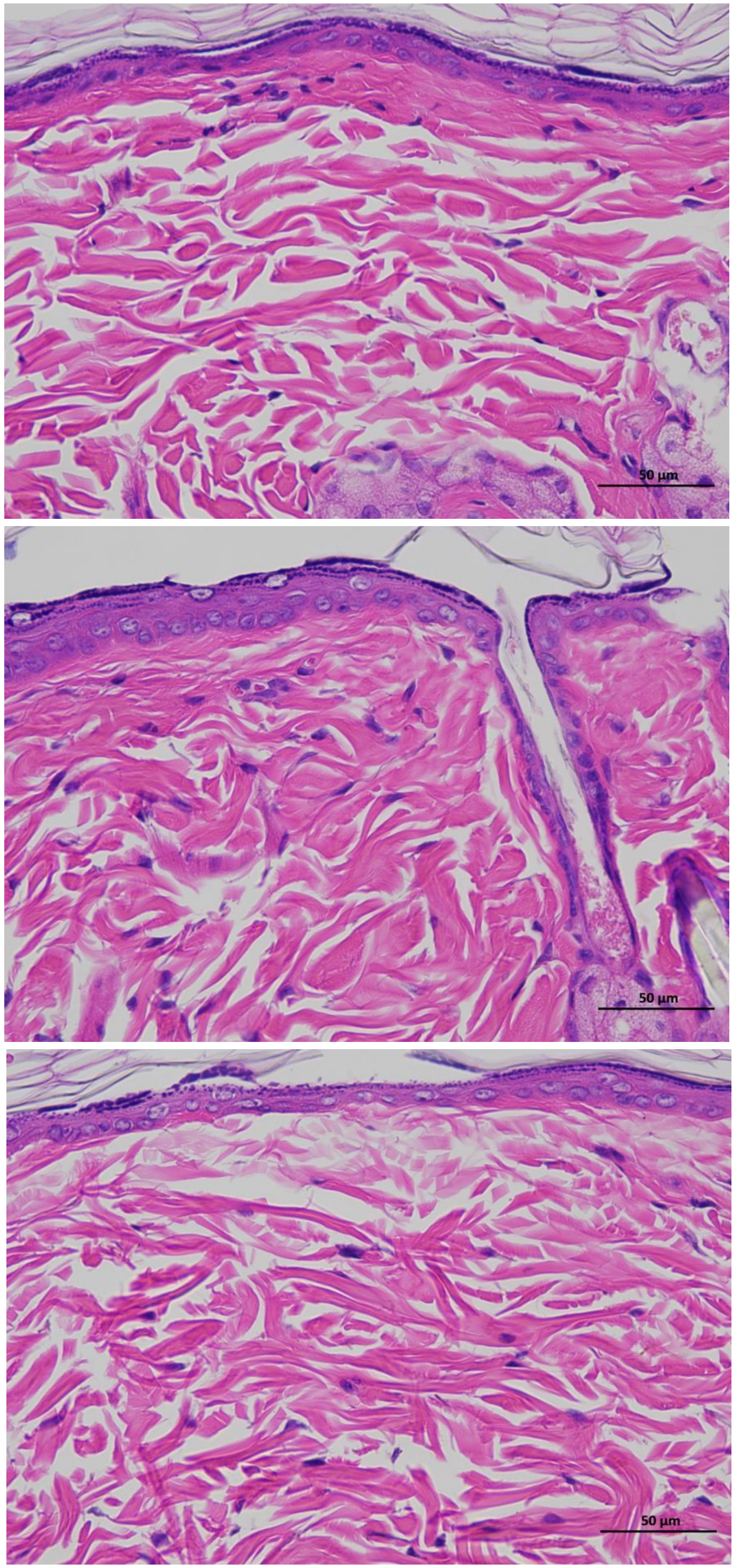

1 RNA-LNPs-H 14day 400-1

1 RNA-LNPs-H 14day 400-2

1 RNA-LNPs-H 14day 400-3

Supplementary Figure 3d | Representative H&E staining of skin tissue of RNA-LNPs-H animals / Day 14.

Animal 2

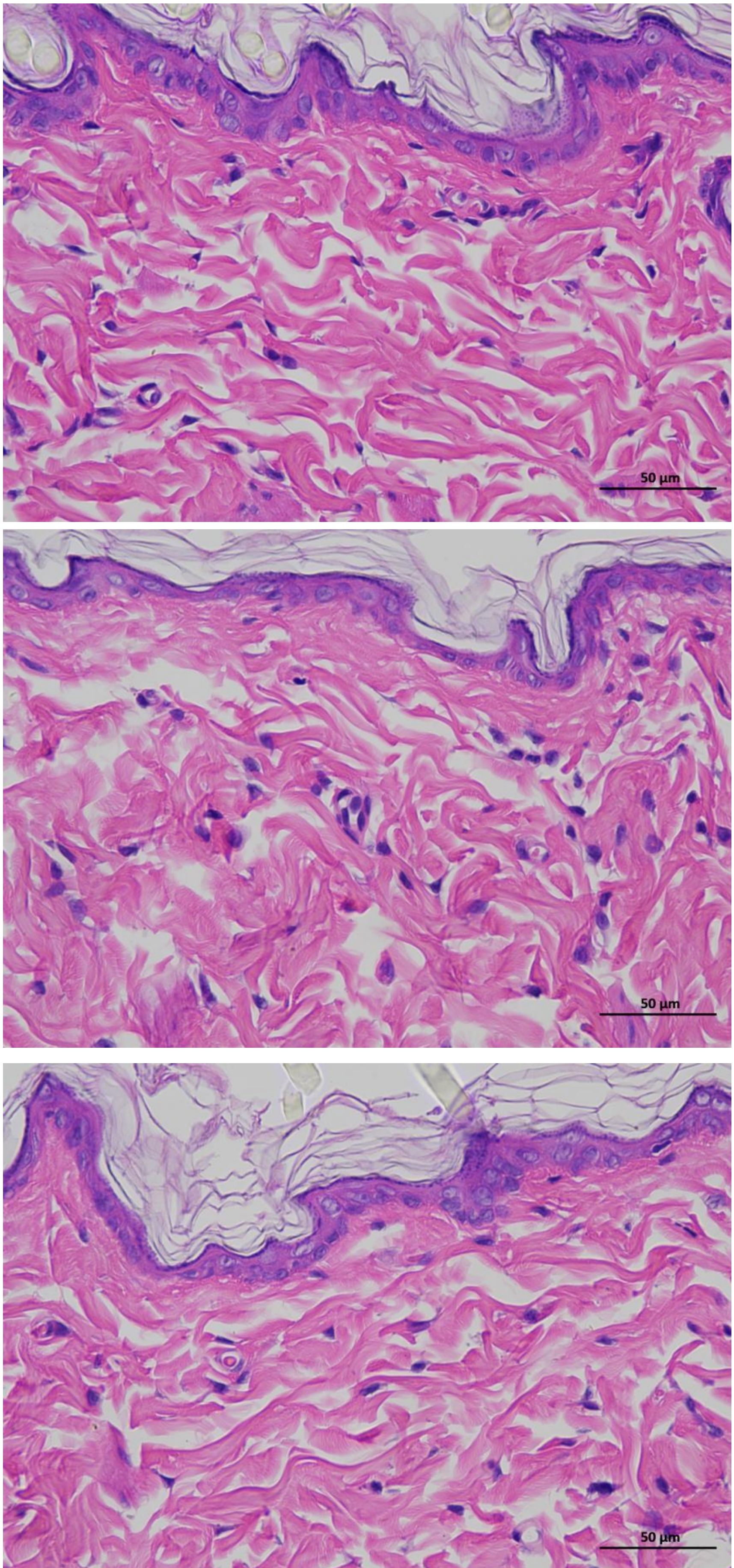

Supplementary Figure 3d | Representative H&E staining of skin tissue of RNA-LNPs-H animals / Day 14.

Animal 3

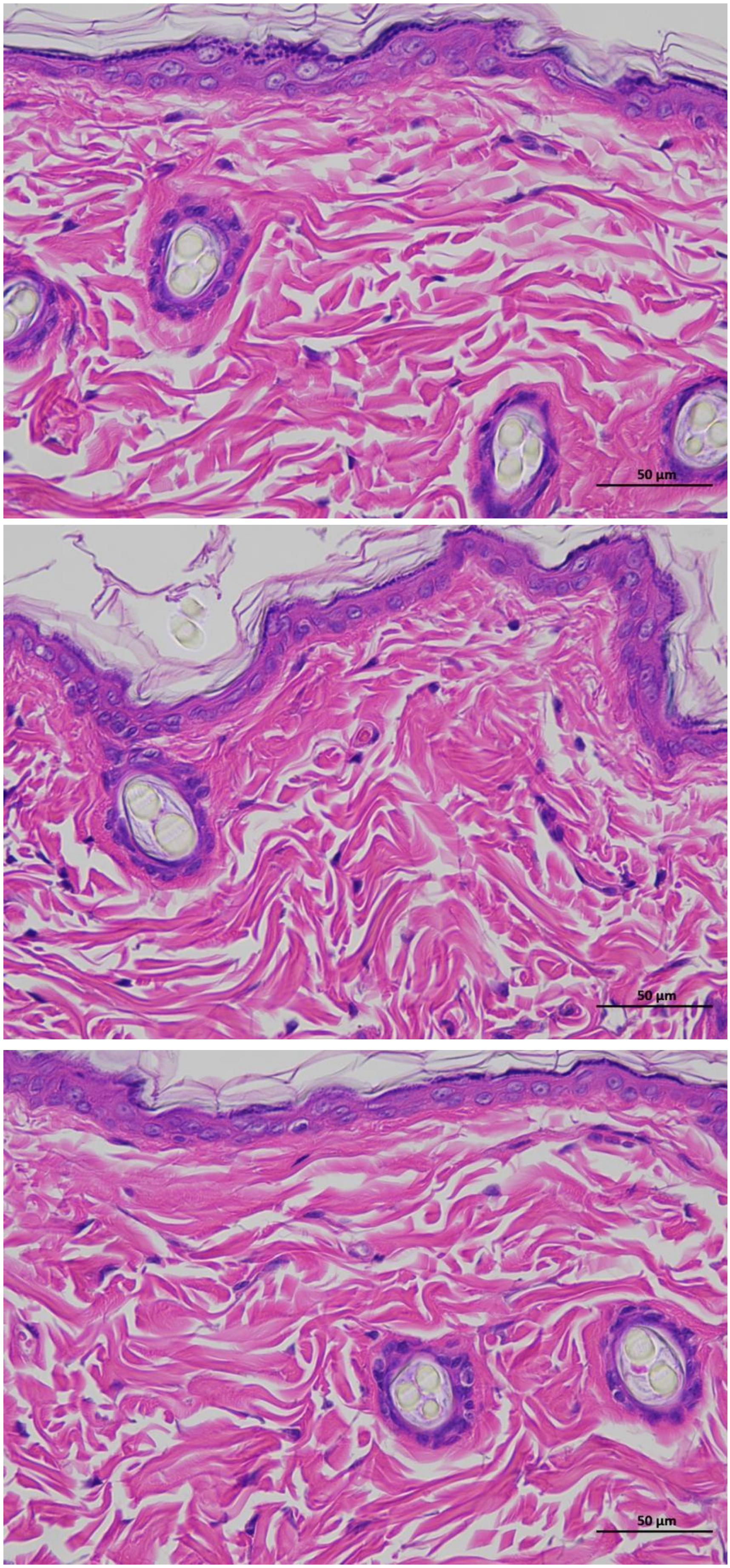

Animal 4

Supplementary Figure 3d | Representative H&E staining of skin tissue of RNA-LNPs-H animals / Day 14.

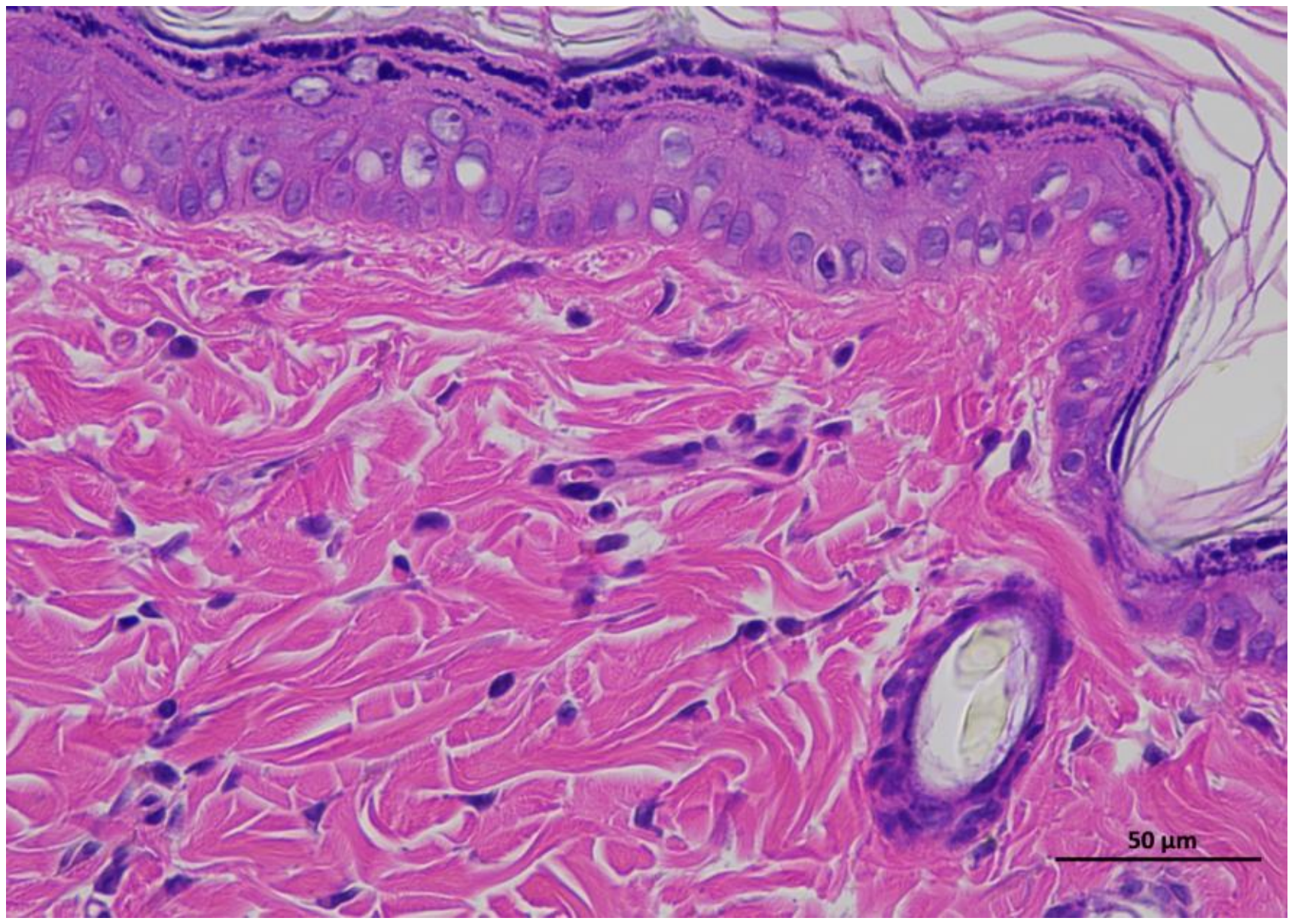

4 RNA-LNPs-H 14day 400-1

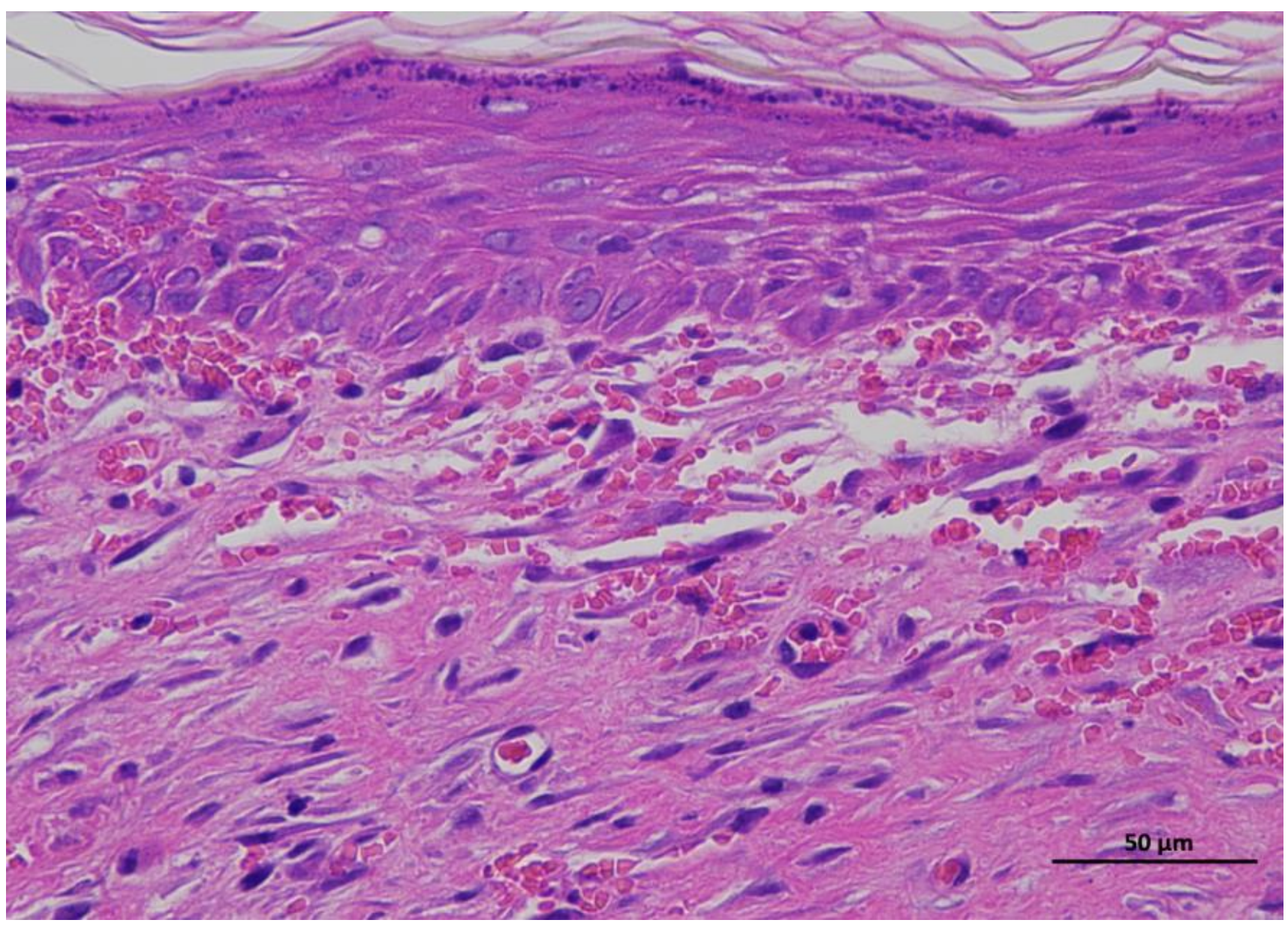

4 RNA-LNPs-H 14day 400-2

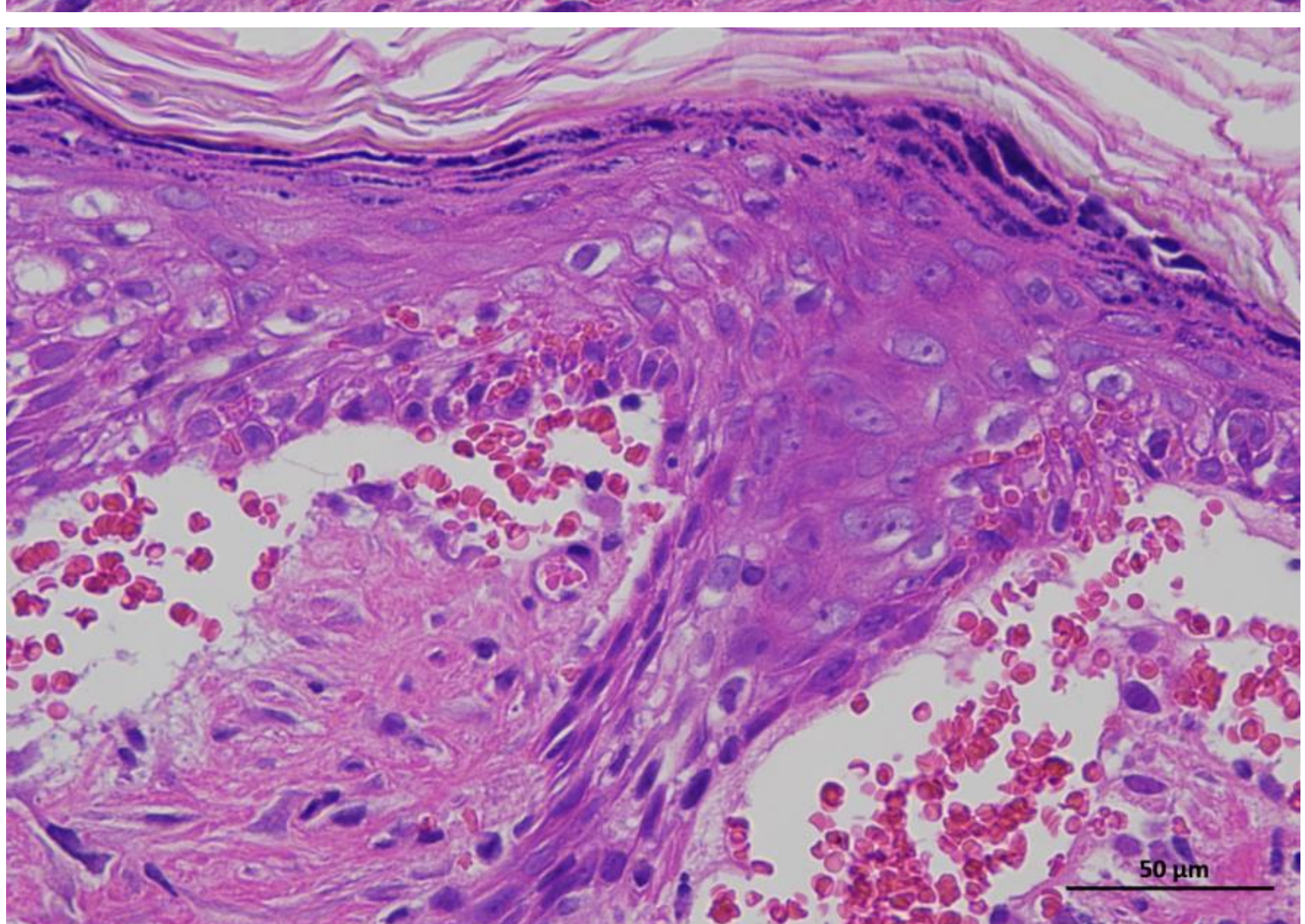

4 RNA-LNPs-H 14day 400-3

Animal 5

Supplementary Figure 3d | Representative H&E staining of skin tissue of RNA-LNPs-H animals / Day 14.

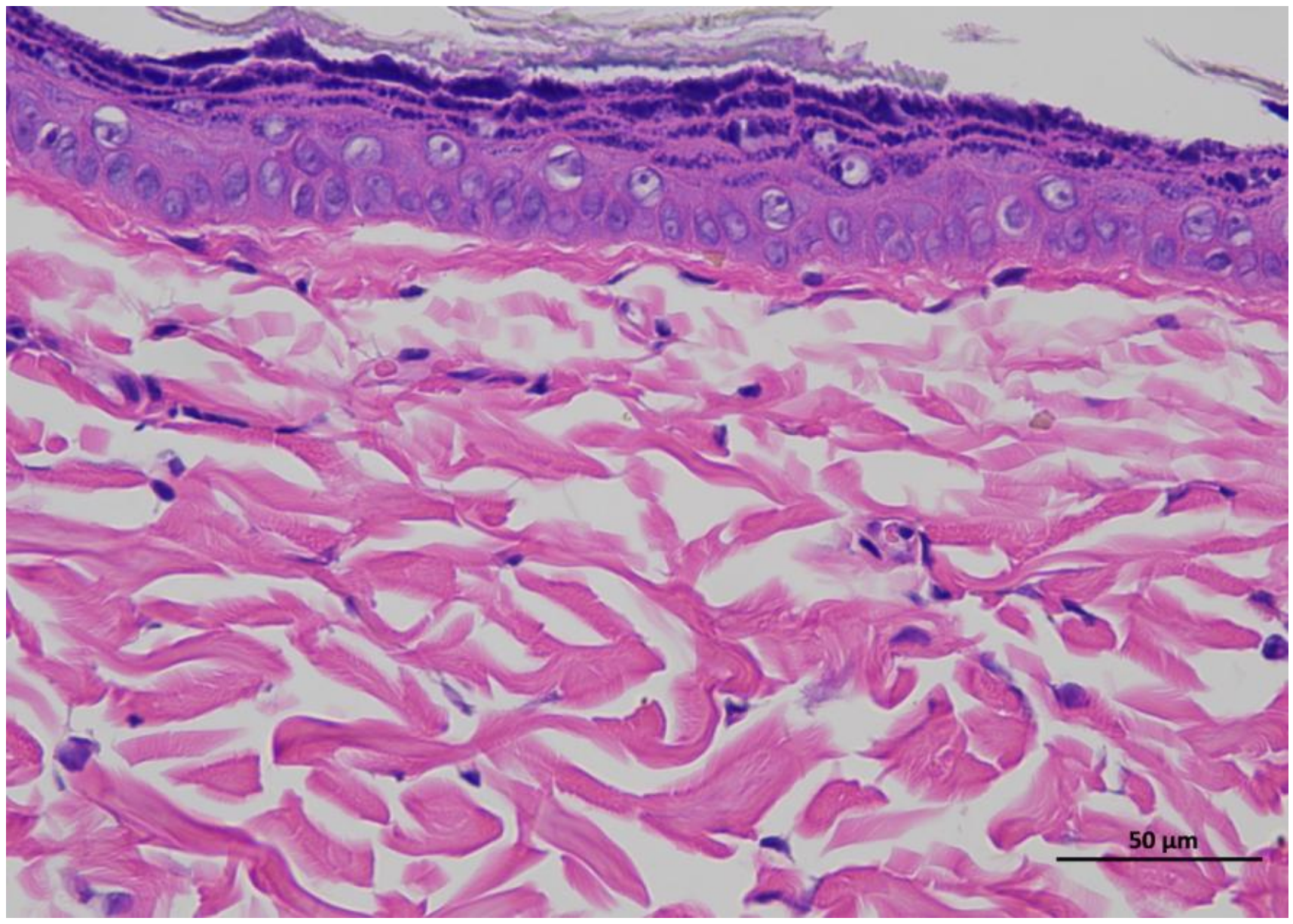

5 RNA-LNPs-H 14day 400-1

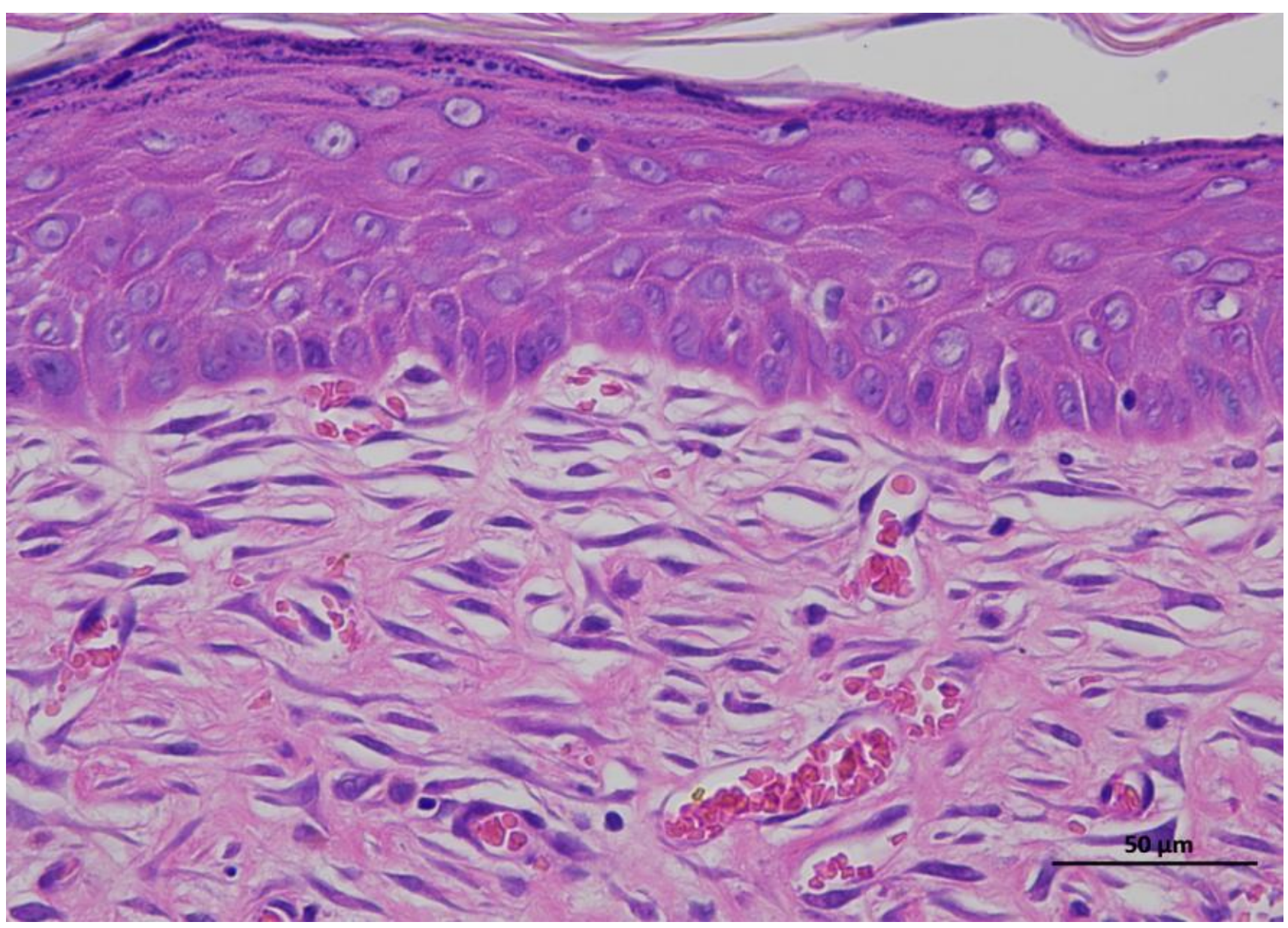

5 RNA-LNPs-H 14day 400-2

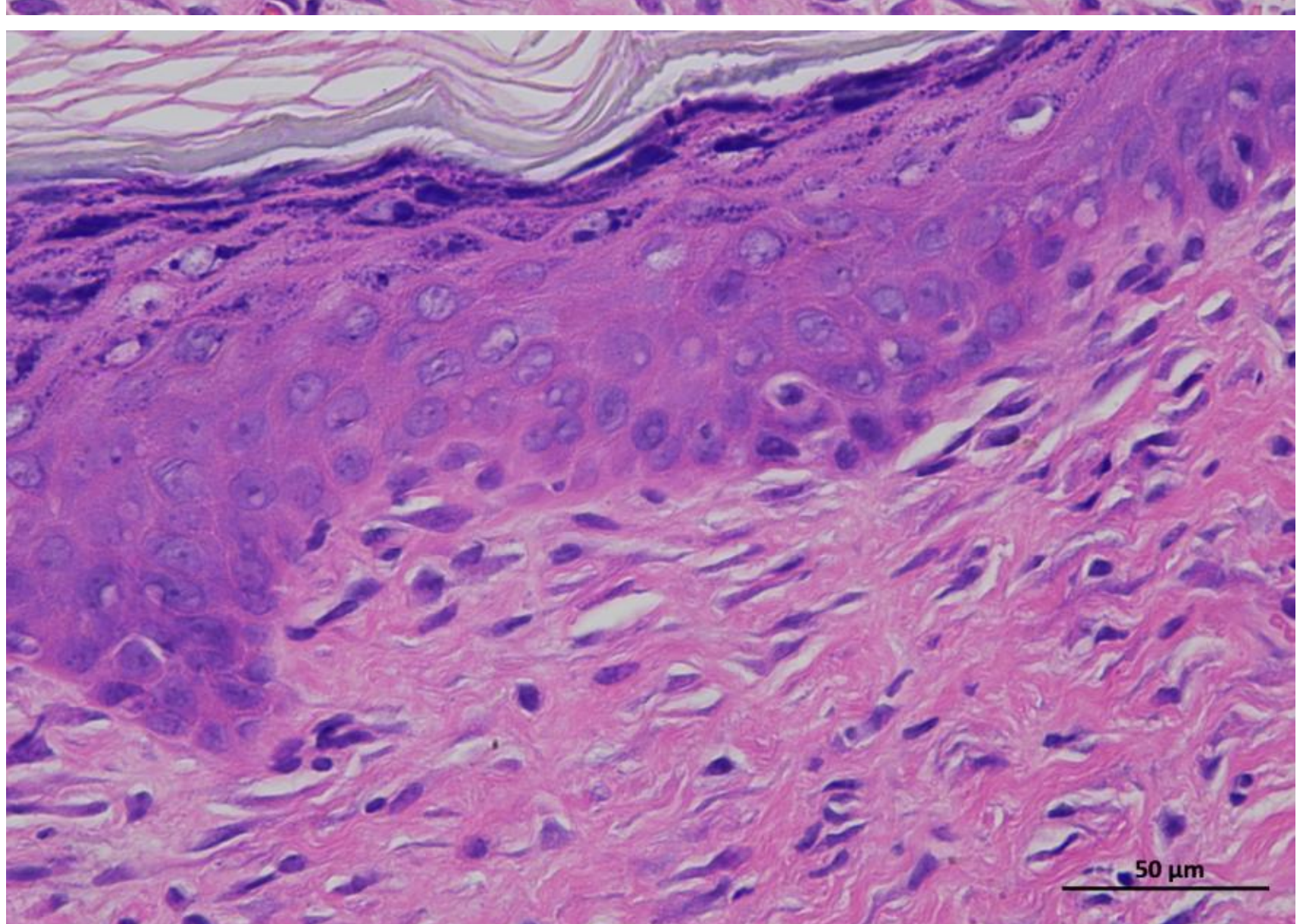

5 RNA-LNPs-H 14day 400-3

Animal 6

Supplementary Figure 3d | Representative H&E staining of skin tissue of RNA-LNPs-H animals / Day 14.

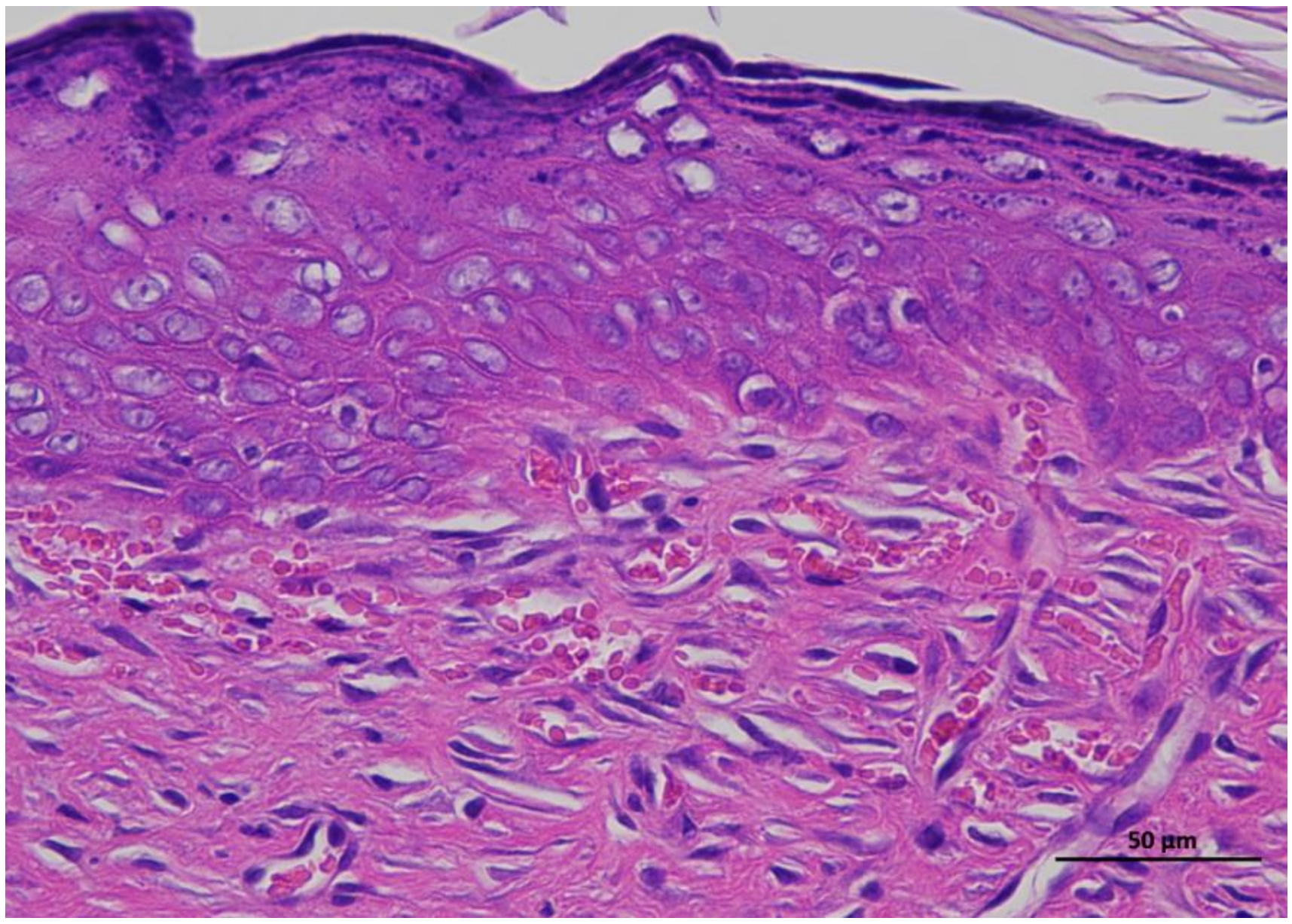

6 RNA-LNPs-H 14day 400-1

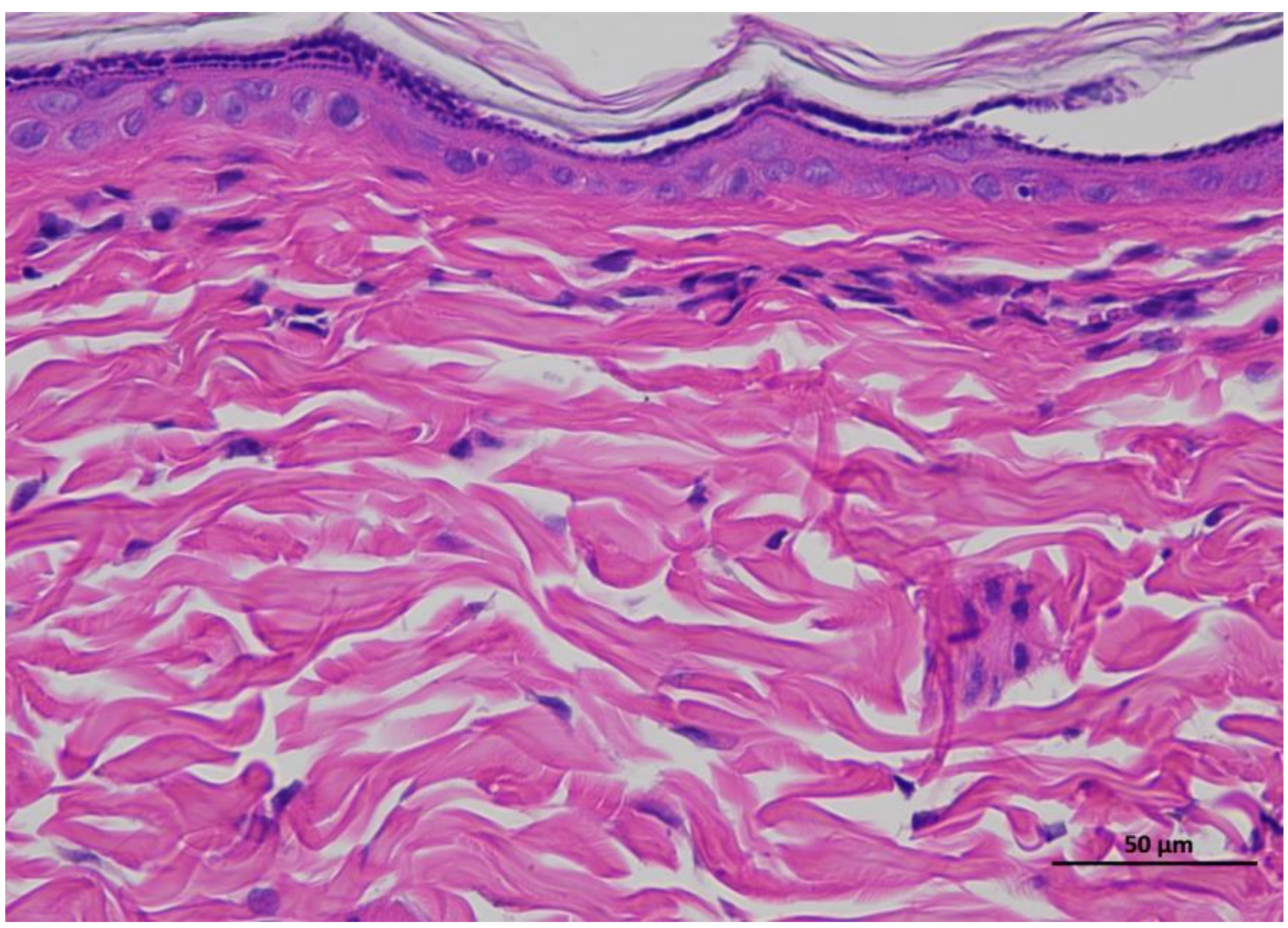

6 RNA-LNPs-H 14day 400-2

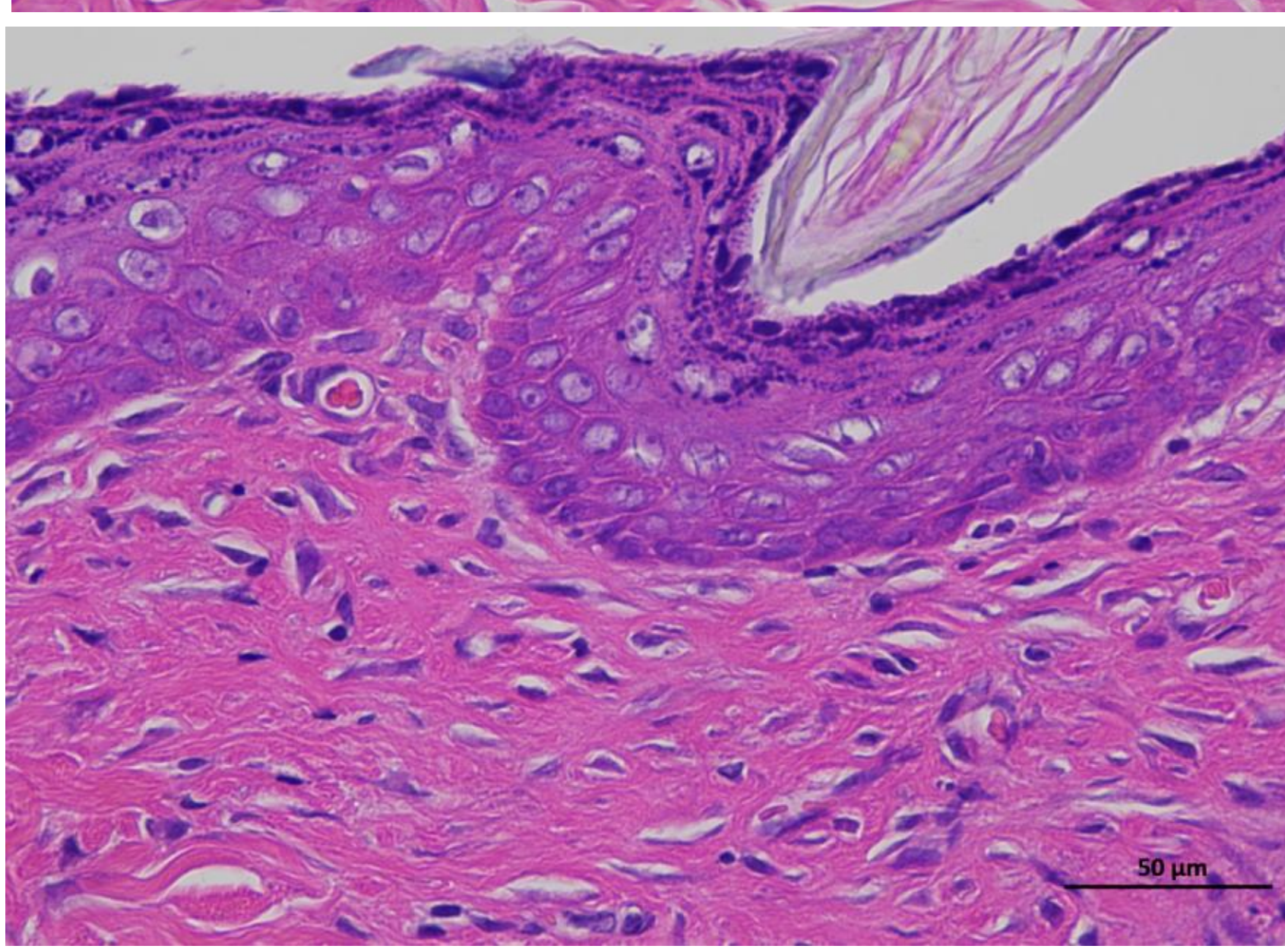

6 RNA-LNPs-H 14day 400-3

Animal 1

Supplementary Figure 2e | Representative H&E staining of skin tissue of RNA-BEV-L animals / Day 0.
Representative hematoxylin and eosin (H&E)–stained section of skin tissue collected from experimental rat. The image shows the typical architecture of the skin, including the epidermal layer with stratified keratinized epithelium and the underlying dermis composed of dense collagen fibers and connective tissue structures.
(n = 6 per group).

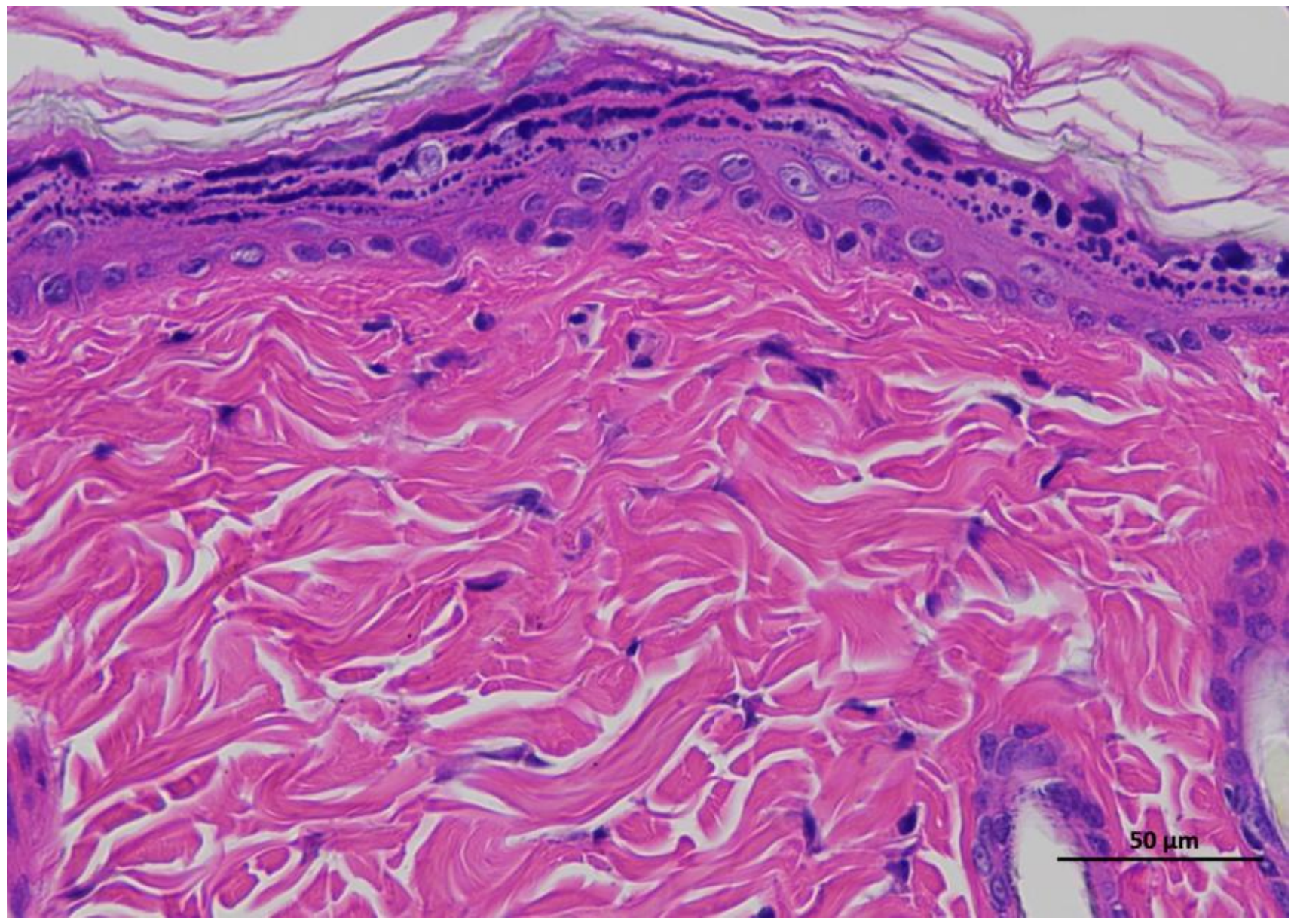

1 RNA-BEV-L 0day 400-1

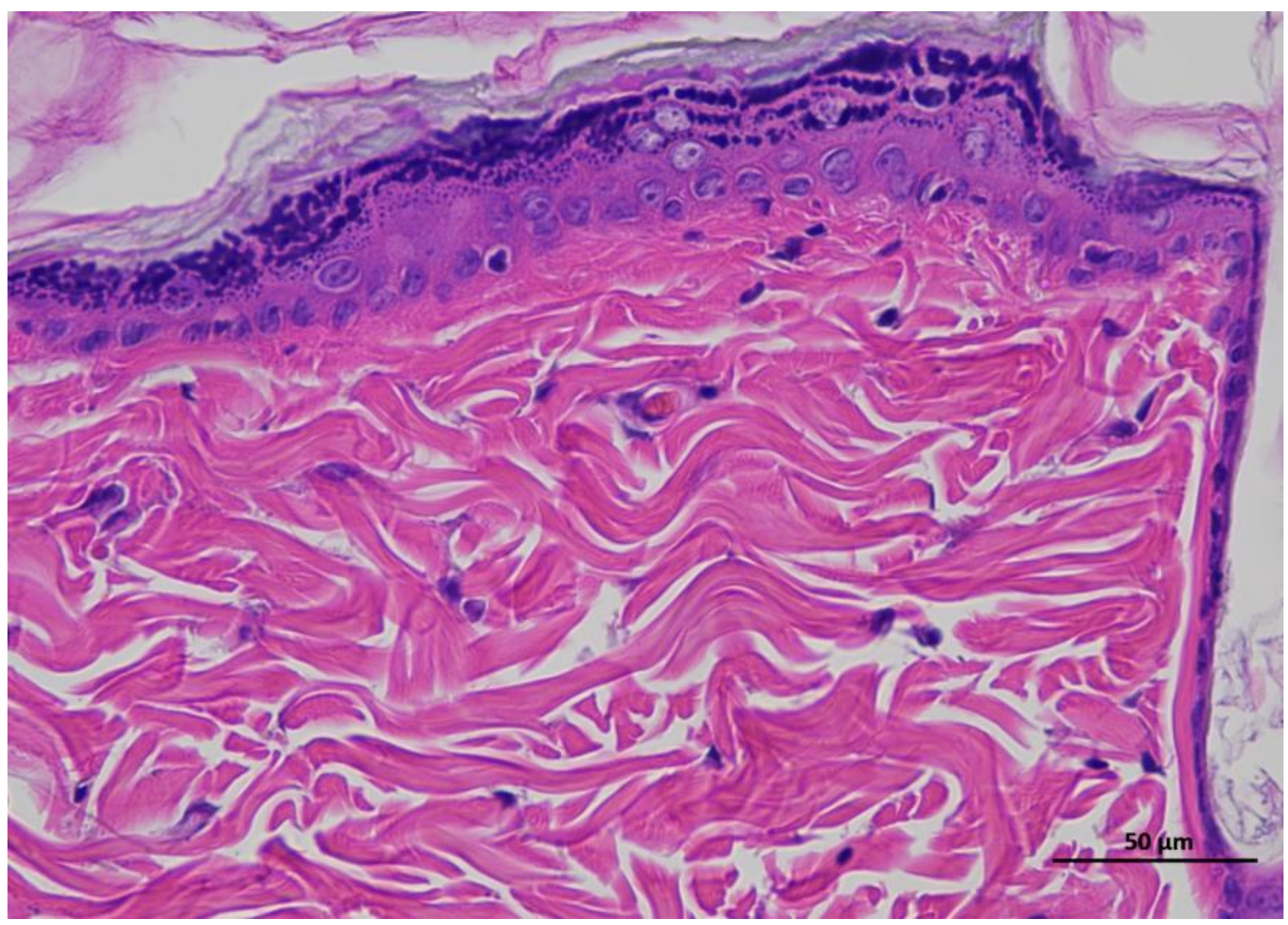

1 RNA-BEV-L 0day 400-2

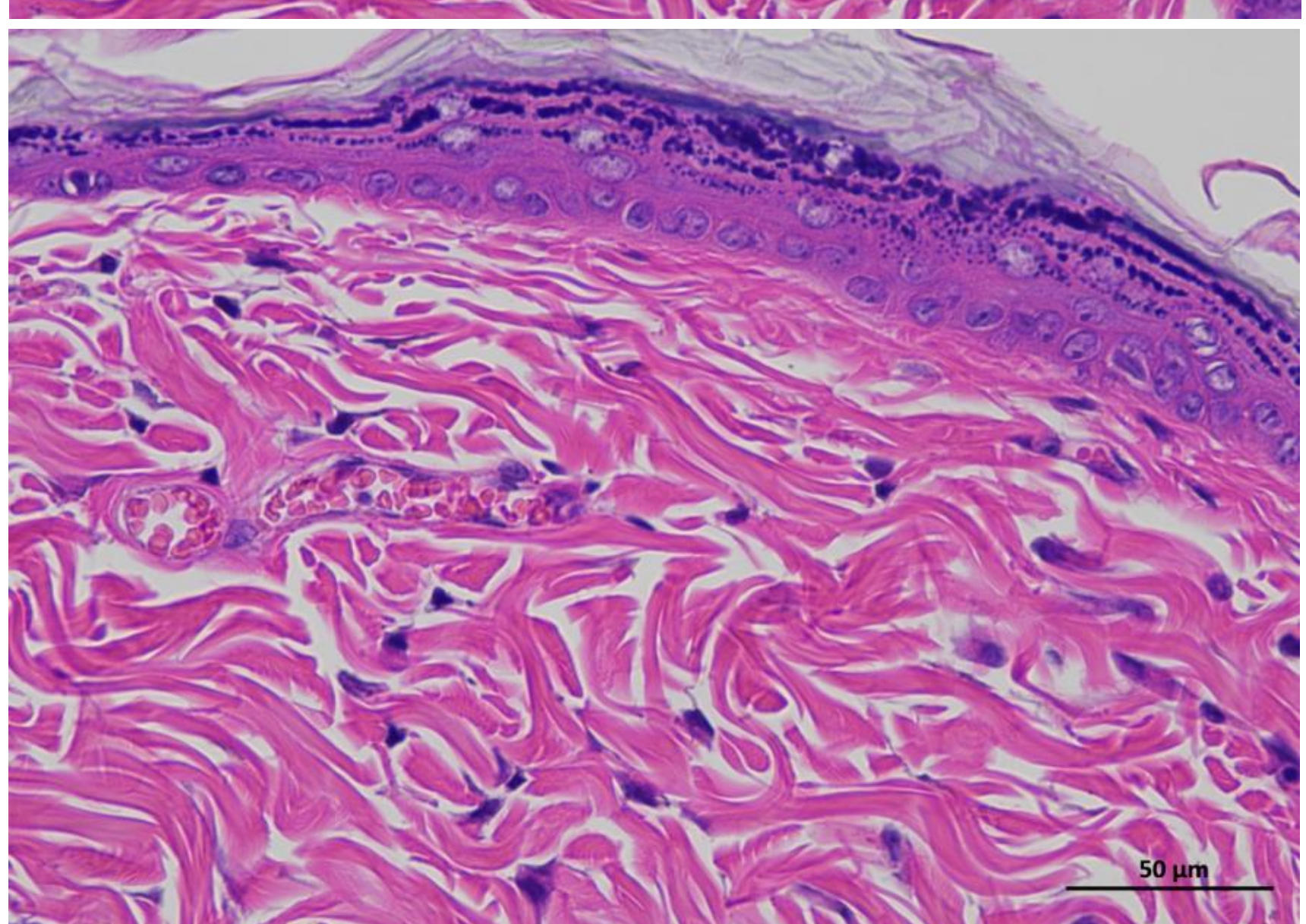

1 RNA-BEV-L 0day 400-3

Animal 2

Supplementary Figure 2e| Representative H&E staining of skin tissue of mRNA RNA-BEV-L animals / Day 0.

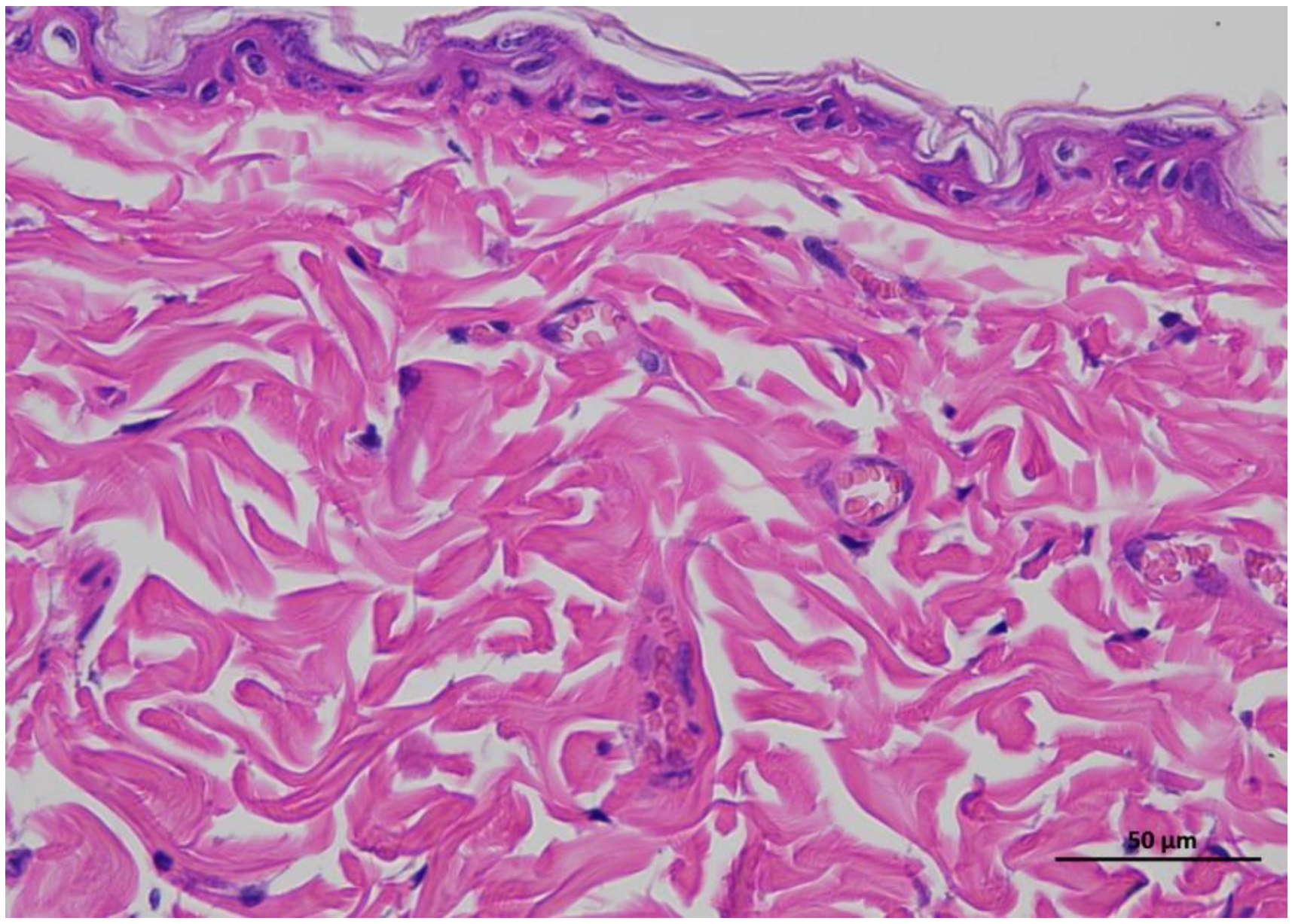

2 RNA-BEV-L 0day 400-1

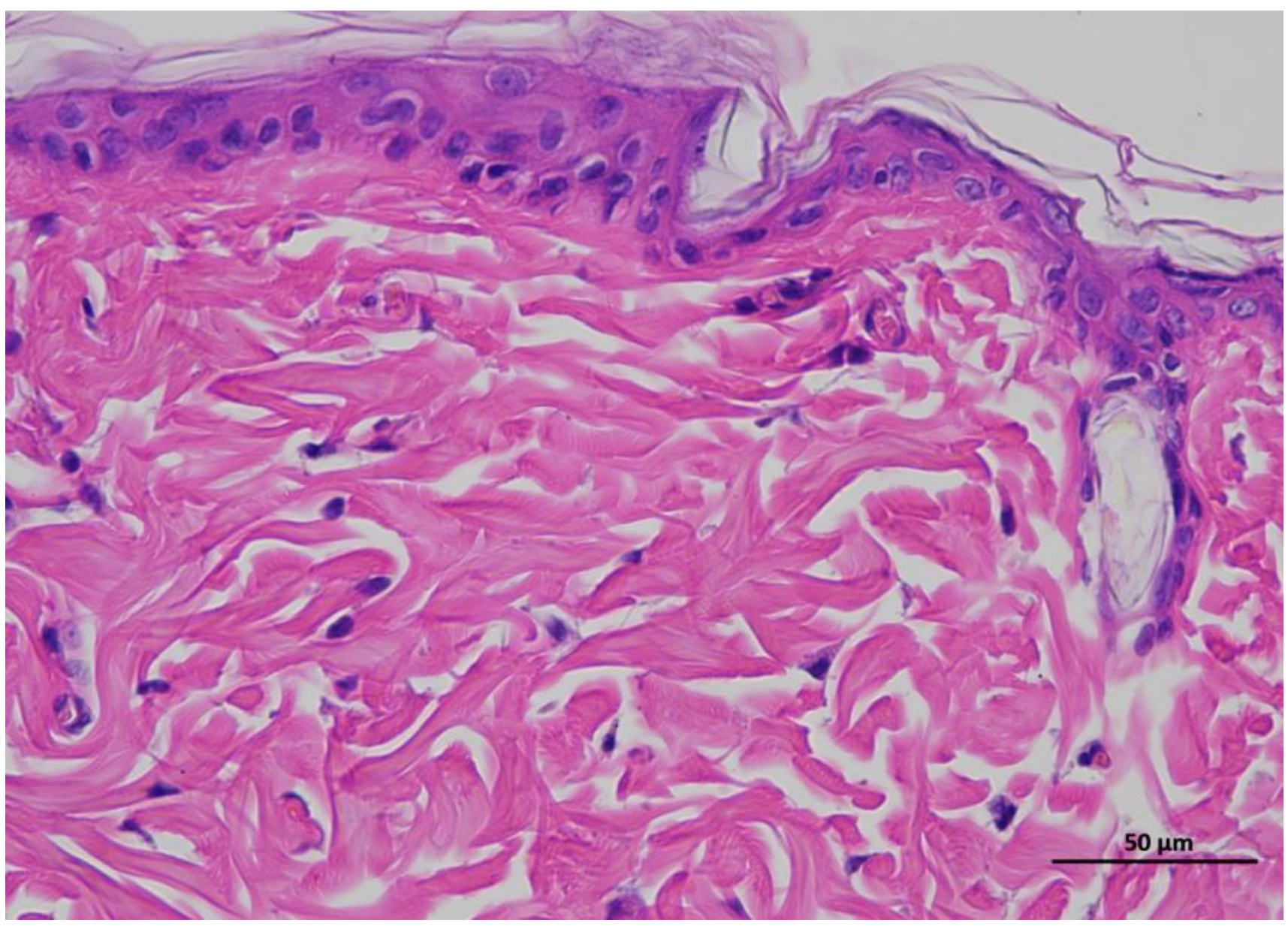

2 RNA-BEV-L 0day 400-2

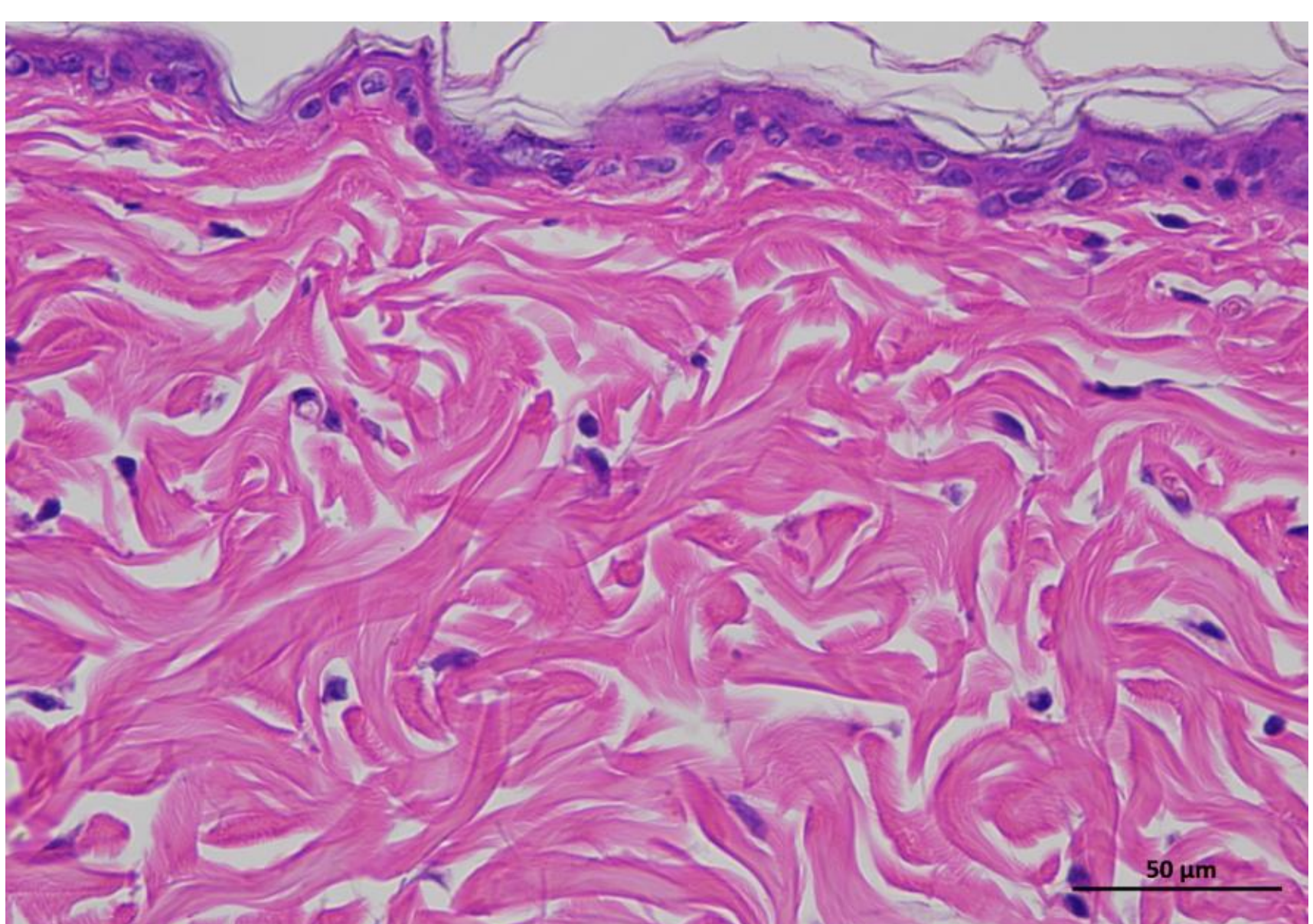

2 RNA-BEV-L 0day 400-3

Supplementary Figure 2e| Representative H&E staining of skin tissue of mRNA RNA-BEV-L animals / Day 0.

Animal 3

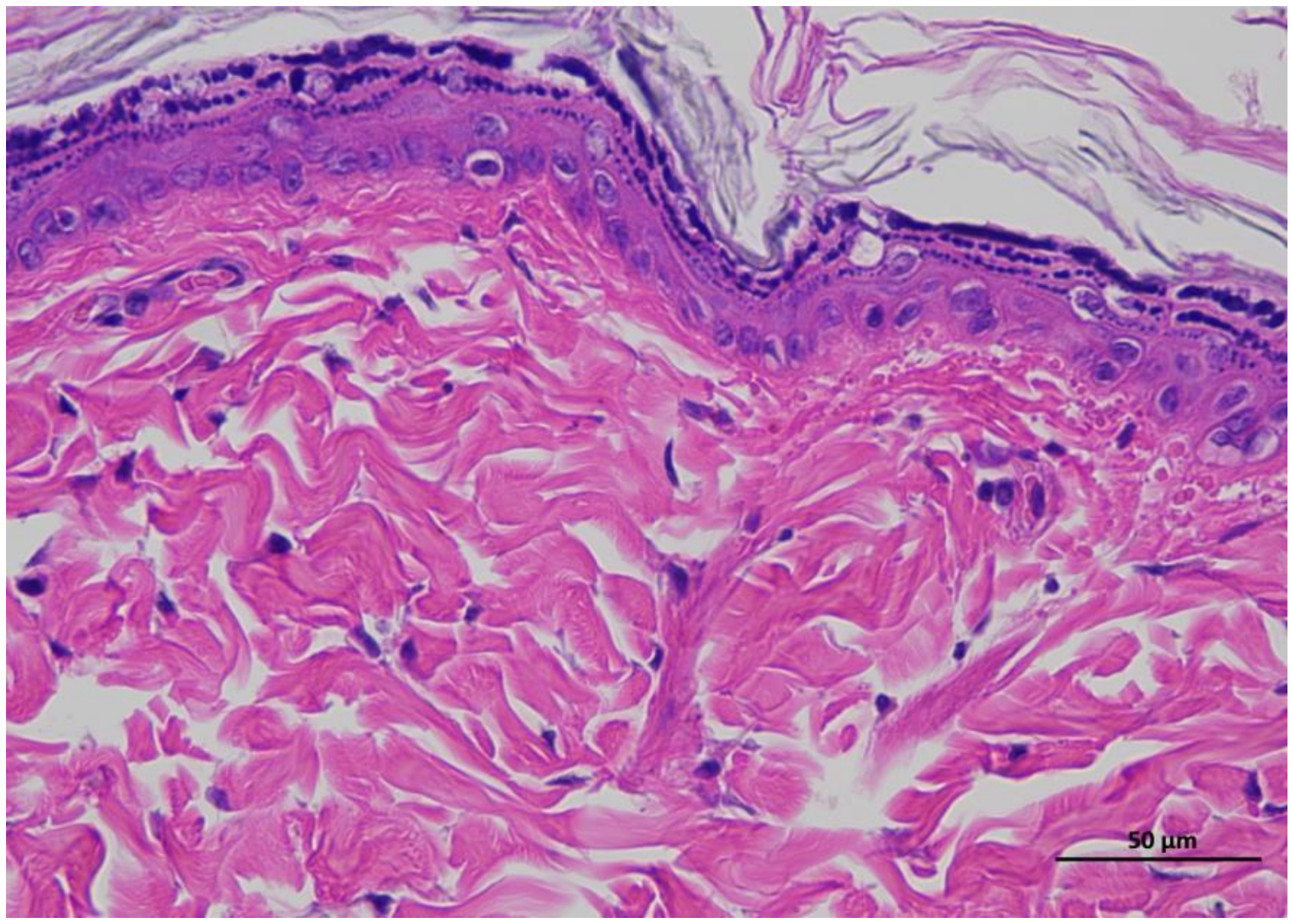

3 RNA-BEV-L 0day 400-1

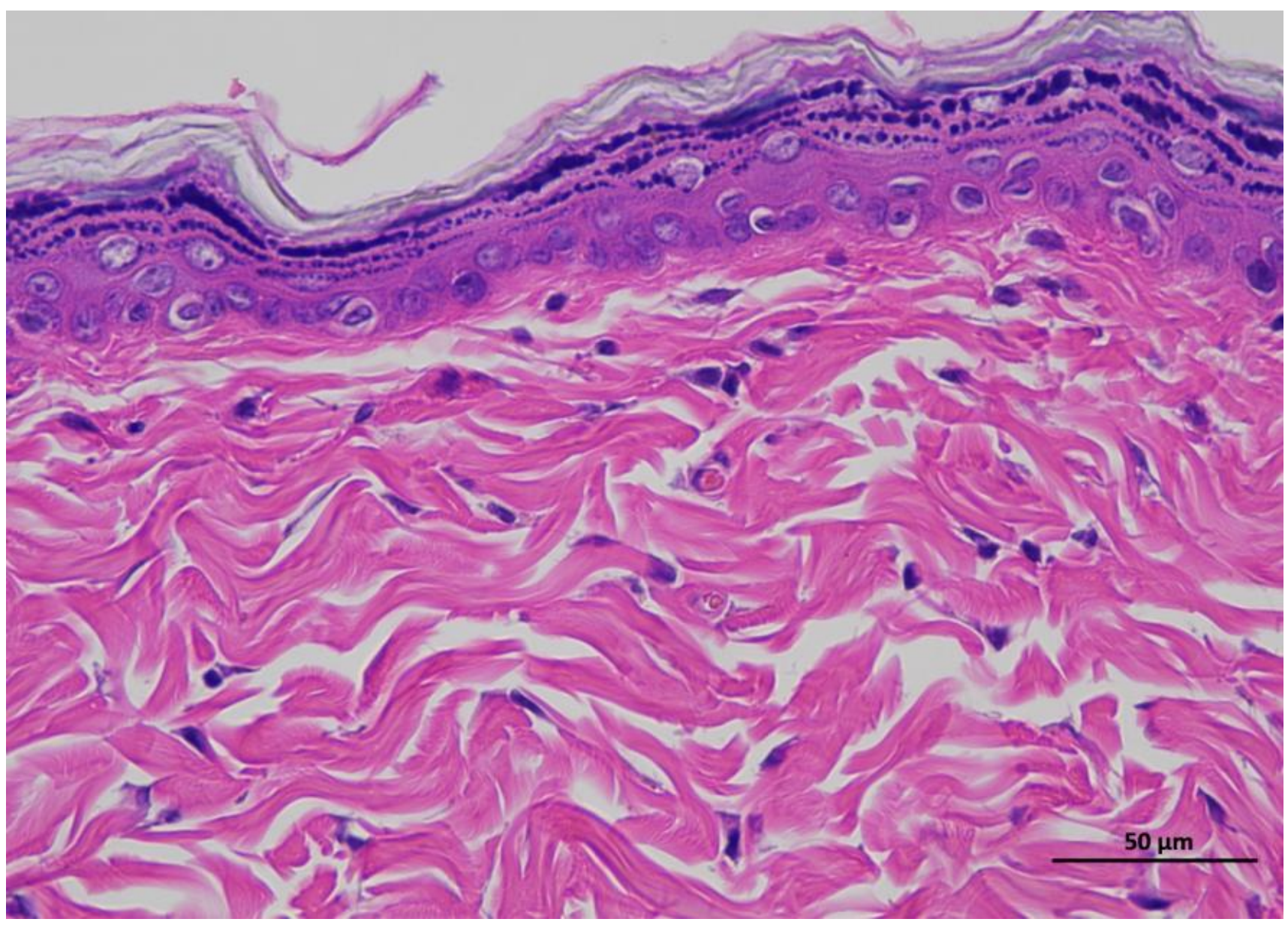

3 RNA-BEV-L 0day 400-2

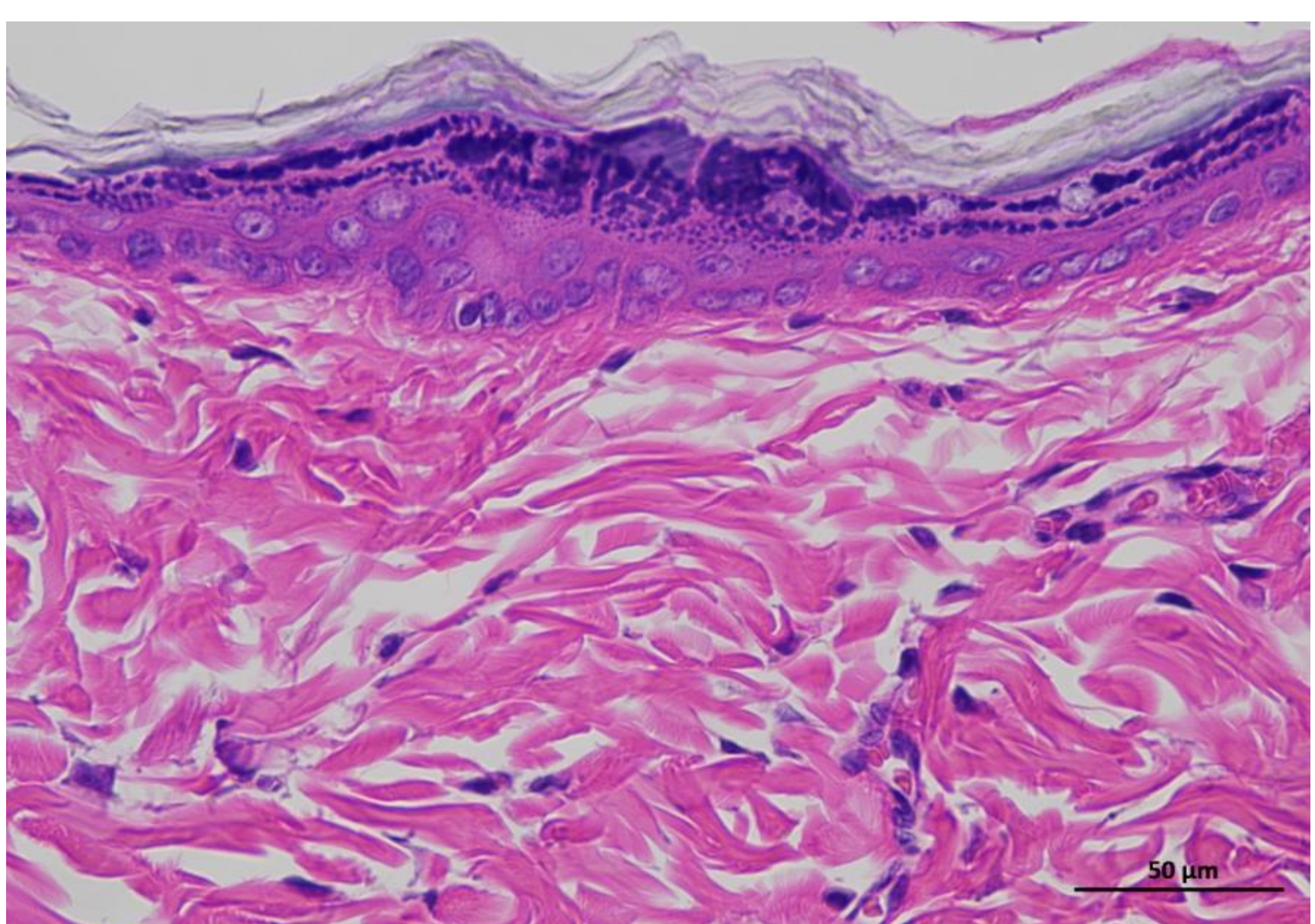

3 RNA-BEV-L 0day 400-3

Animal 4

Supplementary Figure 2e| Representative H&E staining of skin tissue of mRNA RNA-BEV-L animals / Day 0.

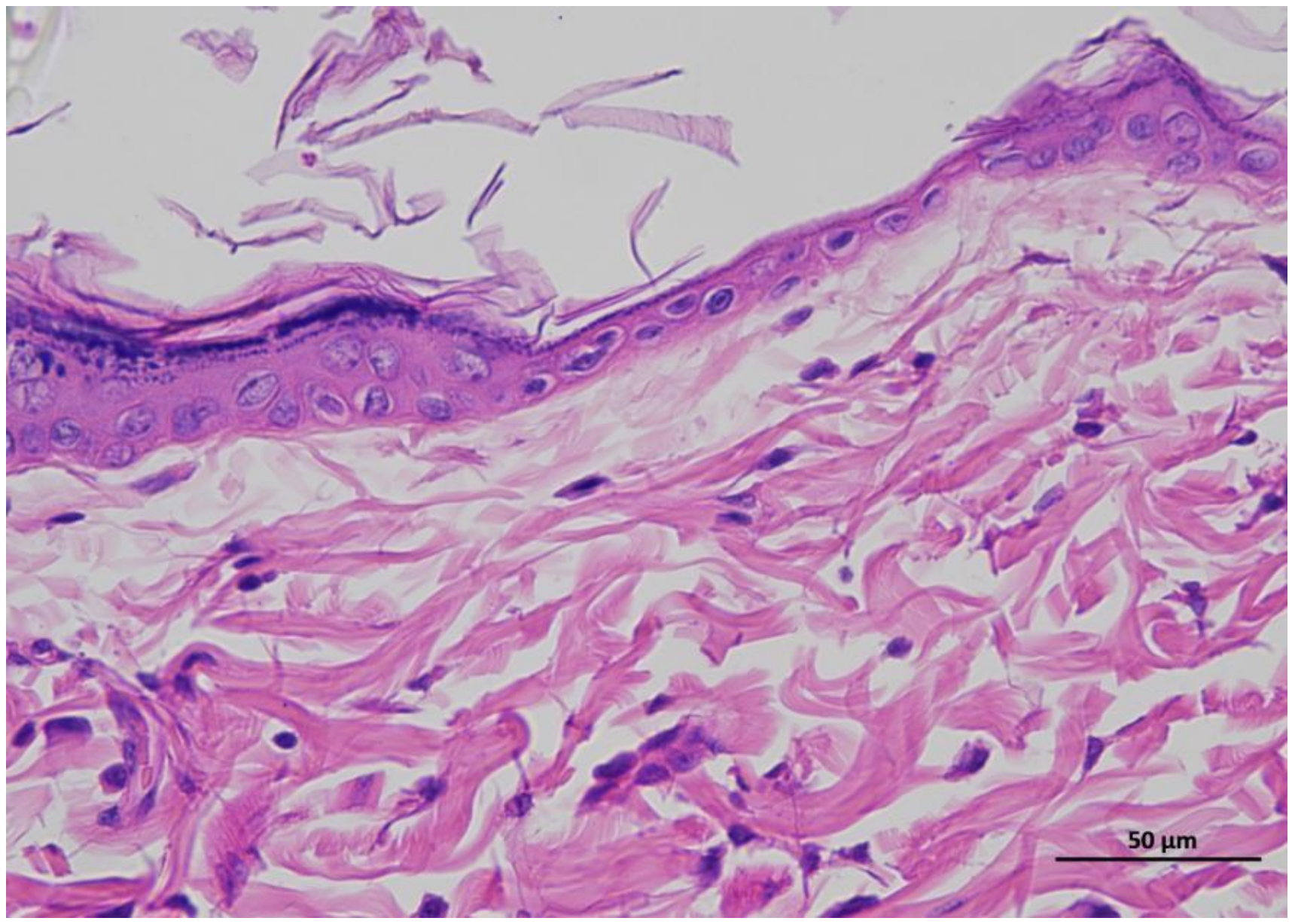

4 RNA-BEV-L 0day 400-1

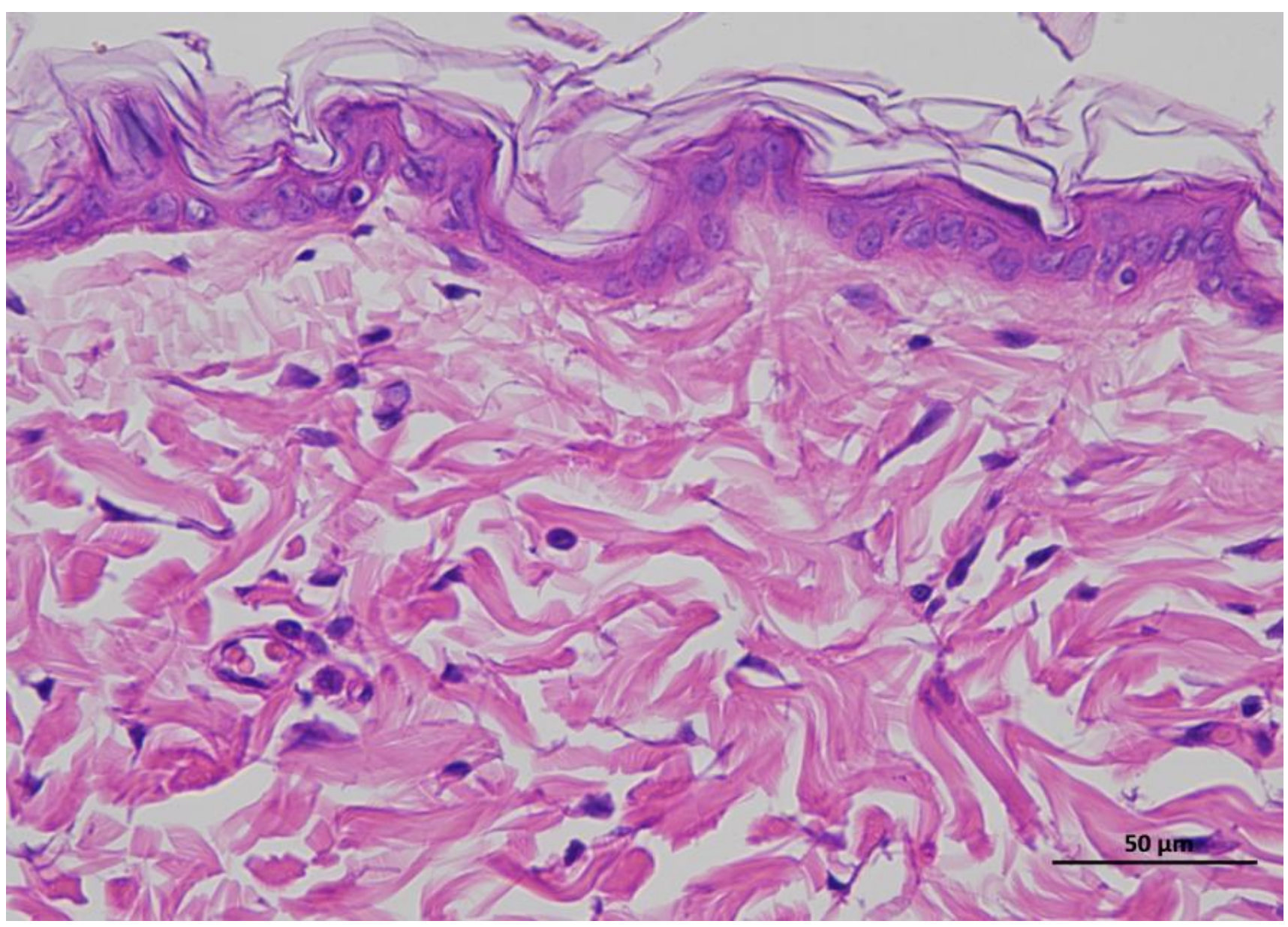

4 RNA-BEV-L 0day 400-2

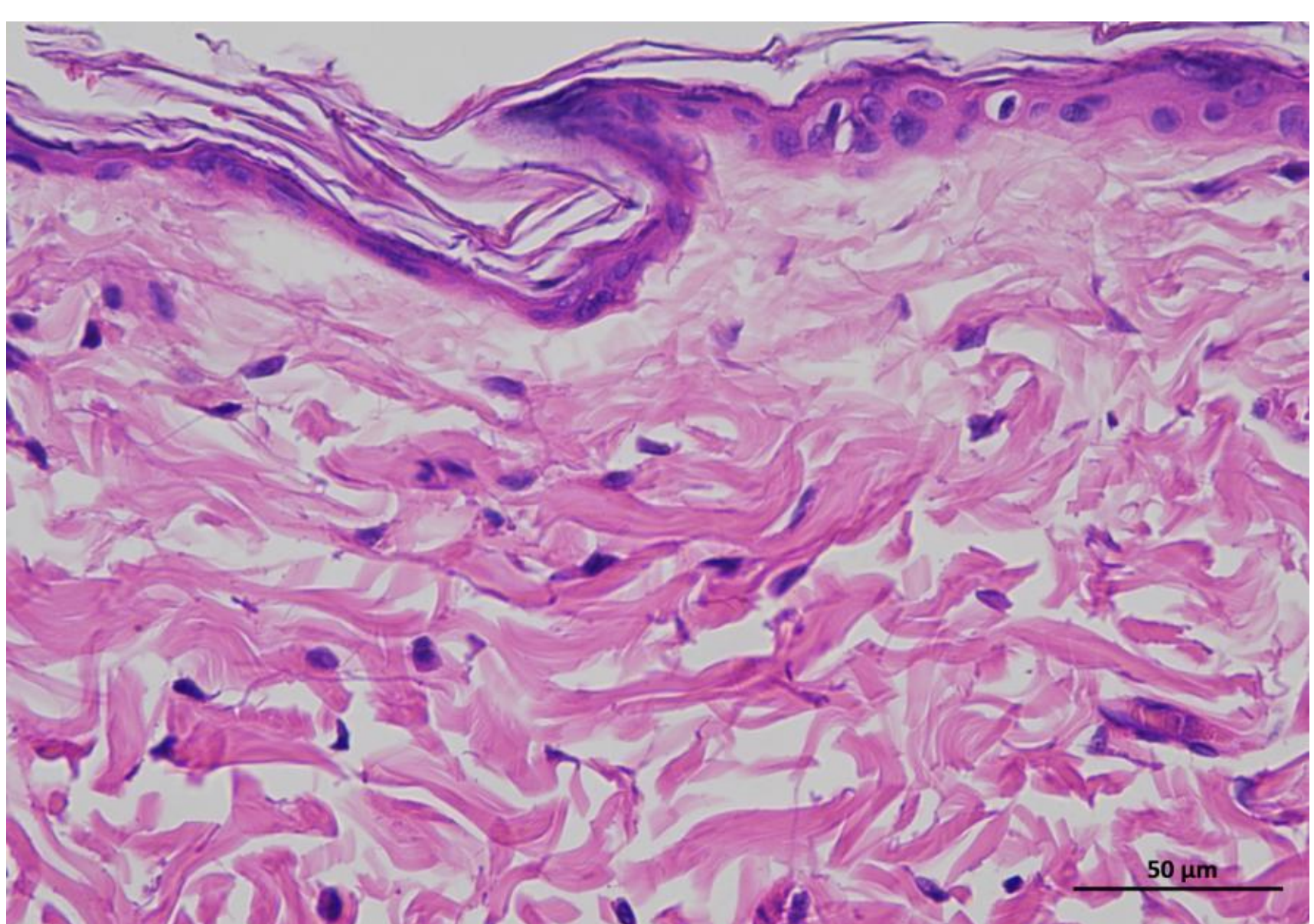

4 RNA-BEV-L 0day 400-3

Animal 5

Supplementary Figure 2e| Representative H&E staining of skin tissue of mRNA RNA-BEV-L animals / Day 0.

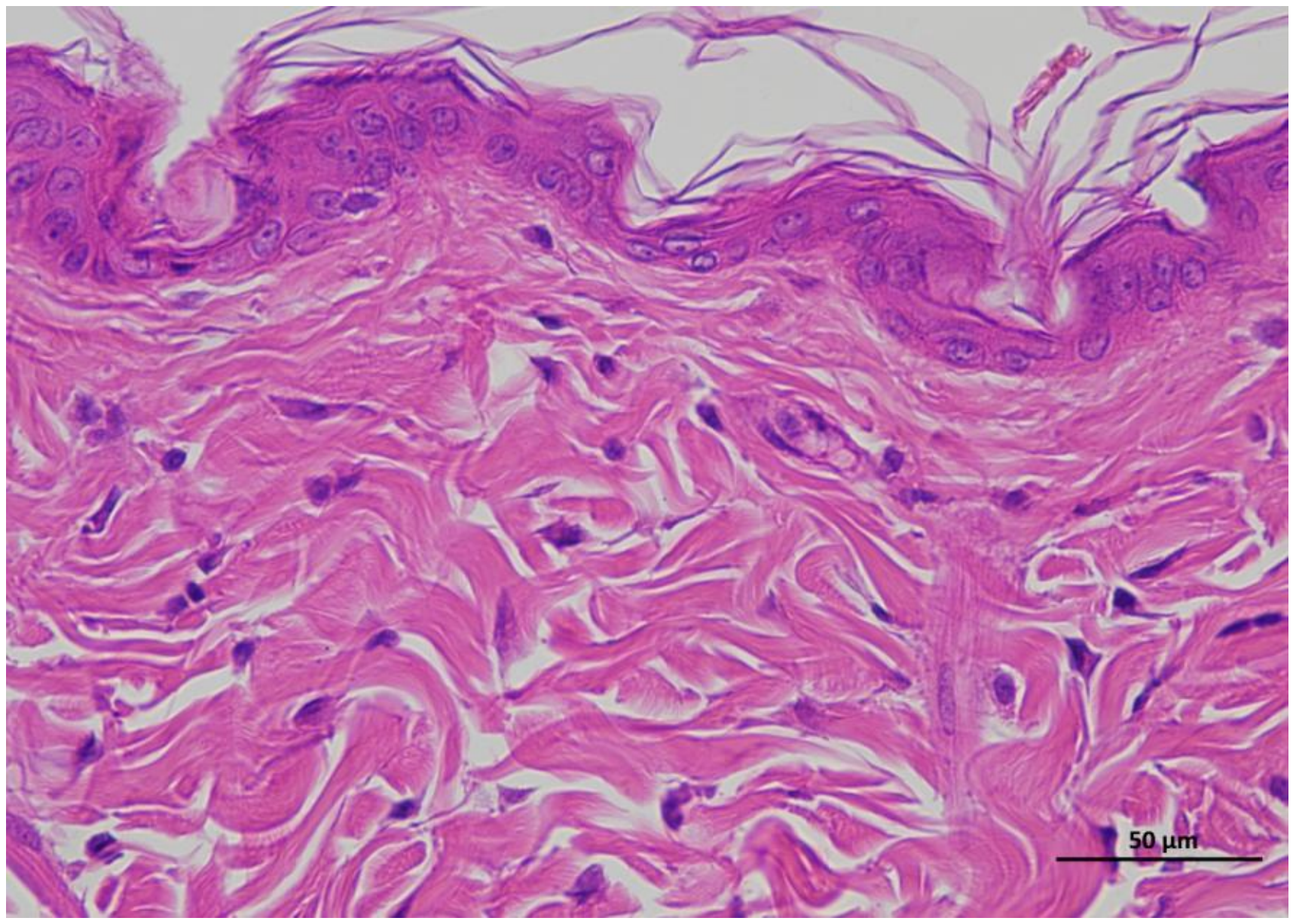

5 RNA-BEV-L 0day 400-1

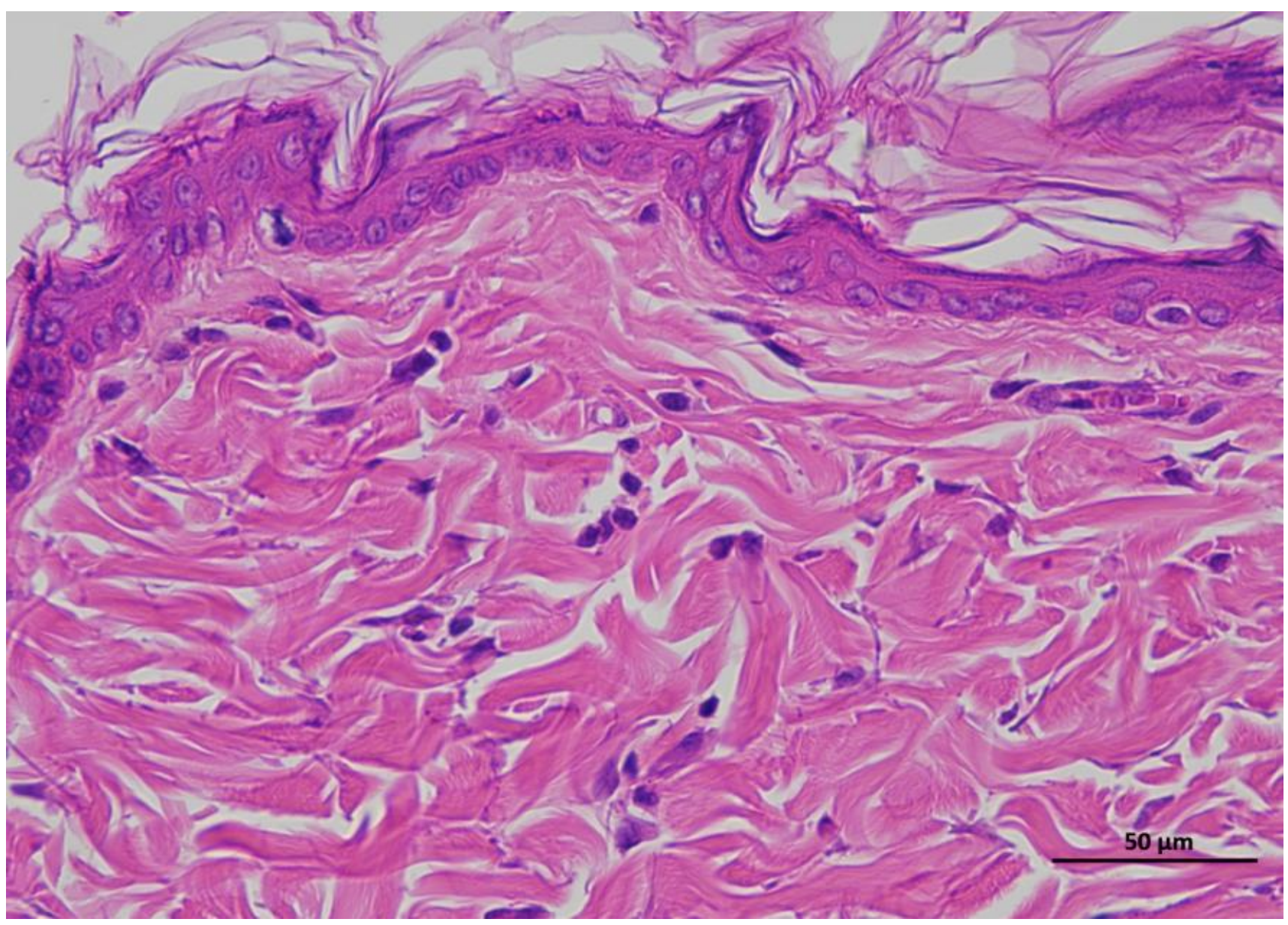

5 RNA-BEV-L 0day 400-2

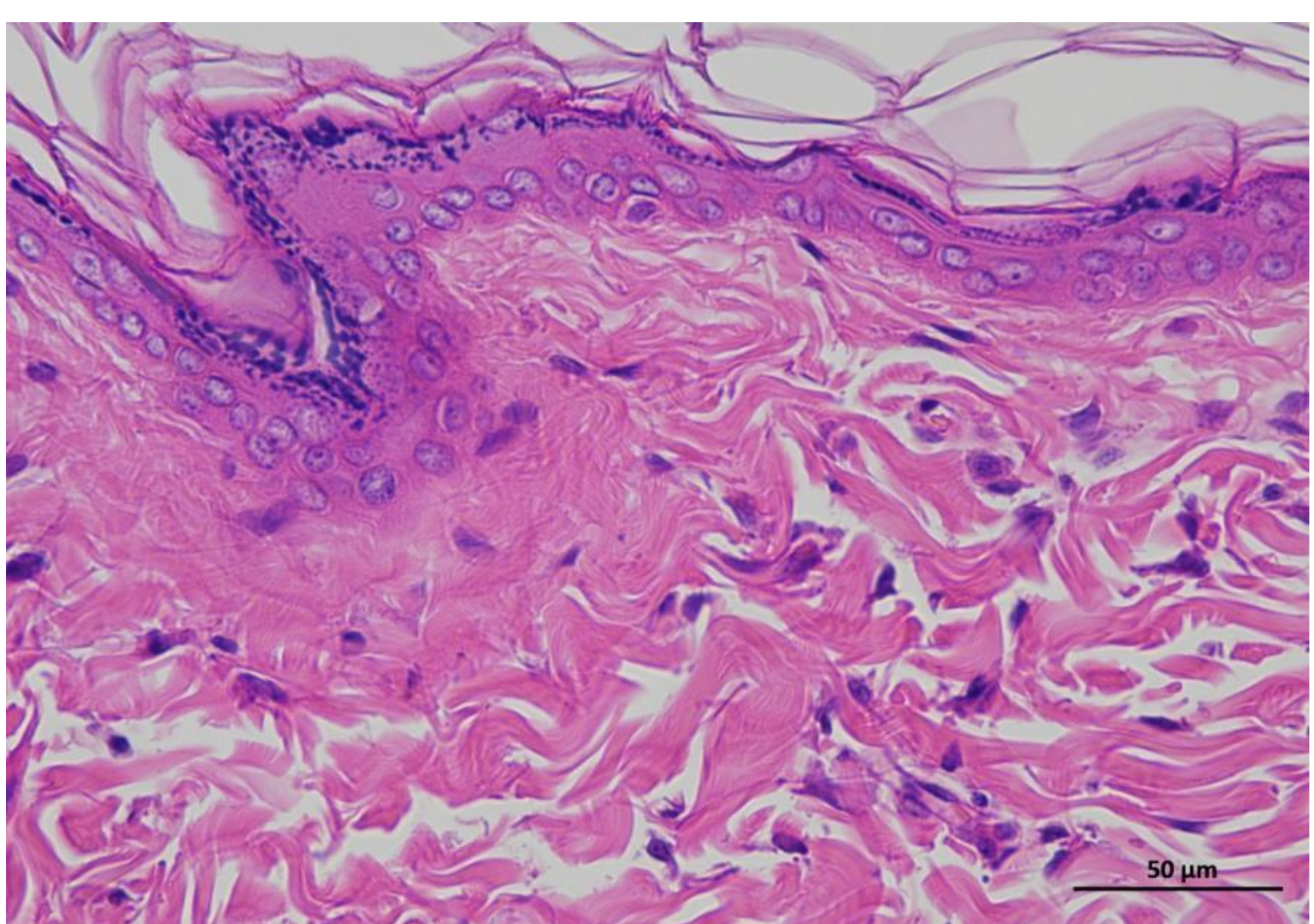

5 RNA-BEV-L 0day 400-3

Supplementary Figure 2e| Representative H&E staining of skin tissue of mRNA RNA-BEV-L animals / Day 0.

Animal 6

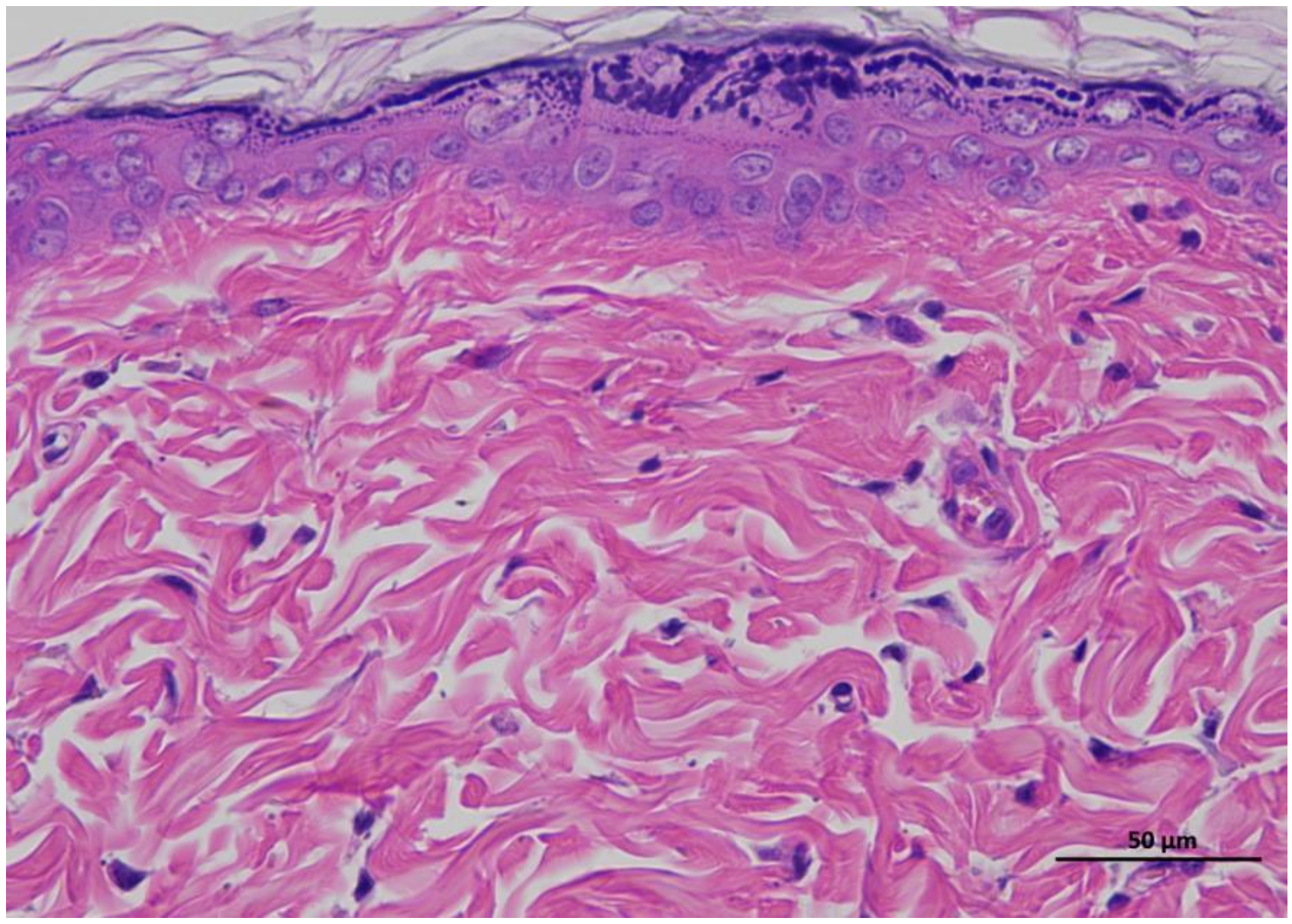

6 RNA-BEV-L 0day 400-1

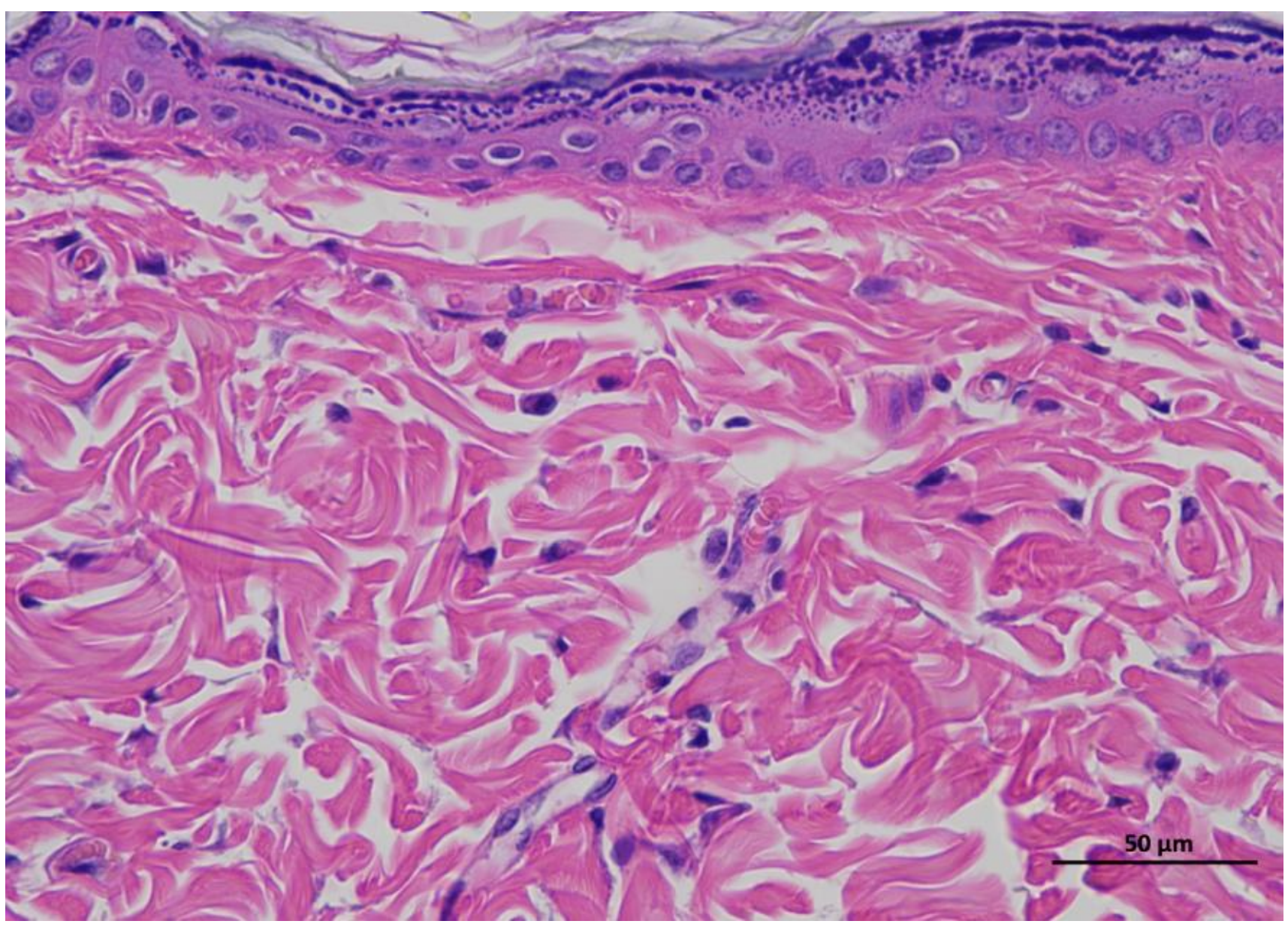

6 RNA-BEV-L 0day 400-2

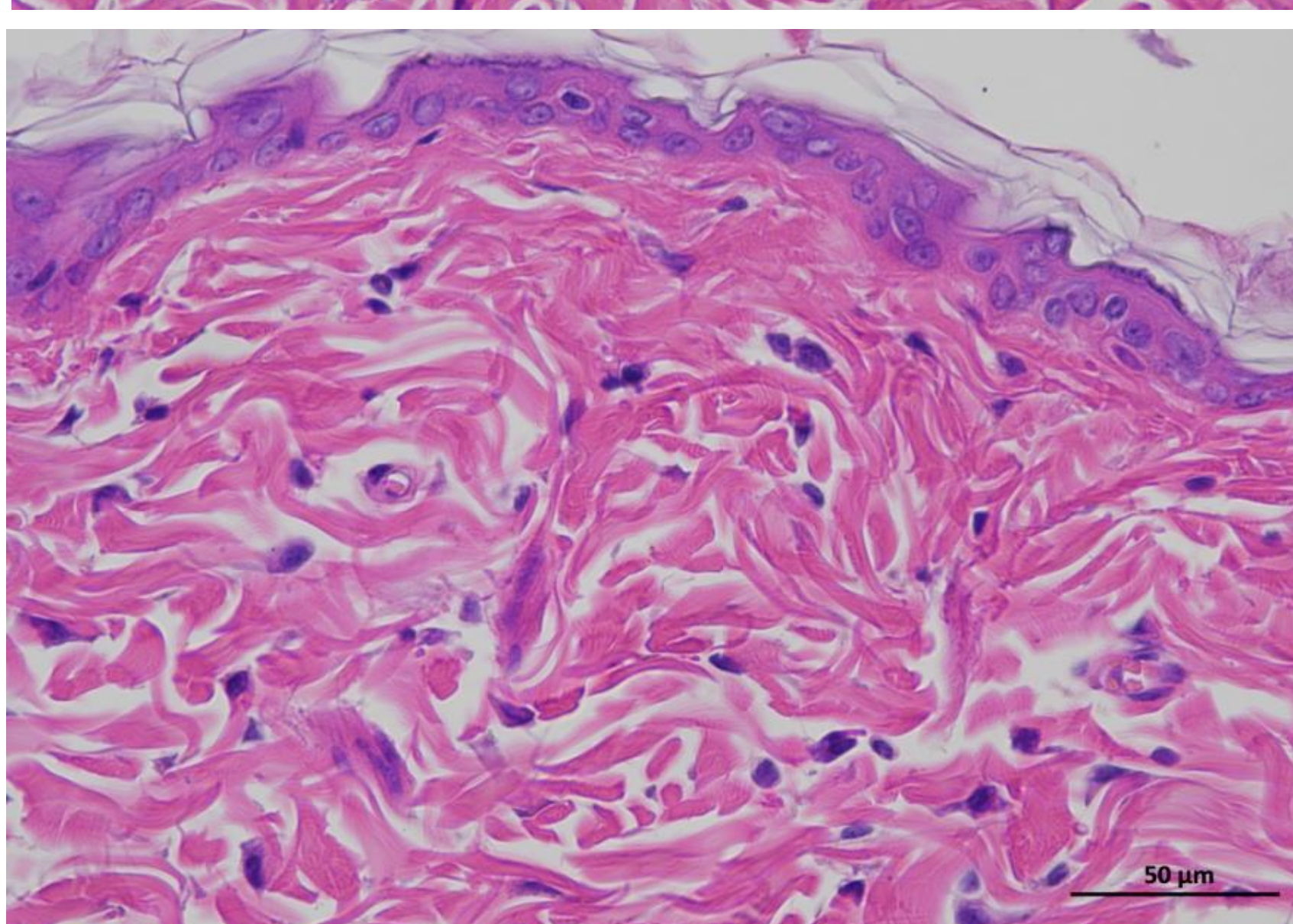

6 RNA-BEV-L 0day 400-3

Supplementary Figure 3e | Representative H&E staining of skin tissue of RNA-BEV-L animals / Day 14.

Representative hematoxylin and eosin (H&E)–stained section of skin tissue collected from experimental rat. The image shows the typical architecture of the skin, including the epidermal layer with stratified keratinized epithelium and the underlying dermis composed of dense collagen fibers and connective tissue structures.
(n = 6 per group).

Animal 1

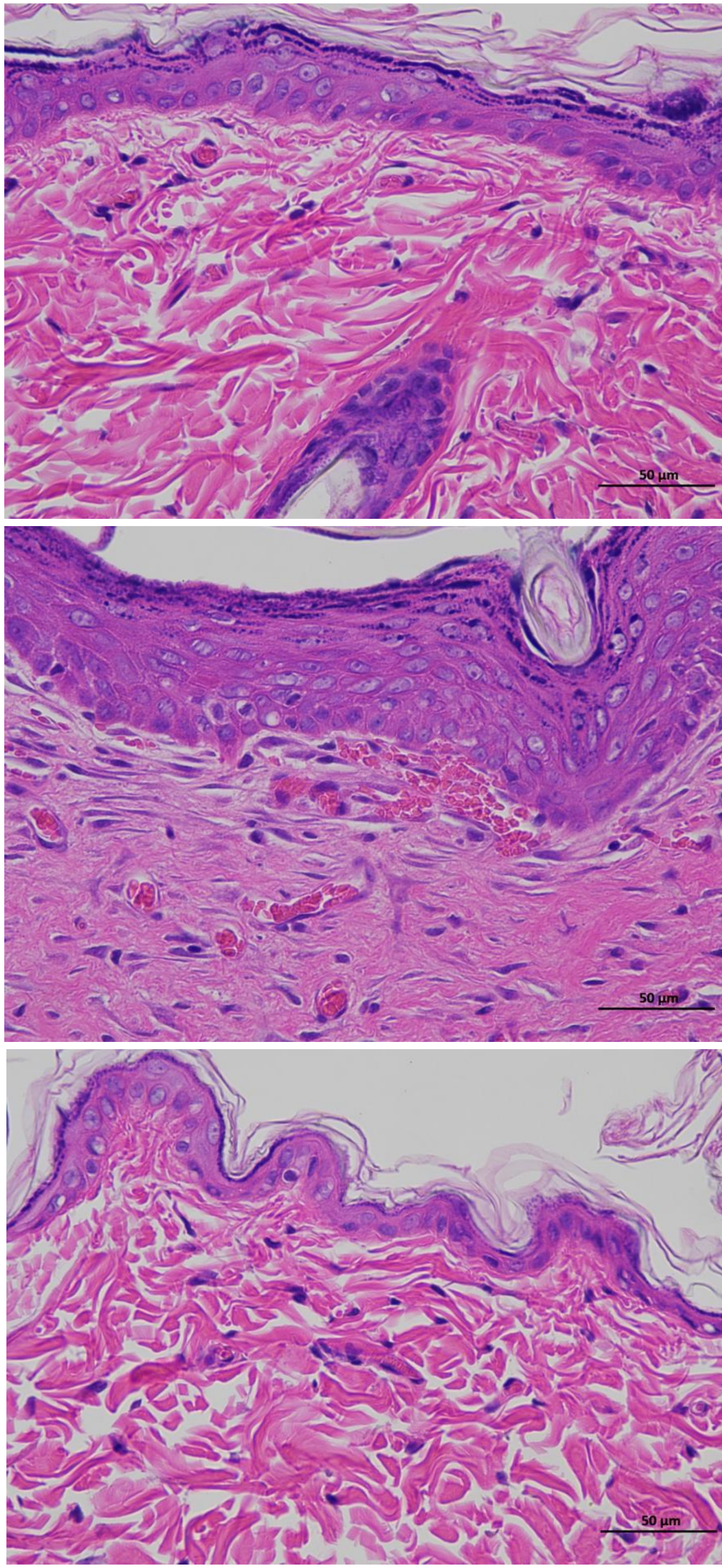

1 RNA-BEV-L 14day 400-1

1 RNA-BEV-L 14day 400-2

1 RNA-BEV-L 14day 400-3

Animal 2

Supplementary Figure 3e | Representative H&E staining of skin tissue of RNA-BEV-L animals / Day 14.

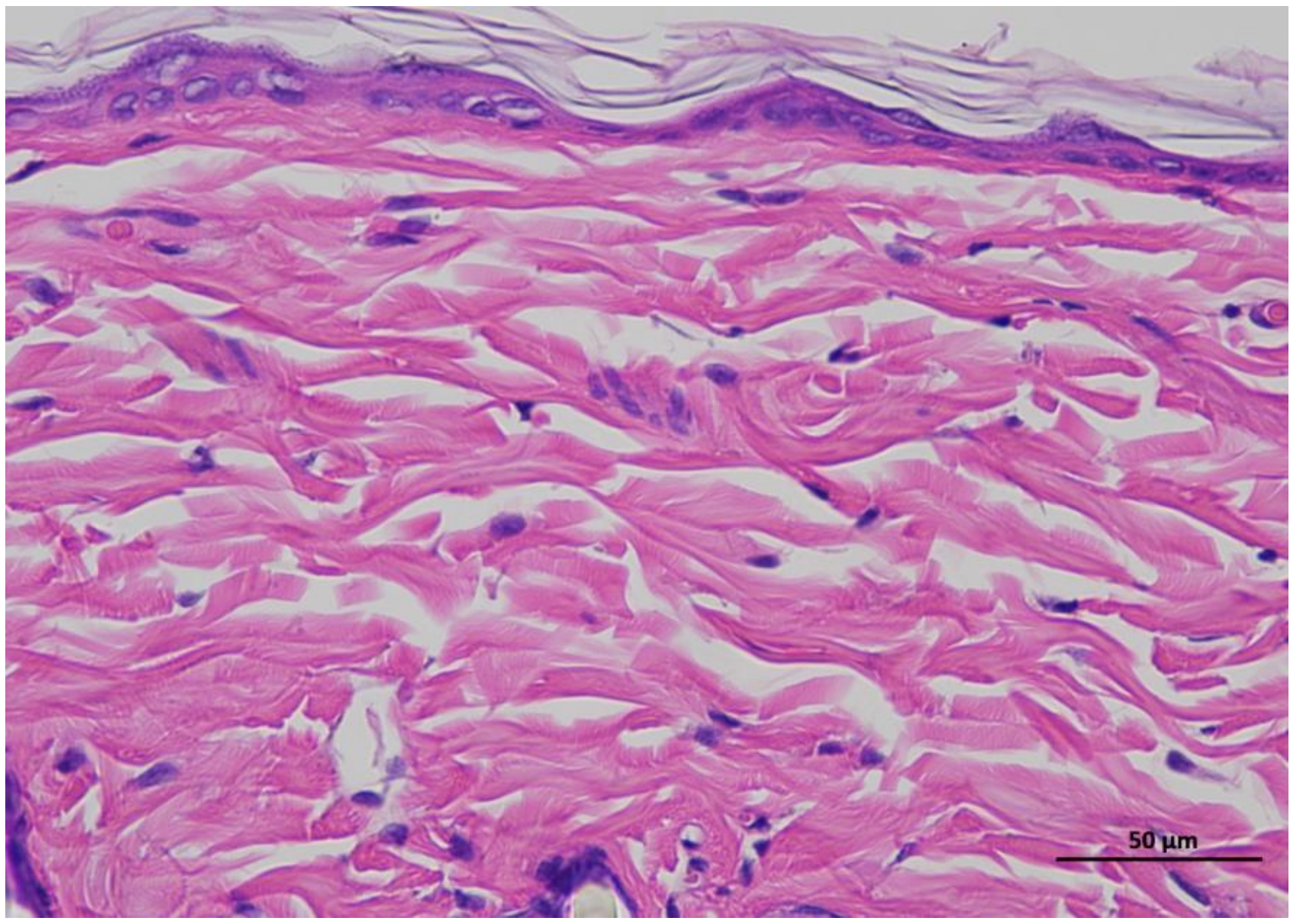

2 RNA-BEV-L 14day 400-1

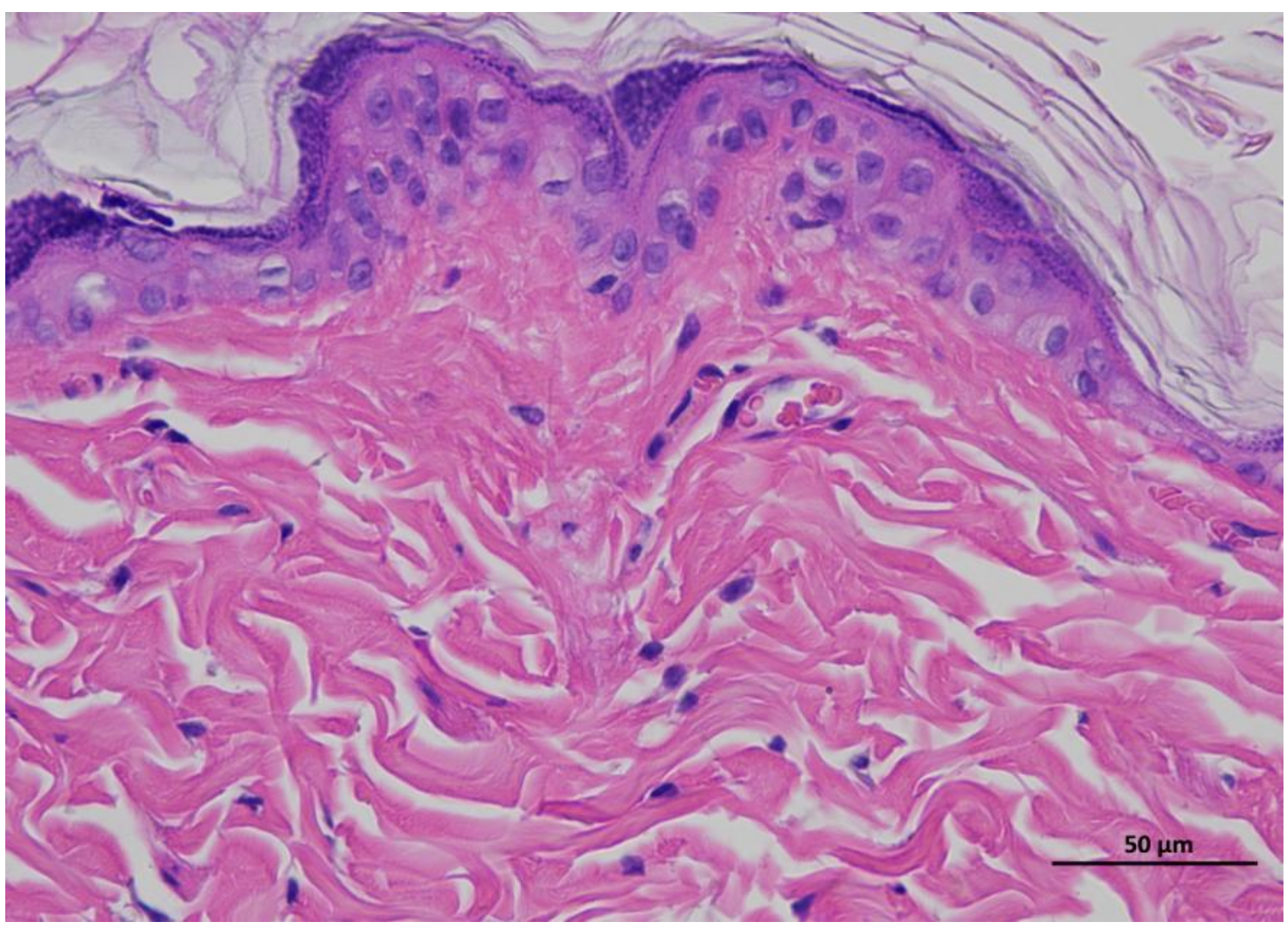

2 RNA-BEV-L 14day 400-2

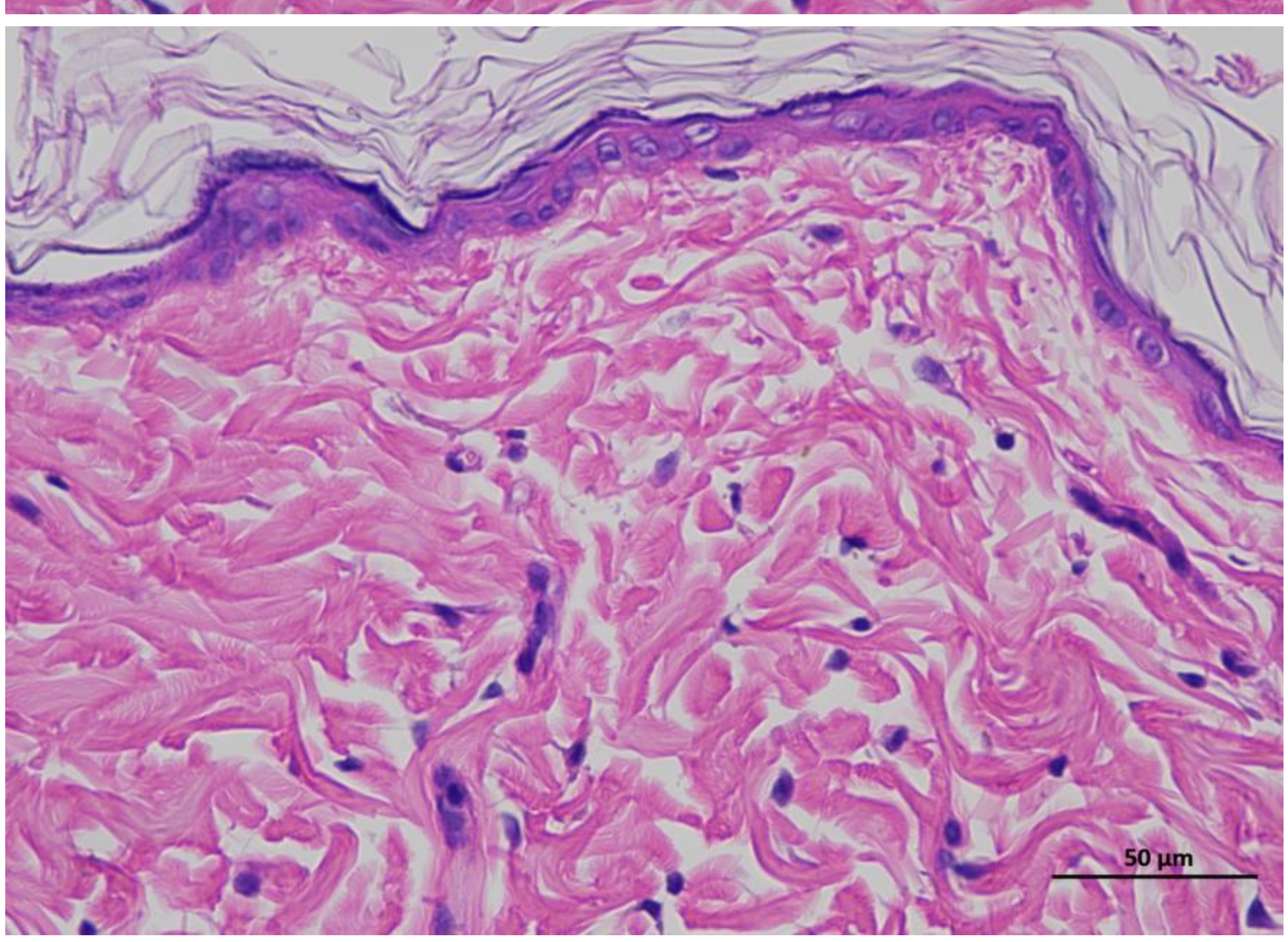

2 RNA-BEV-L 14day 400-3

Supplementary Figure 3e | Representative H&E staining of skin tissue of RNA-BEV-L animals / Day 14.

Animal 3

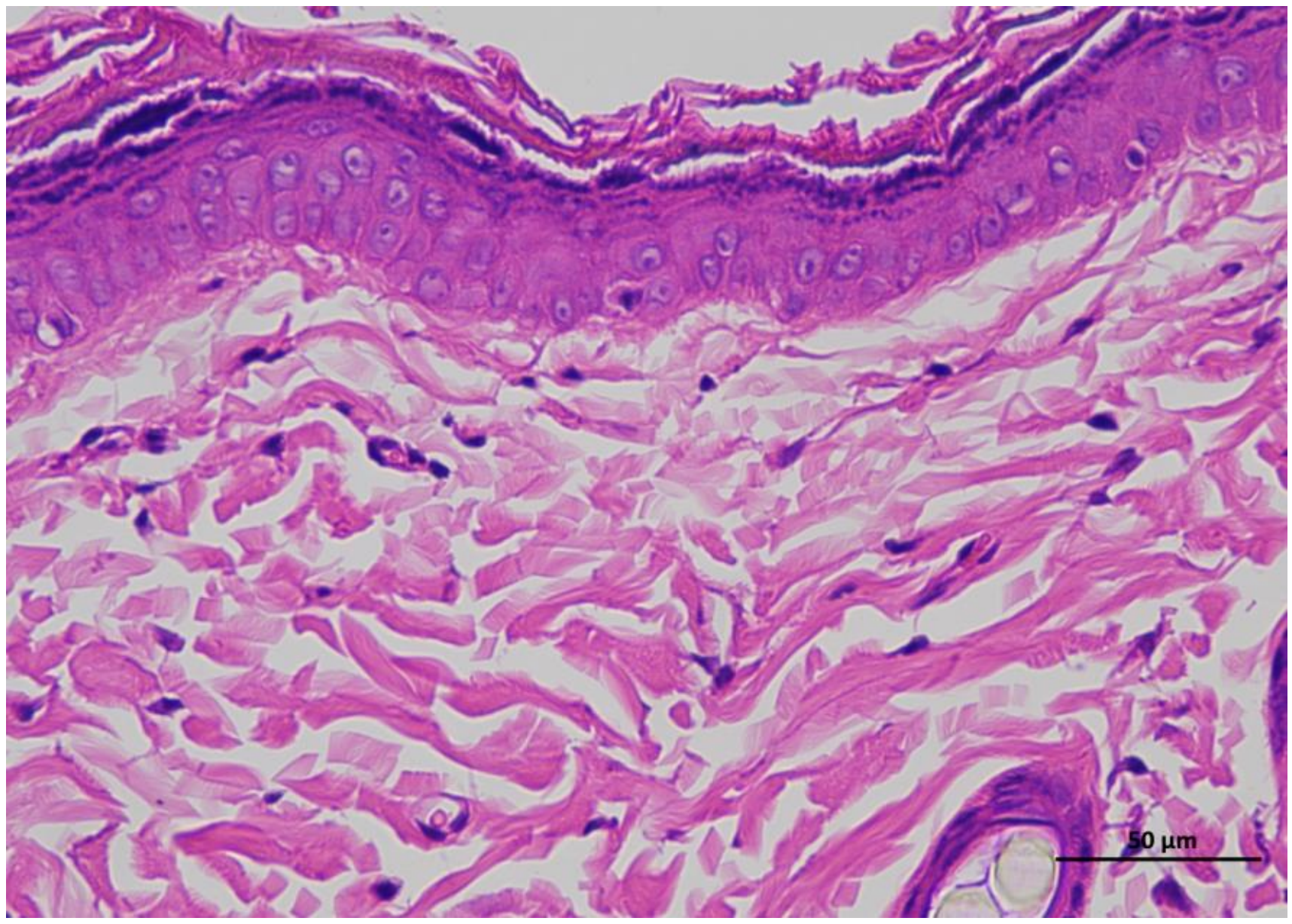

3 RNA-BEV-L 14day 400-1

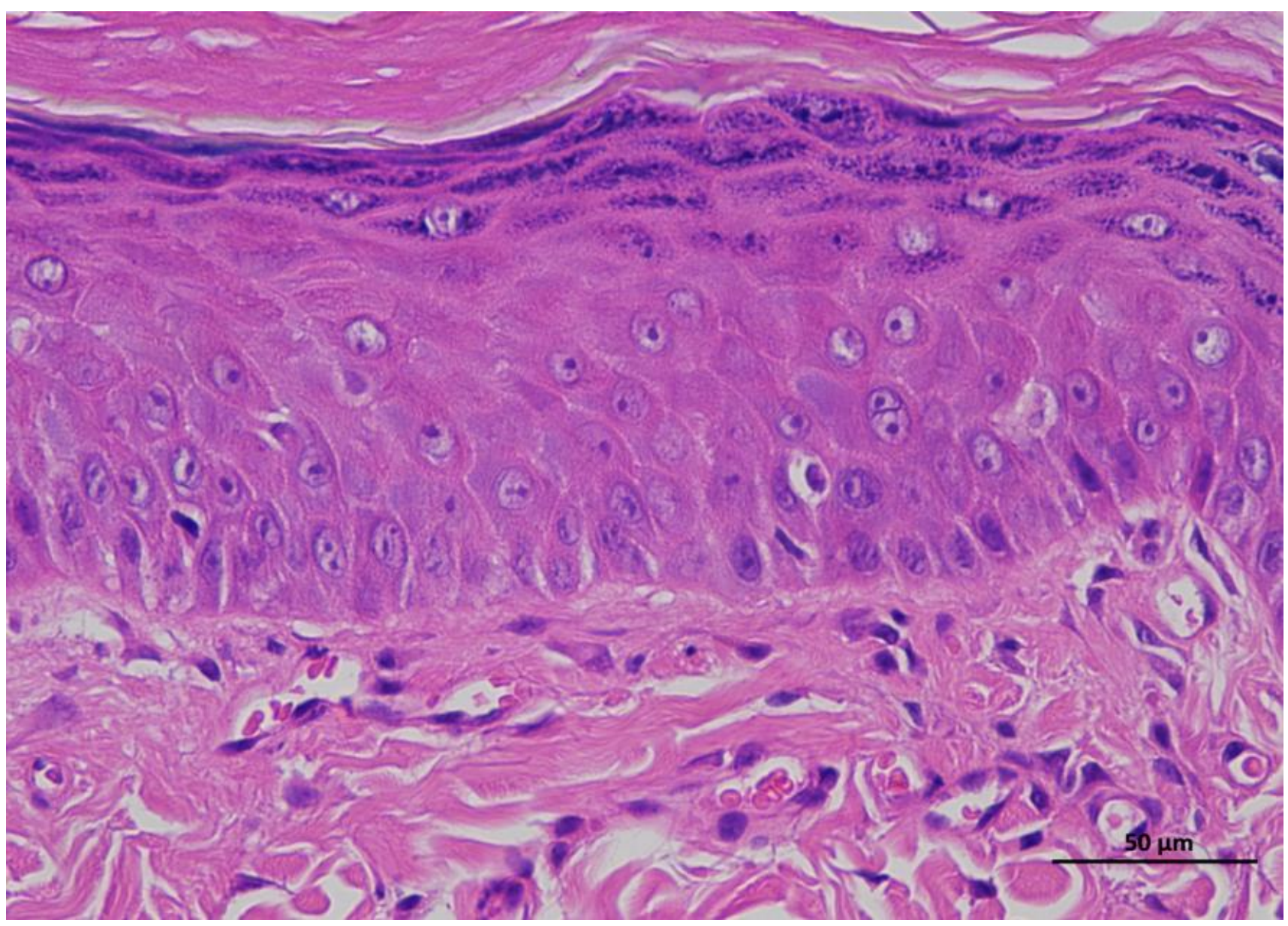

3 RNA-BEV-L 14day 400-2

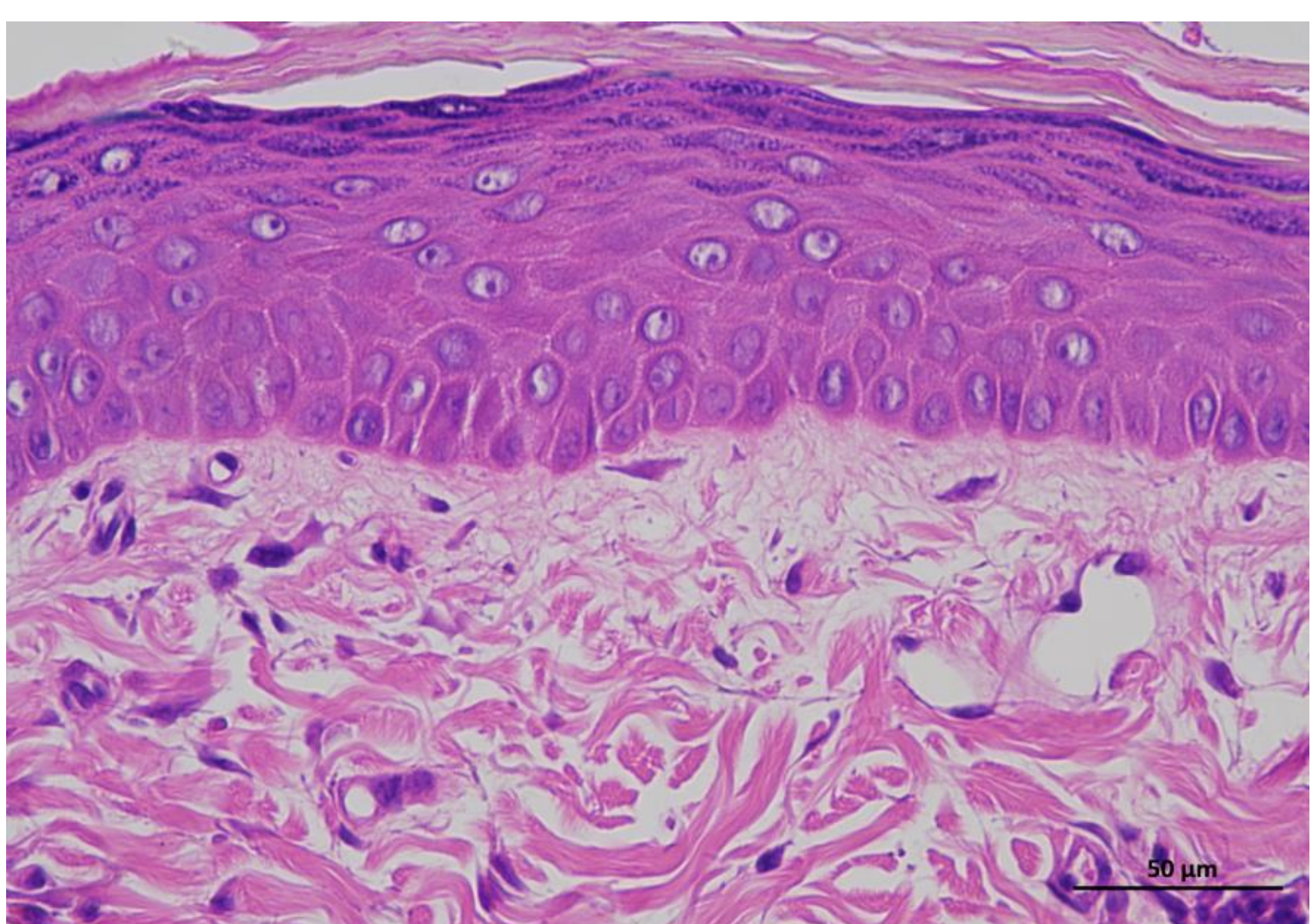

3 RNA-BEV-L 14day 400-3

Animal 4

Supplementary Figure 3e | Representative H&E staining of skin tissue of RNA-BEV-L animals / Day 14.

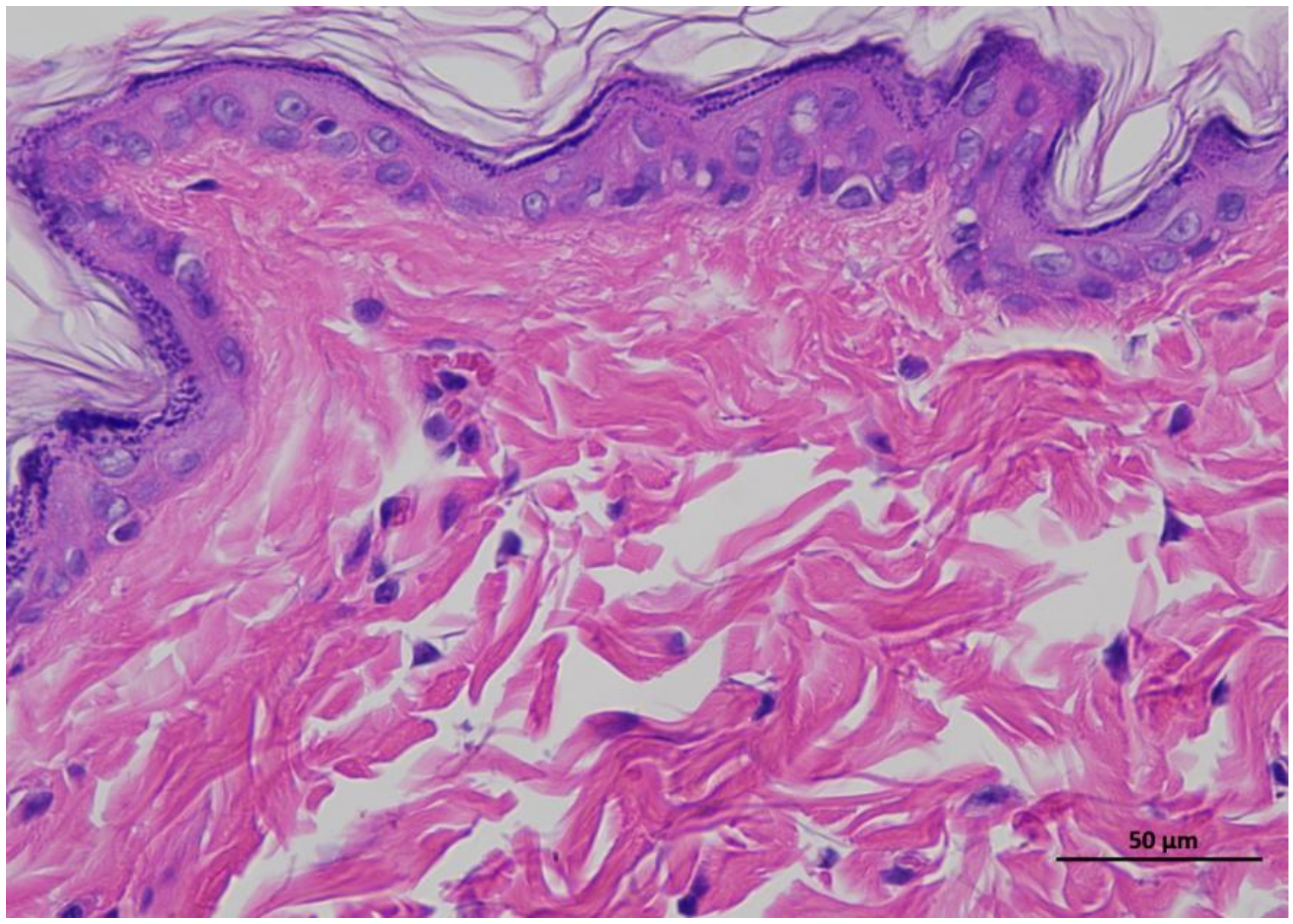

4 RNA-BEV-L 14day 400-1

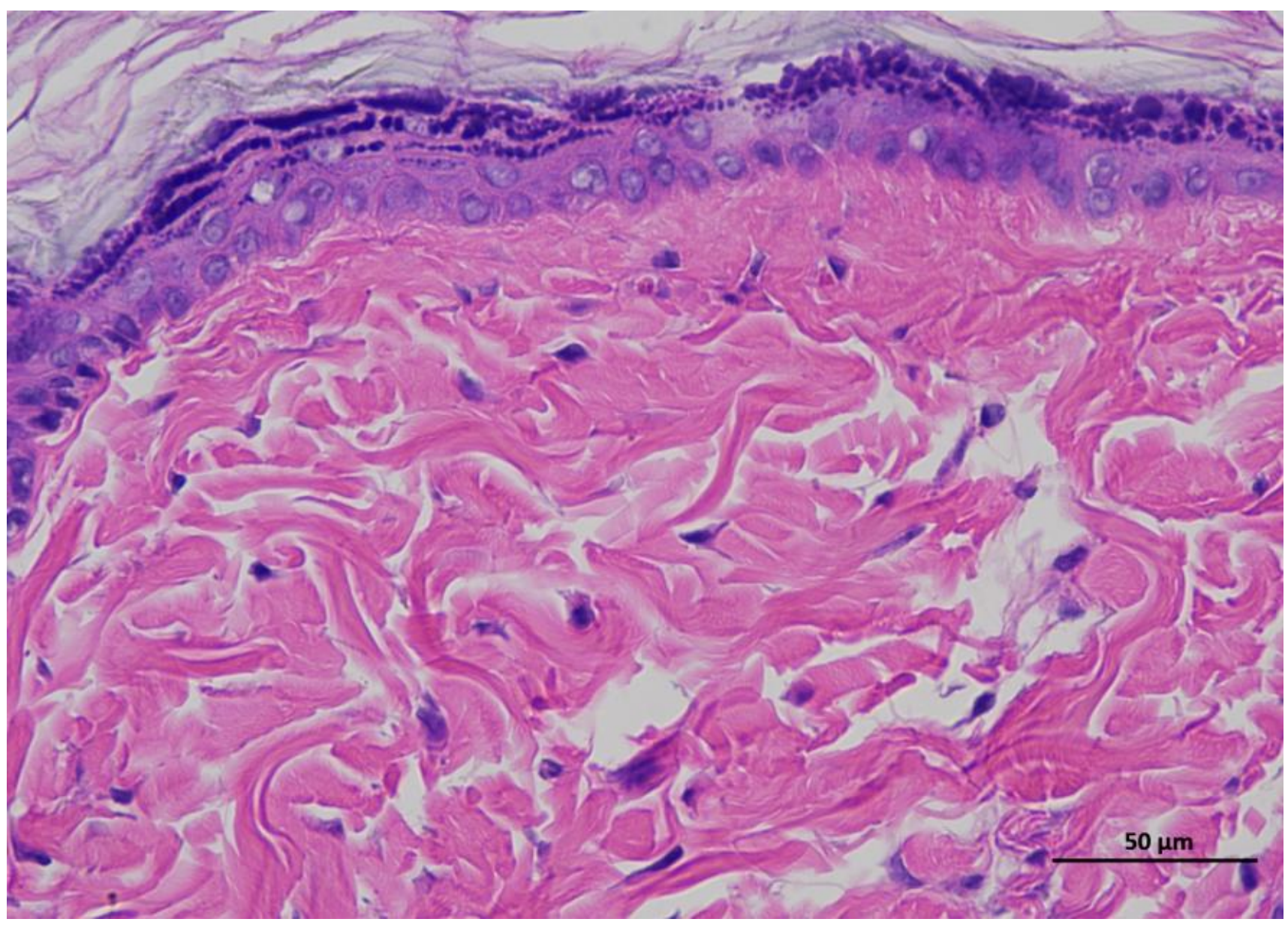

4 RNA-BEV-L 14day 400-2

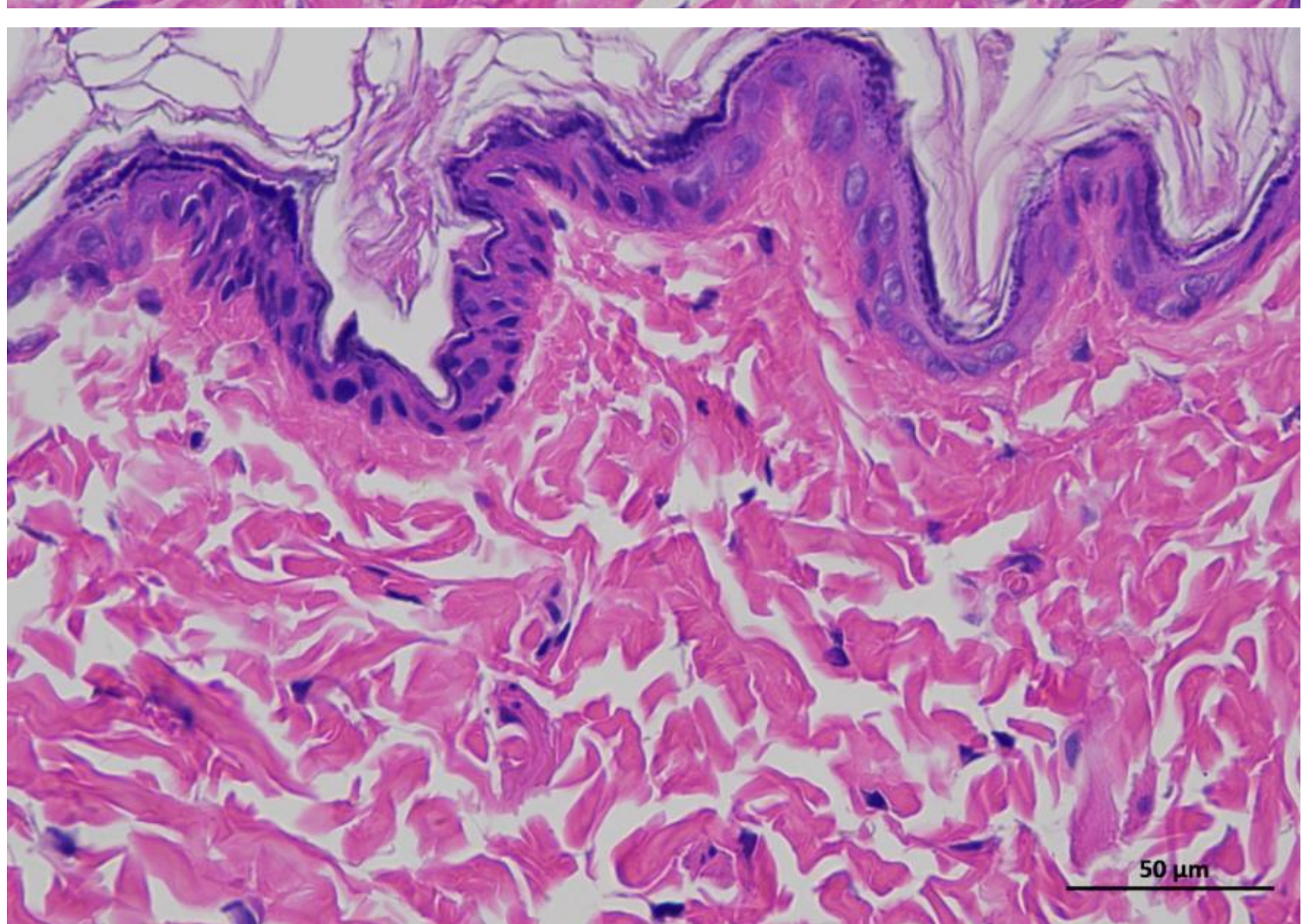

4 RNA-BEV-L 14day 400-3

Supplementary Figure 3e | Representative H&E staining of skin tissue of RNA-BEV-L animals / Day 14.

Animal 5

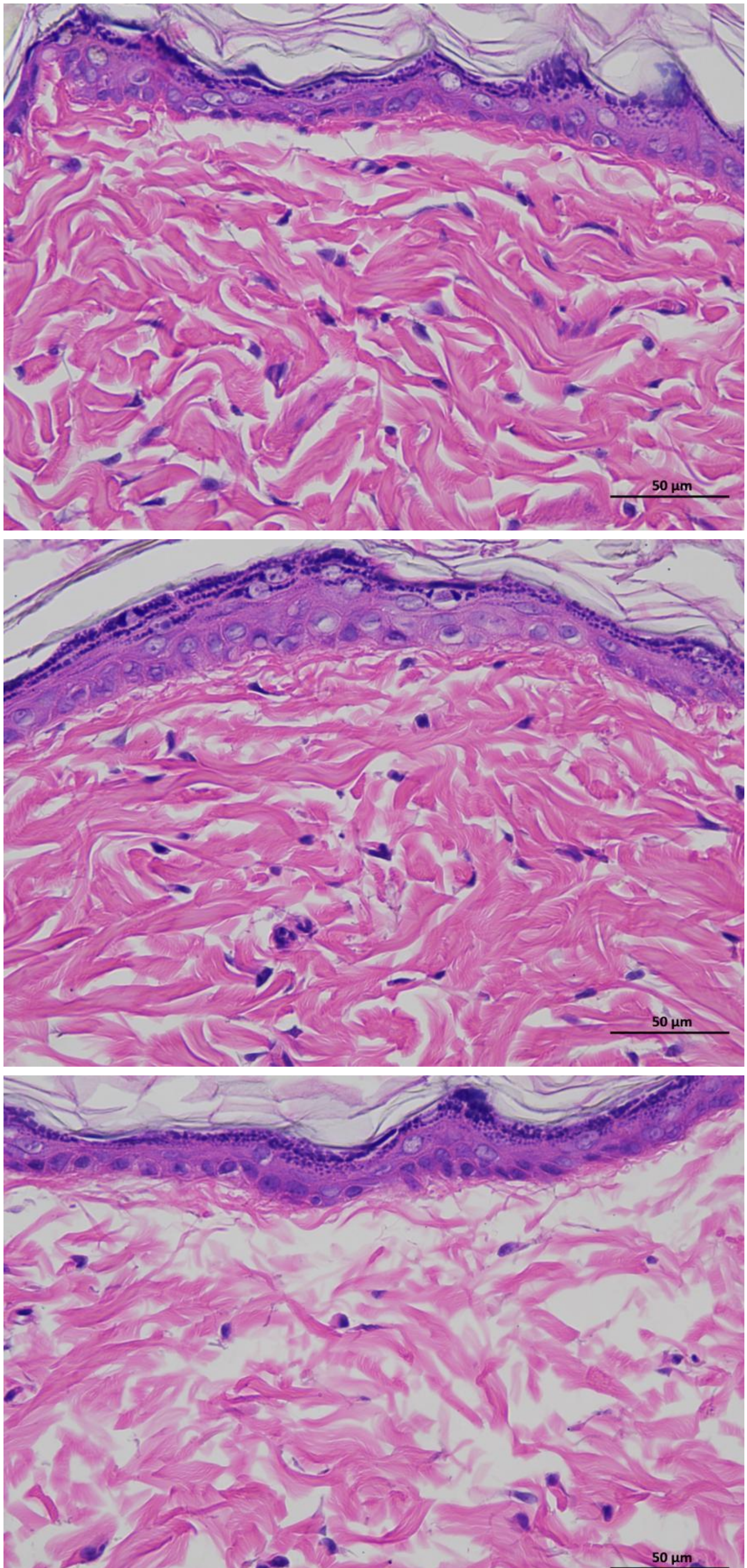

5 RNA-BEV-L 14day 400-1

5 RNA-BEV-L 14day 400-2

5 RNA-BEV-L 14day 400-3

Supplementary Figure 3e | Representative H&E staining of skin tissue of RNA-BEV-L animals / Day 14.

Animal 6

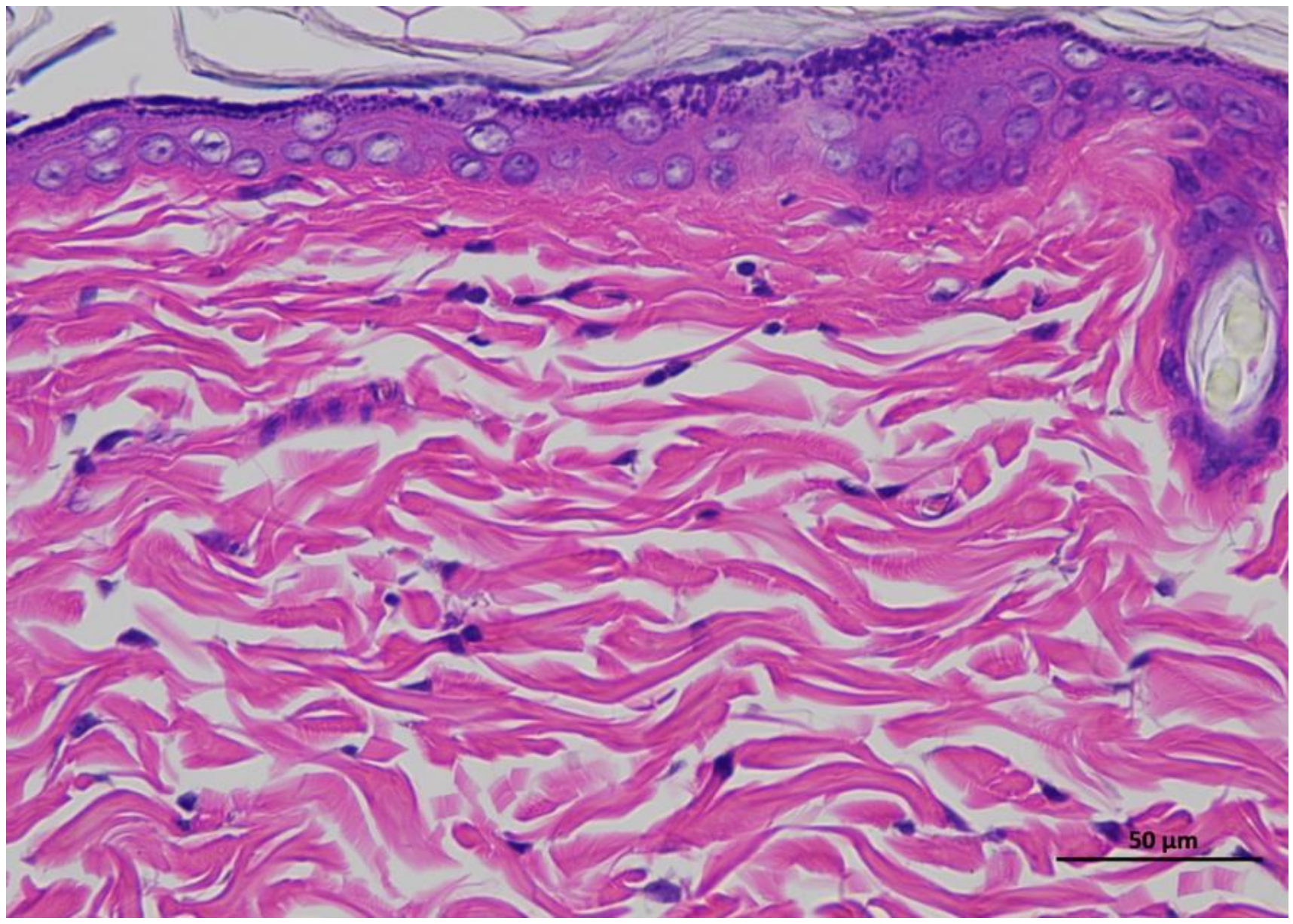

6 RNA-BEV-L 14day 400-1

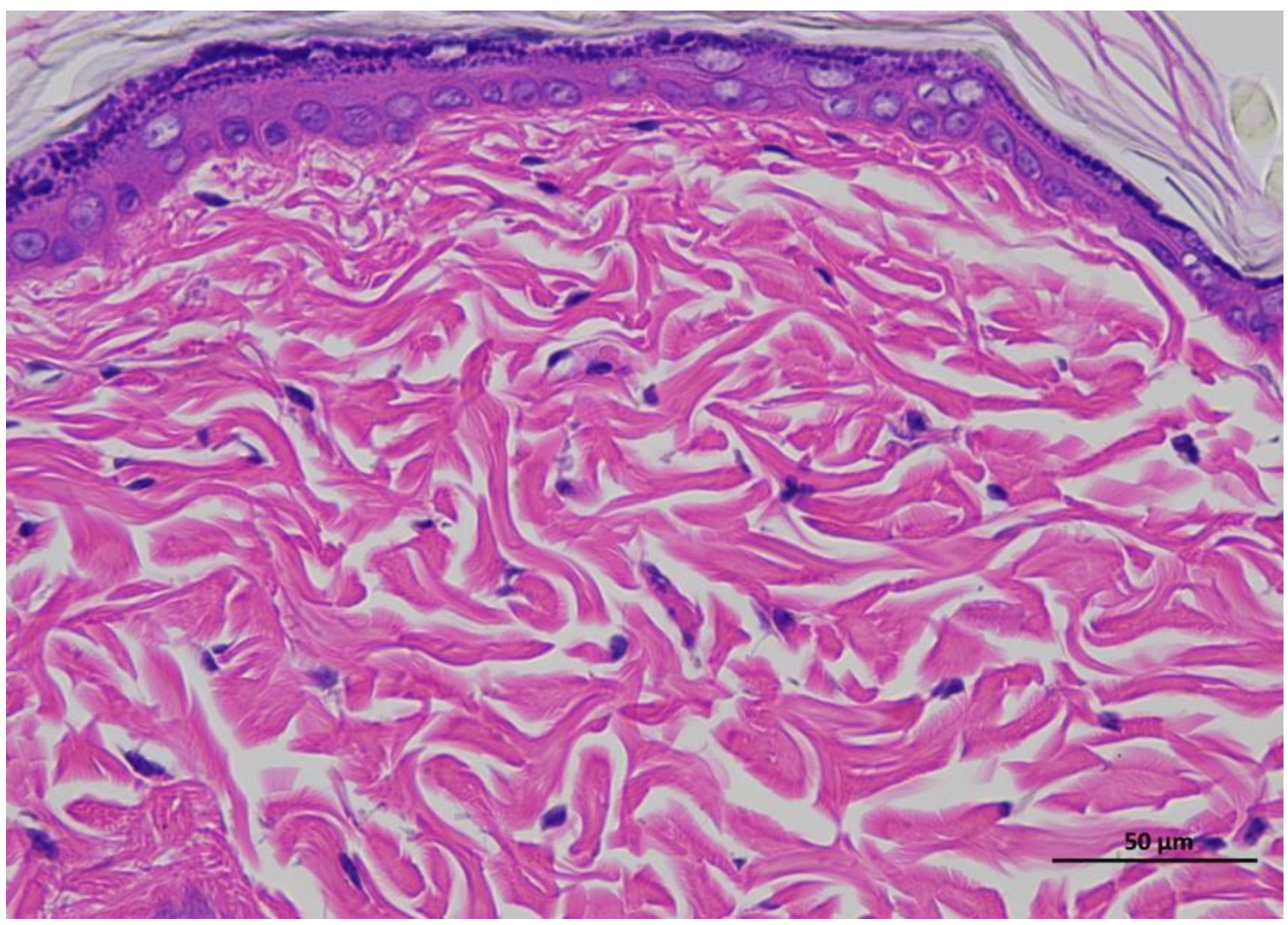

6 RNA-BEV-L 14day 400-2

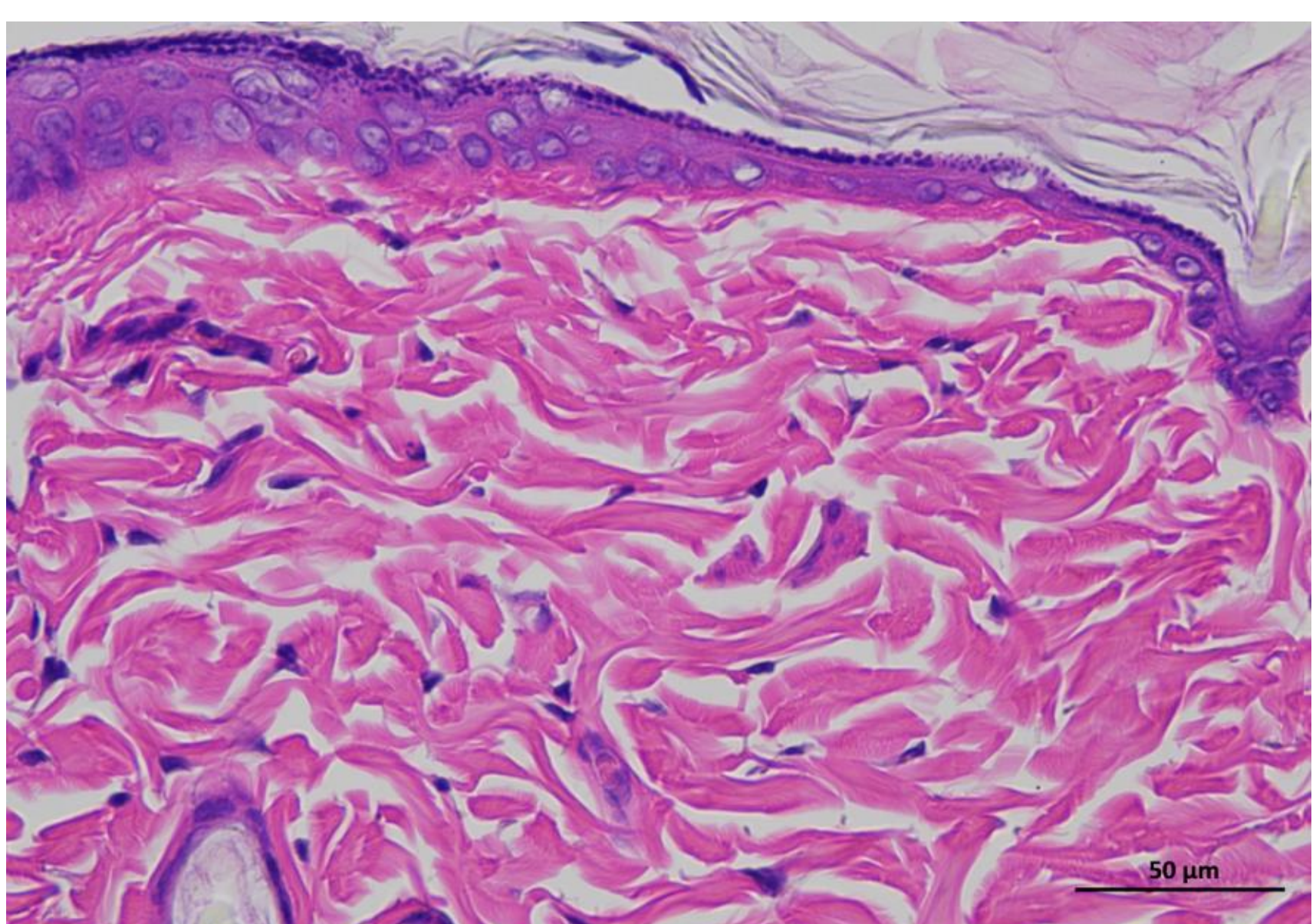

6 RNA-BEV-L 14day 400-3

Animal 1

Supplementary Figure 2f | Representative H&E staining of skin tissue of RNA-BEV-H animals / Day 0.
Representative hematoxylin and eosin (H&E)–stained section of skin tissue collected from experimental rat. The image shows the typical architecture of the skin, including the epidermal layer with stratified keratinized epithelium and the underlying dermis composed of dense collagen fibers and connective tissue structures.
(n = 6 per group).

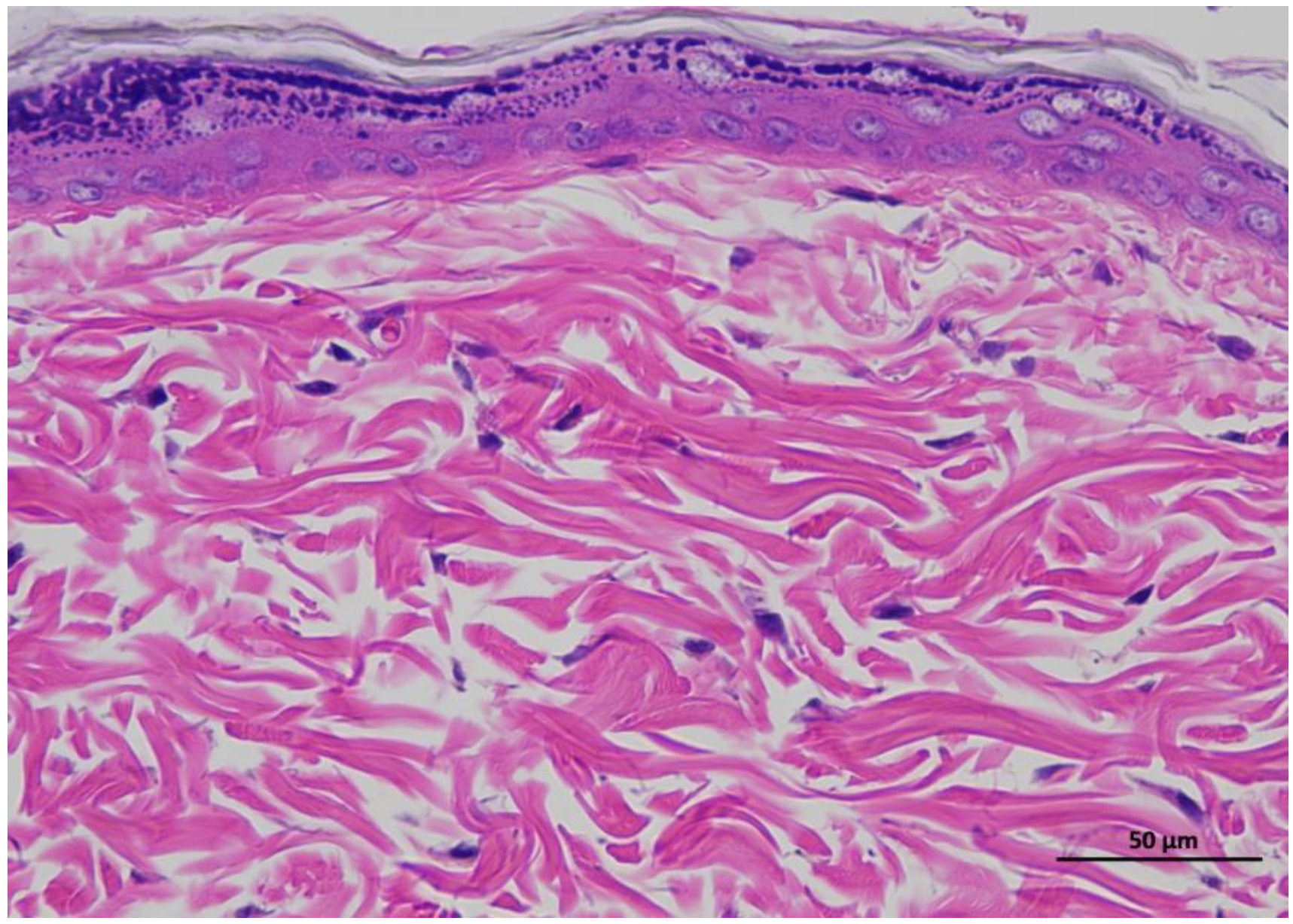

1 RNA-BEV-H 0day 400-1

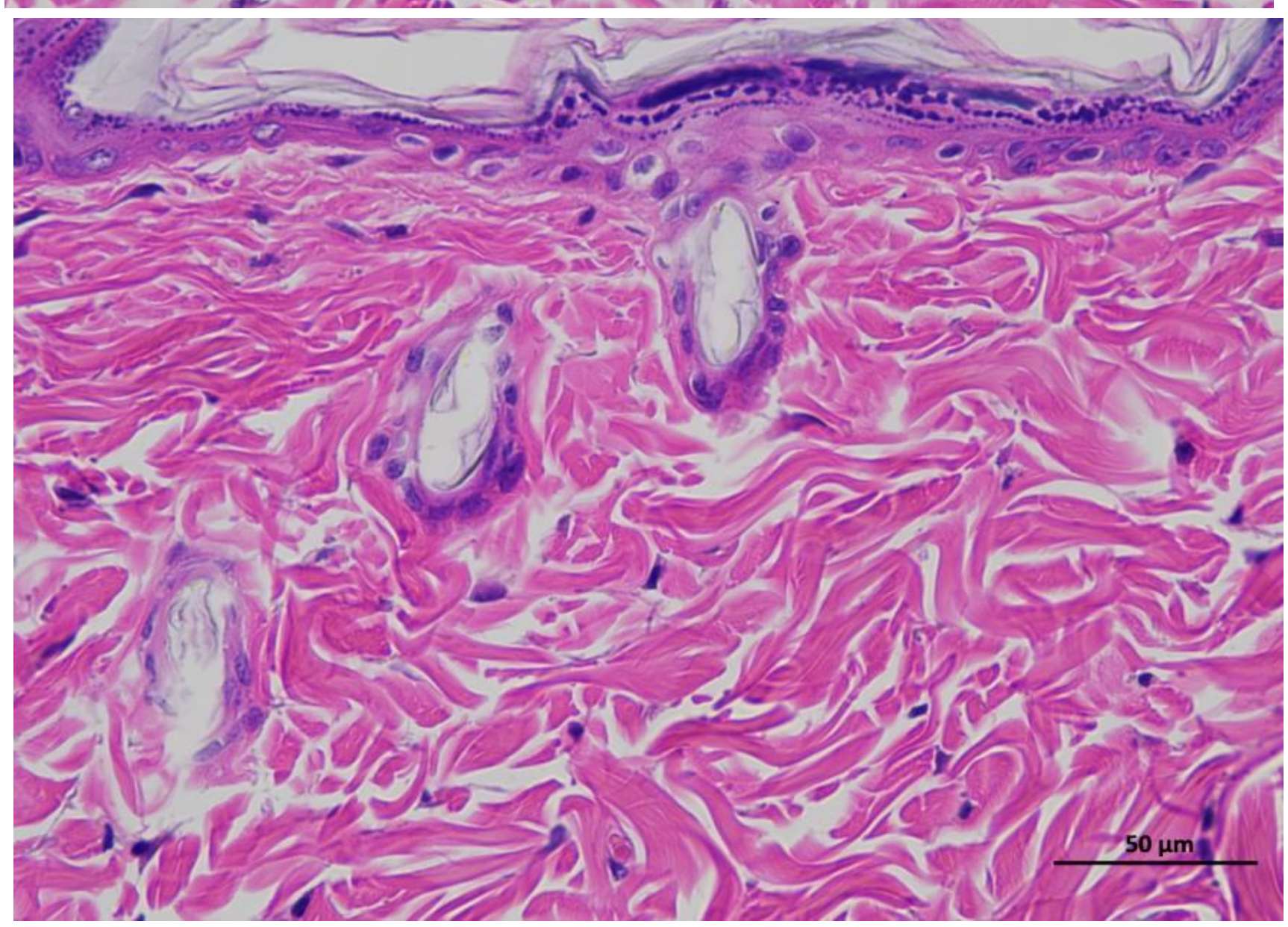

1 RNA-BEV-H 0day 400-2

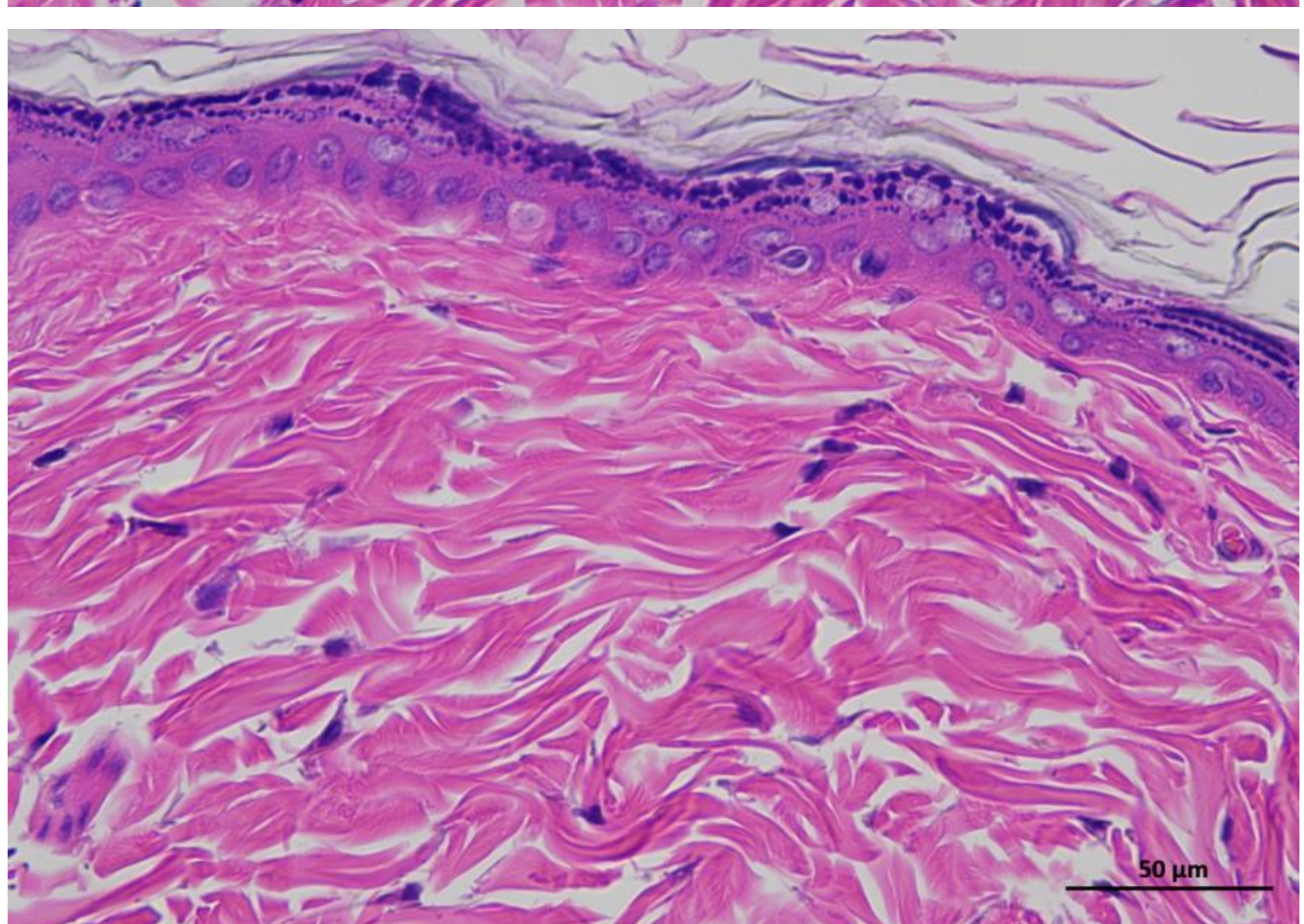

1 RNA-BEV-H 0day 400-3

Supplementary Figure 2f| Representative H&E staining of skin tissue of mRNA RNA-BEV-H animals / Day 0.

Animal 2

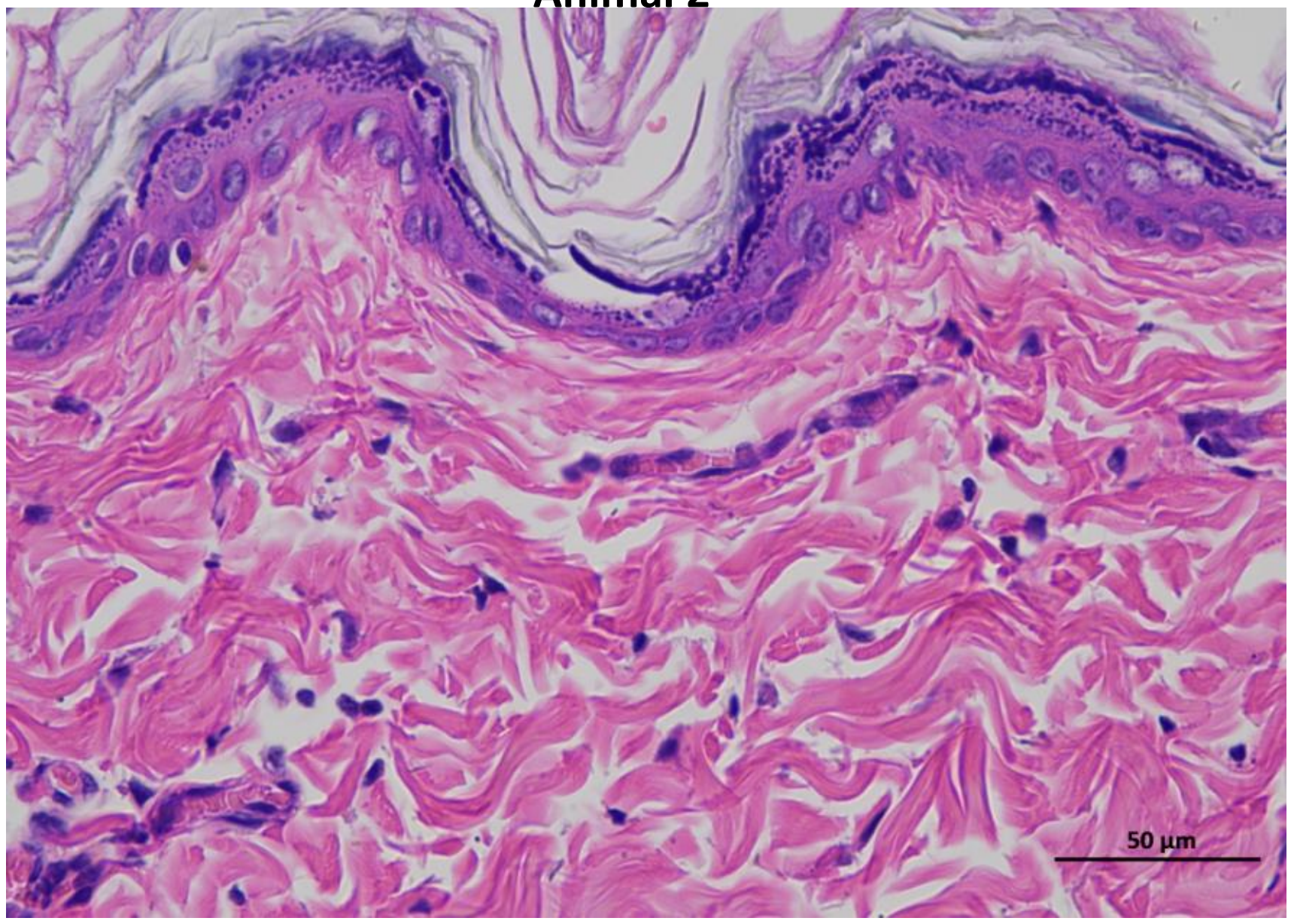

2 RNA-BEV-H 0day 400-1

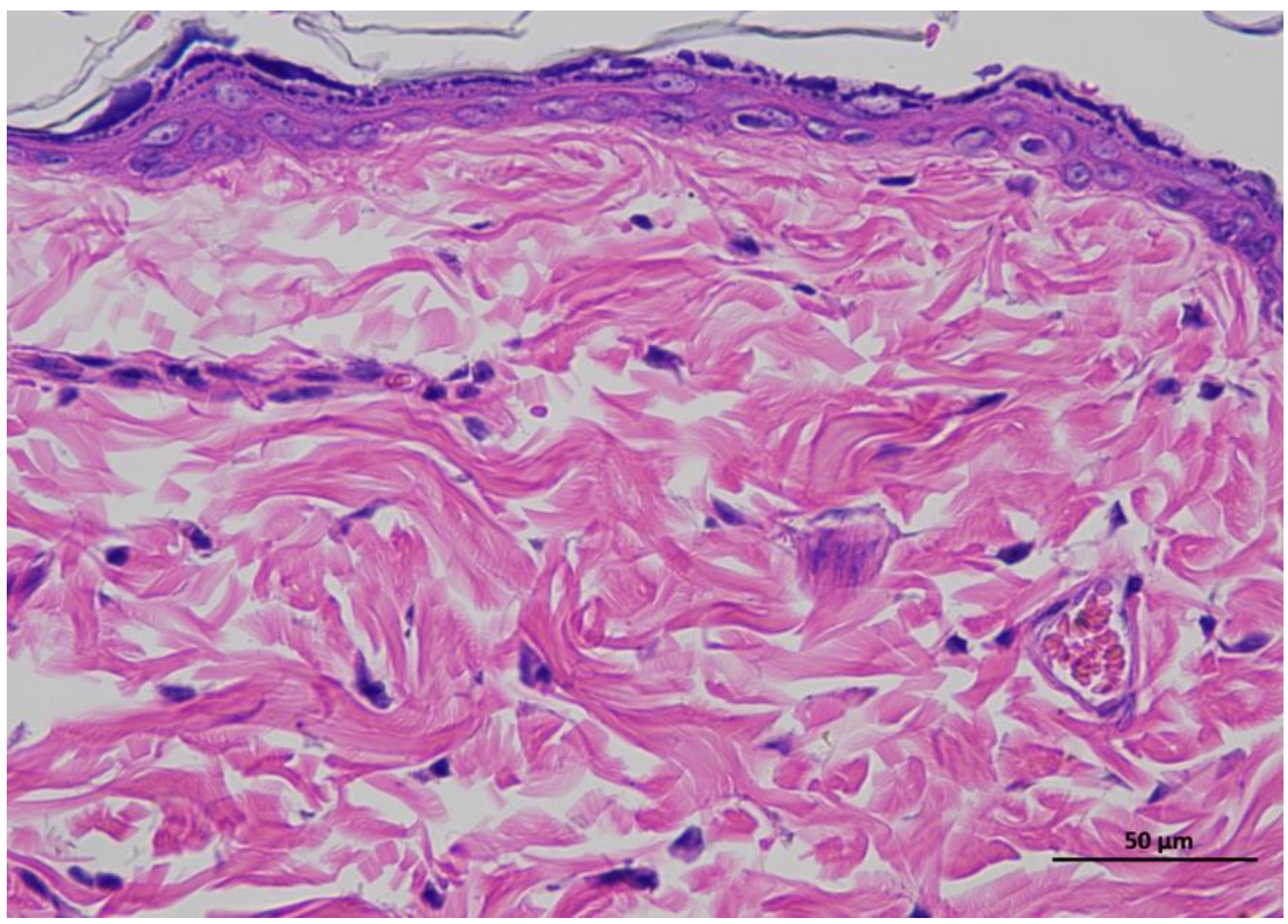

2 RNA-BEV-H 0day 400-2

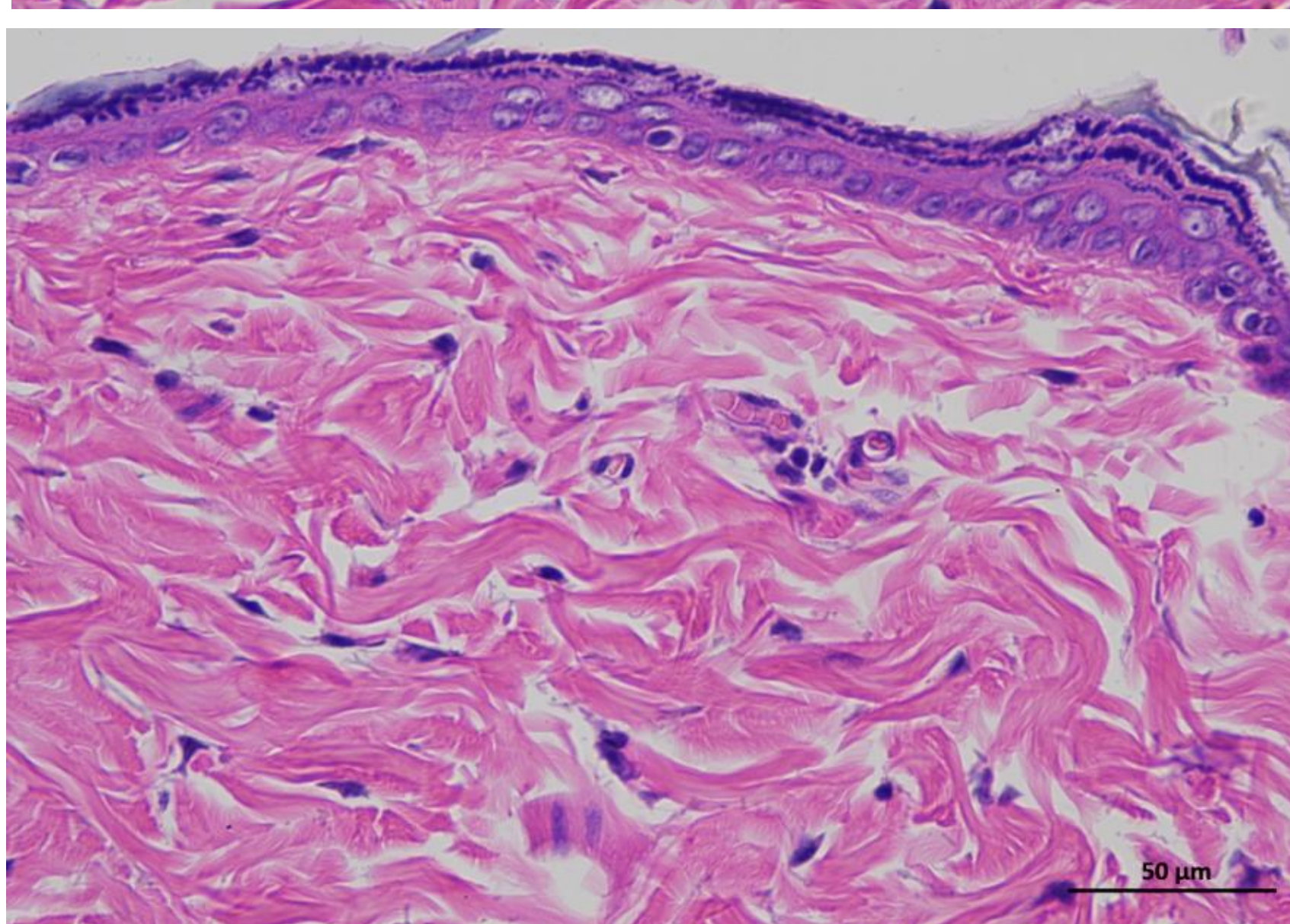

2 RNA-BEV-H 0day 400-3

Animal 3

Supplementary Figure 2f| Representative H&E staining of skin tissue of mRNA RNA-BEV-H animals / Day 0.

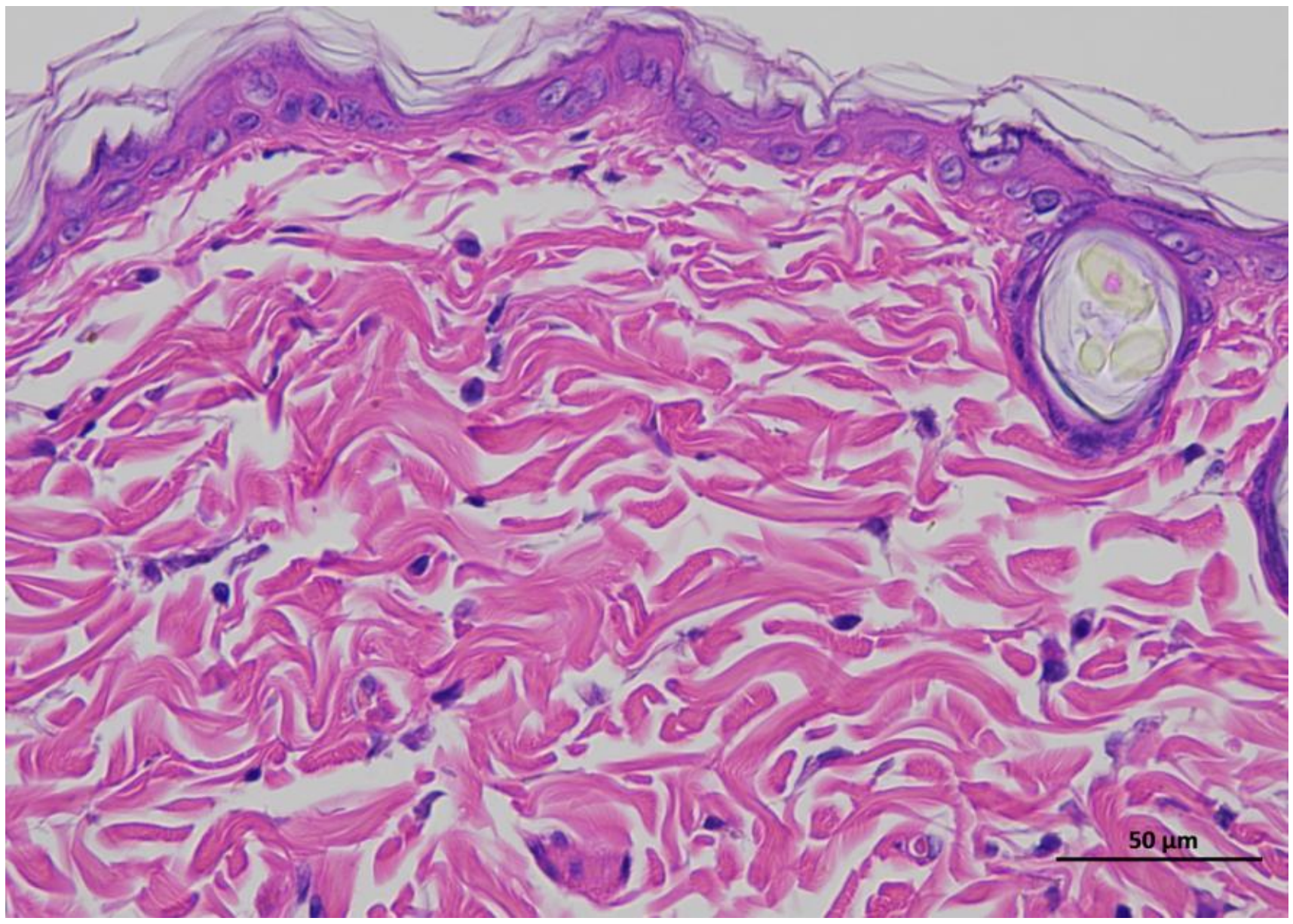

3 RNA-BEV-H 0day 400-1

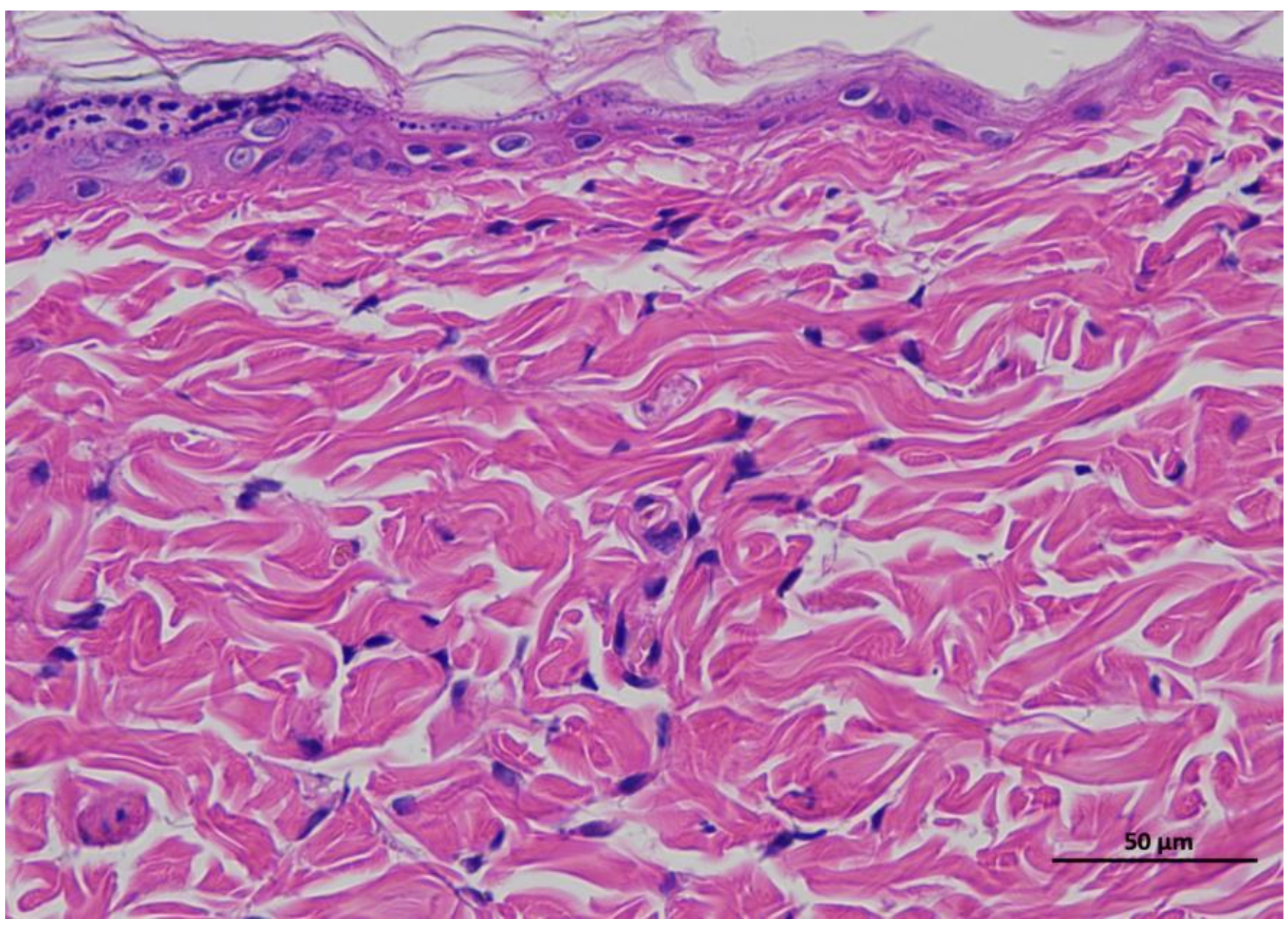

3 RNA-BEV-H 0day 400-2

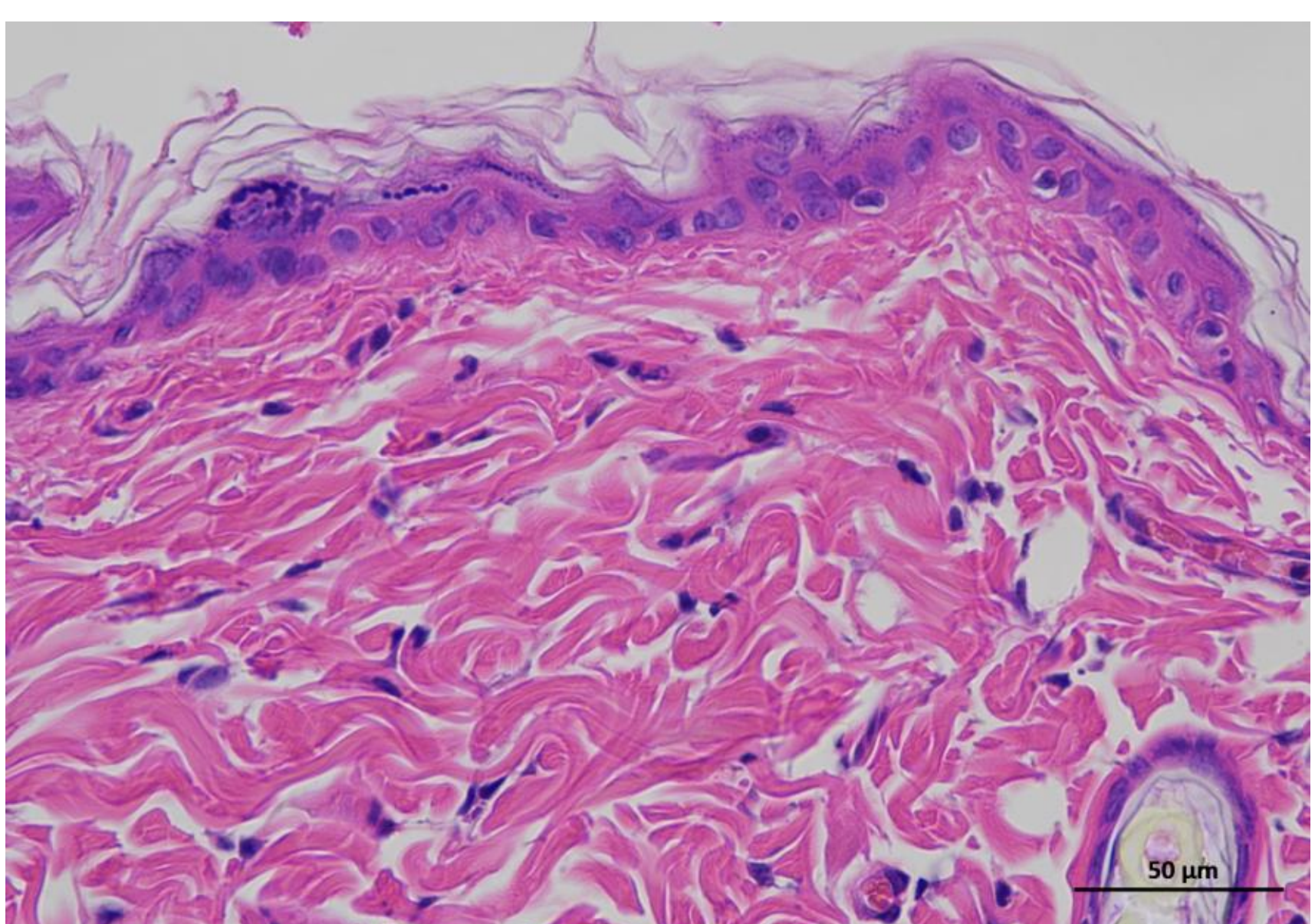

3 RNA-BEV-H 0day 400-3

Animal 4

Supplementary Figure 2f| Representative H&E staining of skin tissue of mRNA RNA-BEV-H animals / Day 0.

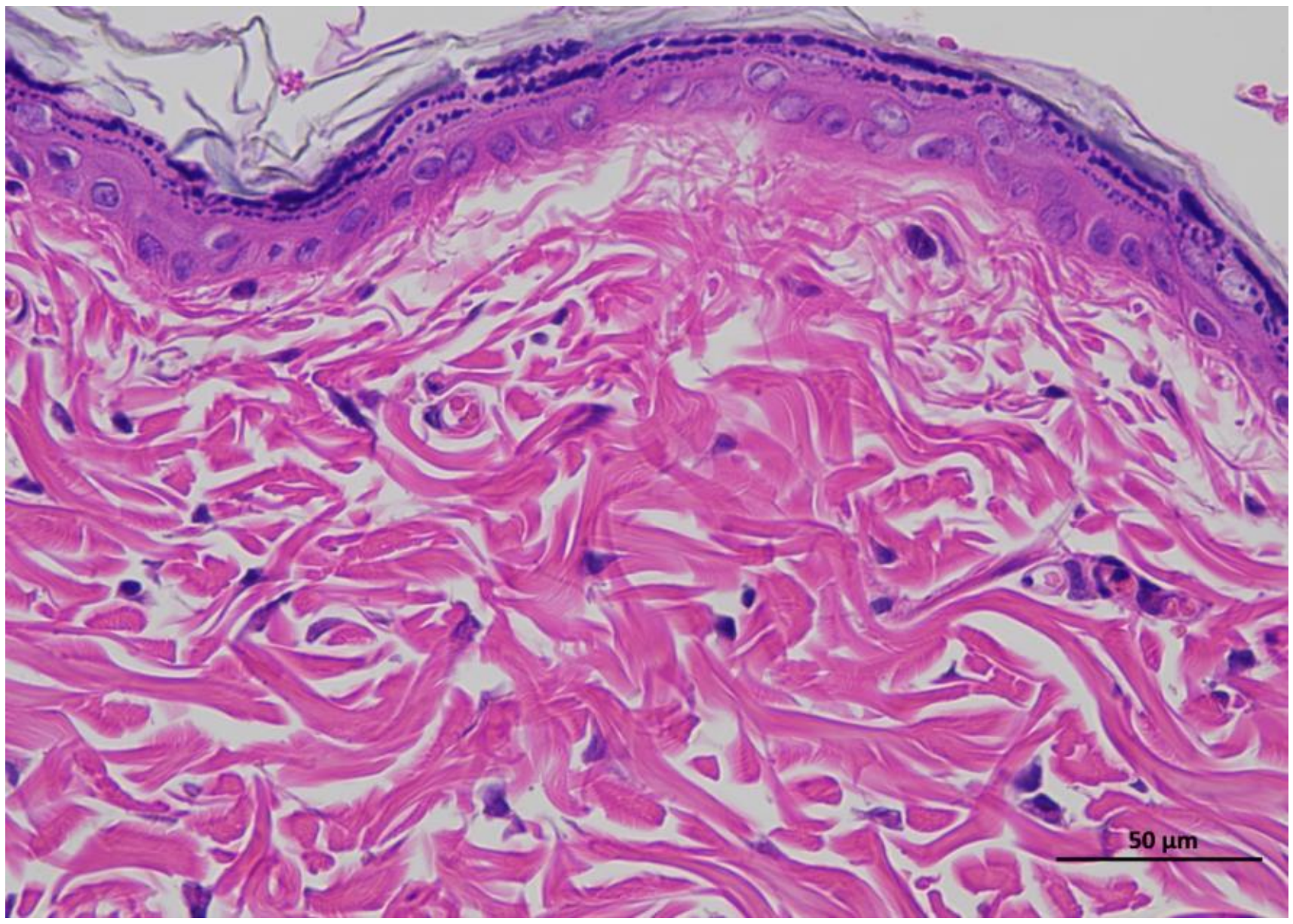

4 RNA-BEV-H 0day 400-1

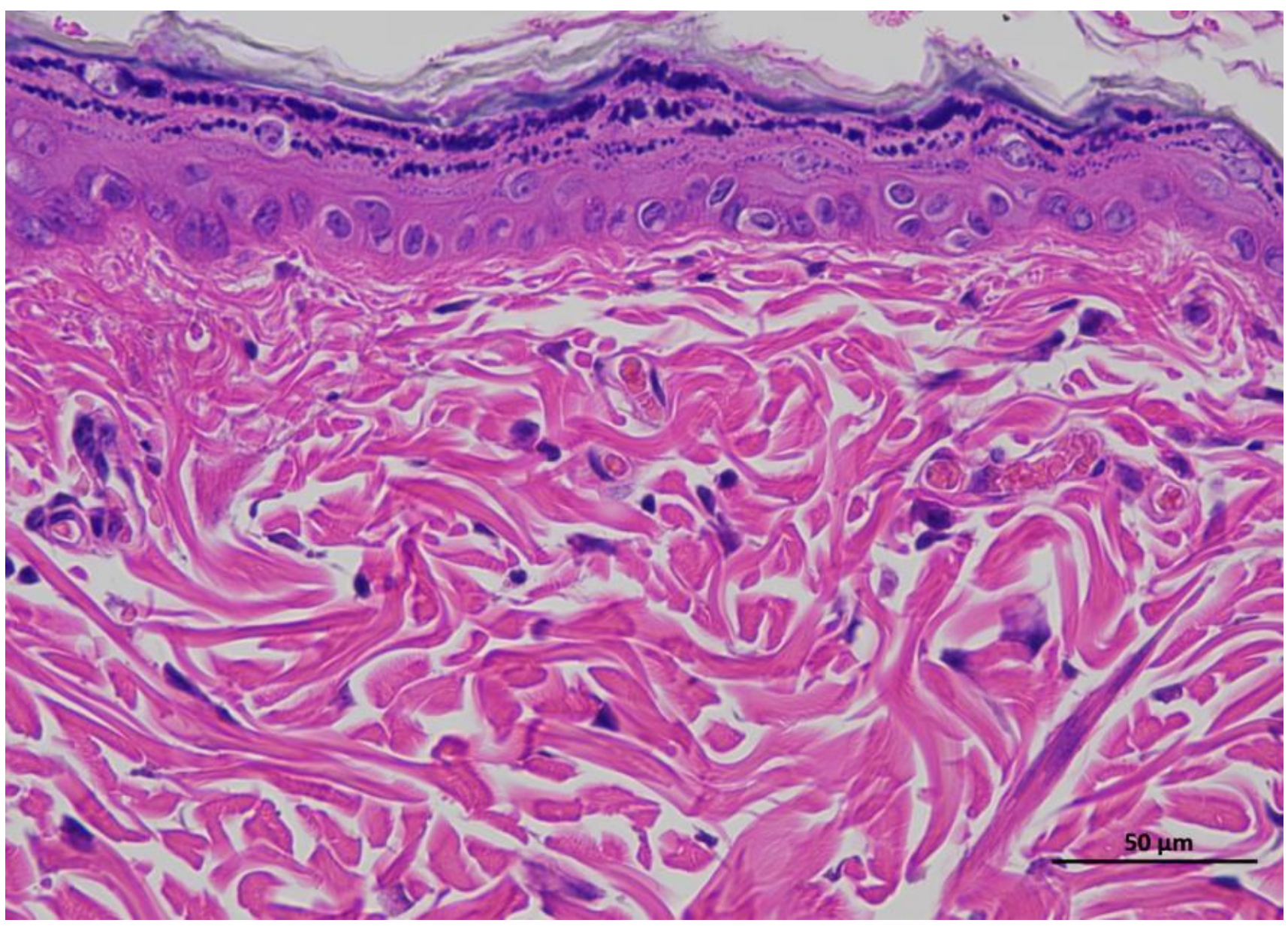

4 RNA-BEV-H 0day 400-2

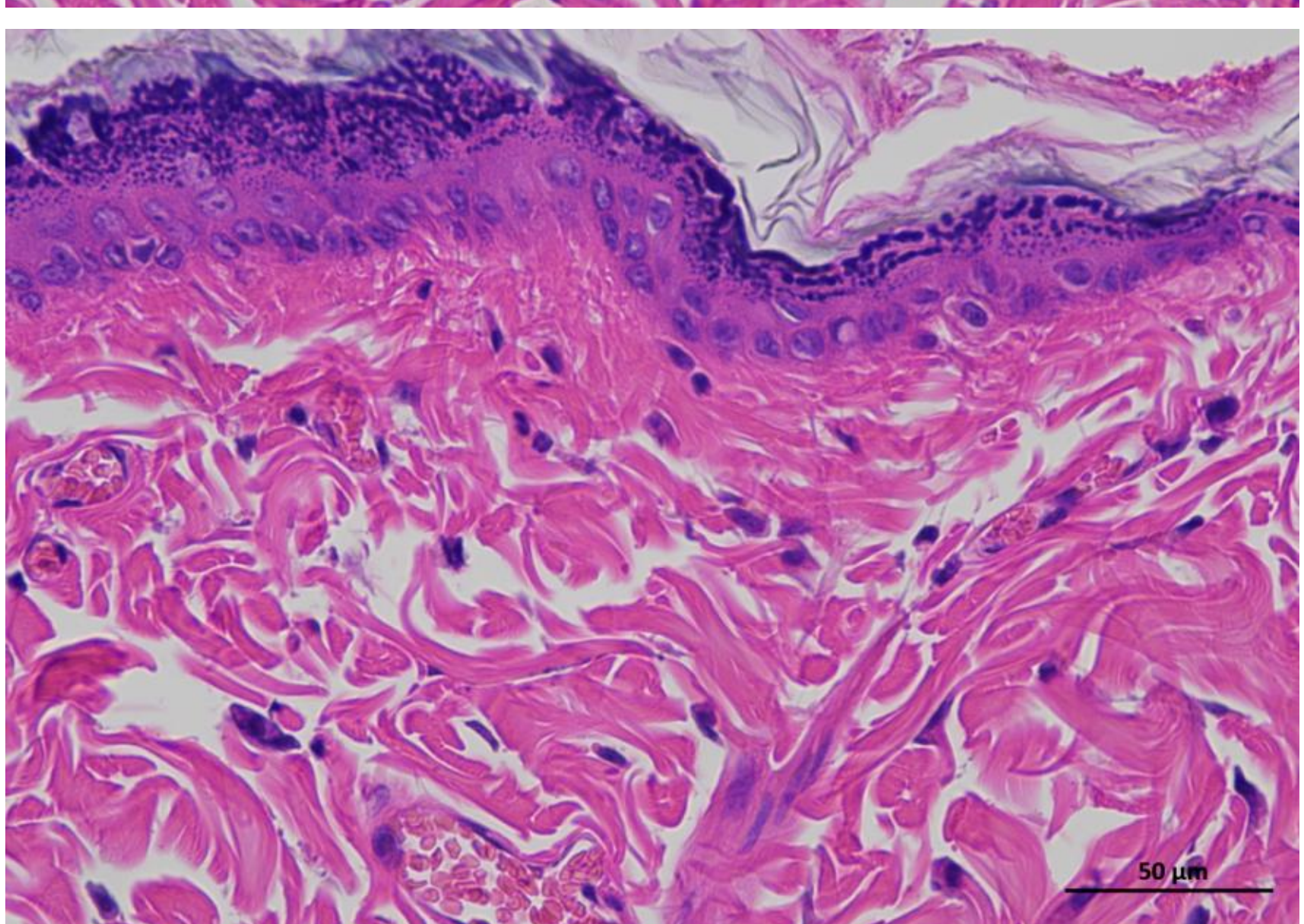

4 RNA-BEV-H 0day 400-3

Animal 5

Supplementary Figure 2f| Representative H&E staining of skin tissue of mRNA RNA-BEV-H animals / Day 0.

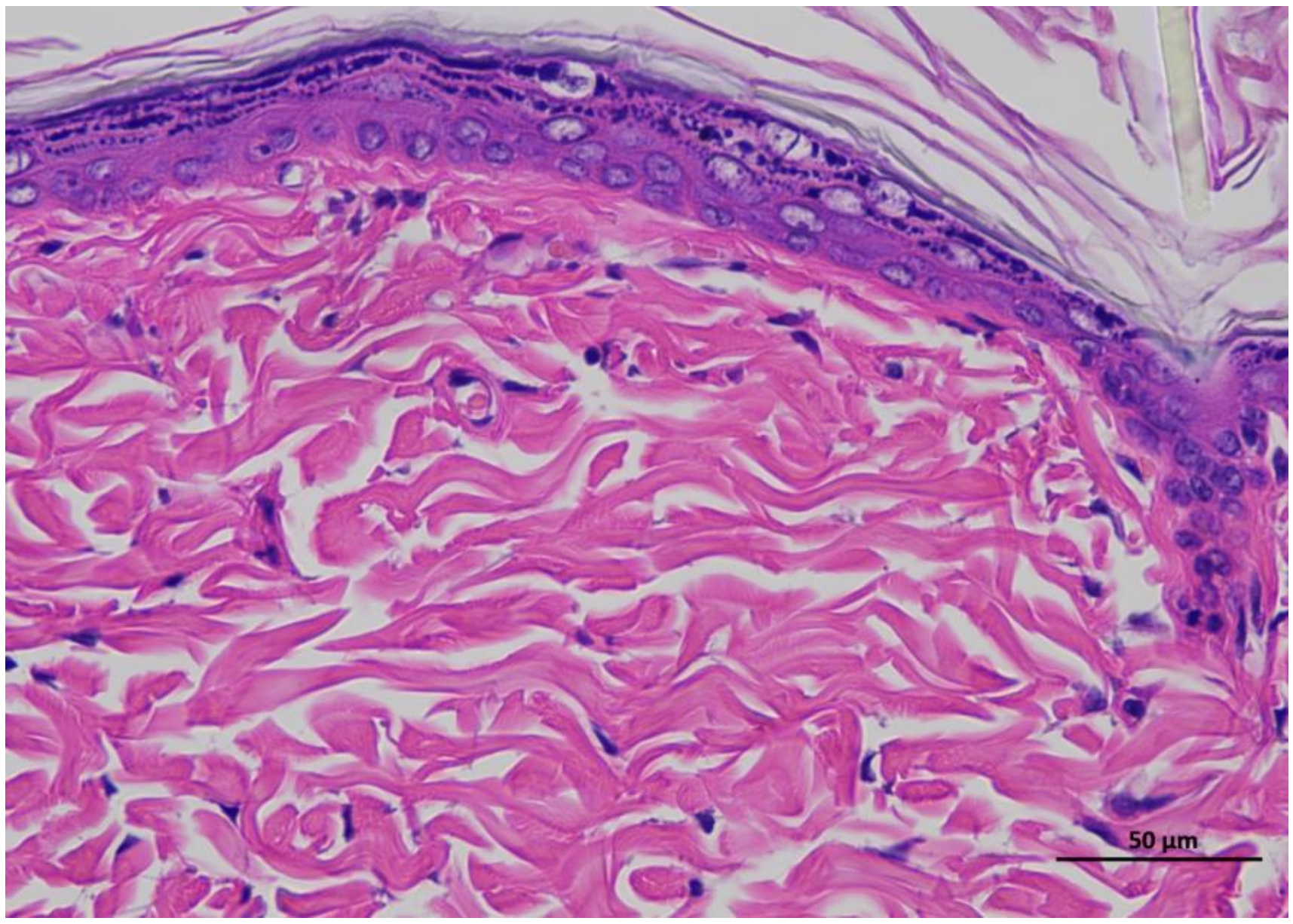

5 RNA-BEV-H 0day 400-1

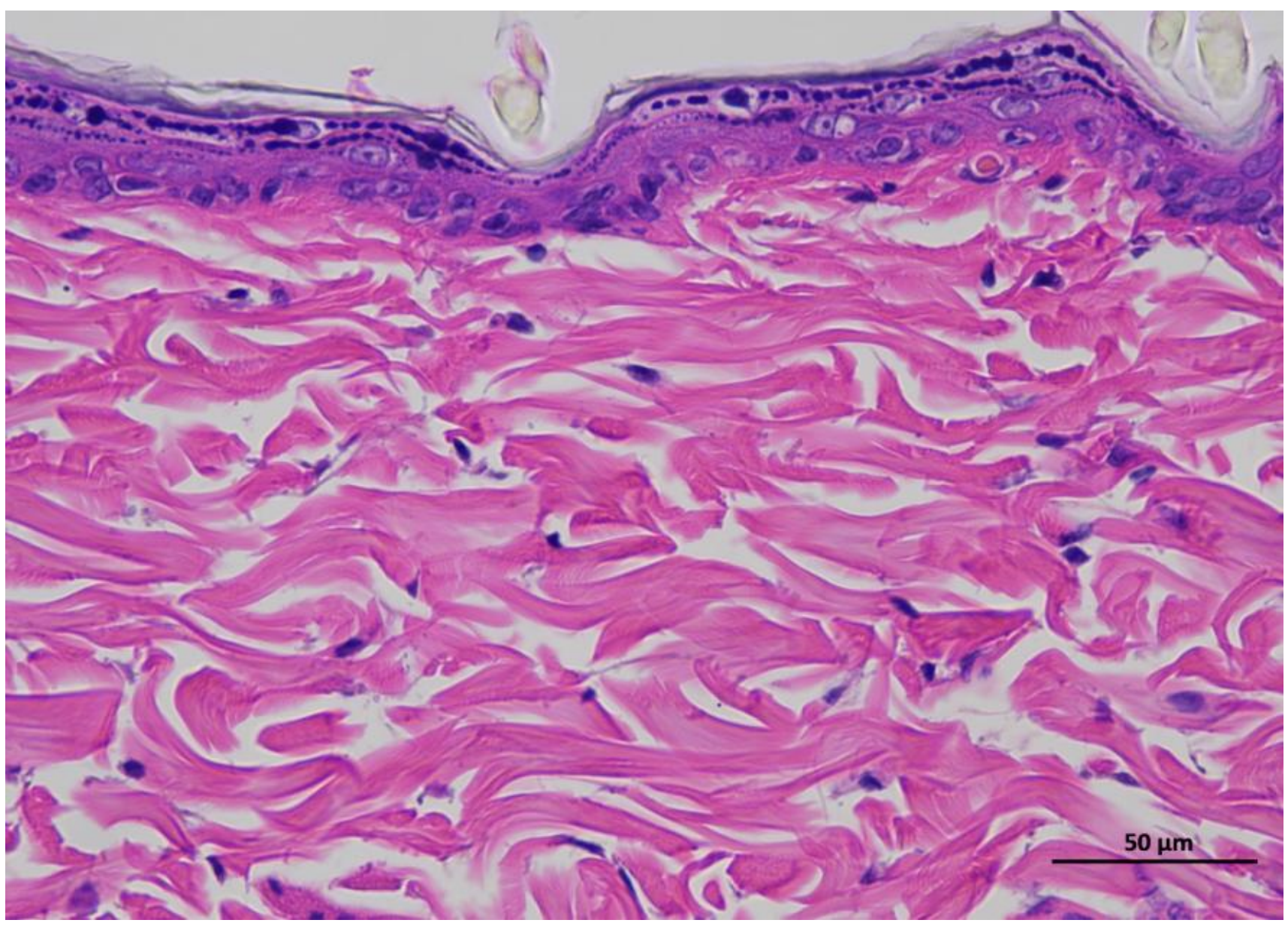

5 RNA-BEV-H 0day 400-2

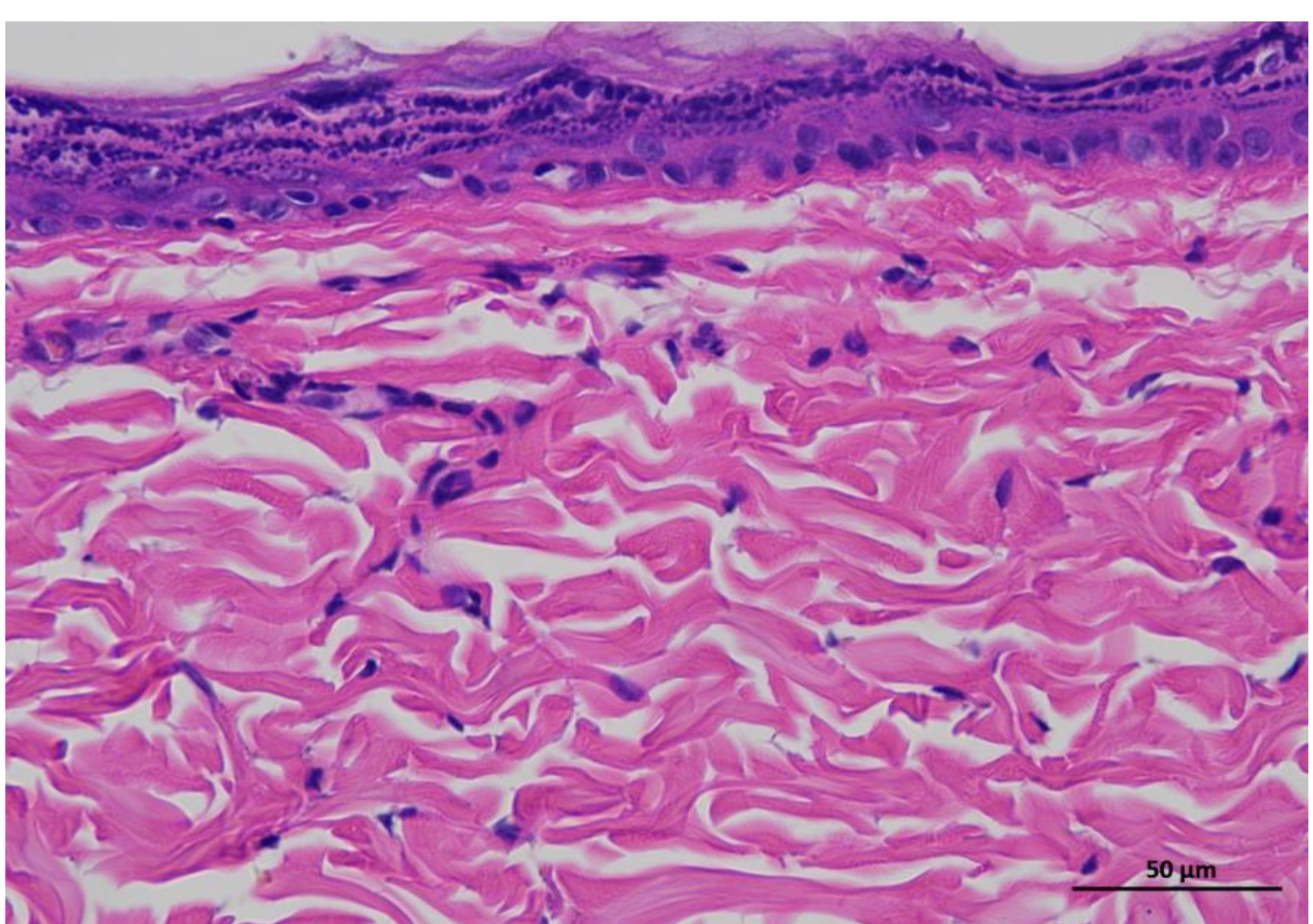

5 RNA-BEV-H 0day 400-3

Supplementary Figure 2f| Representative H&E staining of skin tissue of mRNA RNA-BEV-H animals / Day 0.

Animal 6

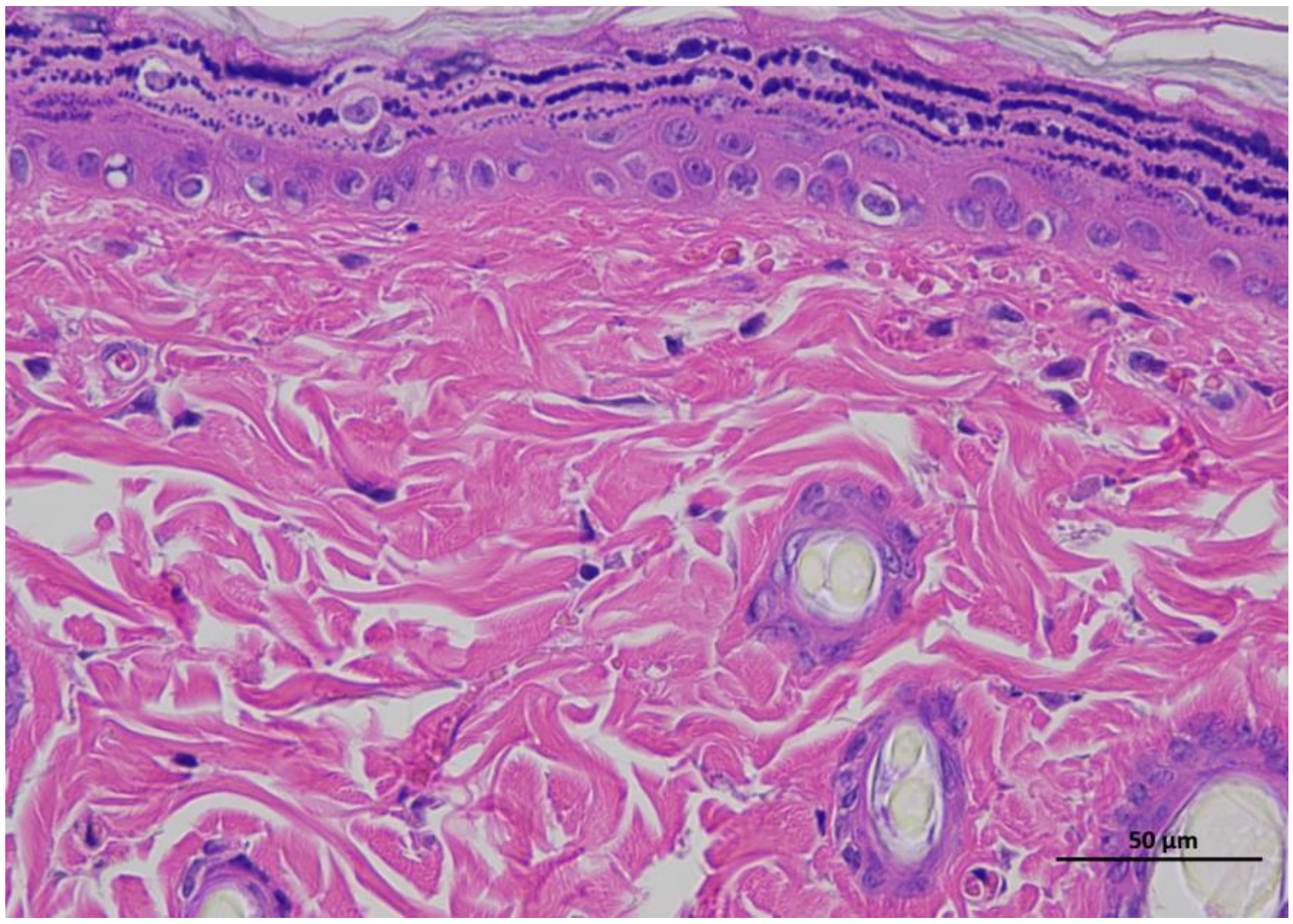

6 RNA-BEV-H 0day 400-1

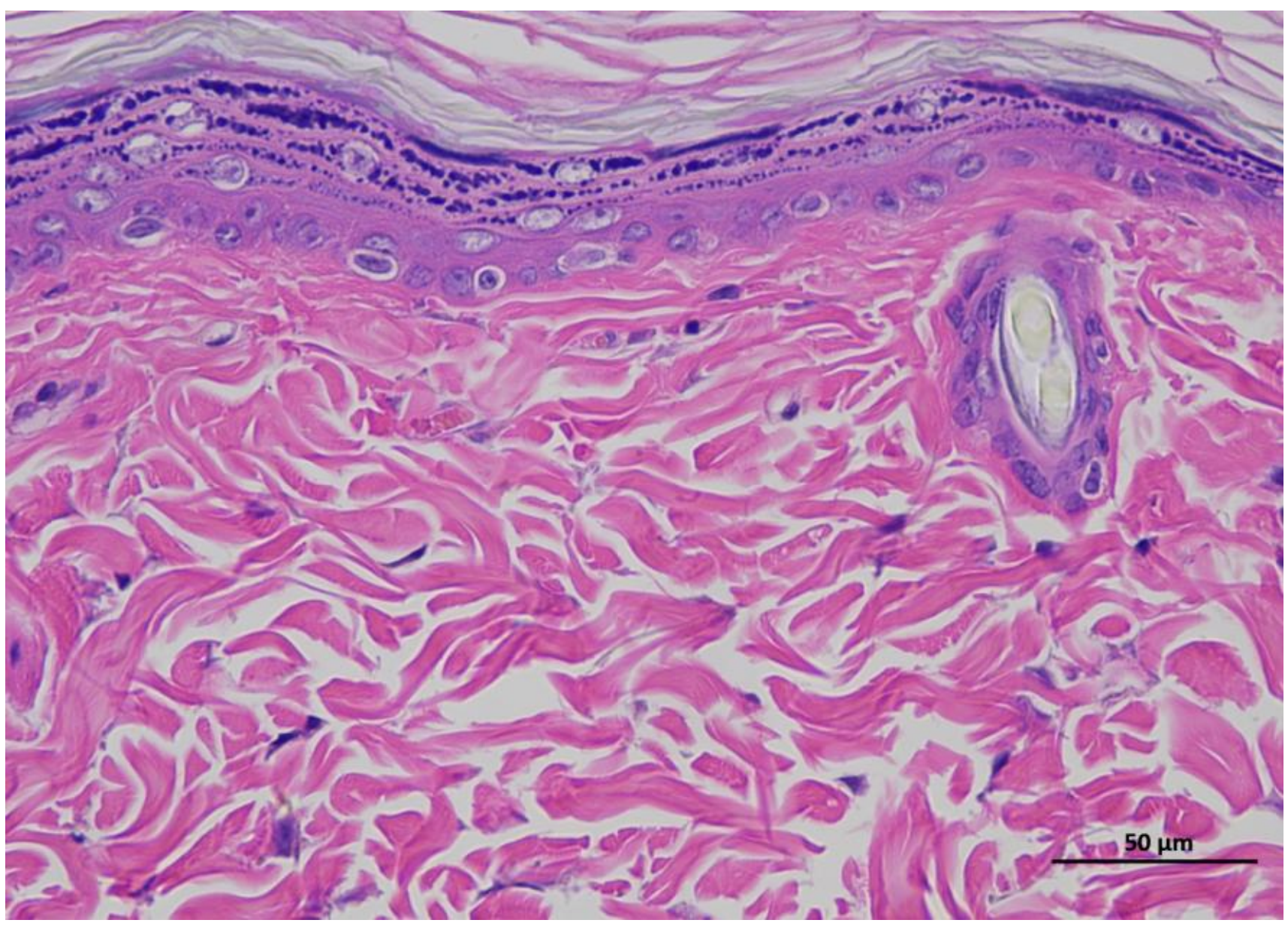

6 RNA-BEV-H 0day 400-2

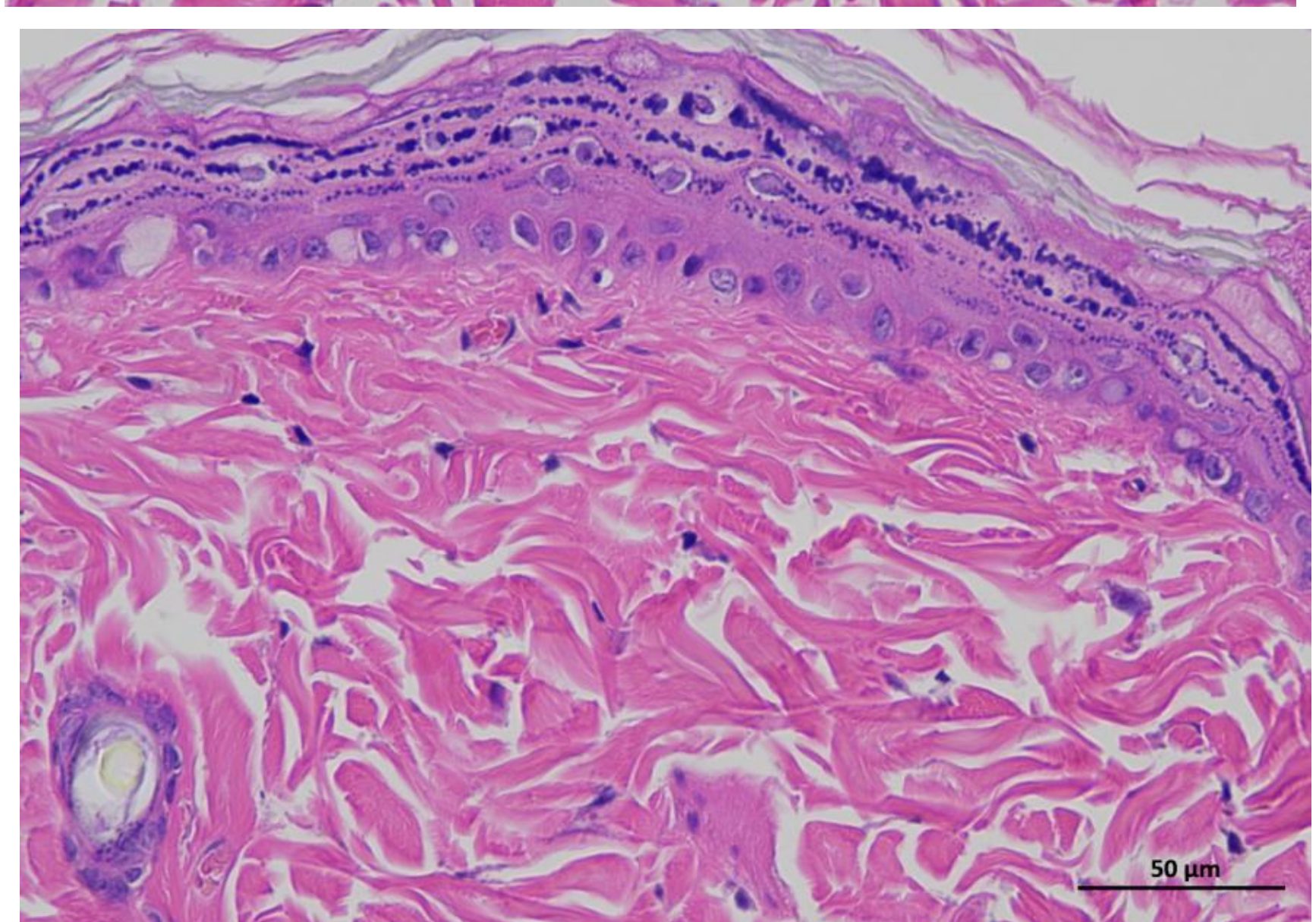

6 RNA-BEV-H 0day 400-3

Supplementary Figure 3f | Representative H&E staining of skin tissue of RNA-BEV-H animals / Day 14.
Representative hematoxylin and eosin (H&E)–stained section of skin tissue collected from experimental rat. The image shows the typical architecture of the skin, including the epidermal layer with stratified keratinized epithelium and the underlying dermis composed of dense collagen fibers and connective tissue structures.
(n = 6 per group).

Animal 1

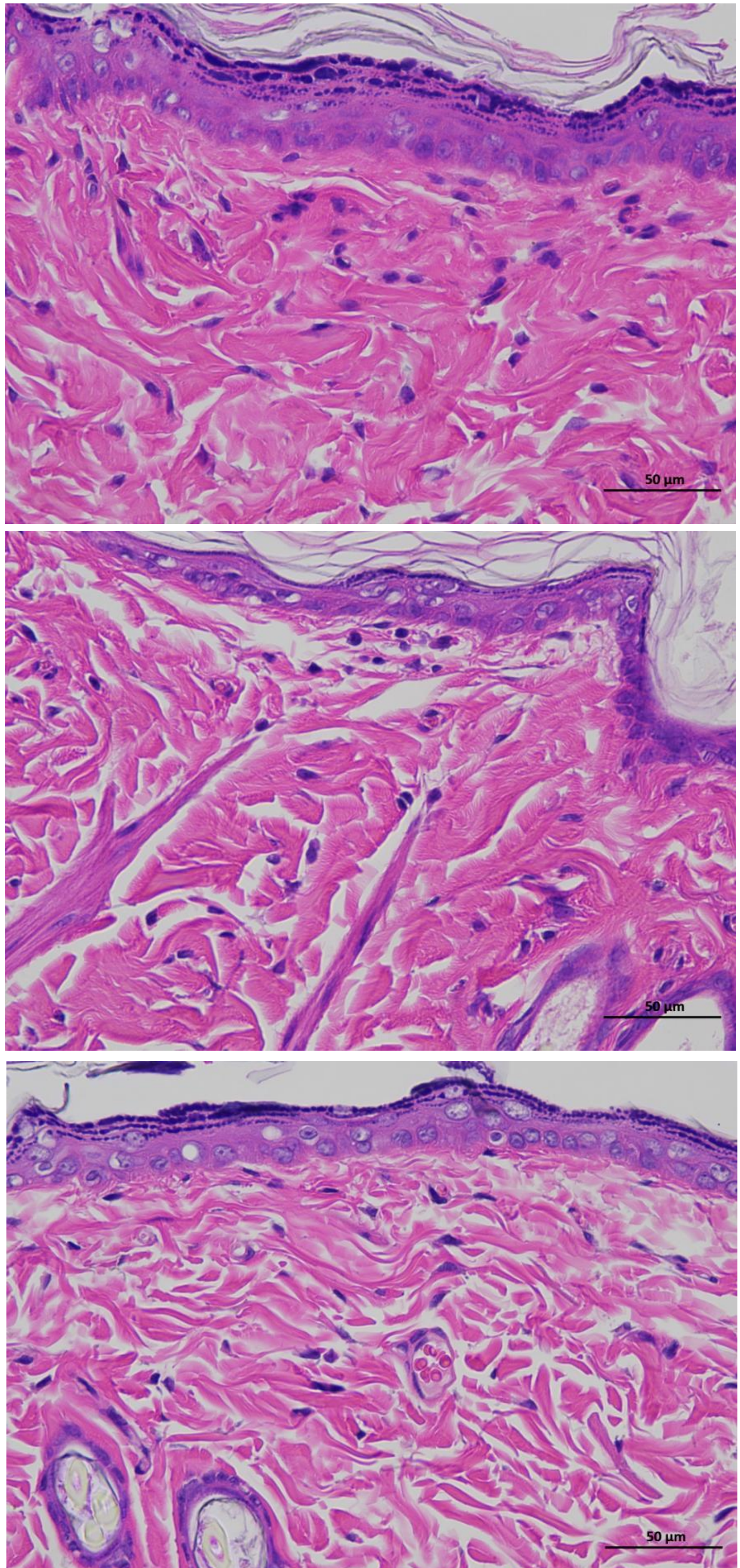

1 RNA-BEV-H 14day 400-1

1 RNA-BEV-H 14day 400-2

1 RNA-BEV-H 14day 400-3

Animal 1

Supplementary Figure 3f | Representative H&E staining of skin tissue of RNA-BEV-H animals / Day 14.

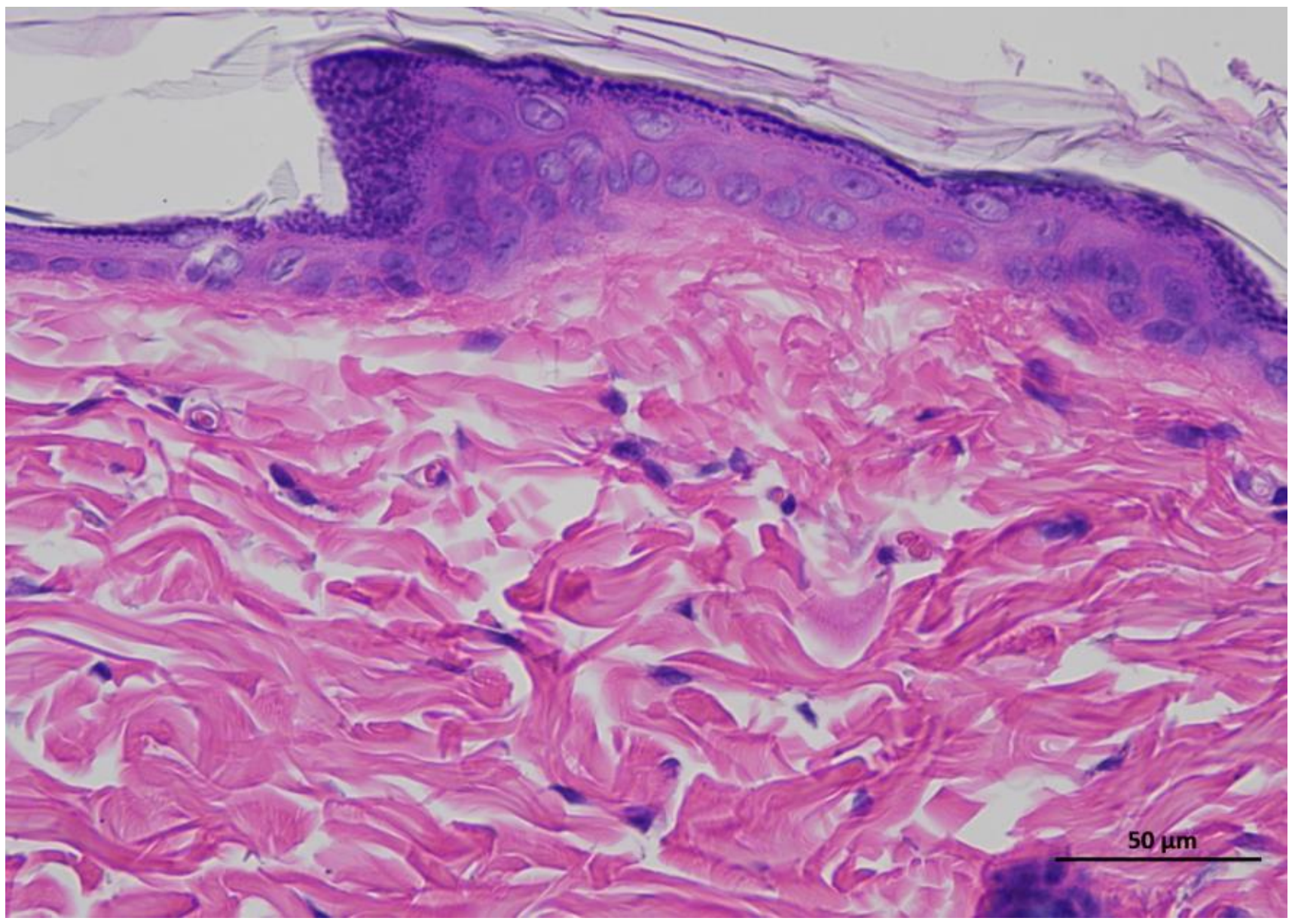

1 RNA-BEV-H 14day 400-4

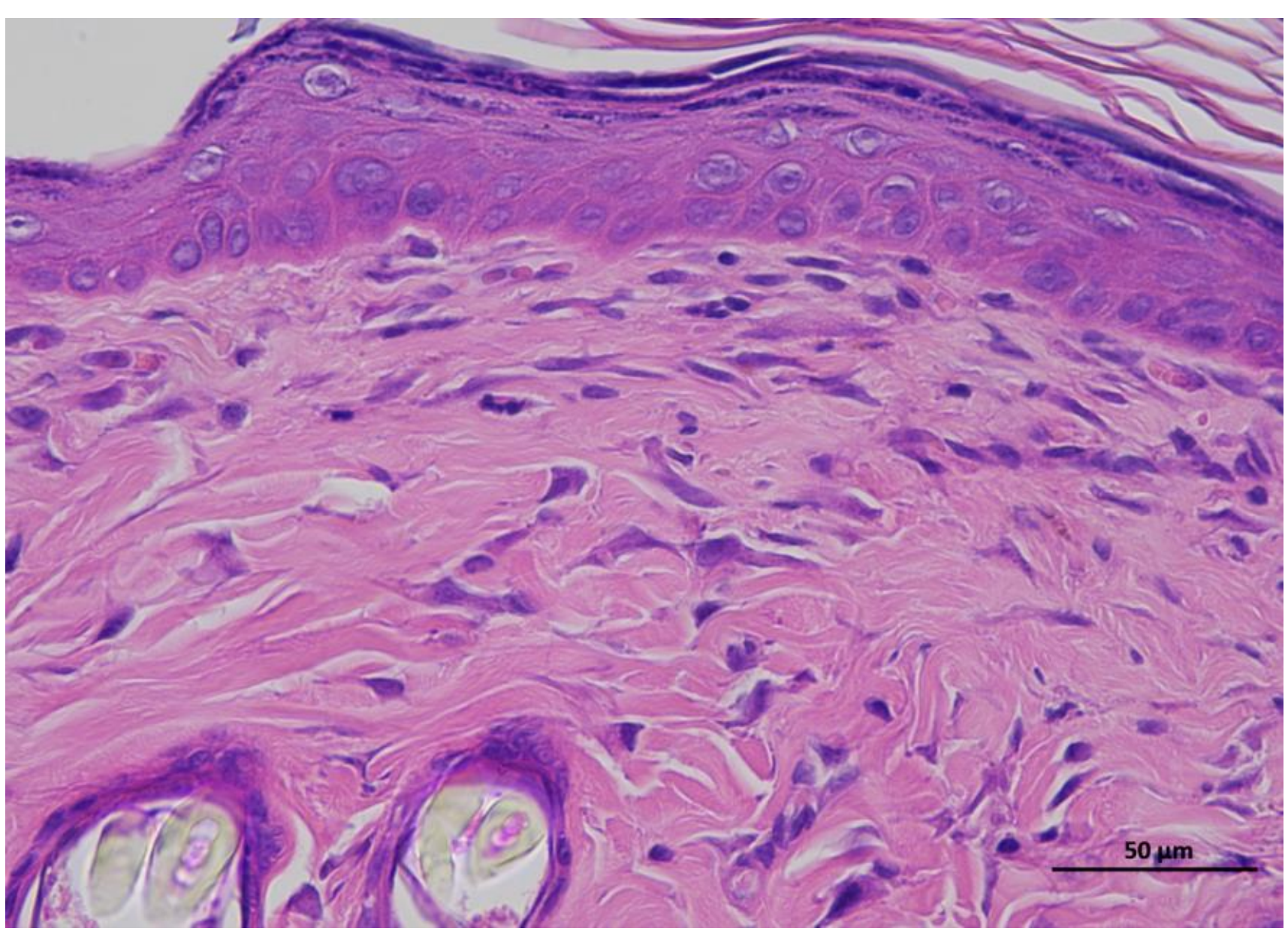

1 RNA-BEV-H 14day 400-5

Supplementary Figure 3f | Representative H&E staining of skin tissue of RNA-BEV-H animals / Day 14.

Animal 2

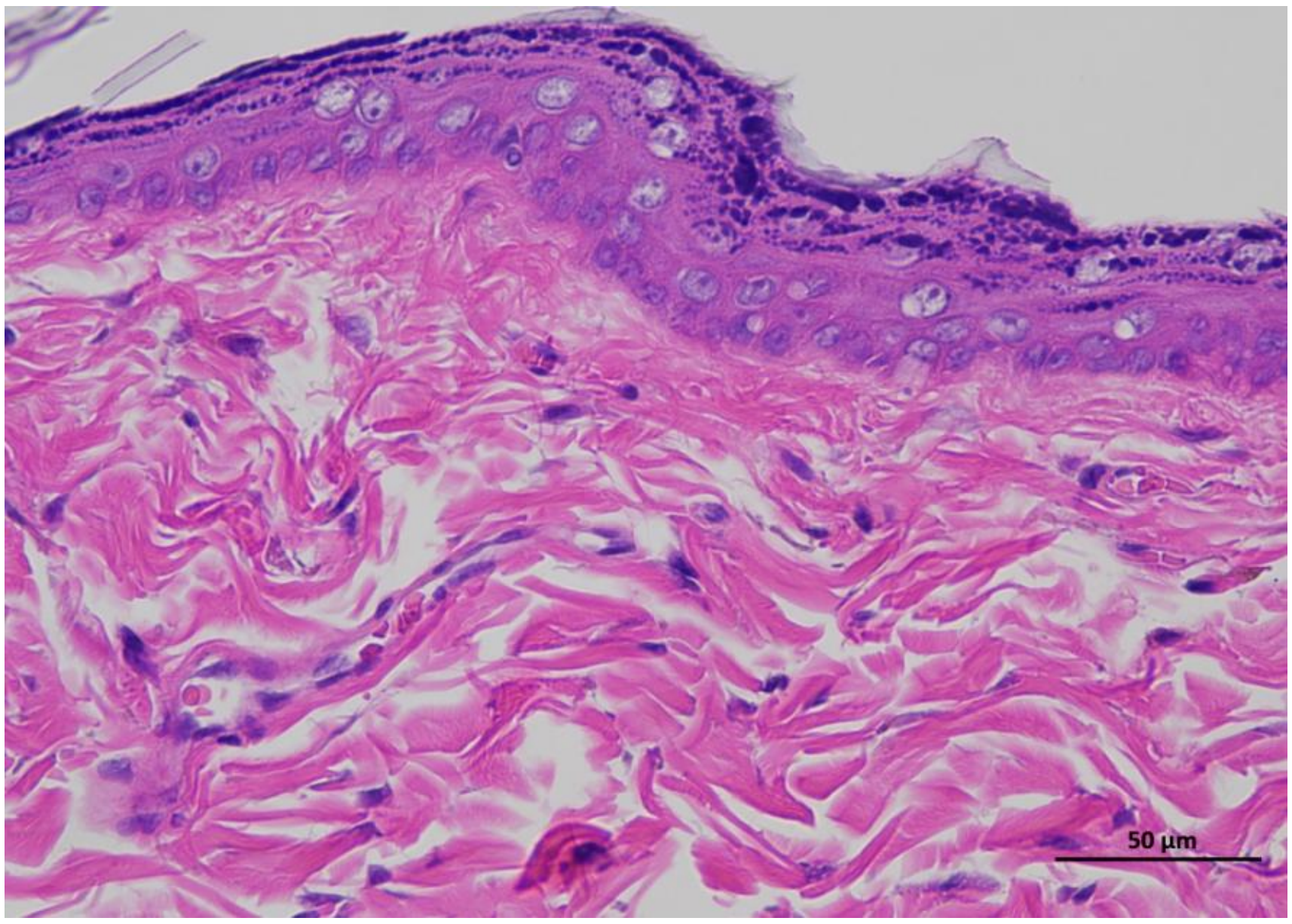

2 RNA-BEV-H 14day 400-1

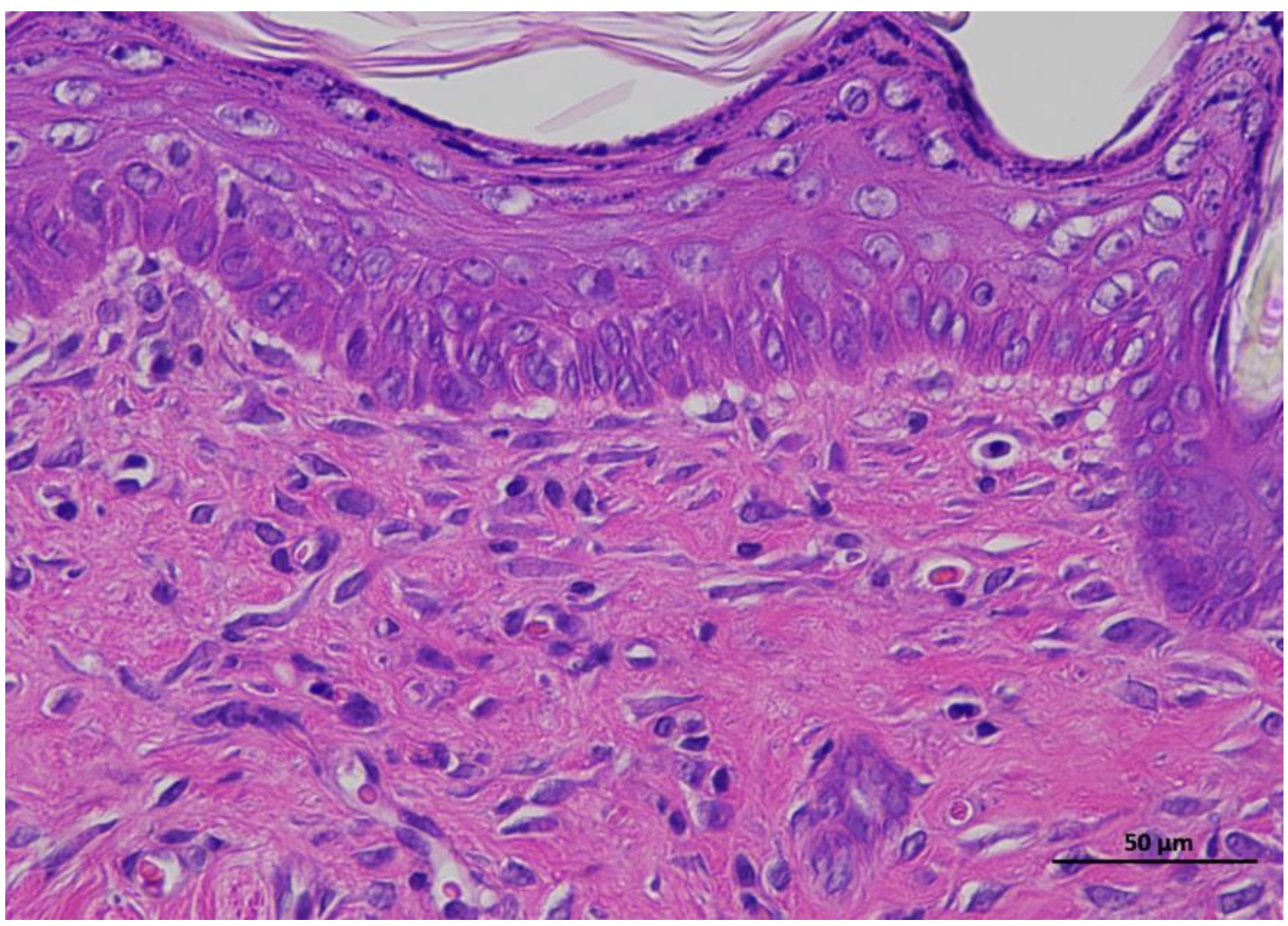

2 RNA-BEV-H 14day 400-2

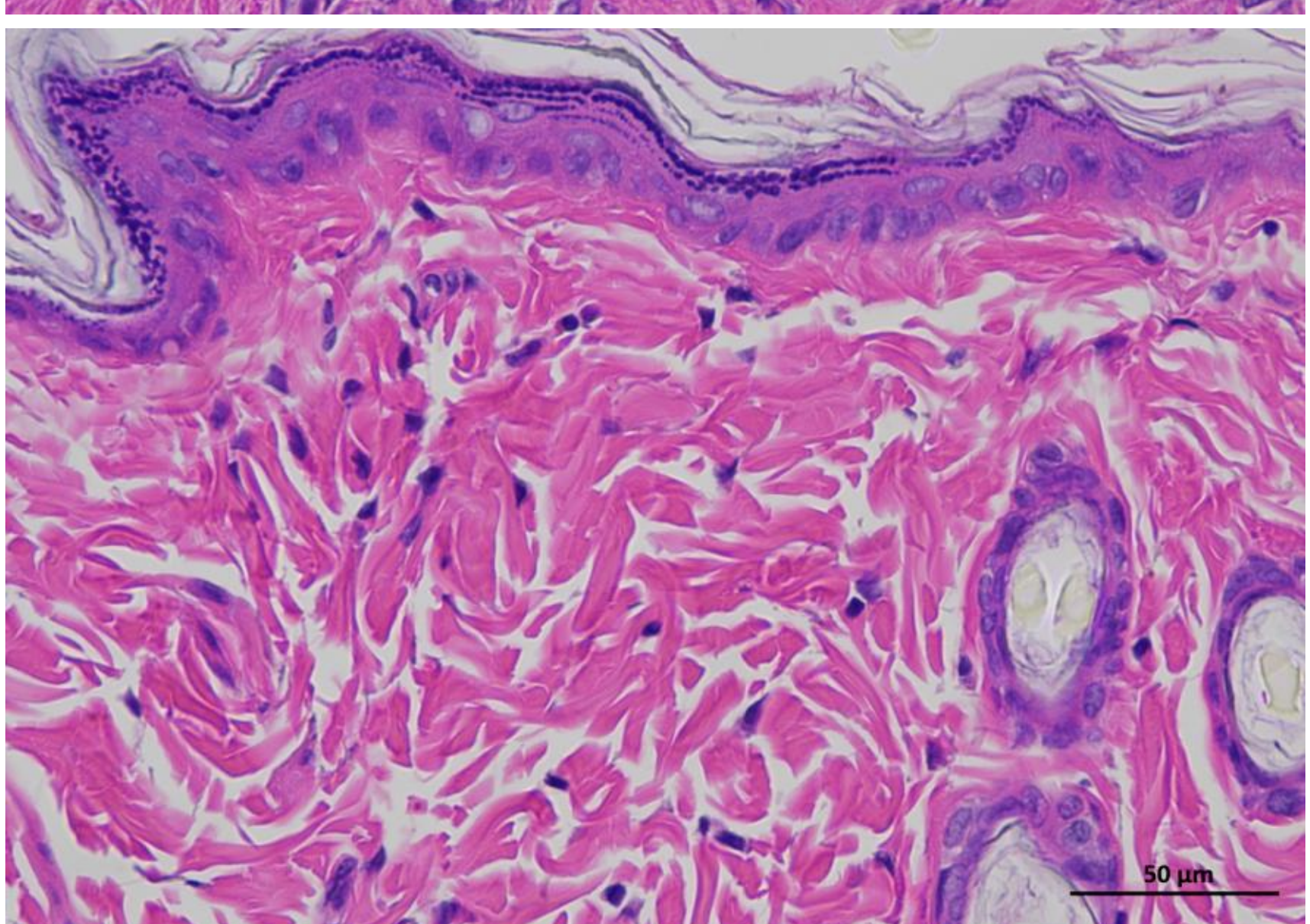

2 RNA-BEV-H 14day 400-3

Animal 2

Supplementary Figure 3f | Representative H&E staining of skin tissue of RNA-BEV-H animals / Day 14.

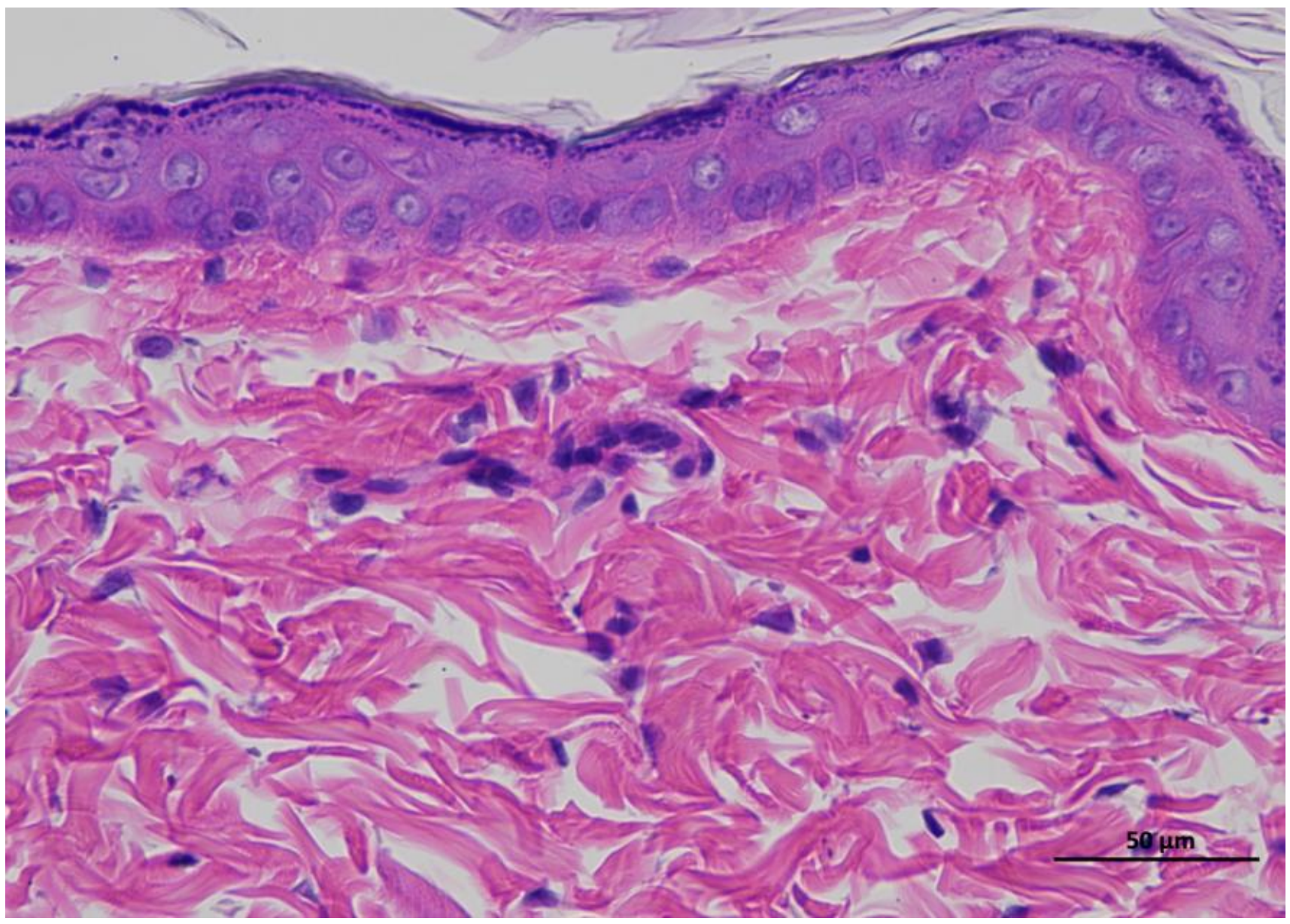

2 RNA-BEV-H 14day 400-4

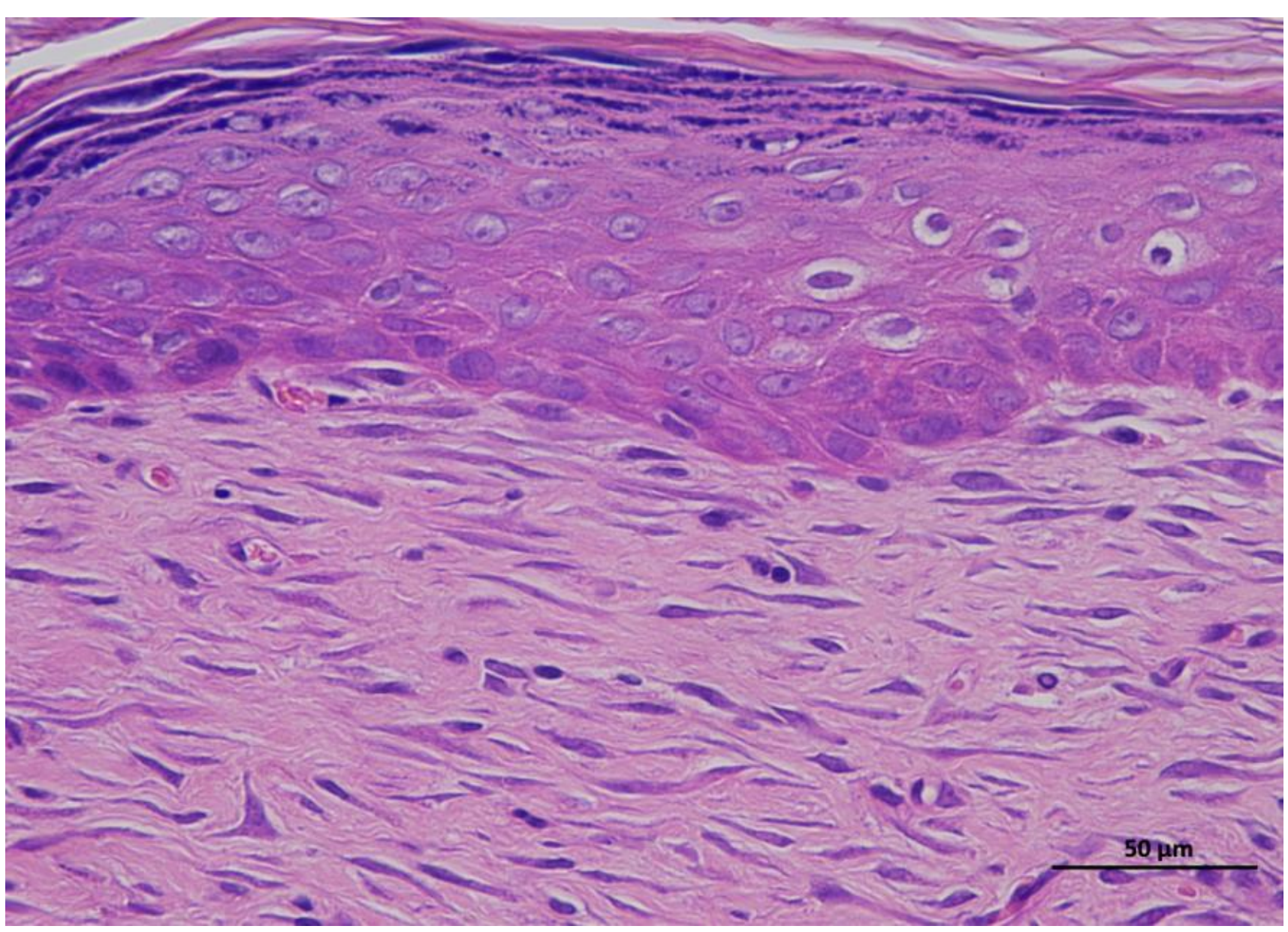

2 RNA-BEV-H 14day 400-5

Supplementary Figure 3f | Representative H&E staining of skin tissue of RNA-BEV-H animals / Day 14.

Animal 3

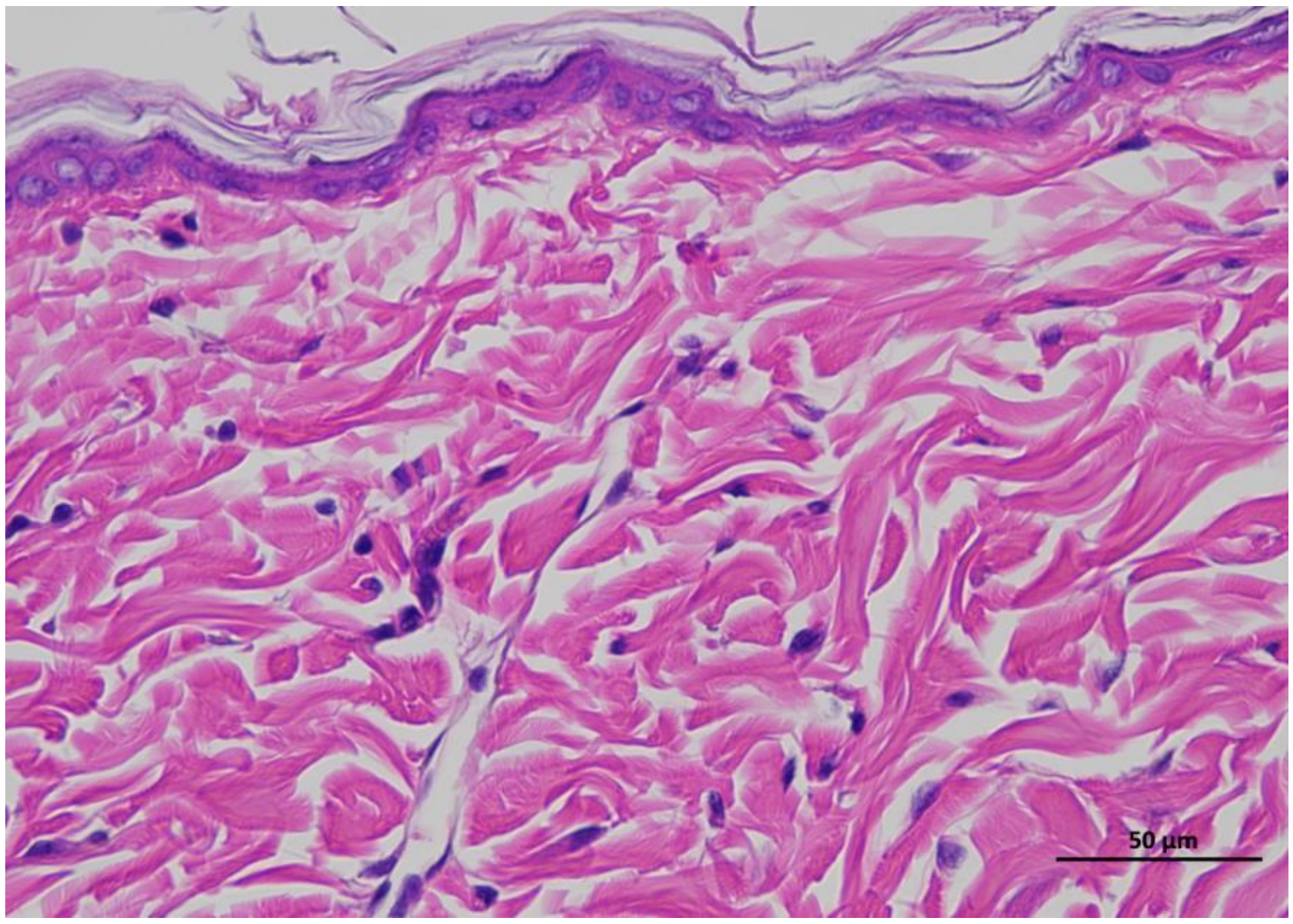

3 RNA-BEV-H 14day 400-1

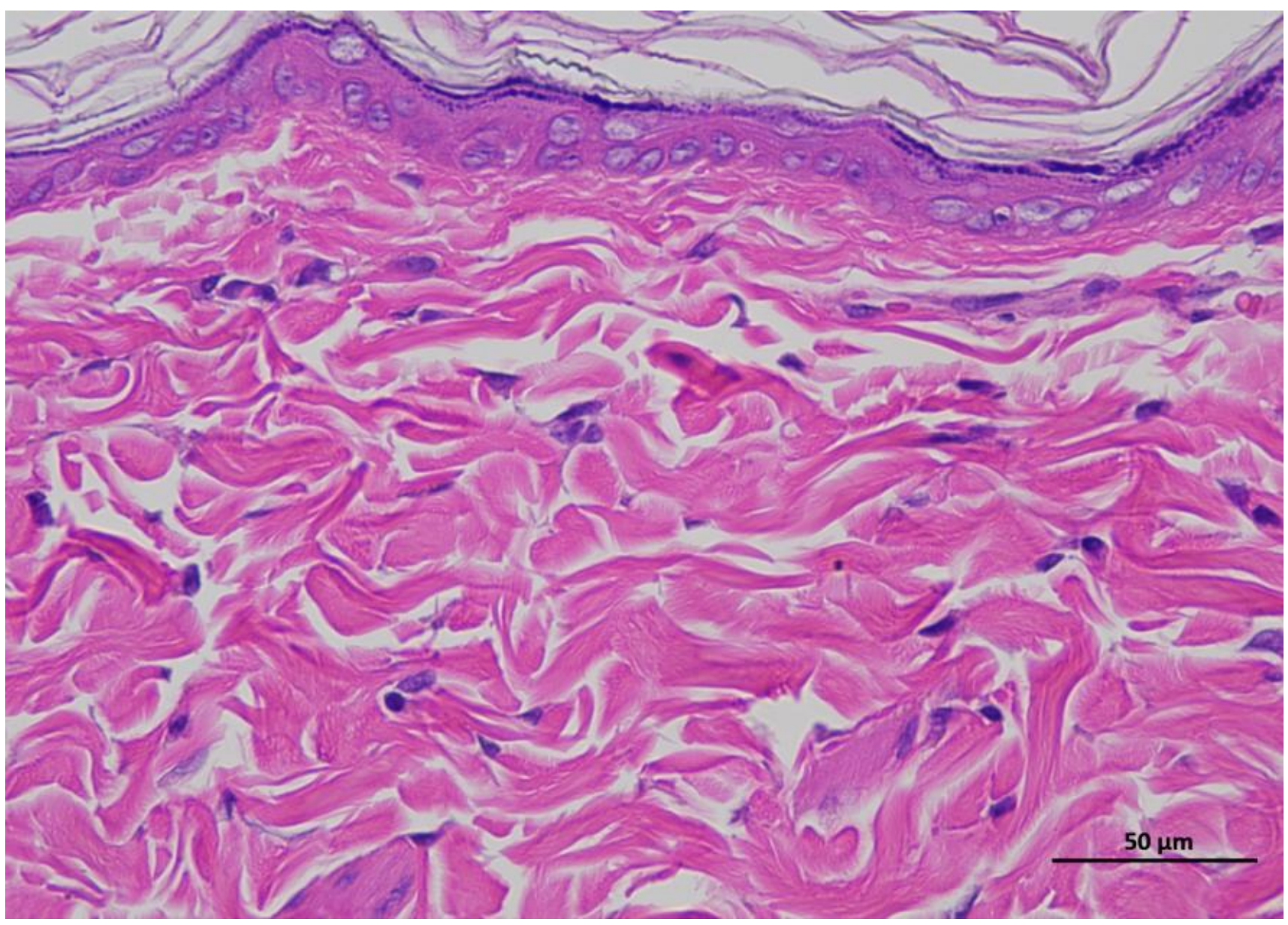

3 RNA-BEV-H 14day 400-2

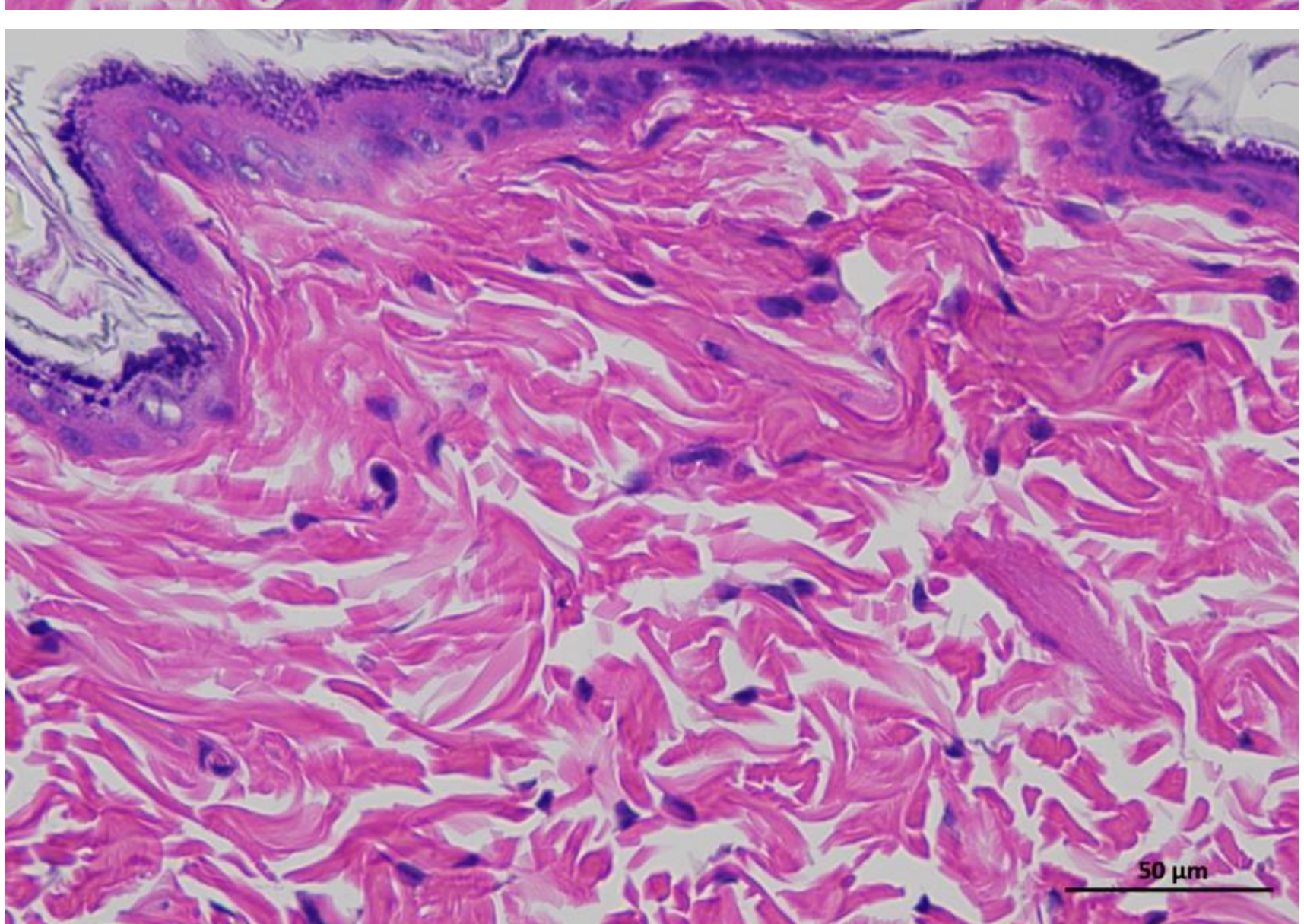

3 RNA-BEV-H 14day 400-3

Animal 3

Supplementary Figure 3f | Representative H&E staining of skin tissue of RNA-BEV-H animals / Day 14.

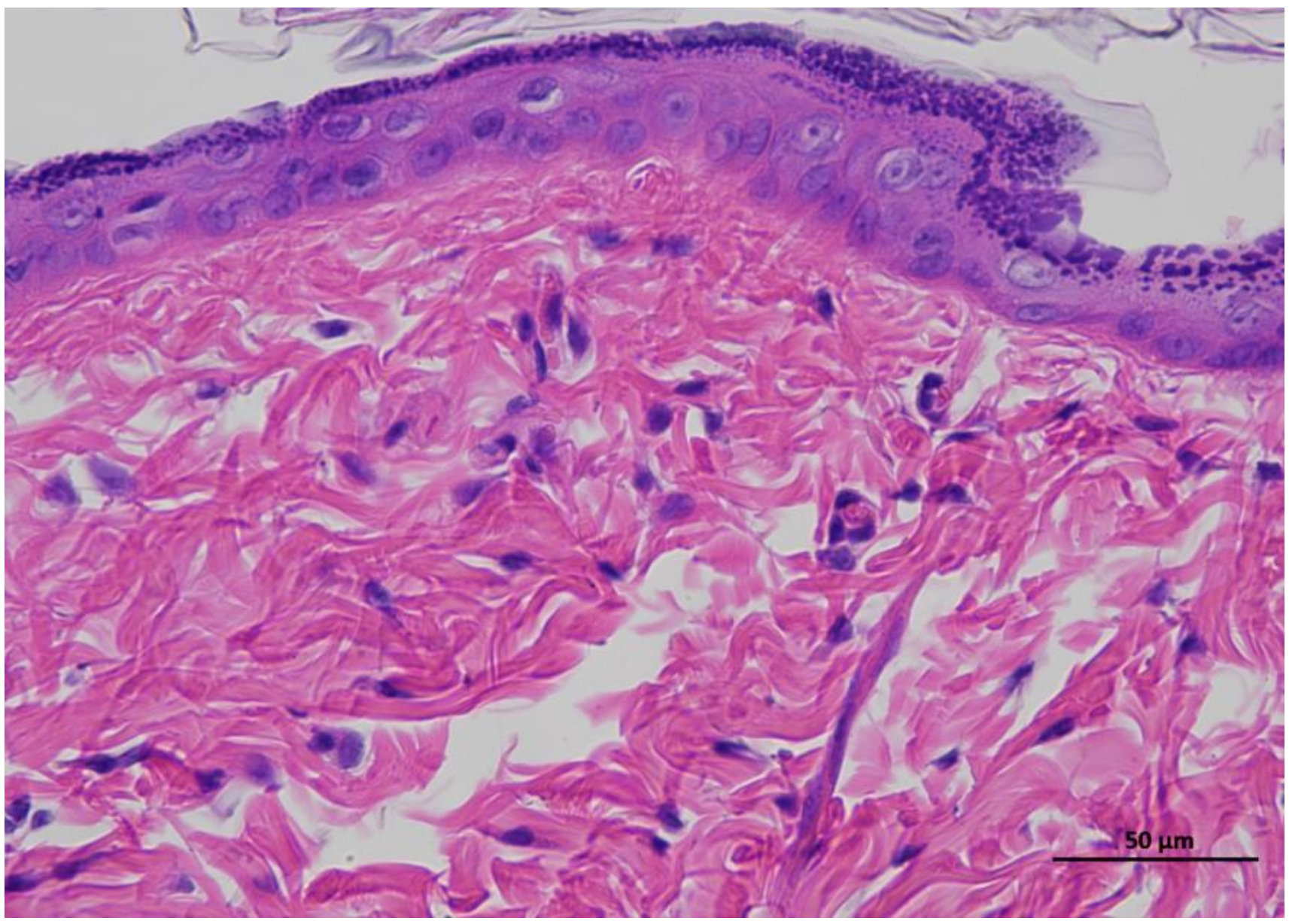

3 RNA-BEV-H 14day 400-4

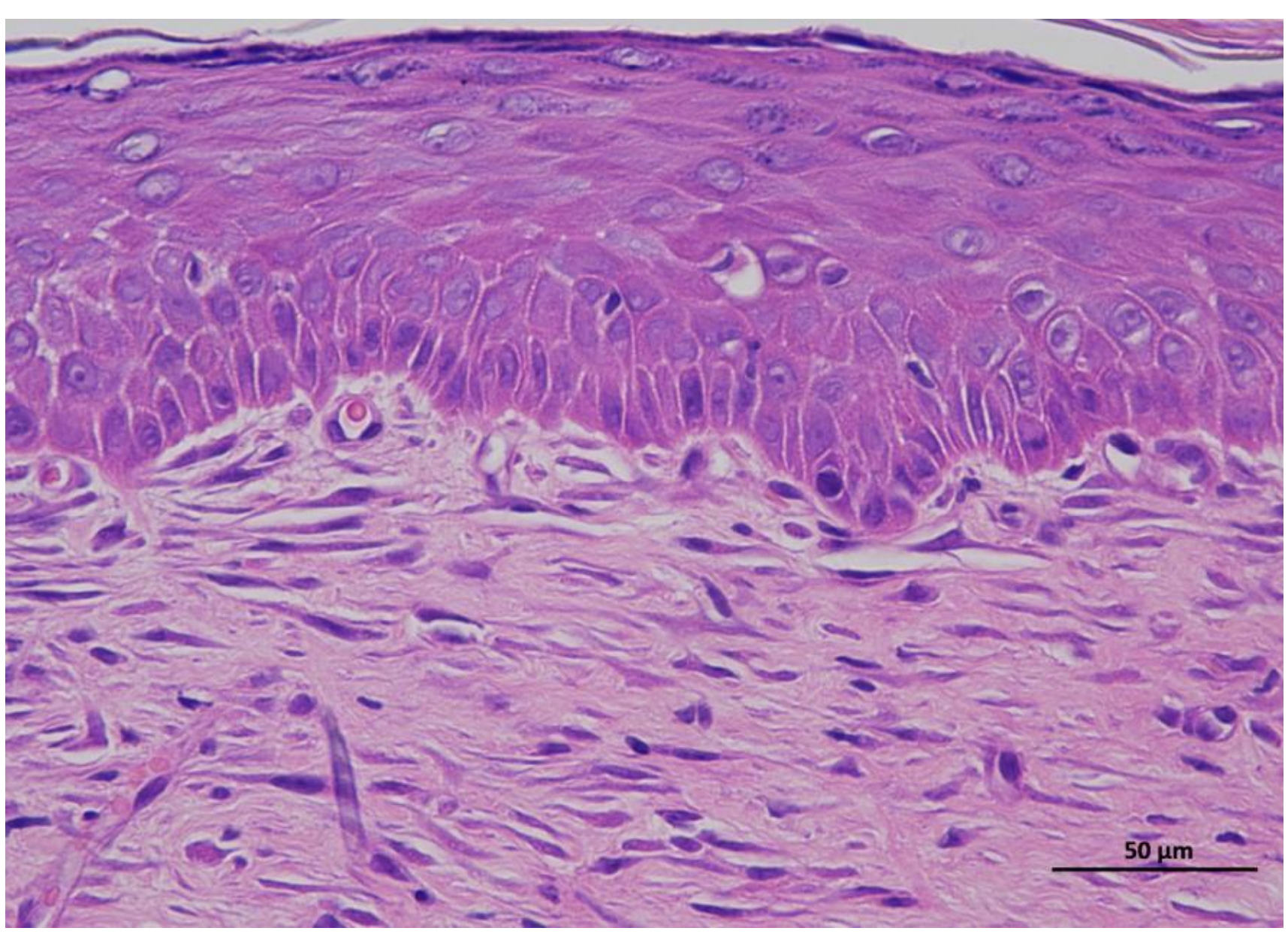

3 RNA-BEV-H 14day 400-5
