# Supplementary material for: Biologically engineered vectors enable topical mRNA delivery for skin regeneration
Source: Commun Biol. 2026 Jul 8;9:1436. doi: 10.1038/s42003-026-10651-9 (PMC13724683; doi:10.1038/s42003-026-10651-9)
Supplement: Supplementary file 3 [file 42003_2026_10651_MOESM3_ESM.pdf]

Description of Additional Supplementary files

File name: Supplementary Data 1

Description: Source data underlying the graphs in the main figures.
