# Supplementary material for: Biologically engineered vectors enable topical mRNA delivery for skin regeneration
Source: Commun Biol. 2026 Jul 8;9:1436. doi: 10.1038/s42003-026-10651-9 (PMC13724683; doi:10.1038/s42003-026-10651-9)
Supplement: Supplementary file 4 [file 42003_2026_10651_MOESM5_ESM.pdf]

nature portfolio

Corresponding author(s): Nicolas Grandcamp

Last updated by author(s): 2026/06/24

# Reporting Summary

Nature Portfolio wishes to improve the reproducibility of the work that we publish. This form provides structure for consistency and transparency in reporting. For further information on Nature Portfolio policies, see our Editorial Policies and the Editorial Policy Checklist.

## Statistics

For all statistical analyses, confirm that the following items are present in the figure legend, table legend, main text, or Methods section.

| n/a | Confirmed | |
|---|---|---|
| ☐ | ☒ | The exact sample size (*n*) for each experimental group/condition, given as a discrete number and unit of measurement |
| ☐ | ☒ | A statement on whether measurements were taken from distinct samples or whether the same sample was measured repeatedly |
| ☐ | ☒ | The statistical test(s) used AND whether they are one- or two-sided<br>*Only common tests should be described solely by name; describe more complex techniques in the Methods section.* |
| ☒ | ☐ | A description of all covariates tested |
| ☐ | ☒ | A description of any assumptions or corrections, such as tests of normality and adjustment for multiple comparisons |
| ☒ | ☐ | A full description of the statistical parameters including central tendency (e.g. means) or other basic estimates (e.g. regression coefficient) AND variation (e.g. standard deviation) or associated estimates of uncertainty (e.g. confidence intervals) |
| ☒ | ☐ | For null hypothesis testing, the test statistic (e.g. *F*, *t*, *r*) with confidence intervals, effect sizes, degrees of freedom and *P* value noted<br>*Give P values as exact values whenever suitable.* |
| ☒ | ☐ | For Bayesian analysis, information on the choice of priors and Markov chain Monte Carlo settings |
| ☒ | ☐ | For hierarchical and complex designs, identification of the appropriate level for tests and full reporting of outcomes |
| ☒ | ☐ | Estimates of effect sizes (e.g. Cohen's *d*, Pearson's *r*), indicating how they were calculated |

*Our web collection on statistics for biologists contains articles on many of the points above.*

## Software and code

Policy information about availability of computer code

| | |
|---|---|
| Data collection | Data were collected using standard laboratory and imaging workflows. Vector quantification was performed by qRT–PCR on a QuantStudio 7 Pro instrument. Image acquisition for reconstructed skin sections was performed on a Nikon microscope with Nikon camera and NIS-Elements software. Keratinocyte migration was monitored using an INCell Analyzer 2200 imaging system. Wound photographs were acquired at defined time points under standardized lighting and distance conditions, and blood glucose was measured with a commercial glucomete |
| Data analysis | Data analysis was performed using GraphPad Prism (v9.0) for statistical analyses and ImageJ for image-based quantification, including wound area, fluorescence intensity and histomorphometric measurements. Nikon NIS-Elements was used for image processing. No custom code was used. |

For manuscripts utilizing custom algorithms or software that are central to the research but not yet described in published literature, software must be made available to editors and reviewers. We strongly encourage code deposition in a community repository (e.g. GitHub). See the Nature Portfolio guidelines for submitting code & software for further information.

# Data

Policy information about availability of data

All manuscripts must include a data availability statement. This statement should provide the following information, where applicable:

- Accession codes, unique identifiers, or web links for publicly available datasets
- A description of any restrictions on data availability
- For clinical datasets or third party data, please ensure that the statement adheres to our policy

All data supporting the findings of this study are available within the Article and its Supplementary Information. Source data underlying the graphs in the main figures are provided in Supplementary Data 1. Additional raw data supporting the findings of this study are available from the corresponding author upon reasonable request.

# Research involving human participants, their data, or biological material

Policy information about studies with human participants or human data. See also policy information about sex, gender (identity/presentation), and sexual orientation and race, ethnicity and racism.

| | |
|---|---|
| Reporting on sex and gender | This study did not involve direct recruitment of human participants by the authors. Commercially obtained human primary keratinocytes from ATCC and a commercially produced reconstructed human skin model from QIMA Bioalternatives were used. |
| Reporting on race, ethnicity, or other socially relevant groupings | N/A |
| Population characteristics | N/A |
| Recruitment | N/A |
| Ethics oversight | Not applicable; no human participants were recruited by the authors. Commercially obtained human primary keratinocytes from ATCC and a commercially produced reconstructed human skin model from QIMA Bioalternatives were used. |

Note that full information on the approval of the study protocol must also be provided in the manuscript.

# Field-specific reporting

Please select the one below that is the best fit for your research. If you are not sure, read the appropriate sections before making your selection.

☒ Life sciences ☐ Behavioural & social sciences ☐ Ecological, evolutionary & environmental sciences

For a reference copy of the document with all sections, see nature.com/documents/nr-reporting-summary-flat.pdf

# Life sciences study design

All studies must disclose on these points even when the disclosure is negative.

| | |
|---|---|
| Sample size | In vitro experiments were generally performed in triplicate (n = 3), including keratinocyte migration assays and reconstructed skin experiments. For the in vivo wound-healing study, diabetic rats were randomly assigned to six experimental groups with n = 6 animals per group. No a priori power calculation is described in the current manuscript. |
| Data exclusions | No data were excluded from the analyses. |
| Replication | In vitro experiments were performed with the biological/technical replication described in the manuscript, including triplicate cultures where indicated. The animal study included six biological replicates per group. Histological scoring was averaged per animal. No data were omitted from the reported analyses. |
| Randomization | Diabetic rats were randomly assigned to six experimental groups. No specific randomization procedure is described for the in vitro experiments. |
| Blinding | Histological scoring was performed independently by two observers blinded to treatment allocation on coded images. Blinding is not explicitly described for the other experiments. |

# Reporting for specific materials, systems and methods

We require information from authors about some types of materials, experimental systems and methods used in many studies. Here, indicate whether each material, system or method listed is relevant to your study. If you are not sure if a list item applies to your research, read the appropriate section before selecting a response.

## Materials & experimental systems

| n/a | Involved in the study | |
|---|---|---|
| ☒ | ☐ | Antibodies |
| ☐ | ☒ | Eukaryotic cell lines |
| ☒ | ☐ | Palaeontology and archaeology |
| ☐ | ☒ | Animals and other organisms |
| ☒ | ☐ | Clinical data |
| ☒ | ☐ | Dual use research of concern |
| ☒ | ☐ | Plants |

## Methods

| n/a | Involved in the study | |
|---|---|---|
| ☒ | ☐ | ChIP-seq |
| ☒ | ☐ | Flow cytometry |
| ☒ | ☐ | MRI-based neuroimaging |

# Eukaryotic cell lines

Policy information about cell lines and Sex and Gender in Research

| | |
|---|---|
| Cell line source(s) | HEK-293T cells and human primary keratinocytes were obtained from ATCC. Human primary keratinocytes were cultured in Dermal Cell Basal Medium (ATCC PCS-200-030) supplemented with Keratinocyte Growth Kit (ATCC PCS200-040). A full-thickness reconstructed skin model (FTSK) was obtained from QIMA Bioalternatives. |
| Authentication | Cells were obtained from ATCC and were used according to supplier specifications. No additional authentication was performed by the authors after receipt. |
| Mycoplasma contamination | Cell cultures were routinely tested for mycoplasma contamination and were negative. |
| Commonly misidentified lines (See ICLAC register) | N/A |

# Animals and other research organisms

Policy information about studies involving animals; ARRIVE guidelines recommended for reporting animal research, and Sex and Gender in Research

| | |
|---|---|
| Laboratory animals | Male Sprague-Dawley rats (initial body weight 220 ± 10 g- aged 8 weeks) were obtained from the Animal Center of Nantong University. Animals were housed under controlled conditions (12-h light/dark cycle, 22 ± 2 °C, 50 ± 10% humidity) with ad libitum access to food and water. After one week of acclimatization, diabetes was induced by intraperitoneal streptozotocin (65 mg/kg). Rats with fasting blood glucose levels ≥16.6 mmol/L were included as diabetic models. |
| Wild animals | This study did not involve wild animals |
| Reporting on sex | Only male animals were used in this study. |
| Field-collected samples | This study did not involve field-collected samples |
| Ethics oversight | All animal procedures were approved by the Institutional Animal Care and Use Committee of Nantong University (Approval No. S20250522-004) and were conducted in accordance with the National Institutes of Health Guide for the Care and Use of Laboratory Animals. |

Note that full information on the approval of the study protocol must also be provided in the manuscript.

# Plants

| | |
|---|---|
| Seed stocks | N/A |
| Novel plant genotypes | N/A |
| Authentication | N/A |
